# Intelligent supervision of PIVAS drug dispensing based on image recognition technology

**DOI:** 10.1371/journal.pone.0298109

**Published:** 2024-04-04

**Authors:** Jianzhi Deng, Ying Chen, Xiaoyu Zhang, Yuehan Zhou, Bin Xiong

**Affiliations:** 1 College of Earth Sciences, Guilin University of Technology, Guilin, China; 2 College of Physics and Electronic Information Engineering, Guilin University of Technology, Guilin, China; 3 Department of Clinical Pharmacy, Guilin Medical University, Guilin, China; Yarmouk University, JORDAN

## Abstract

Pharmacy Intravenous Admixture Services (PIVAS) are places dedicated to the centralized dispensing of intravenous drugs, usually managed and operated by professional pharmacists and pharmacy technicians, and are an integral part of modern healthcare. However, the workflow of PIVAS has some problems, such as low efficiency and error-prone. This study aims to improve the efficiency of drug dispensing, reduce the rate of manual misjudgment, and minimize drug errors by conducting an in-depth study of the entire workflow of PIVAS and applying image recognition technology to the drug checking and dispensing process. Firstly, through experimental comparison, a target detection model suitable for drug category recognition is selected in the drug-checking process of PIVAS, and it is improved to improve the recognition accuracy and speed of intravenous drug categories. Secondly, a corner detection model for drug dosage recognition was studied in the drug dispensing stage to further increase drug dispensing accuracy. Then the PIVAS drug category recognition system and PIVAS drug dosage recognition system were designed and implemented.

## Introduction

Pharmacy intravenous admixture services (PIVAS), is in line with GMP standards, according to the characteristics of the drug designed for the operation of the environment, by the trained pharmacy technicians, in strict accordance with the operation procedures, including intravenous nutritional fluid, cytotoxic drugs and antibiotics and other intravenous drug dispensing, for clinical drug therapy and rational use of services. According to the Code of Pharmacy Management for Medical Institutions issued by the State Ministry of Health in 2002, medical institutions should establish intravenous drug dispensing centers (PIVAS) according to clinical needs [1] for the dispensing of anticancer chemotherapeutic drugs and total parenteral nutrition (TPN). The Code of Quality Management for Pharmacy Intravenous Infusion Services, issued by the Ministry of Health in 2010, also provides the requirements and standard operating procedures of PIVAS with a description. In recent years, significant progress has been made in the establishment of PIVAS, which has become an important part of hospital pharmacies [2]. PIVAS plays an important role in occupational safety by centralizing the dispensing of drugs that were previously distributed in different wards [3]. Centralized dispensing of intravenous drugs can maximize the avoidance of contamination and effectively ensure the safety of patients’ drugs, as well as improve the efficiency of hospital dispensing, reduce the workload of ward nurses and the risk of drug exposure, and promote the standardized management of hospitals [4]. However, once an error occurs in PIVAS, it will cause serious consequences, which not only cause drug wastage and affect the drug of a particular patient, but even group errors may occur [5].

**Fig 1:**
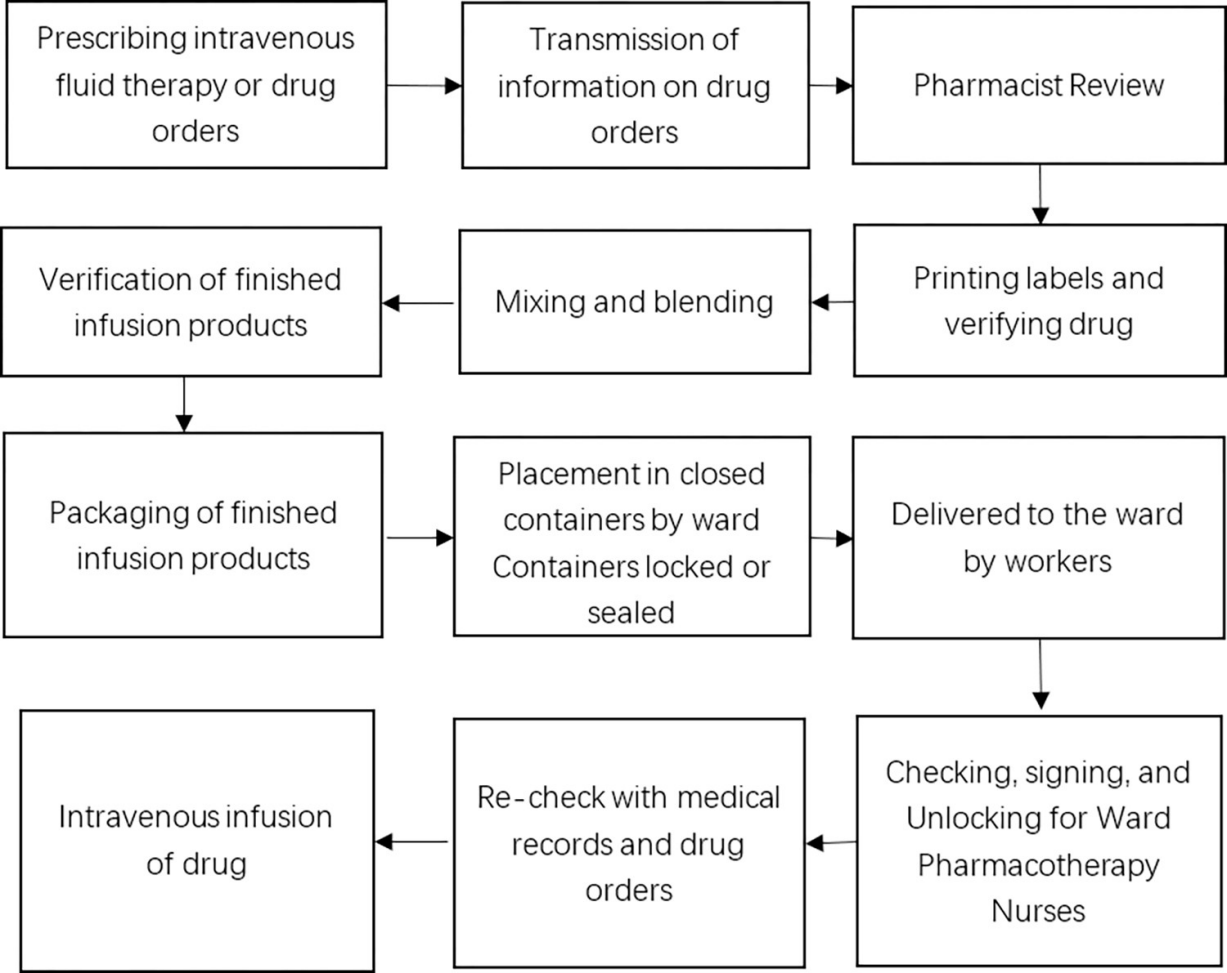
Workflow of the PIVAS.

As shown in Fig 1, the main workflow of PIVAS at this stage is done manually, which has many shortcomings. Firstly, there is a large workload in the process of drug-checking. At present, drug checking still relies on manual operations, which require a lot of time and manpower. Medical staff need to concentrate on checking, which can lead to operator fatigue and increase the risk of drug-checking errors. This poses a potential threat to the safety and efficacy of drug treatment, and may even lead to medical disputes [6]. According to data provided by the U.S. Food and Drug Administration, at least one patient dies every day as a result of drug-dispensing errors, and the number of patients harmed as a result is approximately 1.3 million per year [7]. Secondly, in the stage of drug dispensing, syringes during drug extraction may be affected by factors such as angle and light, resulting in the extraction amount not matching the expected dosage and increasing the risk of drug errors. However, it is difficult to determine the real precise drug extraction volume by just observing with the naked eye, coupled with the problems of observation angle and illumination, leading to errors in the actual drug dosage judgment. This can easily lead to a situation where the dose of the infused drug used does not match the dose required for clinical trials in healthy subjects, leading to adverse reactions in patients. Therefore, as the number of PIVAS increases, excellent management guidelines become critical in eliminating drug errors [8].

PIVAS standardization plays a key role in ensuring the safety of infusion products and reducing the burden on clinical nurses. During the COVID-19 pandemic in 2019, PIVAS not only ensured the supply of infusion products but also reduced the infusion preparation workload, enabling nurses to provide better quality care for COVID-19 patients [9]. Image recognition technology, as an inevitable need for modern medical development [10], is applied to PIVAS management to assist healthcare professionals in diagnosis and treatment, improve work efficiency, and prevent the occurrence of cross-infection. With the popularization of PIVAS and the increase in work intensity across the country, many hospitals have begun to utilize image recognition technology in different work segments to improve healthcare services. In the operation process of PIVAS, video monitoring equipment is usually used to record the complete process of drug dispensing for subsequent inquiries. However, this method cannot realize real-time monitoring and intelligent checking of the dispensing operation. With the continuous progress of computer technology, the application of image recognition in the medical field has become more and more extensive [11, 12], which has an important application value. Therefore, image recognition technology can be applied to PIVAS practical scenarios [13]. This method can help healthcare workers to complete the whole process of PIVAS more accurately and quickly, and effectively reduce the labor cost.

### Improvements in the process of drug-checking

Drug checking is the most important step in the entire PIVAS process. Its main purpose is to improve the accuracy and safety of drug dispensing and to improve the effect of drug treatment. In the process of drug-checking, healthcare workers need to accurately check the categories and quantities of drugs, minimize drug errors in drug dispensing, and ensure the safety and efficacy of drug treatment. However, in the process of drug-checking, the manual operation may waste a lot of labor resources and time, and at the same time, healthcare workers are prone to misjudging the drugs due to visual fatigue and repetitive labor, which may lead to errors in the subsequent processes such as drug dispensing and bring unnecessary risks and losses to patients. Therefore, the study introduces image recognition technology to improve the accuracy and efficiency of drug checking, reduce the interference of human factors, and protect the safety of patients’ drugs. In this chapter, a drug category recognition model applicable to the drug-checking process is investigated to improve the accuracy of intravenous drug dispensing.

### YOLOv5 network

**Fig 2:**
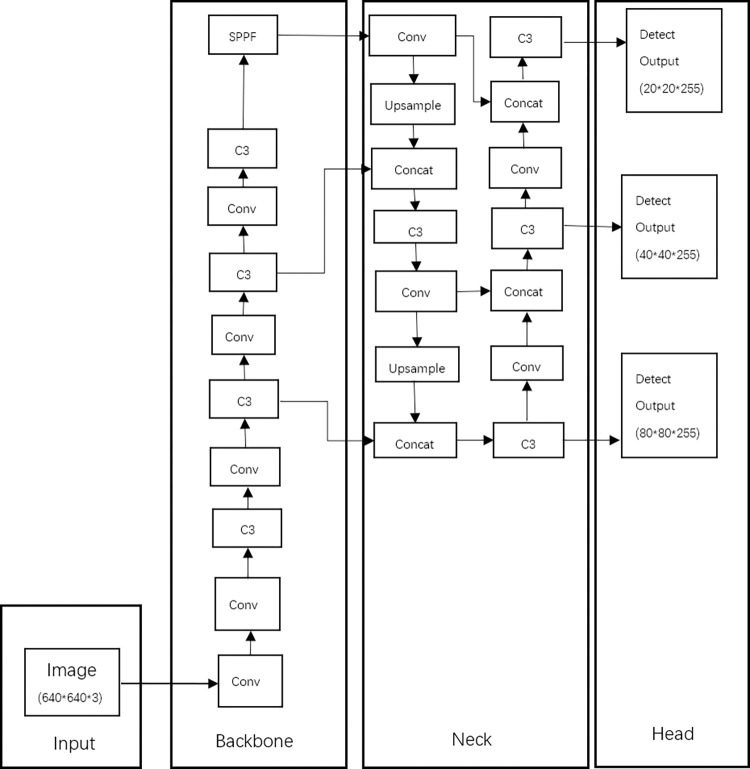
YOLOv5s network structure diagram.

YOLOv5 is characterized by high flexibility and speed [14]. Its network model structure, as shown in Fig 2, is composed of four parts: Input, Backbone, Neck, and Head. Input mainly preprocesses the input dataset, scaling the image to a fixed size of 640×640. The Backbone, mainly composed of C3, Conv, and SPPF modules, is used to extract rich information features from the input images. The Neck uses the PANet structure, which achieves the fusion of feature information at different scales. The Head is the detection structure of YOLOv5, outputting feature maps of three different sizes: large, medium, and small, which correspond to the detection of small, medium, and large targets respectively.

### Improved YOLOv5s model for drug category recognition

#### Data augmentation

**Fig 3:**
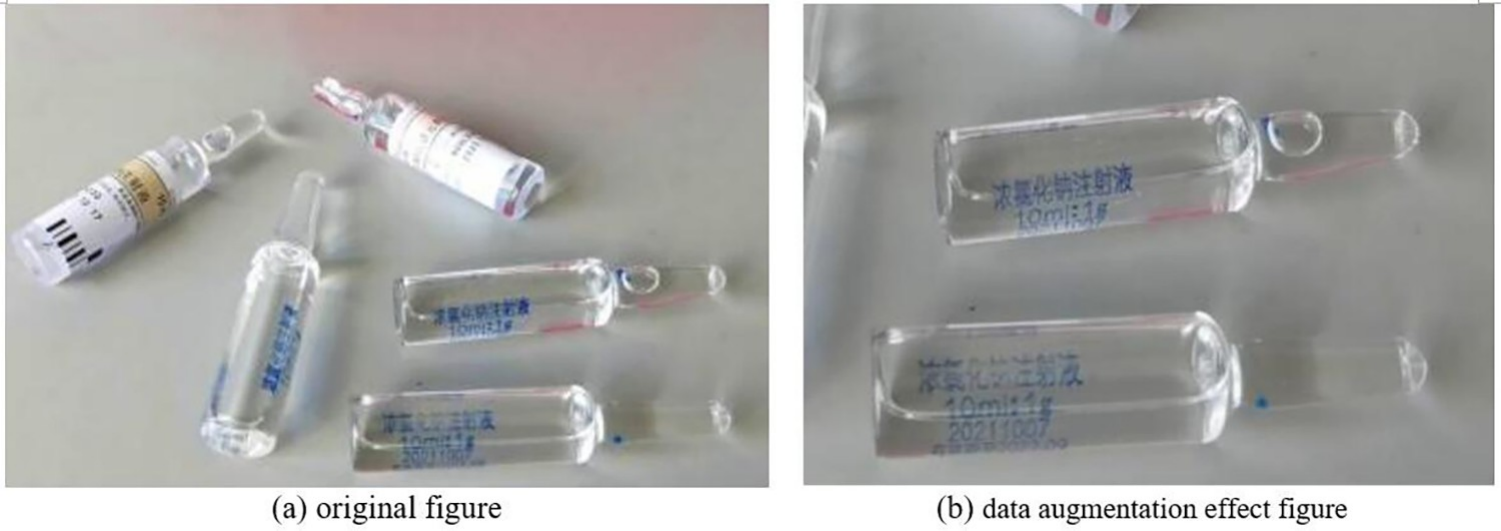
Results of data augmentation.

In the actual process of drug-checking, drug images are acquired through cameras, which usually have more noise and most photos contain multiple targets at the same time. In terms of improving the accuracy of drug recognition, preprocessing the images can appropriately reduce the redundant information and external interference, while increasing the number of desired targets and improving the quality of the dataset. Therefore, this study balances the number of different drug samples in the dataset by performing data augmentation on different drug data. Data augmentation methods include mirroring, rotation, cropping, color perturbation, translation, scaling, scale transformation, and contrast transformation [15]. These data augmentation techniques can increase the number and diversity of training data, while effectively reducing overfitting phenomenon and improving the robustness and stability of the model. The augmentation results are shown in Fig 3.

**Fig 4:**
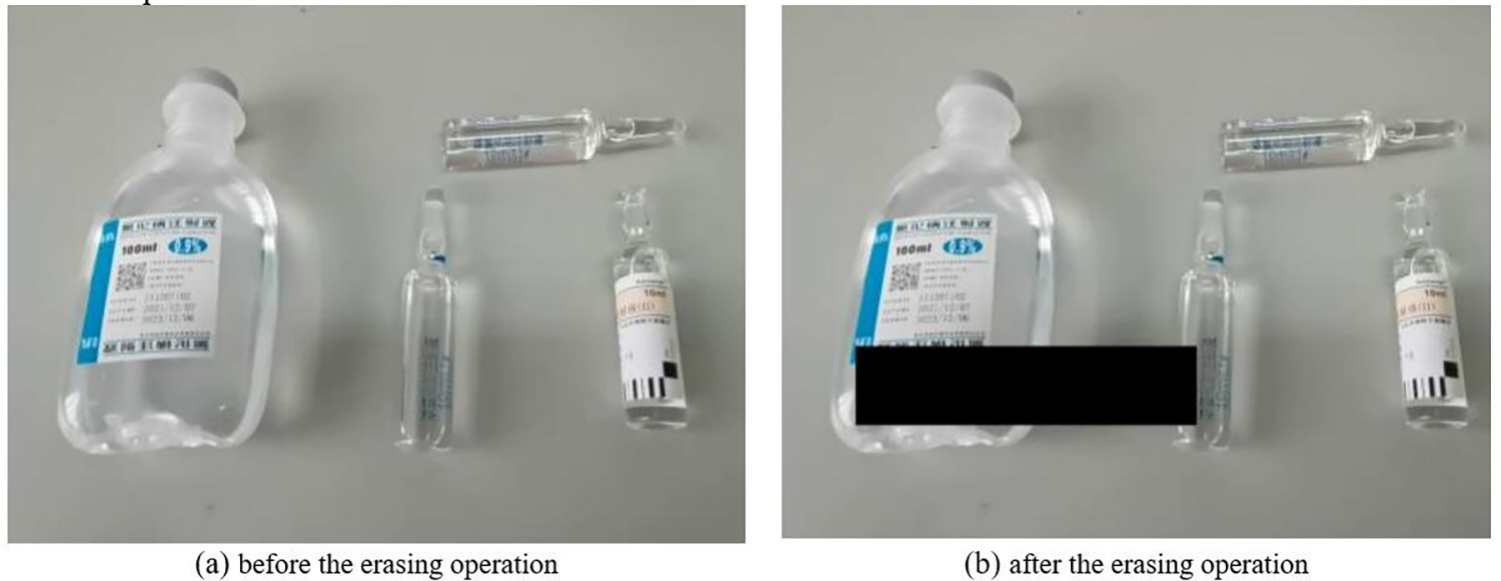
The comparison of the effects before and after the erasing operation.

Occlusion is an important factor that affects the generalization ability of CNS networks. In the process of drug-checking, drugs are not neatly placed and sometimes, they are even occluded by other drugs due to their random placement. In terms of improving the model’s ability to learn the target after occlusion, the study used a random erasure technique to process the drugs in the dataset to increase the number of samples with different levels of occlusion. This allows the model to better learn how to recognize occluded drugs and improve the accuracy of drug-checking in PIVAS scenarios. The erasure operation randomly removes a portion of pixels in the image, including the target and the background. Fig 4 shows the comparison of the effect before and after the erasure operation.

**Fig 5:**
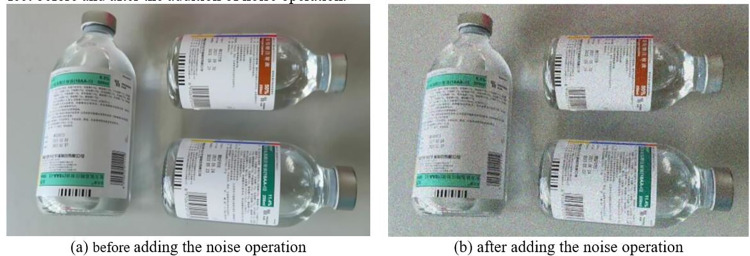
The comparison of the effects before and after adding the noise operation.

In terms of improving the ability of the target detection network to recognize drugs in complex scenes, this study applies Gaussian noise to some images in the dataset. These noises can simulate stains or slight absences on the images. By adding Gaussian noise, the model can be better adapted to the complex scene, thus improving the accuracy of drug recognition in the process of drug-checking. Fig 5 demonstrates the comparison of the effect before and after the addition of noise operation.

#### Improved C3Ghost

**Fig 6:**
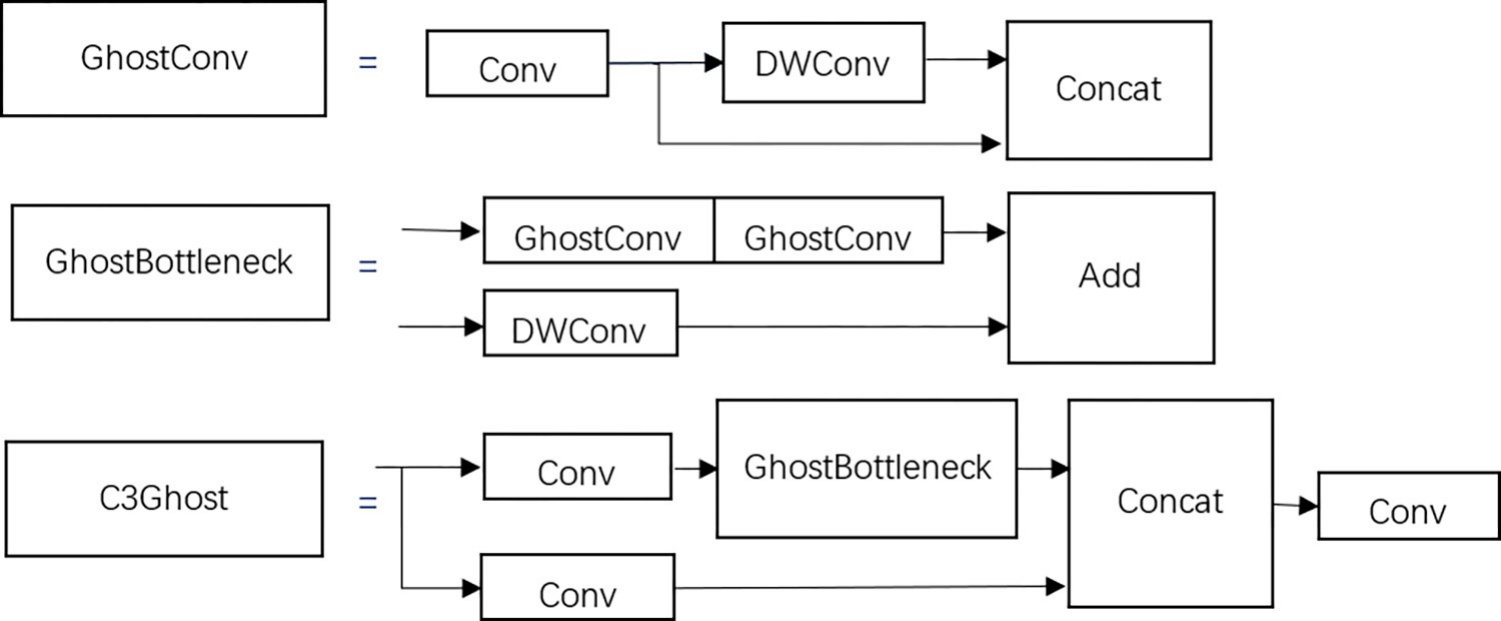
C3Ghost structure.

The YOLOv5 backbone feature extraction network uses the C3 structure, which has a large number of parameters and slow detection speed, limiting its application. The C3 structure, referring to the design of CSPNet [16], divides the input feature map into two parts, which are merged after their respective stage operations. To reduce the computational load and the number of parameters of the network, and to meet the model’s demand for detection speed, this section draws on the idea of the GhostNet module [17], integrating the GhostNet network into the C3 structure to form the new C3Ghost structure, as shown in Fig 6.

The improved GhostBottleneck reduces the number of channels of the input feature map by half through the first GhostConv, restores the number of feature map channels through the second GhostConv, and then adds and integrates features with the residual edge after 3x3 deep convolution. The GhostBottleneck replaces the Bottleneck in the original C3 module to form the C3Ghost module, reducing most of the traditional 3x3 convolutions in the original structure, compressing the model, and reducing the computational load, thereby improving the running speed.

#### CA attention module

In the whole process of PIVAS, commonly used infusion bag drugs are usually transparent packaging. However, in the process of target recognition, drugs in transparent packaging are often missed. This is because these drugs carry fewer valid pixels in their appearance, and after repeated feature extraction by convolution layers, information loss can easily occur. To improve the detection accuracy of the algorithm for targets of drugs in transparent packaging, we incorporate the CA attention module into the backbone network [18]. The algorithm of this attention module is described as follows:

Step 1: Input a feature map of shape [C, H, W].

Step 2: Perform global average pooling in the width and height directions on the feature map input from Step 1 to obtain two directional feature maps. After average pooling in the width direction, the obtained feature map shape1 is [C, H, 1], and after average pooling in the height direction, the obtained feature map shape2 is [C, 1, W].

Step 3: Transpose the width and height of shape1 and shape2 obtained in Step 2 to the same dimension, then stack them, merge the width and height features, and get the feature map: [C, 1, H+W]. Then they are sent into the shared convolution module with a kernel of 1×1, reducing its dimension to the original C/r, and get the feature map: [C/r, 1, H+W].

Step 4: Use convolution + normalization + activation function to process the feature map obtained in Step 3, and the obtained feature map is still: [C/r, 1, H+W].

Step 5: Divide the feature map obtained in Step 4 into: [C, 1, H] and [C, 1, W], and then transpose it. Obtain two feature maps [C, H, 1] and [C, 1, W].

Step 6: The two feature maps obtained in Step 5 respectively using the sigmoid method to obtain the attention situation on the width and height dimensions, and the results are merged to obtain the final feature map with attention weights on the width and height directions.

**Fig 7:**
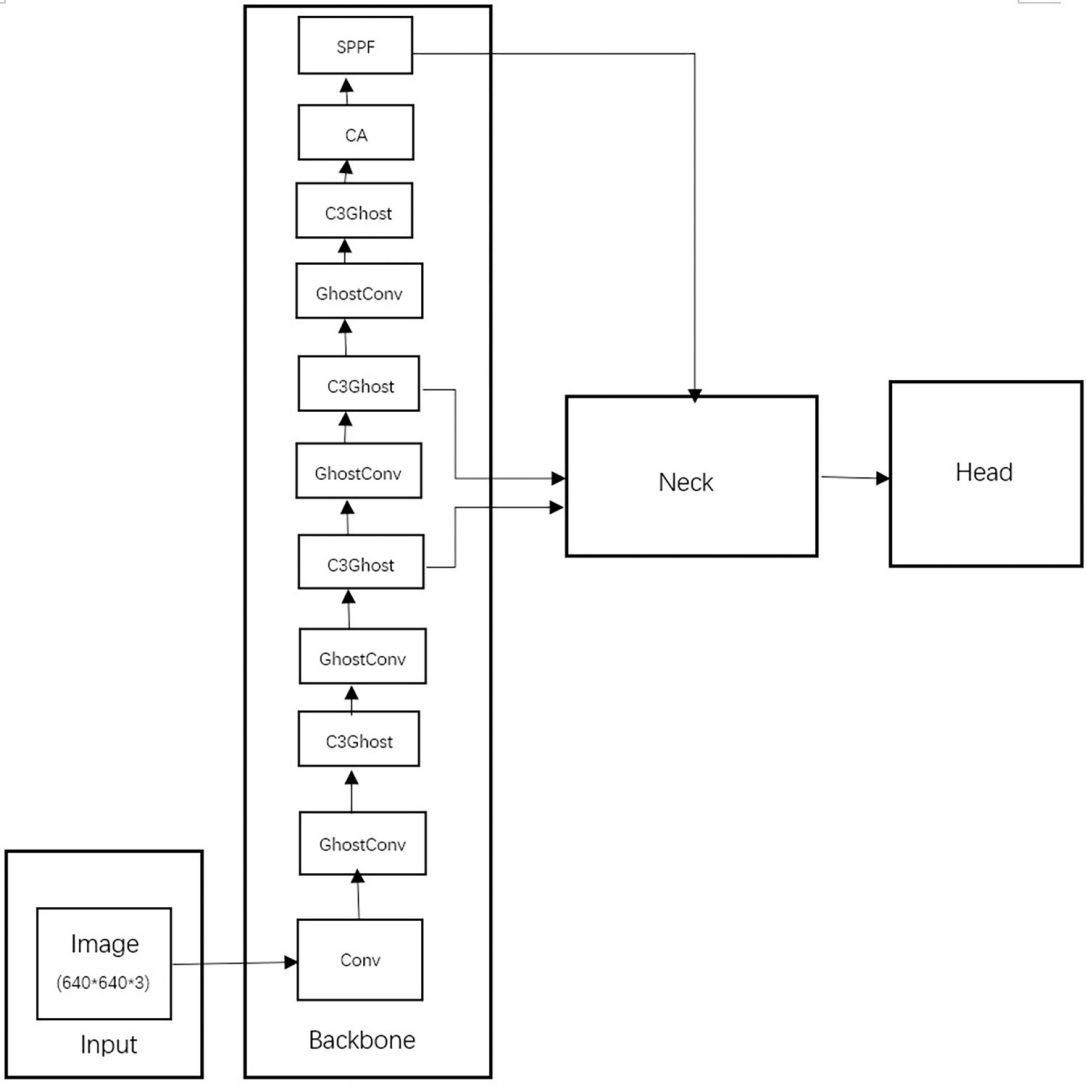
YOLOv5s backbone network of converged CA modules.

The CA attention module is introduced into the backbone network. This module can capture positional information from multiple directions, which can help the YOLOv5 model to locate and recognize the objects of interest more accurately. As shown in Fig 7, it can improve the positioning ability of drugs, thereby reducing the missed detection rate of the model. At the same time, the CA module is very convenient in application, it can be added after the convolutional layer at any time and complete the training, without considering the performance overhead brought by embedding it into the neural network.

#### Bi-FPN

Bi-FPN is a feature pyramid network architecture used for object detection. The main ideas are two points: efficient bidirectional cross-scale connections, and weighted feature map fusion. To solve the problem that drugs with similar appearance are wrongly detected as the same drug, this study uses the idea of a Bi-FPN network [19] for reference, improves the feature fusion network, increases the information coupling between different scales, and enables the model to better perform multi-scale feature fusion. In terms of adapting Bi-FPN to the YOLOv5s network for 3-scale detection, the five input feature layers in Bi-FPN are simplified to three. For different scales of information, the feature resolution scales are unified by upsample and downsample, and then the results obtained from the bottom up are fused with the initial input feature map for feature fusion, and then a new round of fusion is performed with the results obtained from the top down to reduce the loss of feature information. This further improves the positioning ability for transparent drugs.

### Experimental results and analyses

#### Experimental datasets

Since little research has been done on drug recognition in PIVAS scenarios, there is no publicly available drug dataset applicable to this scenario. Therefore, in terms of accomplishing the task of recognizing drug categories in PIVAS scenarios, it is necessary to collect drug pictures to make a dataset by ourselves. In terms of making the positive and negative samples in the dataset as balanced as possible, with higher model accuracy and better detection effect, this section adopts multi-angle, multi-direction, multi-placement and other ways to take pictures of the target drugs in different backgrounds and under different lighting, and collects a total of 5,000 pictures of the drugs in the PIVAS scenario, which contain a variety of drugs such as sodium chloride injection, potassium chloride injection and so on, all of which are labeled manually by using the LabelImg All of them were manually labeled using LabelImg, and divided into training set and test set according to the ratio of 7:3.

#### Experimental settings

The experimental environment of the study mainly includes an NVIDIA RTX 2060 graphics card with 6GB video memory, an Intel i7-10875H processor running at 2.3GHz, Python3.8 for the development environment, CUDA11.3, and Pytorch 1.8.1 for the deep learning framework. The training is divided into two phases, the freezing phase and the unfreezing phase: in terms of preventing the weights from being destroyed, at the beginning of training, we choose to freeze a part of the training first to speed up the training, train 50 rounds, and the number of images passed into the network is 8 each time; after unfreezing, the learning rate becomes 0.0001, and the number of images passed into the network becomes 4.

#### Evaluation indicators

The performance evaluation of detection models usually needs to consider both detection accuracy and inference speed. Detection precision can be evaluated using metrics such as AP (Average Precision) and mAP (mean Average Precision), which can help us choose the most suitable detection model for our application scenarios.AP is the average precision of the model under different recall rates, and mAP is the multi-class average precision. mAP is the average precision of multiple classes. The higher the mAP value, the better the model performance. The higher the mAP, the better the model performance. Inference speed is usually measured by FPS (Frames Per Second), i.e., the number of frames the model can process per second. In a real-world checking session, drug category recognition requires considering the balance between accuracy and speed and selecting a suitable detection model. This means that a trade-off between accuracy and processing speed needs to be made to ensure optimal performance and efficiency.

The formula for mAP is given in Eqs (1)–(4).

$$mAP=\frac{\sum_{i=0}^{N}AP_{i}}{N}$$ (1)

$$AP=\int_{0}^{1}p(r)dr$$ (2)

$$P=\frac{TP}{TP+FP}$$ (3)

$$R=\frac{TP}{TP+FN}$$ (4)

where TP represents the number of accurately detected drugs, FP represents the number of falsely detected drugs, FN represents the number of undetected drugs, P represents the precision rate, R represents the recall rate, N represents the total number of categories, and *i*. represents the current category. For each category, the average precision (AP) is the area below the corresponding precision-recall (PR) curve.

#### Comparative experimental analysis of drugs

To verify the effectiveness of the improved algorithm, this study conducted an experimental comparative analysis of the detection accuracy of different drug targets in the drug dataset. The experimental results are shown in Tables 1 and 2. The model’s merits are judged by comparing the performance indicators of the two algorithms such as AP, mAP, and FPS.

**Table 1:** Comparison results of transparent drug data algorithms.

algorithm	AP				FPS	mAP
	NaCl	NaCl+	sodium	m trace elements		
YOLOv5s	0.8178	0.7335	0.7078	0.7635	45.6	0.9232
improved algorithm	0.9326	0.8527	0.7754	0.8217	51.6	0.9531

**Table 2:** Comparison results of similar drug data algorithms.

algorithm	AP				FPS	mAP
	KCl	sterile	glucose	glucose+		
YOLOv5s	0.7275	0.8962	0.7335	0.7781	45.6	0.9232
improved algorithm	0.8167	0.9208	0.8527	0.8365	51.6	0.9531

It can be concluded from Tables 1 and 2 that the improved algorithm has demonstrated good detection performance in both transparent drug data and similar drug data, effectively reducing the false detection rate and missed detection rate of drugs, achieving a good balance between recognition speed and recognition rate, thereby making the overall detection performance superior. The improved model has shown stronger generalization ability on the drug dataset, which has certain positive significance for subsequent work research.

**Fig 8:**
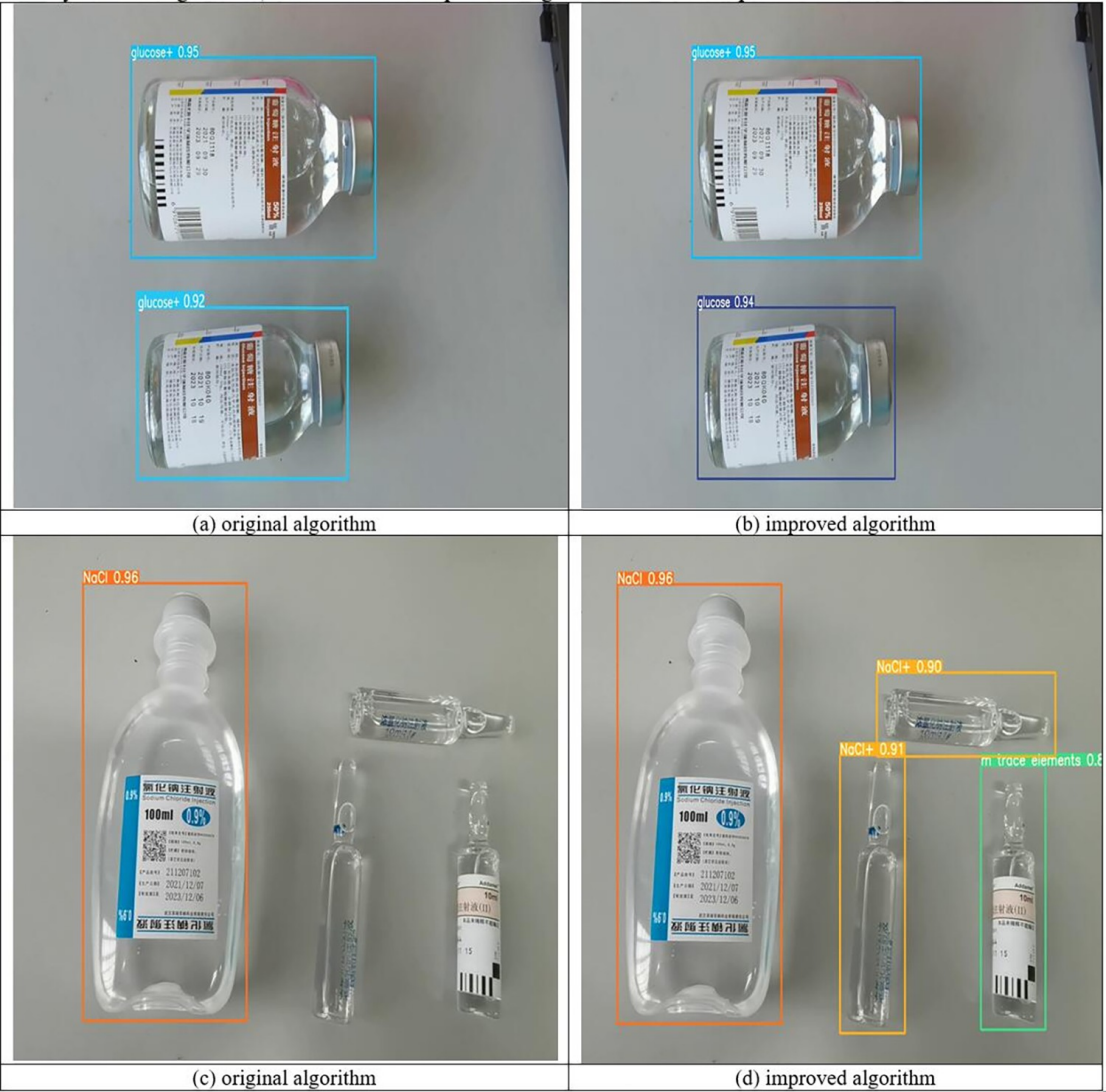
Comparison of the recognition effect of the improved algorithm and the original algorithm.

The study used two sets of PIVAS scene pictures containing multiple drugs for drug recognition, as shown in Fig 8. It can be seen from Fig 8(A) that the model before the improvement mistakenly detected two different volumes of glucose injection as the same volume of glucose injection. This is because the packaging of these two drugs is very similar and is easily recognized by the model as the same drug. However, it can be seen from Fig 8(B) that the improved model can accurately recognize two different volumes and different categories of drugs. This shows that the model proposed in this study can accurately recognize drugs and reduce the false detection rate of similar drugs. In addition, it can be seen from Fig 8(C) that the model before the improvement missed the detection of the drug due to the high transparency of the packaging of the hypertonic sodium chloride injection. However, it can be seen from Fig 8(D) that the improved model can accurately recognize the hypertonic sodium chloride injection. This fully demonstrates that the model proposed in this study can reduce the missed detection rate of transparent drugs and improve the positioning ability of drugs. The comparison results show that the improved model has universality in recognizing intravenous drugs.

#### Ablation experiment

To verify the optimization effect of the improvement strategy on YOLOv5s, the study adds different modules such as GhostNet, CA attention module, and Bi-FPN structure to YOLOv5s in terms of comparison and checking. In terms of conducting the experiments, the settings of the training parameters were kept consistent to ensure the comparability of the experimental results. Through these experiments, it is possible to clearly understand the degree of performance enhancement of YOLOv5s by each module, to better optimize the algorithm. The results of the ablation experiments of the studied algorithms on the drug dataset are shown in Table 3, which records the FPS, mAP, and model size of the model with the addition of different modules.

**Table 3:** Results of ablation experiments.

algorithm	FPS	mAP	Model Size
YOLOv5s	45.6	0.9232	14.1MB
YOLOv5s+GhostNet	53.1	0.8986	12.2MB
YOLOv5s+GhostNet+CA	52.6	0.9127	12.4MB
improved algorithm	51.6	0.9531	12.7MB

As shown in Table 3, the model size of the network with GhostNet module is 12.2M, which is 1.9M less than that of the original network, and its detection speed reaches 53.1FPS, which is 16.4% higher than that of the original network, which indicates that the GhostNet module has the effect of lightweight and improving the detection speed. After incorporating the CA attention module, the mAP value increases by 1.41%. This indicates that the CA attention module can accurately localize the position of the drug target and improve the accuracy of drug detection. Finally, the use of a Bi-FPN structure instead of a PANet feature fusion network makes the mAP value of the model reach 95.31%, which is improved by 4.04%. The experimental results demonstrate that all the improvements proposed in this paper contribute to the network performance.

#### Comparison of the effectiveness of different detection models

In terms of validating the effectiveness of the improved algorithm, the study conducted performance comparison experiments using the improved YOLOv5s algorithm on the intravenous drug dataset and compared it with the original YOLOv5s and the current mainstream target detection models. The experimental results are shown in Table 4.

**Table 4:** Comparative experiments with different detection models.

algorithm	FPS	mAP	Model Size
Faster R-CNN	14.3	0.7632	109MB
YOLOv3	22.6	0.8626	235MB
YOLOv4	24.1	0.9053	244MB
YOLOv5s	45.6	0.9232	14.1MB
improved algorithm	51.6	0.9531	12.7MB

The experimental results demonstrate that the improved model has obvious advantages in detection accuracy, speed, and model size compared with several mainstream target detection models. Compared with the two-stage algorithm Faster R-CNN, it is not only 18.99% ahead in average detection accuracy, but also significantly improves in detection speed; compared with the single-stage algorithms YOLOv3 and YOLOv4, it is not only 9.05% and 4.78% ahead in average detection accuracy, but also improves in detection speed by 29FPS and 27.5FPS. Especially in terms of model size, the improved model is much smaller than these three target detection algorithms. Compared with the pre-improved YOLOv5s network, the mAP value of the improved model increases by 2.99% and still maintains a high detection speed, which can meet the requirements of the real-time detection task, which demonstrates the feasibility of the proposed model for pharmaceutical target recognition.

### Improvements in the process of drug-dispensing

The drug dispensing needs to be strictly by the prescription requirements to ensure the accurate ratio of the drug and also needs to be personalized according to the specific situation of the patient to ensure the effectiveness and safety of the drug. In the process of drug dispensing, pharmacists need to accurately check the dosage of drugs and strictly implement the operating procedures to ensure the quality and safety of drugs. Accurate checking of drug dosage is a key part of the drug preparation process. In the process of drug dispensing, healthcare professionals usually use syringes to extract drugs. However, the syringe may be affected by factors such as angle and light during the process of drug extraction, resulting in discrepancies between the extracted amount and the expected dosage, and the checking of drug dosage relies entirely on manual identification, which can easily lead to errors in drug dosage, affecting the accuracy and safety of drug dispensing, and may even lead to drug disputes. To improve the precision and safety of drug dispensing, this chapter has studied a drug dosage recognition model. This model accurately identifies the dosage of the drug by processing and analyzing the syringe images during the drug extraction process and performing a check.

### Problem analysis

In the process of drug-dispensing, healthcare professionals use syringes to draw drugs and cannot directly recognize the volume of the drug. Therefore, it is investigated to obtain the volume of drug extraction by recognizing the position of the piston in the syringe. However, two problems need to be solved to accurately recognize the volume of the drug in the syringe: the first problem: in the actual process of drug dispensing, the position of the syringe may be randomly placed, resulting in the back of the syringe’s scale surface to the instrument, and the scale on the surface of the syringe could not be obtained, which resulted in the study not being able to accurately recognize the volume of the drug in the syringe by using methods such as target detection. In addition, the darker color of the drug also affects the clarity of the scale, causing interference with methods such as target detection. The second problem: the position of the black piston in the syringe may be between the black scales, which can lead to a subjective judgment of the amount of medicine to be used, making it impossible to determine the actual amount to be drawn, or even mistakenly believing that the actual amount to be drawn is in line with the expected amount. This kind of subjective judgment may lead to incorrect checking of drug dosage, which may result in drug contamination and waste, and ultimately affect the safety and efficacy of drug therapy. To address the above problems, the study adopts the corner detection model to obtain the coordinates of the syringe apex and the piston position and calculates the ratio of the actual extracted dosage to the total syringe capacity to accurately calculate the actual dosage of drugs. This method can solve the problem of recognizing the syringe scale being occluded. At the same time, the placement position of the syringe and the color of the medicine will not affect the calculation of the medicine dosage.

### Image preprocessing

The commonly used infusion drugs in PIVAS are divided into colorless drugs and colored drugs. Colored drugs are easy to recognize because they have a distinct color and do not blend in with the background. However, in PIVAS scenarios, colorless drugs are more common. Since colorless drugs tend to blend in with the background, it is difficult to recognize the edges of the drug in the syringe, which makes it difficult for the corner detection model to accurately measure the volume of the drug. In addition, in practice, the background noise is strong and the lighting conditions are complex, which also affects the accuracy of the corner detection model and may lead to the loss of the true corner or the appearance of pseudo-corner. This affects the calculation of the true extraction volume of the drug. In terms of more accurately recognizing the volume of drugs in the syringe, the study uses image preprocessing techniques such as binarization, morphological processing, and Gaussian smoothing filtering. These techniques can effectively remove noise and interference and accurately recognize the amount of drug extraction.

#### Binarization process

The study begins with binarization of the image to extract the syringe contour from the image. In terms of obtaining an ideal binarized image, the threshold segmentation technique is generally used. However, a lot of information will be lost if not used properly in the binarization process, the key lies in determining an appropriate gray scale threshold [20] to divide the image into two parts, one is the target and the other is the background. This process can help us better recognize and analyze the target object in the image and improve the efficiency and accuracy of image processing. Therefore, it is very important to choose the appropriate binarization method and grayscale threshold. In practical applications, the threshold needs to be dynamically adjusted according to the specific situation to obtain the best results.

The study uses the maximum inter-class variance method [21] to segment the image. Its basic principle is to divide the image into two classes such that the intra-class variance is minimized and the inter-class variance is maximized. This method is suitable for the situation that there are obvious foreground and background in the image, and can effectively separate them. In a specific implementation, the image is divided into two parts by calculating the grayscale histogram to select a suitable threshold. Then the mean and variance of the two parts are calculated and the threshold that maximizes the variance between classes is selected as the final segmentation result.

It is known that the grayscale image F, whose full-image gray value is (0, *L*-1), the grayscale is L, and the image is of *N*×*M* resolution, and let *n_i_* pixel points of grayscale *i*, and P*_i_* be the probability of the occurrence of a pixel point of that grayscale i.e:

$$P_{i}=\frac{n_{i}}{N\times M},\;P_{i}\geq 0,\;\sum_{i=0}^{L-1}P_{i}=1$$ (5)

Set the segmentation threshold *k* ⊂ [0, *L*-1], according to this threshold the image is divided into two categories *C*_0_ and *C*_1_ and the segmentation result can be expressed as:

$$F(x,y)=\{\begin{array}{ll} C_{0} & i<k\\ C_{1} & i\geq k \end{array}$$ (6)

Where *C*_0_ represents the image target and *C*_1_ represents the image background. The grayscale less than k is contained in *C*_0_, and vice versa in *C*_1_, *W*_0_ and *W*_1_ denote the probability of the occurrence of *C*_0_ and *C*_1_ respectively; and the mean value of the gray scales of *C*_0_ and *C*_1_ are denoted as *μ*_0_ and *μ*_1_.

The *C*_0_ and *C*_1_ variances can be calculated as:

$$\sigma_{0}^{2}=\sum_{i=0}^{k}{\left(i-\mu_{0}\right)}^{2}\frac{P_{i}}{\omega_{0}}$$ (7)

$$\sigma_{1}^{2}=\sum_{i=k+1}^{L-1}{\left(i-\mu_{1}\right)}^{2}\frac{P_{i}}{\omega_{1}}$$ (8)

The between-class variance of the target and background is:

$$\sigma_{k}^{2}=\omega_{0}\omega_{1}{\left(\mu_{1}-\mu_{0}\right)}^{2}$$ (9)

The size of the $\sigma_{k}^{2}$ value indicates the degree of pixel difference between *C*_0_ and *C*_1_ and the quality of the separation effect. When $\sigma_{k}^{2}$ is the maximum value, k is the optimal image segmentation threshold.


$$k=Arg_{\max}\{\sigma_{k}^{2}(k)\},0\leq k\leq L$$ (10)

#### Morphological processing

After threshold segmentation, the syringe image may have holes due to the presence of surface texture. In terms of solving this problem, hole filling operation needs to be performed on the transformed binary image. By this method, the syringe image can be made clearer for better subsequent image processing and analysis.

Morphological processing is a commonly used image processing method, which includes operations such as open operation, closed operation, and morphological gradient. Among them, the computational order of the open operation is corrosion followed by expansion. Smaller noise can be removed by the erosion process, and then the gaps caused by excessive erosion are filled by expansion. As a result, the open operation can effectively remove isolated points in the syringe image, making the edge line smoother. This method can effectively deal with the noise and edge lines in the syringe image, making that image clearer and more accurate. The closed operation first performs the expansion operation and then performs the erosion process. This method can connect two very close elements without much effect on the shape and area outside the connection area. With the closed operation, the syringe image can be smoothed and some small noises can be removed, resulting in a clearer image. Both operations have the same input parameters. The results of open and closed operations on the same set of map processing are very different, the open operation will eliminate the small noise in the image, while the closed operation will expand the connected area in the process, which needs to choose the appropriate operation according to the specific application scenario.

In the study, the morphological open operation [22] is first used to remove smaller noise in the image, which can effectively improve the quality and clarity of the syringe image. Then, the image is processed using closed operations, which can connect the narrow gaps in the syringe image and fill the holes that are smaller than the structural elements, thus smoothing the contour of the object and making it more continuous and fluid.

#### Gaussian smoothing filter

Although a continuous and smooth syringe image can be obtained by the previous image processing, the image will still have some noise, which will interfere with the quality of the image. In terms of eliminating the noise in the image, a Gaussian filter is used in this section to smooth the image [23] to suppress the effects of noise and texture on the image and to make the gradient of the image smooth and continuous. In this way, a clearer and more natural syringe image can be obtained. Gaussian filtering is done by scanning each pixel in the image using a template. The template is a fixed-size matrix, usually 3x3 or 5x5 in size. During the scanning process, the pixel points in the center of the template are replaced with a weighted average gray value of the pixels in the neighborhood. This weighted average is computed using a Gaussian function, which is a commonly used probability distribution function that assigns pixel weights to pixels within the neighborhood.

The Gaussian kernel in two dimensions is:

$$G(x,y)=\frac{1}{2\pi \delta^{2}}e^{-\frac{x^{2}+y^{2}}{2\delta^{2}}}$$ (11)

Let the original image be *f*_0_(*x*,*y*), then the smoothed image is:

$$f(x,y)=f_{0}(x,y)*G(x,y)$$ (12)

Where, * denotes convolution.

### Preprocessing results

**Fig 9:**
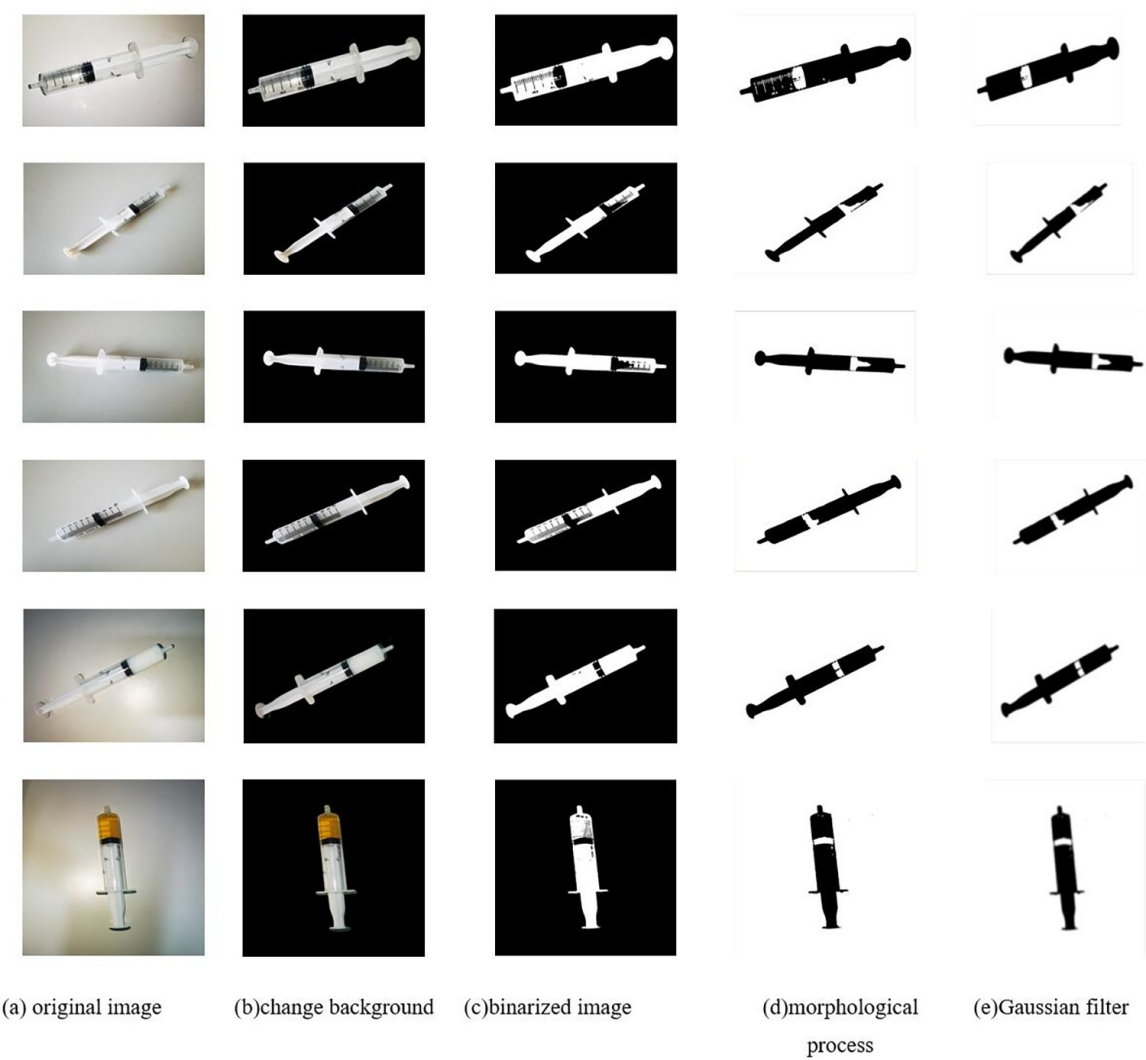
Image preprocessing results.

This study changed the background color of the image according to the unique properties of the syringe and used a series of image preprocessing techniques such as binarization, morphological processing, and Gaussian smoothing filtering to effectively remove noise and interference and obtain smooth, continuous, and clear syringe images. The processing results are shown in Fig 9. The application of these techniques improves the accuracy of subsequent corner detection operations, can accurately extract the required corner coordinates, and thereby improves the accuracy of drug dosage calculation.

### Corner detection

Corners are pixels in an image with high variation, and these pixels are usually the significant feature points in the image [24]. Corner detection is a common technique in computer vision, often used in applications such as image matching, target tracking, 3D reconstruction, etc. Common corner detection algorithms include the Harris corner detection algorithm [25], the FAST corner detection algorithm [26], and the SIFT feature point detection algorithm. The suitable corner detection algorithm should be determined according to the specific application scenario and performance requirements.

The purpose of the image recognition study is to perform geometric measurements and calculate the actual extraction volume of the drug, not to extract feature points for recognition. Therefore, when processing images, it is necessary to exclude those pixels that are not corners as much as possible to ensure that they do not participate in the calculation process.

The study uses the Harris algorithm to detect corners in the image to accurately locate the real corner positions and extract their coordinates. The specific calculation method is to use the self-correlation function of the gradient in the horizontal and vertical directions of the image to obtain the self-correlation matrix M, and then calculate the two eigenvalues of the matrix M. When both eigenvalues are high, the point can be considered as a corner. This can be simply divided into three steps. First, you need to calculate the gradient value of each pixel in the image. Then, use these gradient values to calculate the self-correlation matrix M of each pixel. Finally, by calculating the eigenvalues of the matrix M, it is judged whether the point is a corner. When the grayscale around a pixel changes greatly, it may be a corner. Therefore, only when the two eigenvalues of the matrix M are very high, can it be explained that the grayscale changes greatly around the point, and it is judged that the point is a corner. The calculation formula is:

$$\begin{array}{l} E_{u,v}(x,y)={\sum_{u,v}w_{u,v}[I(x+u,y+v)-I(x,y)]}^{2}\\ ={\sum_{u,v}w_{u,v}[u\frac{\partial I}{\partial y}+v\frac{\partial I}{\partial x}+o(\sqrt{u^{2}+v^{2}})]}^{2}\\ =\left[\begin{array}{c} u\\ v \end{array}\right]M[\begin{array}{cc} u & v \end{array}] \end{array}$$ (1)

$$M=[\begin{array}{cc} \sum_{u,v}I_{x}{\left(u,v\right)}^{2} & \sum_{u,v}I_{x}(u,v)I_{y}(u,v)\\ \sum_{u,v}I_{x}(u,v)I_{y}(u,v) & \sum_{u,v}I_{y}{\left(u,v\right)}^{2} \end{array}]$$ (2)

$$CRF(x,y)=\det(M)-k{\left[tr(M)\right]}^{2}$$ (3)

Then according to the obtained coordinates, calculate the distance between (*x*_1_, *y*_1_) and (*x*_2_, *y*_2_) and the distance between (*x*_1_, *y*_1_) and (*x*_3_, *y*_3_), and then launch the ratio of the actual extraction amount to the total capacity of the syringe through the ratio of the two distances on the image to get the actual extraction amount of the medicine. The calculation formula is as follows:

$$L=\frac{\sqrt{{\left(x_{2}-x_{1}\right)}^{2}+{\left(y_{2}-y_{1}\right)}^{2}}}{\sqrt{{\left(x_{3}-x_{1}\right)}^{2}+{\left(y_{3}-y_{1}\right)}^{2}}}\times R$$ (4)

In the formula: R is the actual extraction volume of the drug. (*x*_1_, *y*_1_) and (*x*_3_, *y*_3_) are the vertex coordinates of the syringe image, (*x*_2_, *y*_2_) is the position coordinate of the piston in the syringe.

**Fig 10:**
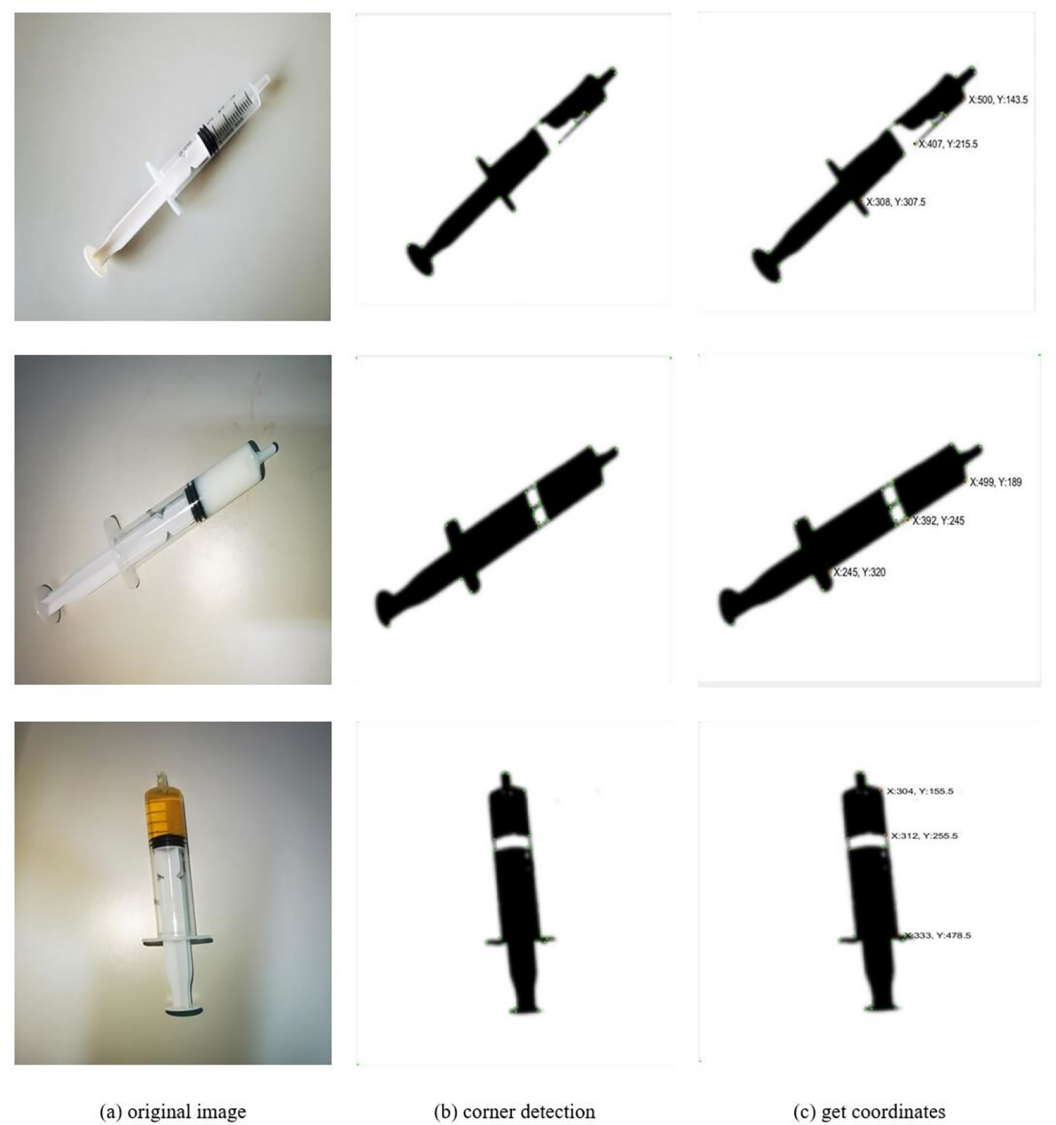
Harris corner detection after image.

The result is shown in Fig 10, the study performs Harris corner detection on the previously processed image, and through the calibration program, displays the corner coordinates of the piston position in the syringe and the vertex coordinates of the syringe image. Then, by calculating the ratio of the two distances from the coordinates of the three points on the image, the ratio of the actual extraction volume to the total volume of the syringe is deduced.

### Results and analyses

In terms of verifying the accuracy and universality of the drug dosage recognition model, this paper has done comparative experiments of drug dosage recognition in different scenarios. The experimental results are shown in Tables 5 and 6. In the table, groups 1–4 is the control group, and the scale of the syringe is visible, group 5–10 is the experimental group, in which the black piston position of the syringe in groups 5 and 6 is located between the scales and the position is blurred, the scale in the syringe in groups 7 and 8 is affected by the light and the angle and the scale is not clear, and the scale in the syringe in group 9 and 10 is occluded. The "None" in the table means that it is not observable by human eyes. Tables 5 and 6 records the recognized dosages of colorless and colored drugs after drawing from syringes of different capacities.

**Table 5:** Comparative experiment table for recognizing the dosage of colorless drugs.

	5ml capacity syringe			10ml capacity syringe			20ml capacity syringe		
	expected dosage	human eye observation	research method	expected dosage	human eye observation	research method	expected dosage	human eye observation	research method
1	2	2	2.00	5	5	5.00	10	10	10.00
2	2.4	2.4	2.40	5.6	5.6	5.60	11.5	11.5	11.50
3	3	3	3.00	6	6	6.00	12	12	12.00
4	3.2	3.2	3.20	7.4	7.4	7.4	12.5	12.5	12.50
5	3.3	3.3	3.30	8.1	8	8.08	12.8	12.5	12.78
6	3.7	3.6	3.70	8.5	8.5	8.51	13.6	13.5	13.60
7	3.5	None	3.49	8.2	None	8.20	13.5	None	13.50
8	4	None	4.00	8.5	None	8.48	14	None	14.00
9	4.5	None	4.52	9.2	None	9.20	15.5	None	15.50
10	4.6	None	4.60	9.5	None	9.52	17.8	None	17.78

**Table 6:** Comparative experiment table for recognizing the dosage of colored liquids.

	5ml capacity syringe			10ml capacity syringe			20ml capacity syringe		
	expected dosage	human eye observation	research method	expected dosage	human eye observation	research method	expected dosage	human eye observation	research method
1	1	1	1.00	6	6	6.00	11	11	11.00
2	1.6	1.6	1.60	6.4	6.4	6.40	12.5	12.5	12.50
3	2	2	2.00	5	5	5.00	10	10	10.00
4	2.5	2.4	2.51	7.7	7.8	7.69	13.7	14	13.60
5	2.7	2.6	2.72	8.3	8.3	8.29	14.2	14.5	14.20
6	3.5	3.6	3.50	8.5	8.5	8.51	13.6	13.5	13.60
7	3.7	None	3.70	8.8	None	8.81	14.2	None	14.20
8	4	None	4.00	9.2	None	9.20	15.7	None	15.68
9	4.3	None	4.30	9.5	None	9.52	16.6	None	16.60
10	4.5	None	4.52	9.3	None	9.29	18.5	None	18.50

As seen in Tables 5 and 6, when the syringe scales are visible, both the research method and human eye observation can accurately recognize the drug dosage. However, when the position of the black piston of the syringe is between the scales, the human eye cannot accurately determine the dosage of a drug and can only rely on subjective judgment. In this case, the drug dosage obtained may have a larger error than the expected dosage. In contrast, the method proposed by the research can obtain the drug dosage through calculation. The error between the drug dosage recognized by this method and the expected dosage is relatively small. Furthermore, when the syringe scale is affected by lighting, angles, or is blocked, the human eye cannot obtain the drug dosage, but the method proposed by the research can still accurately recognize the drug dosage. This shows that the methodology used in the study is applicable and generalizable for drug dosage recognition in different scenarios.

### System design and implementation

**Fig 11:**
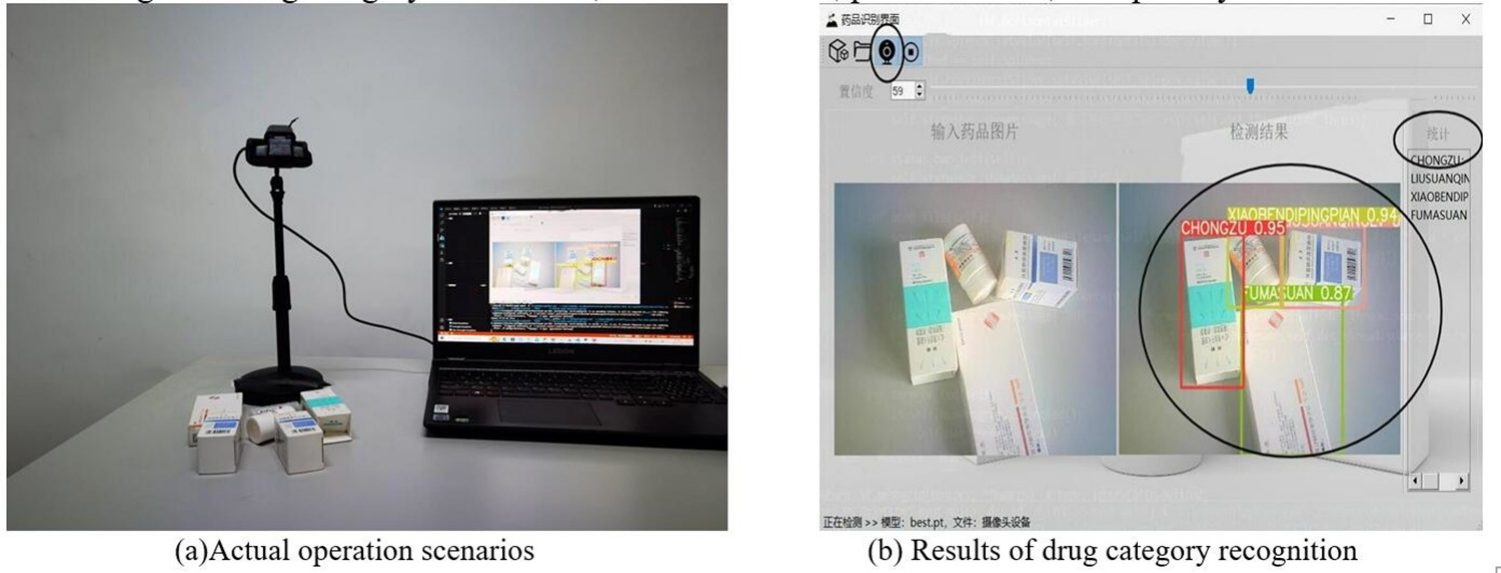
Real-time recognition scenarios.

Fig 11(A) shows the actual operation scene of the drug-checking process. In this scenario, the drugs to be recognized are placed under the camera, and the camera above the workbench will automatically capture the images of the drug packaging, and the recognized drug information will be displayed on the computer interface. Fig 11(B) shows the recognized drug category information, confidence level, prediction box, and quantity.

**Fig 12:**
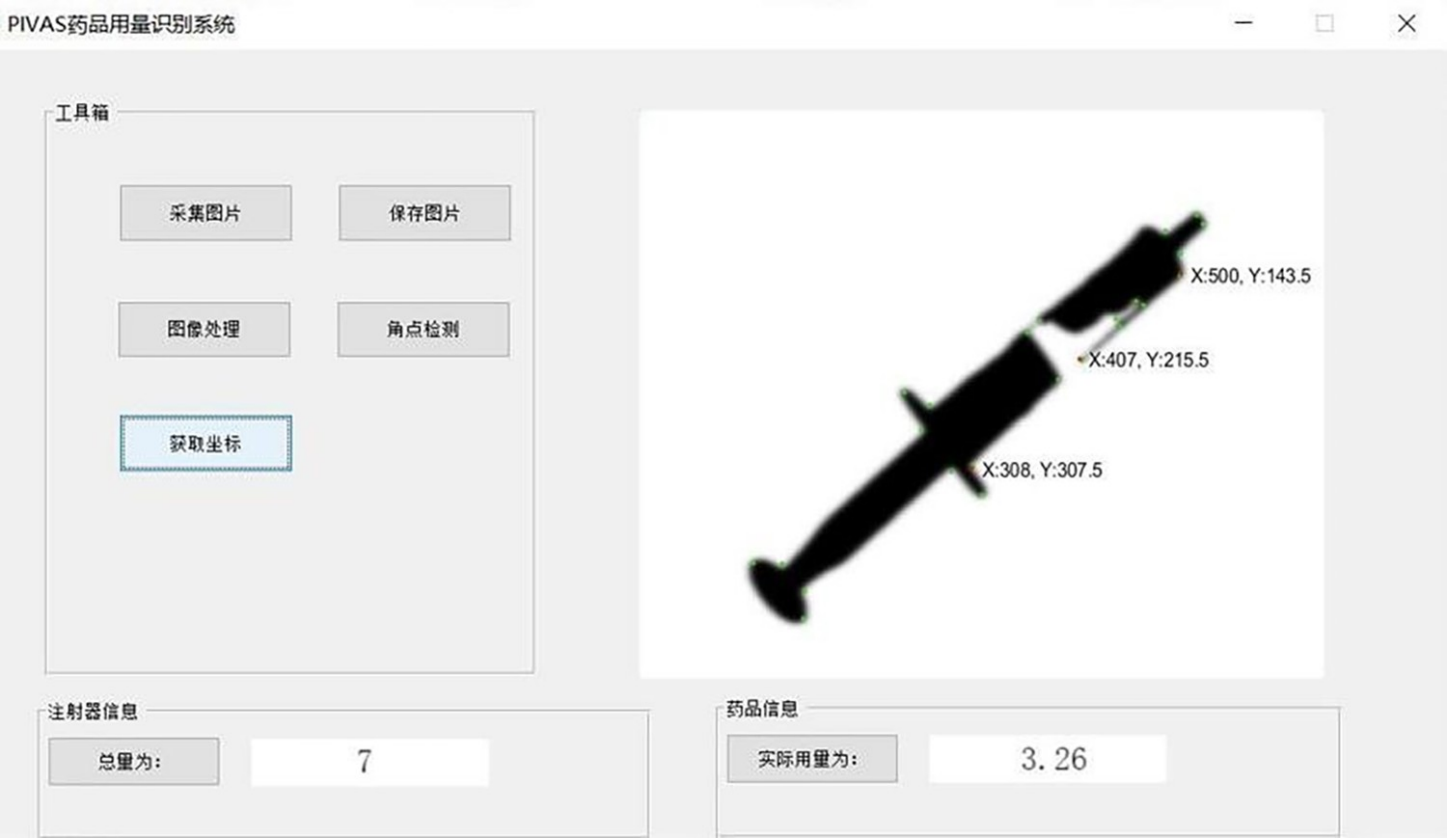
PIVAS drug corner detection interface.

Fig 12 shows the interface for corner detection of the syringe image. Firstly, the image of the syringe after drug extraction is captured by the camera, and then the positions of the piston in the syringe and the vertices of the image are obtained through the program. Finally, the system calculates the actual extraction amount of the drug, completes the task of drug dosage recognition, and provides the actual extraction amount of the drug in the result display area.

## Conclusion and outlook

### Summarize

PIVAS offers several advantages in terms of occupational protection and drug safety. However, intravenous infusions are associated with a higher incidence of adverse reactions than oral administration. Therefore, IV infusion safety is critical to treatment outcomes. The application of image recognition technology can improve drug safety and reduce drug errors. Before we introduced image recognition technology, the main workflow of PIVAS was manual. Although some intelligent methods have been introduced recently, they have little effect and waste a lot of time instead. The method proposed in this study may become an ideal choice for large hospitals with heavy workloads, huge time pressures, and strict quality control requirements.

This study centers on the whole workflow of PIVAS and integrates image recognition technology into the drug-checking and drug-dispensing process of PIVAS. Firstly, through experiment comparisons, a target detection model suitable for drug category recognition is selected in the drug-checking stage of PIVAS, and it is improved to enhance the recognition accuracy and speed of intravenous drug categories. Meanwhile, a drug dosage recognition model is studied in the drug-dispensing stage to further increase the accuracy of drug-dispensing.

The main work and results of the study are as follows:

(1) Collecting and constructing intravenous drug datasets. The study chooses to photograph the target drugs from different angles, different orientations, and different placement situations in the PIVAS scenario, collects a large amount of intravenous drug data, and labels the drugs with the annotation tool to form a trainable intravenous drug dataset.

(2) Analyze the nature of the intravenous drug dataset and perform data augmentation on it. The study adopts data augmentation methods such as mirroring, scaling, etc., which are commonly used nowadays, to expand the intravenous drug dataset, and to improve the model’s generalization ability and robustness. Meanwhile, the study uses random erasure processing and adding Gaussian noise for data augmentation to improve the detection performance of the model in complex scenes;

(3) Optimize the drug category recognition model of YOLOv5s. To improve the model’s recognition speed for drugs, we study and adopt the idea of the GhostNet module, replacing the Conv module in the YOLOv5s network with GhosConv, and combining it with the C3 module to generate the C3Ghost module. To address the issues of poor detection performance and missed/false detection of drugs, we study the fusion of the coordinate attention module at the C3 stage of the backbone network feature extraction to more accurately locate and recognize drug targets. Furthermore, we improve the feature fusion network in the Neck by using the Bi-FPN network to enhance the model’s feature fusion capability. Experimental verification is conducted using a constructed dataset of intravenous drugs, and ablation experiments are performed to compare the model with mainstream networks to validate its detection capability.

(4) To study the method of recognizing the drug dosage in the process of drug-dispensing. Based on the characteristics of the application scenario, we choose to recognize the position of the piston in the syringe to get the actual dosage of drugs. In terms of analyzing the nature of the image, to accurately extract the real and unique corners, the preprocessing strategies such as binarization, morphological processing, and Gaussian smoothing filtering are designed to effectively remove the noise and interference, and considering that the required corners are right-angled, the study adopts the Harris corner detection method to extract the coordinates of the corner, and based on the principle of scaling, the extracted coordinates of the corners are related to the computation, and the actual dosage of drugs is obtained. The analysis of the experimental results shows that the corner detection method adopted in the study is suitable for recognizing the dosage of drugs in different scenarios;

(5) Design and implement the PIVAS drug category recognition system and PIVAS drug dosage recognition system, divide and encapsulate the functions of each module of the system, describe the system functions and outputs in detail, and encapsulate the model into the system, and design the visualization interface to facilitate the user’s operation.

### Outlook

At present, how to reduce labor costs and complete the whole process of PIVAS accurately and quickly is still the main problem facing large-scale PIVAS applications. This study provides an intelligent solution to improve the accuracy and safety of drug dispensing in PIVAS, which is a good reference for promoting the communication of PIVAS pharmacists in hospitals at home and abroad as well as the future development of PIVAS.

However, there are still some problems, and continued research can be focused on the following areas in the future:

(1) Expansion of dataset diversity. Nowadays, most of the image recognition networks applied to drug category recognition use fully supervised learning, although the dataset size can be expanded by data augmentation operations, it is still difficult to meet the requirements of sample data. In the future, we can consider using semi-supervised and weakly supervised learning to reduce the requirement of sample data, or using adversarial generative networks (GAN) to realize the expansion of the dataset.

(2) Although the drug recognition algorithm can recognize drug categories in PIVAS scenarios, it cannot cover all drugs in the pharmacy due to the limited drug categories. Considering that it may be necessary to recognize drugs in other scenarios in the future, future research will explore a method that can effectively detect all drugs in the pharmacy based on the existing target detection algorithms for drugs and combine them with text recognition, label recognition, and other techniques.

(3) Although the study can realize the task of recognizing the dosage of injectable drugs based on the corners, it is limited to the collected pictures, and the real-time recognition of the dosage of drugs is susceptible to external interference, which leads to the discrepancy between the final dosage and the actual dosage. Subsequent research will address this aspect and explore a method that can detect drug dosage in real-time.

(4) Optimize this system and maximize the integration of this system with other intelligent devices. For example, the introduction of automatic sorters and logistics robots, thereby increasing efficiency, reducing the risk of error, and enhancing occupational protection.

## Supporting information

S1 Dataset (ZIP)

S1 File (ZIP)
