# Supplementary figures and images for: Intelligent supervision of PIVAS drug dispensing based on image recognition technology (part 2 of 2)
Source: PLoS One. 2024 Apr 4;19(4):e0298109. doi: 10.1371/journal.pone.0298109 (PMC10994394; doi:10.1371/journal.pone.0298109)

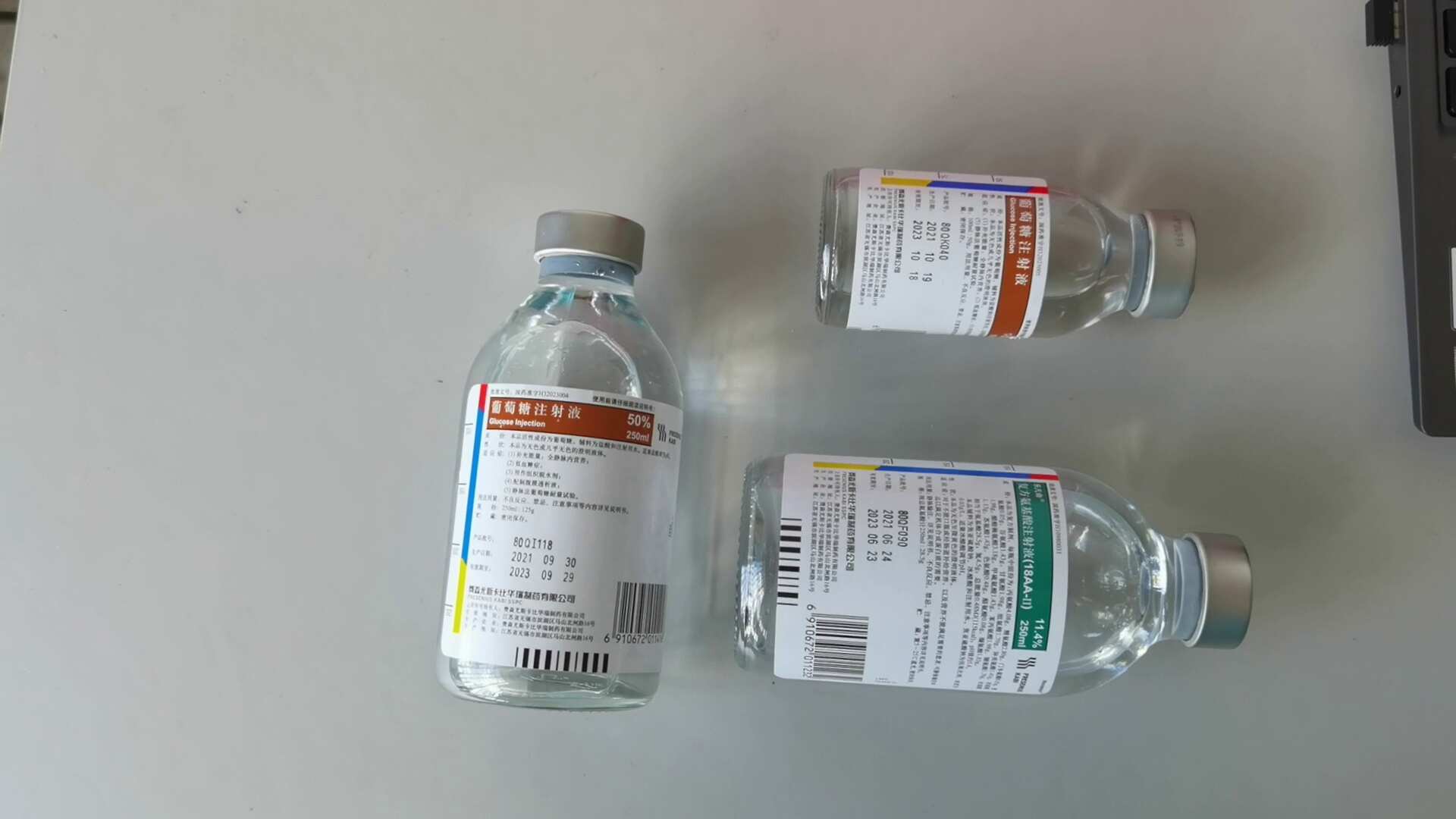

Supplement: S1 Dataset — (ZIP) [file pone.0298109.s001.zip › minimal data set/VOC2007/images/1089.jpg]

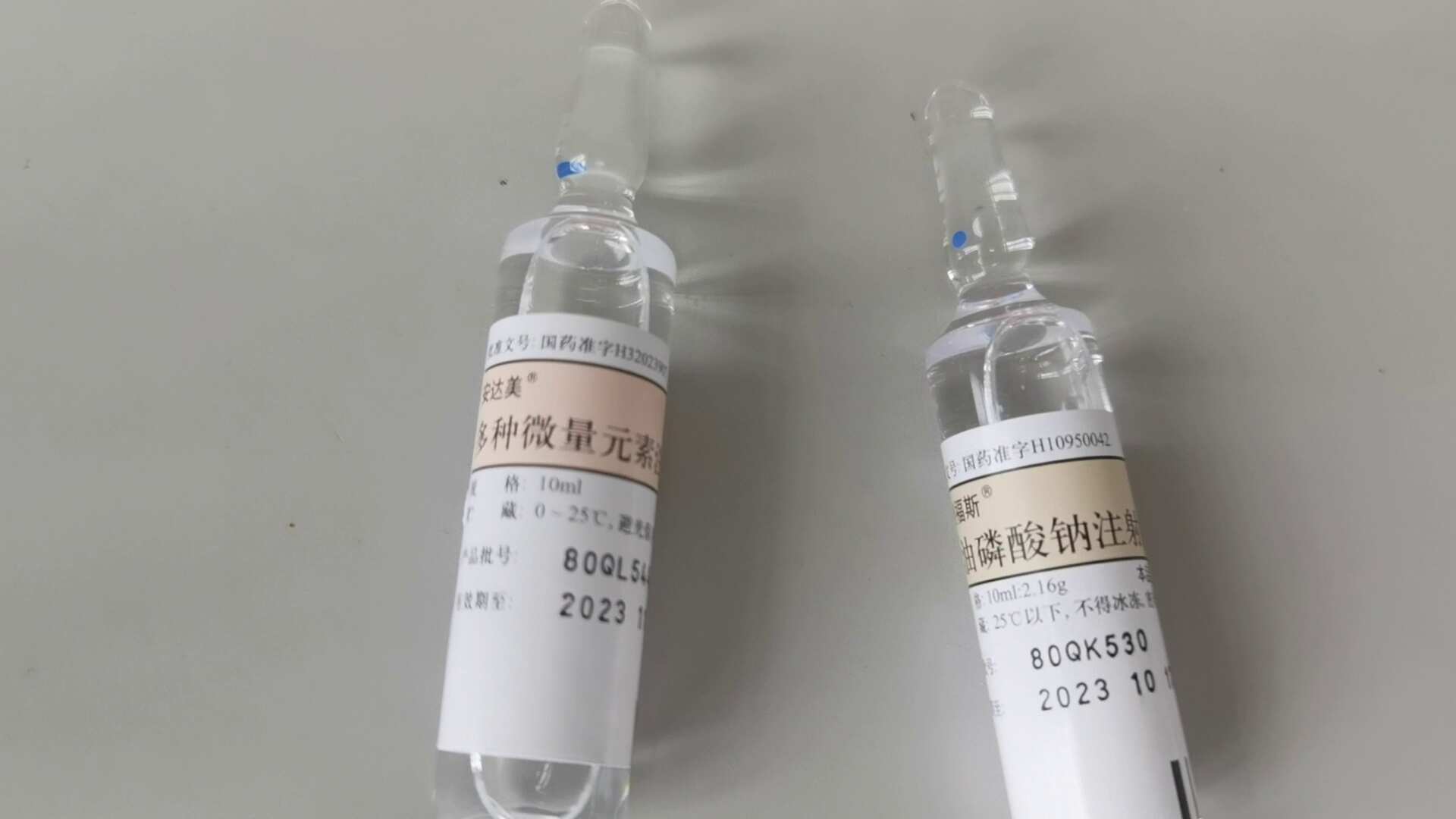

Supplement: S1 Dataset — (ZIP) [file pone.0298109.s001.zip › minimal data set/VOC2007/images/109.jpg]

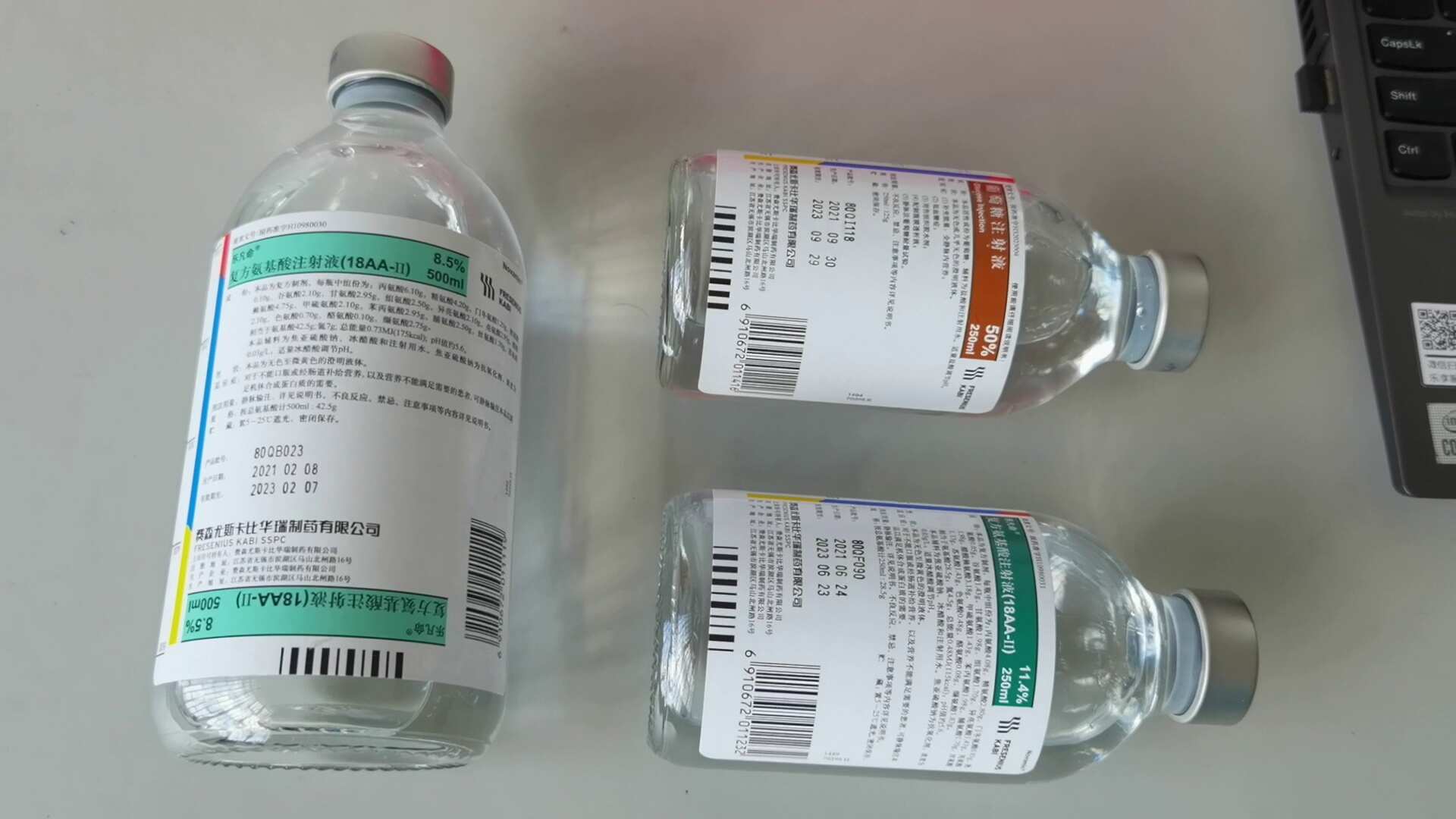

Supplement: S1 Dataset — (ZIP) [file pone.0298109.s001.zip › minimal data set/VOC2007/images/1090.jpg]

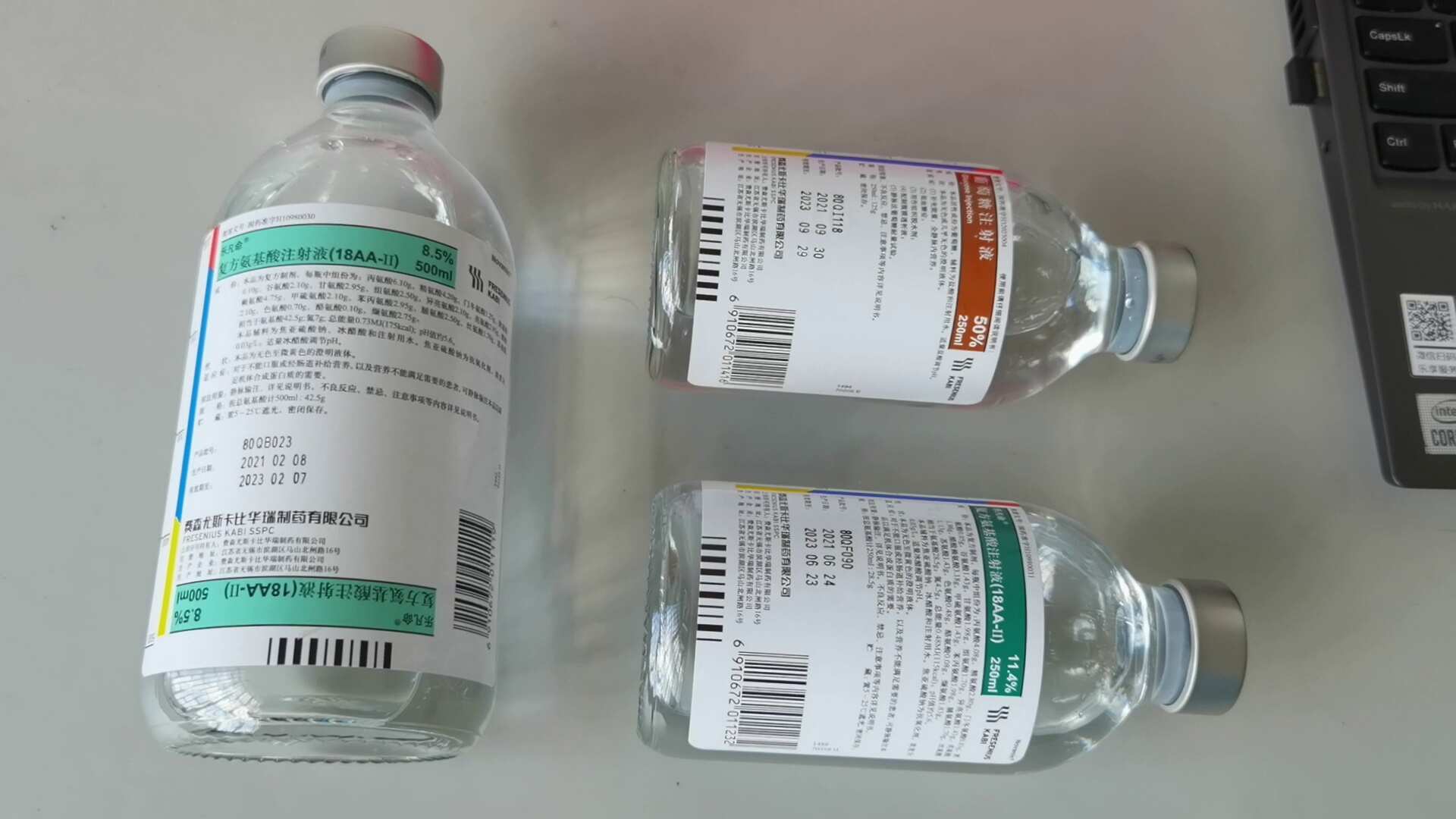

Supplement: S1 Dataset — (ZIP) [file pone.0298109.s001.zip › minimal data set/VOC2007/images/1091.jpg]

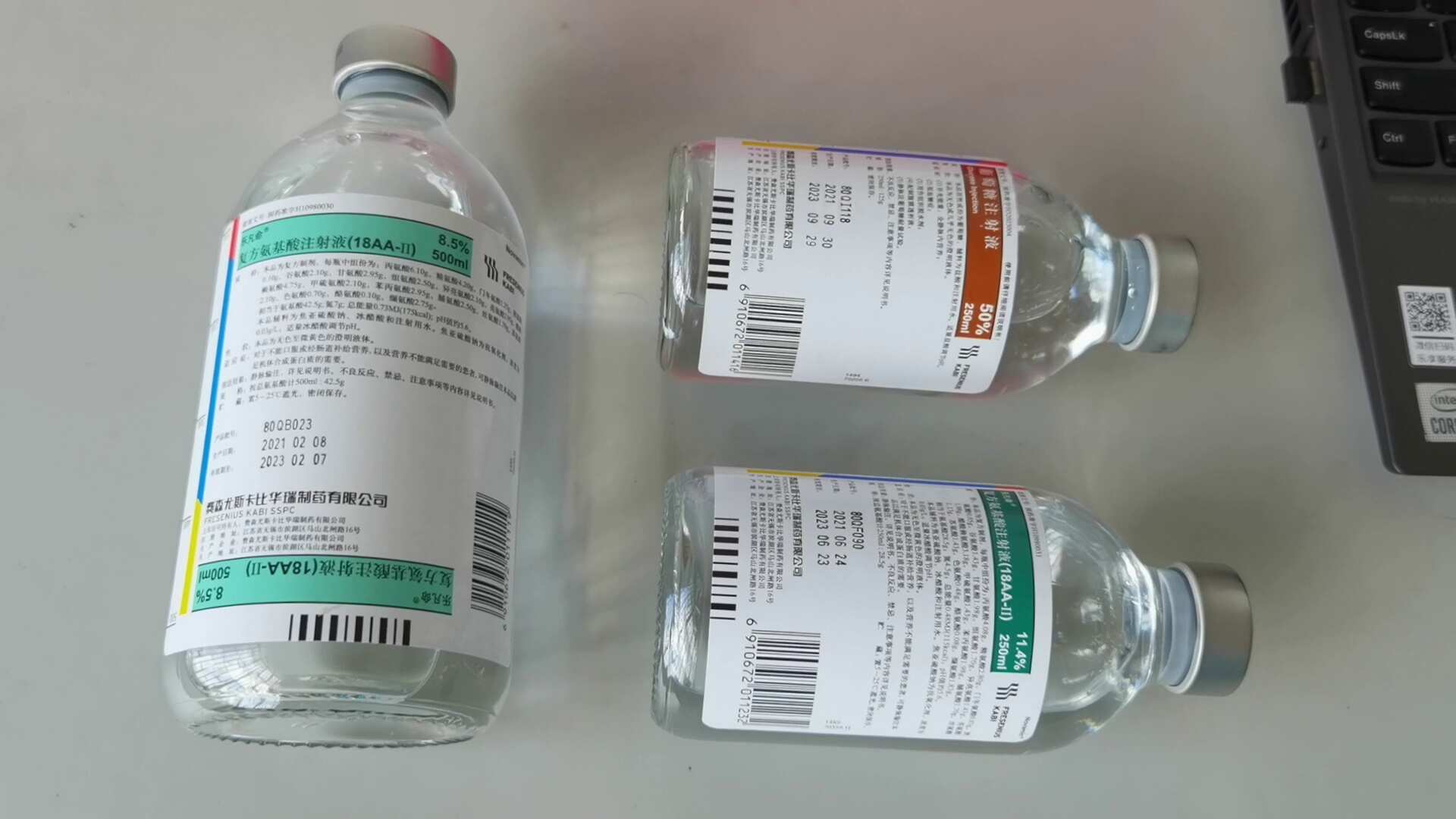

Supplement: S1 Dataset — (ZIP) [file pone.0298109.s001.zip › minimal data set/VOC2007/images/1092.jpg]

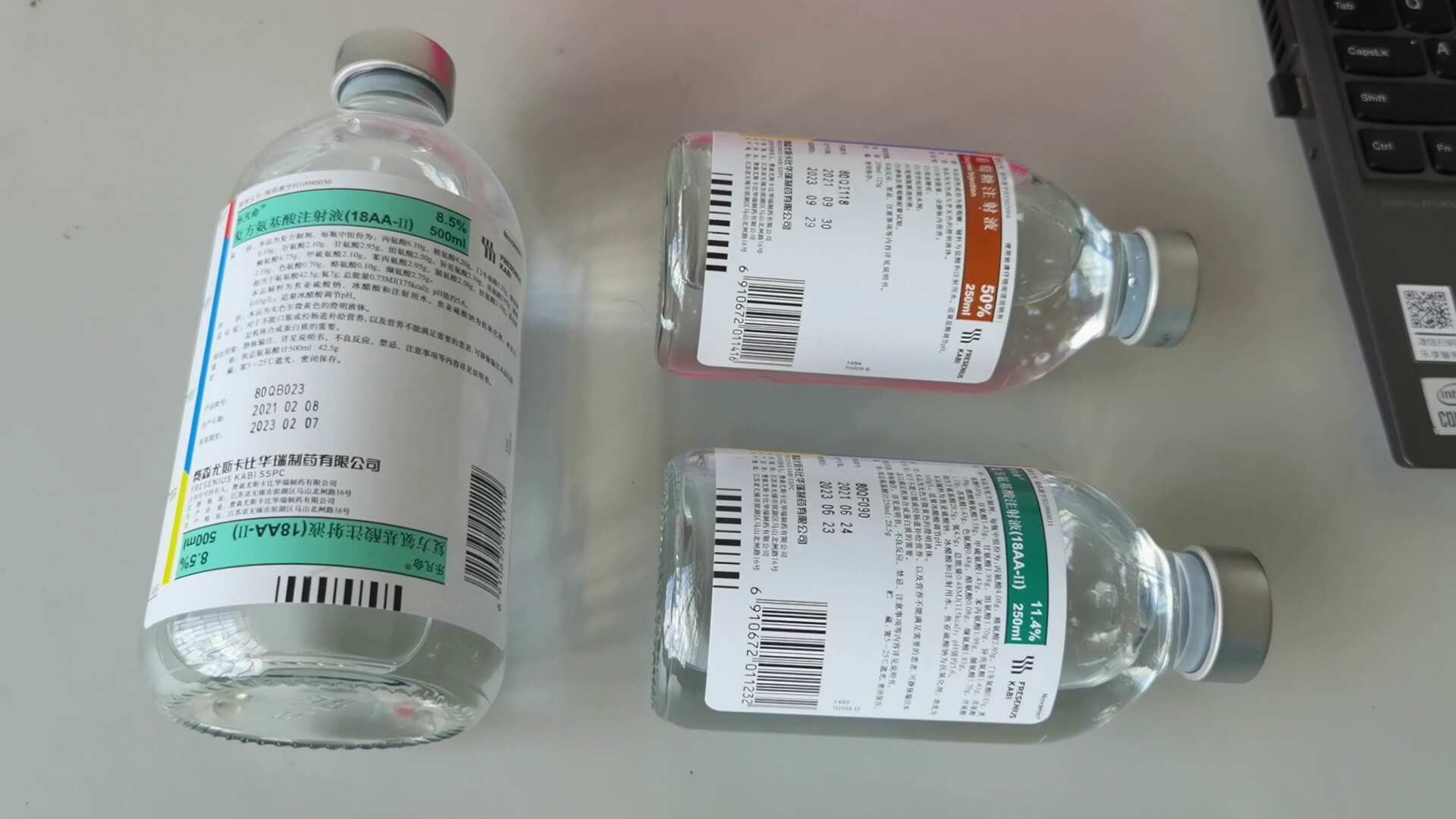

Supplement: S1 Dataset — (ZIP) [file pone.0298109.s001.zip › minimal data set/VOC2007/images/1093.jpg]

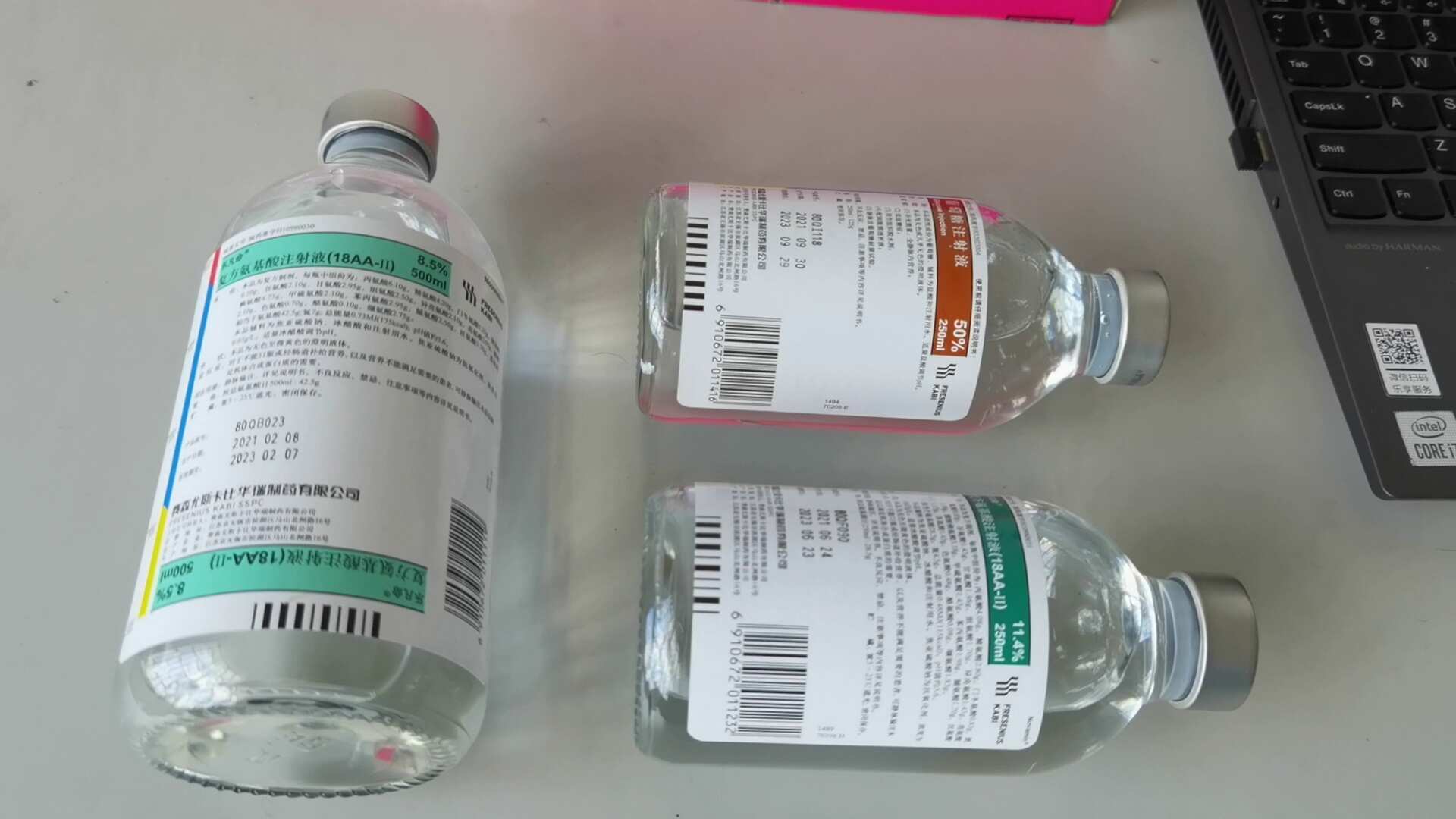

Supplement: S1 Dataset — (ZIP) [file pone.0298109.s001.zip › minimal data set/VOC2007/images/1094.jpg]

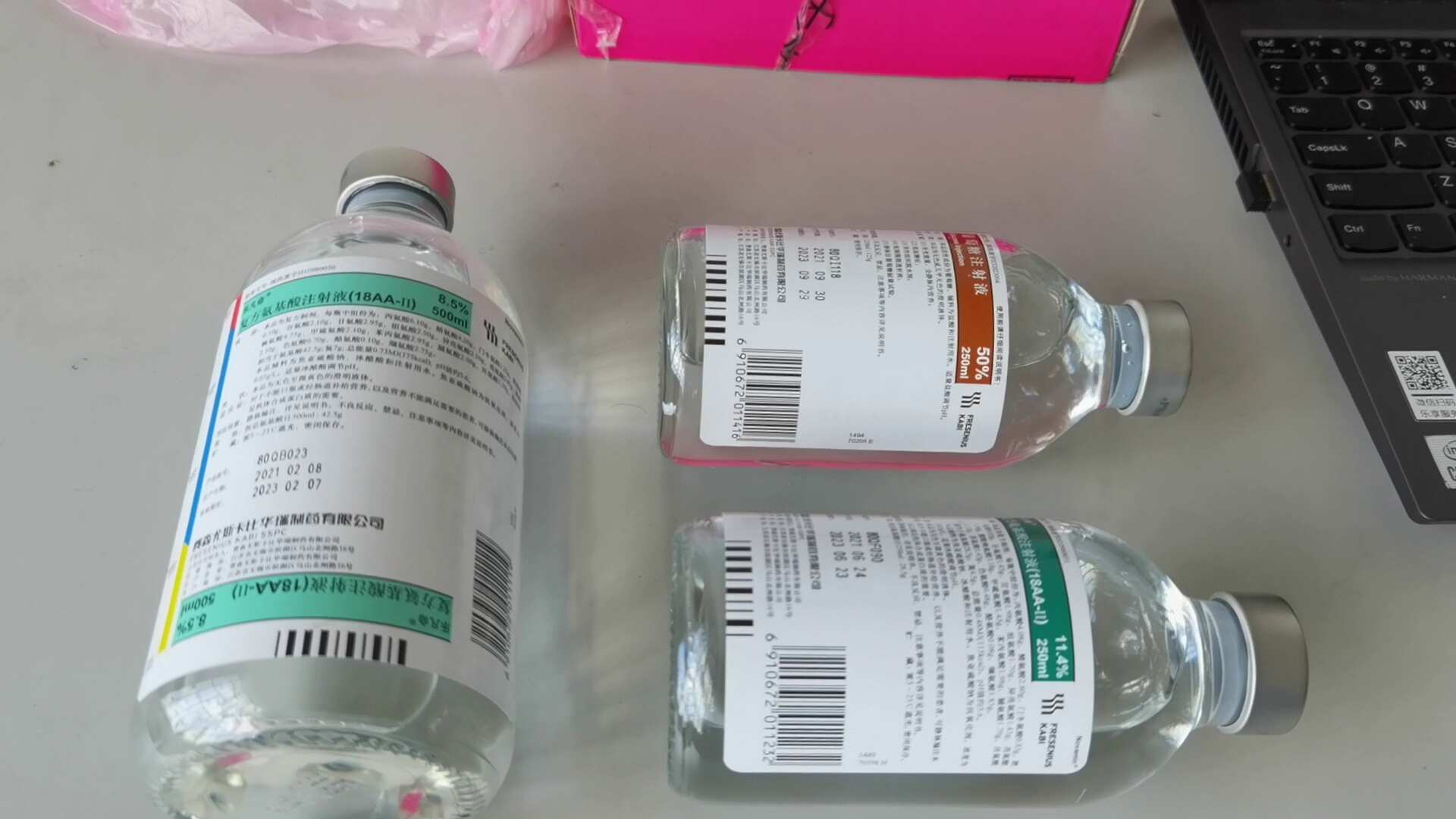

Supplement: S1 Dataset — (ZIP) [file pone.0298109.s001.zip › minimal data set/VOC2007/images/1095.jpg]

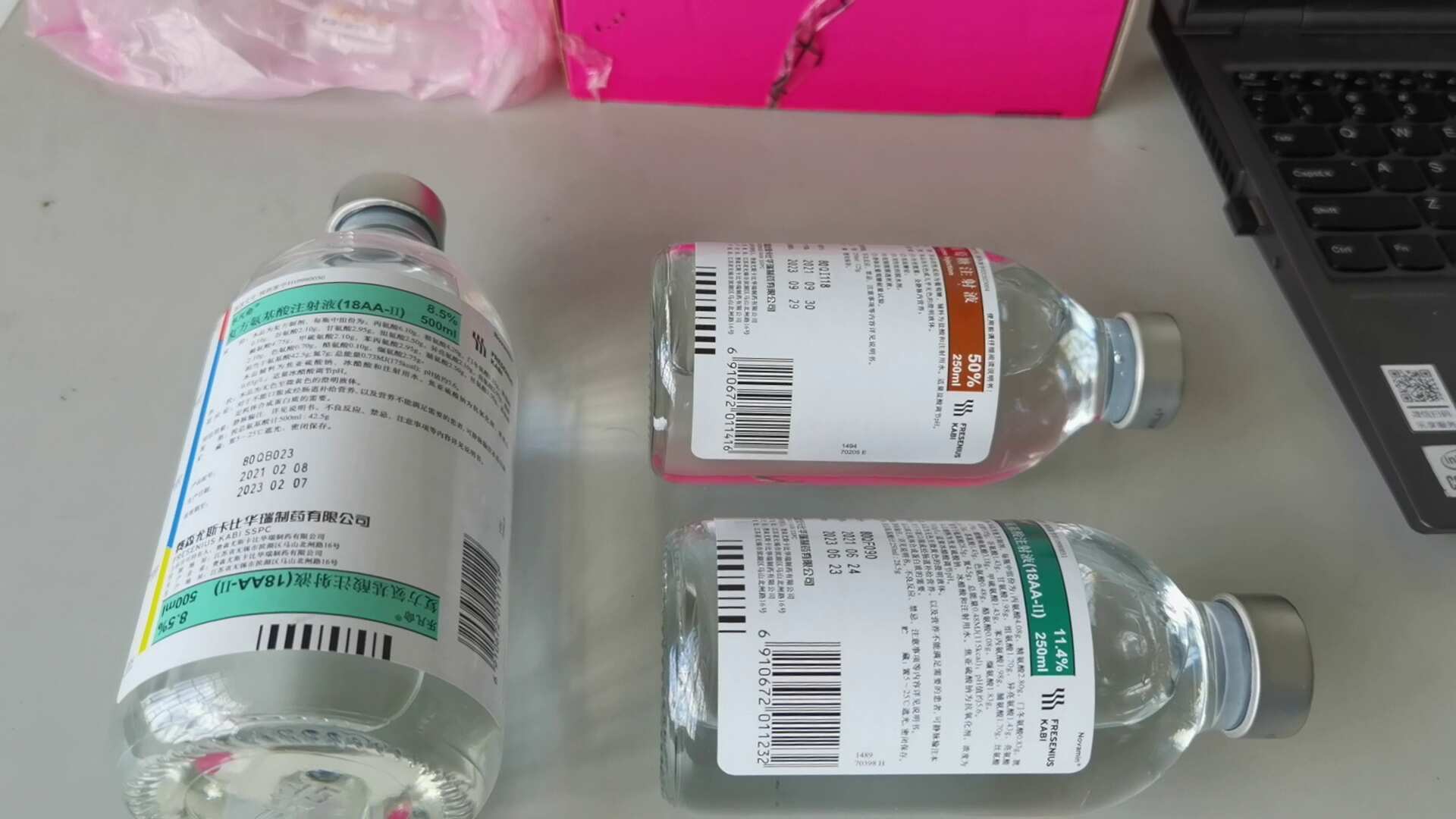

Supplement: S1 Dataset — (ZIP) [file pone.0298109.s001.zip › minimal data set/VOC2007/images/1096.jpg]

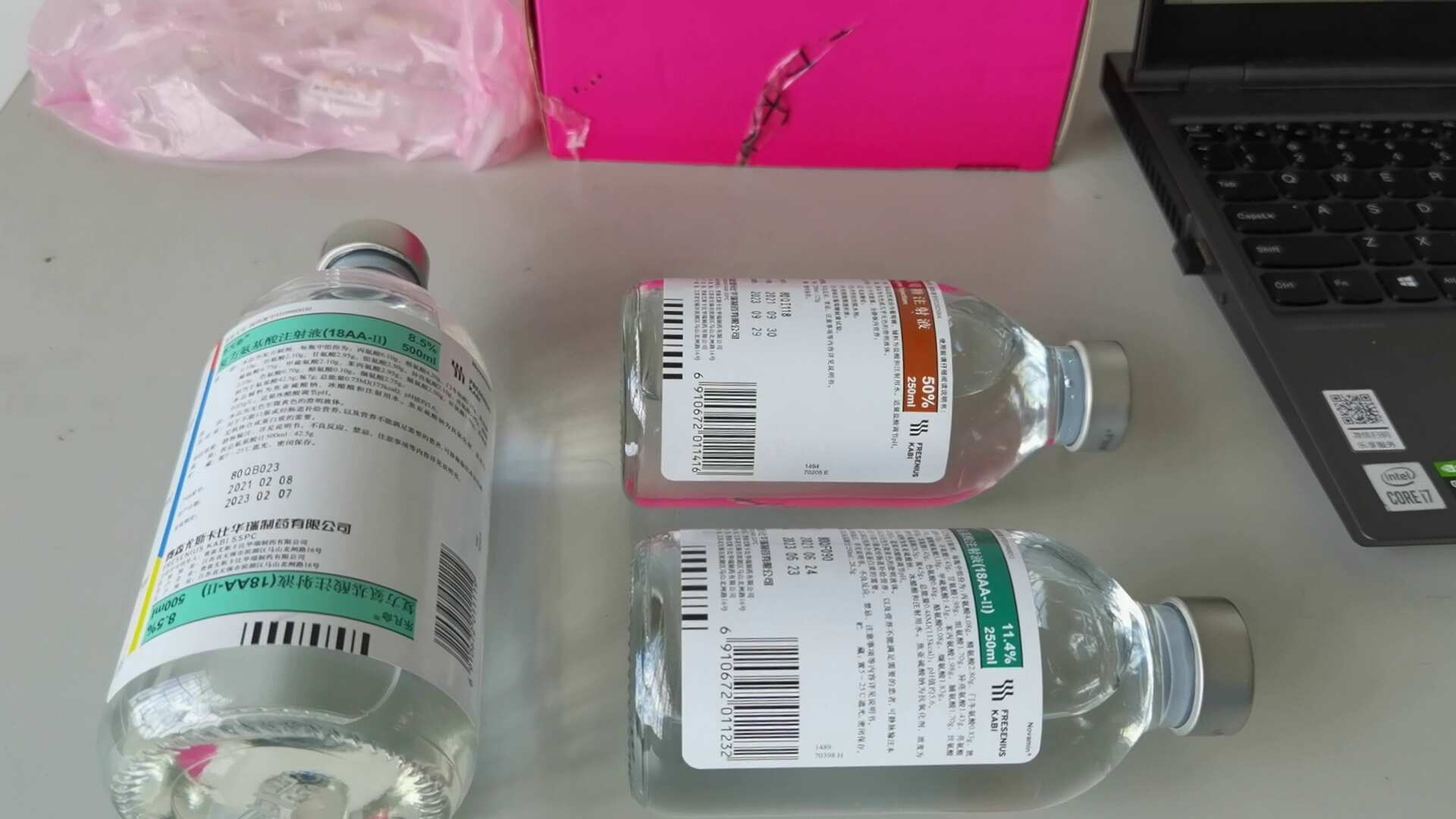

Supplement: S1 Dataset — (ZIP) [file pone.0298109.s001.zip › minimal data set/VOC2007/images/1097.jpg]

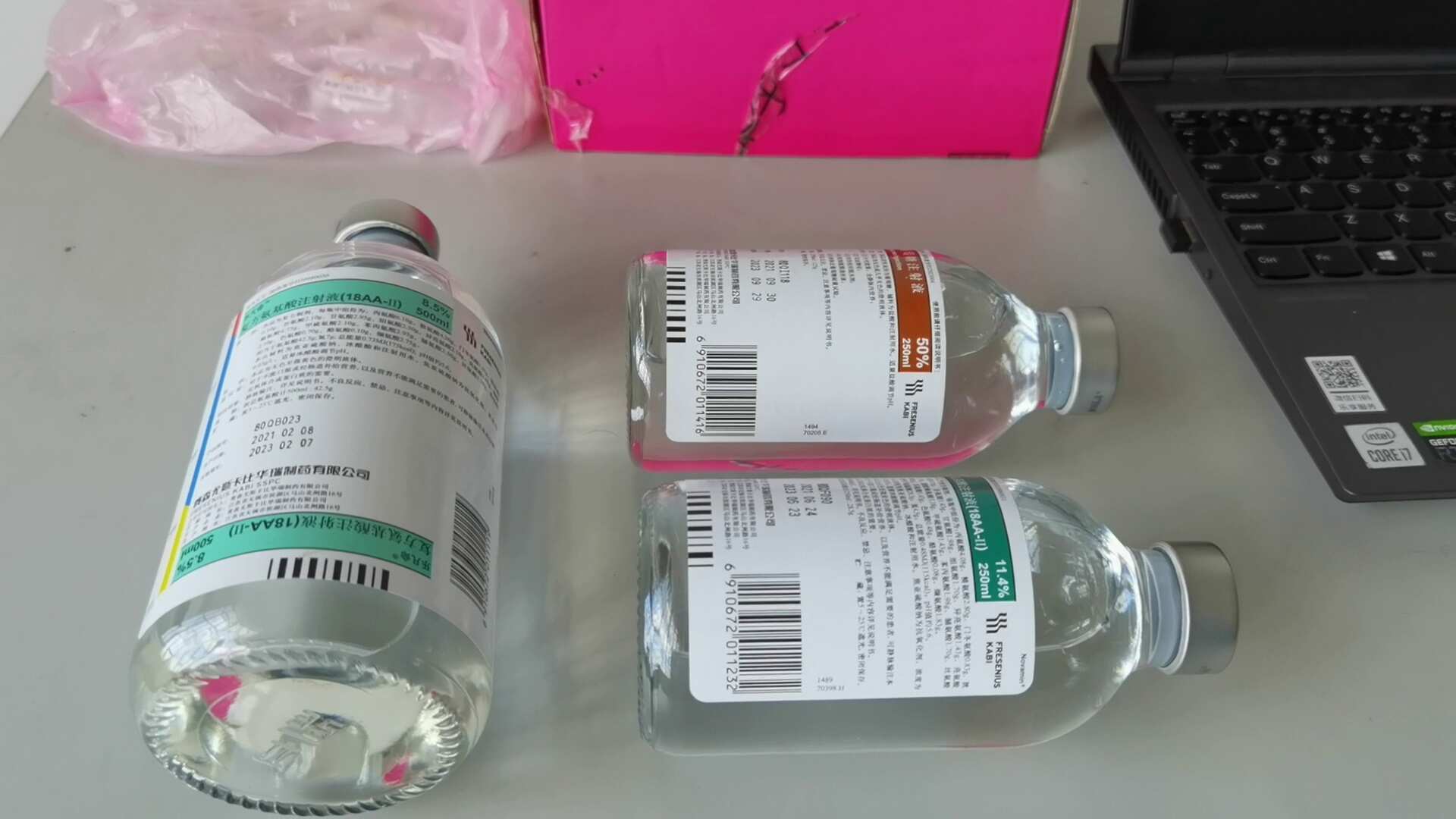

Supplement: S1 Dataset — (ZIP) [file pone.0298109.s001.zip › minimal data set/VOC2007/images/1098.jpg]

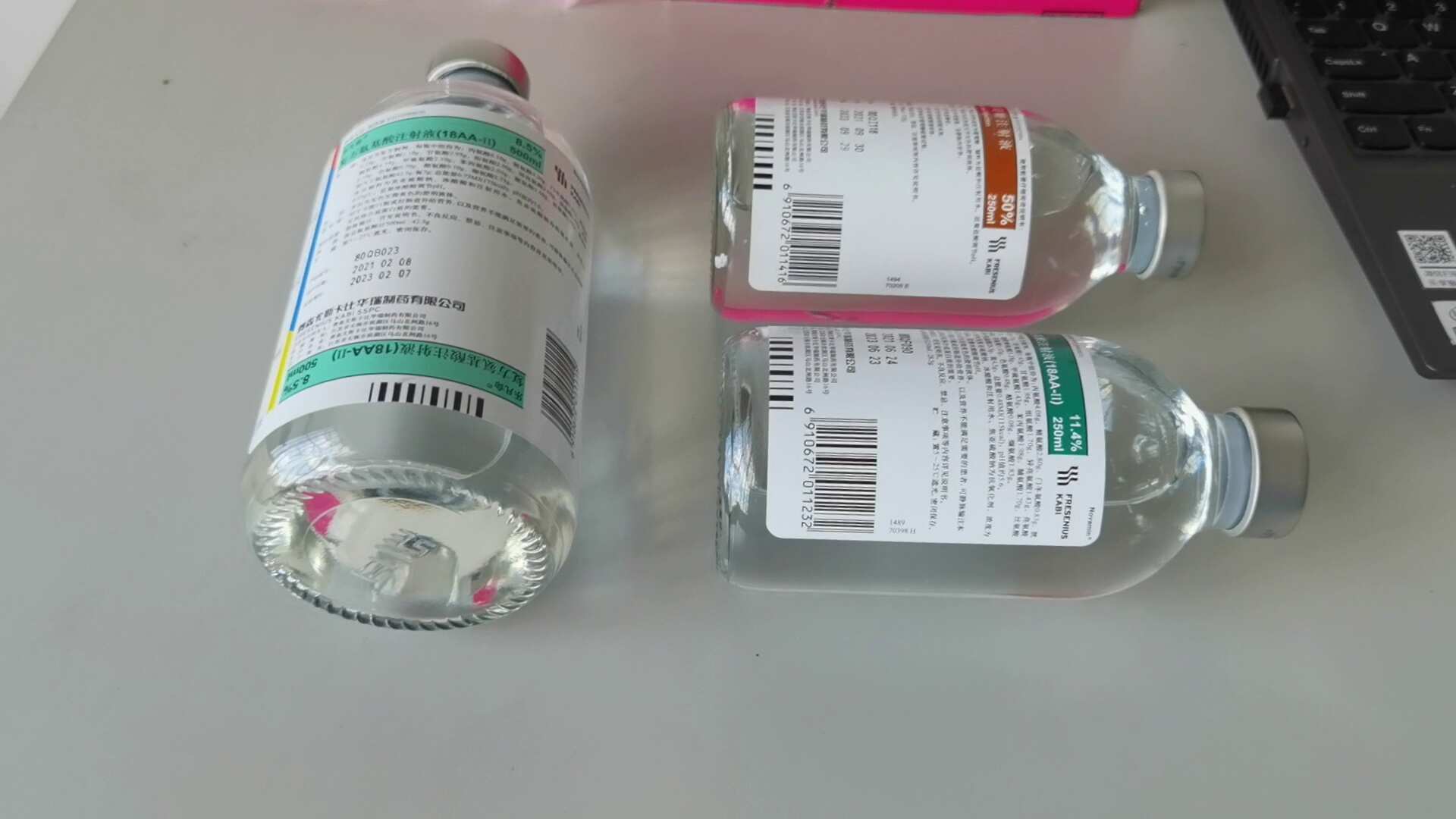

Supplement: S1 Dataset — (ZIP) [file pone.0298109.s001.zip › minimal data set/VOC2007/images/1099.jpg]

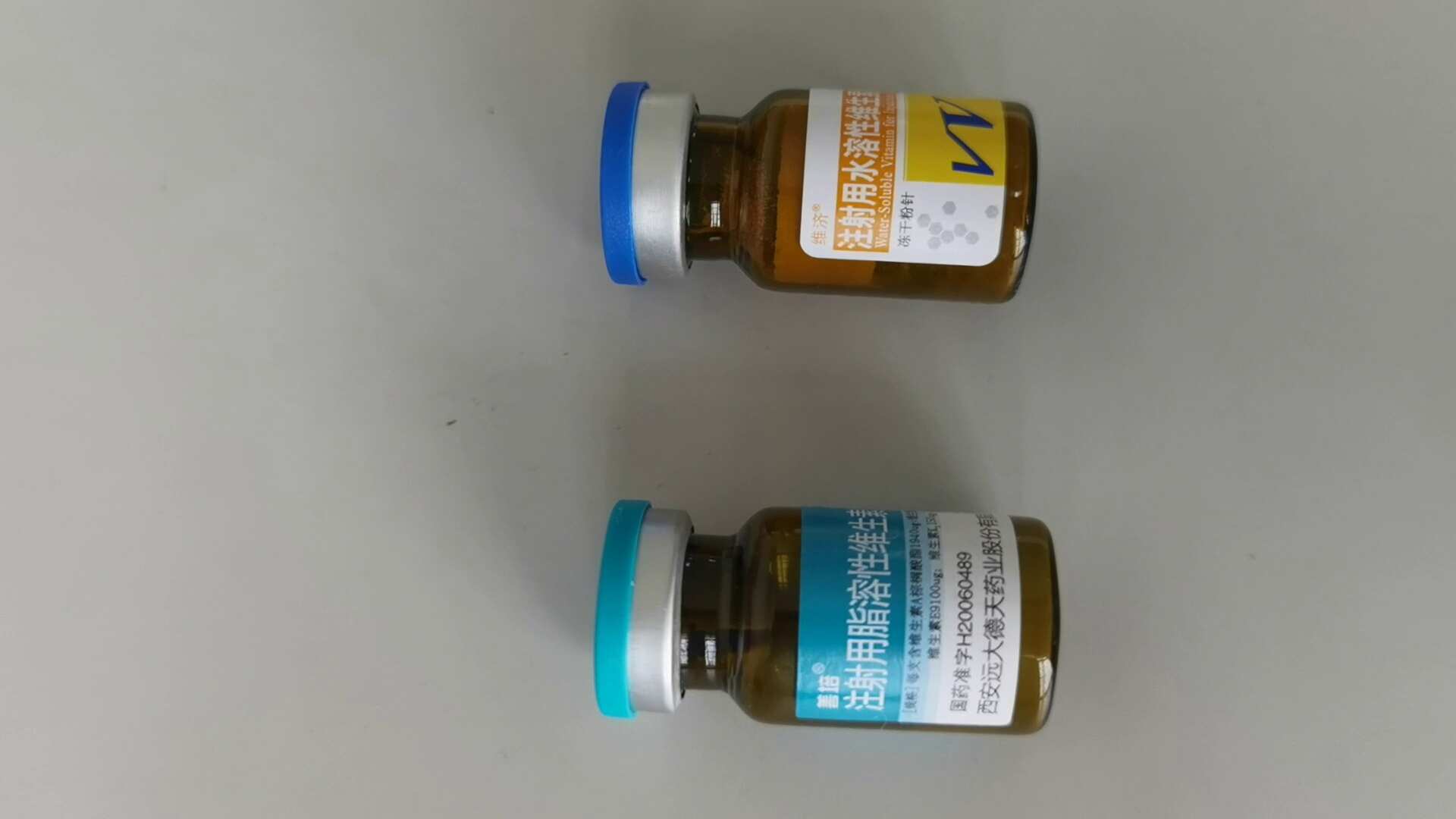

Supplement: S1 Dataset — (ZIP) [file pone.0298109.s001.zip › minimal data set/VOC2007/images/11.jpg]

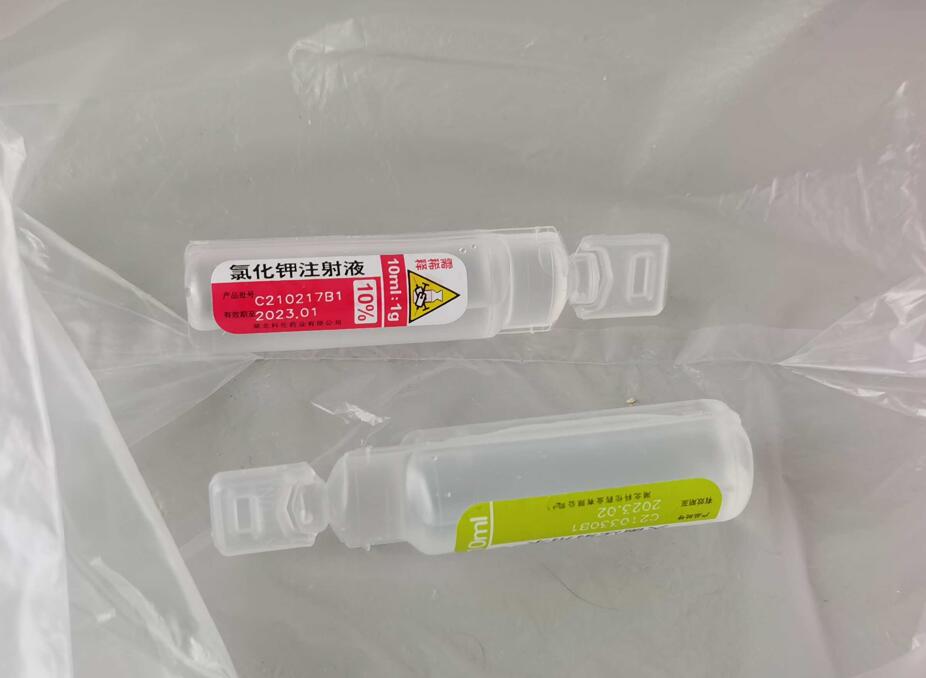

Supplement: S1 Dataset — (ZIP) [file pone.0298109.s001.zip › minimal data set/VOC2007/images/110.jpg]

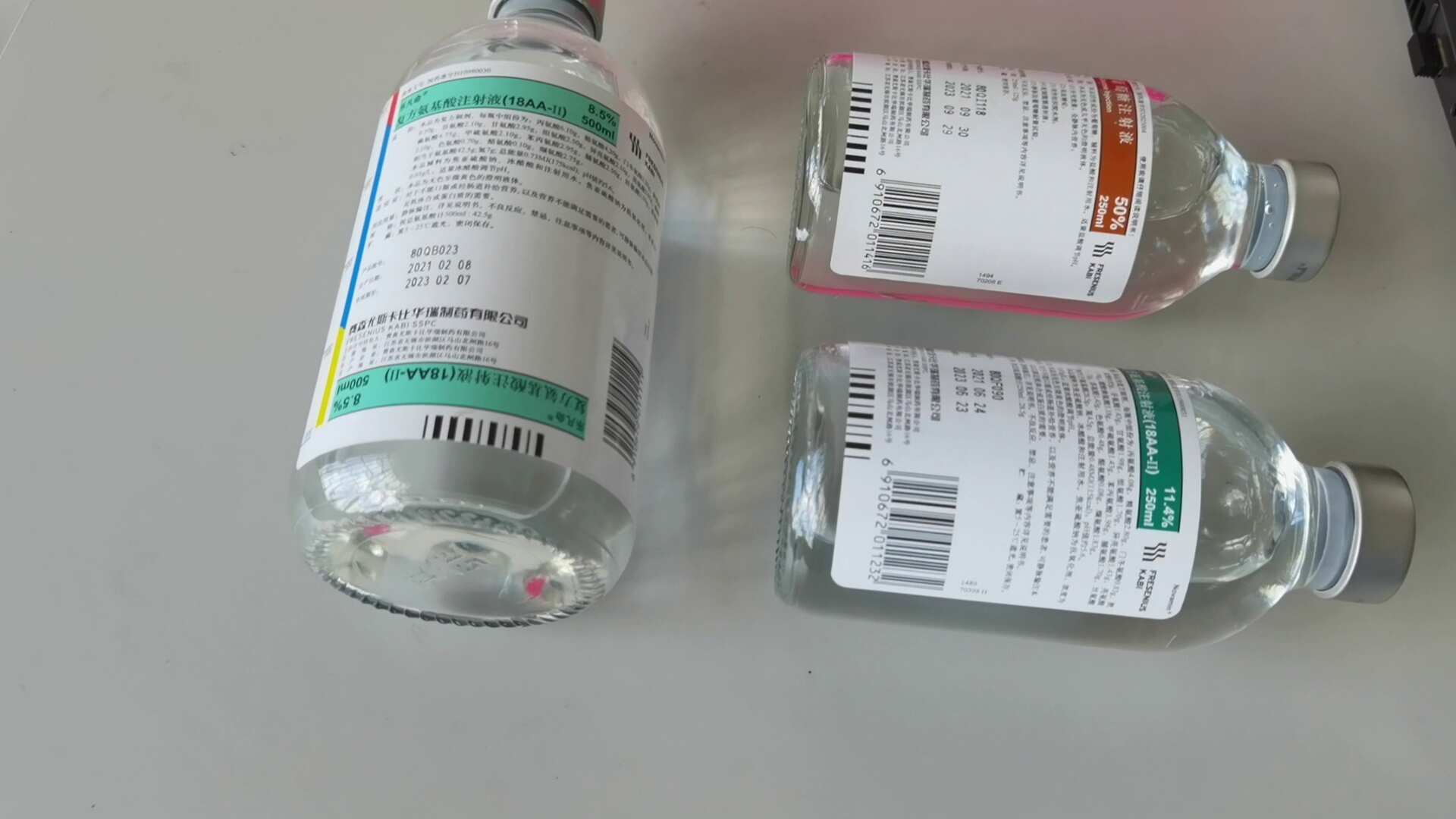

Supplement: S1 Dataset — (ZIP) [file pone.0298109.s001.zip › minimal data set/VOC2007/images/1100.jpg]

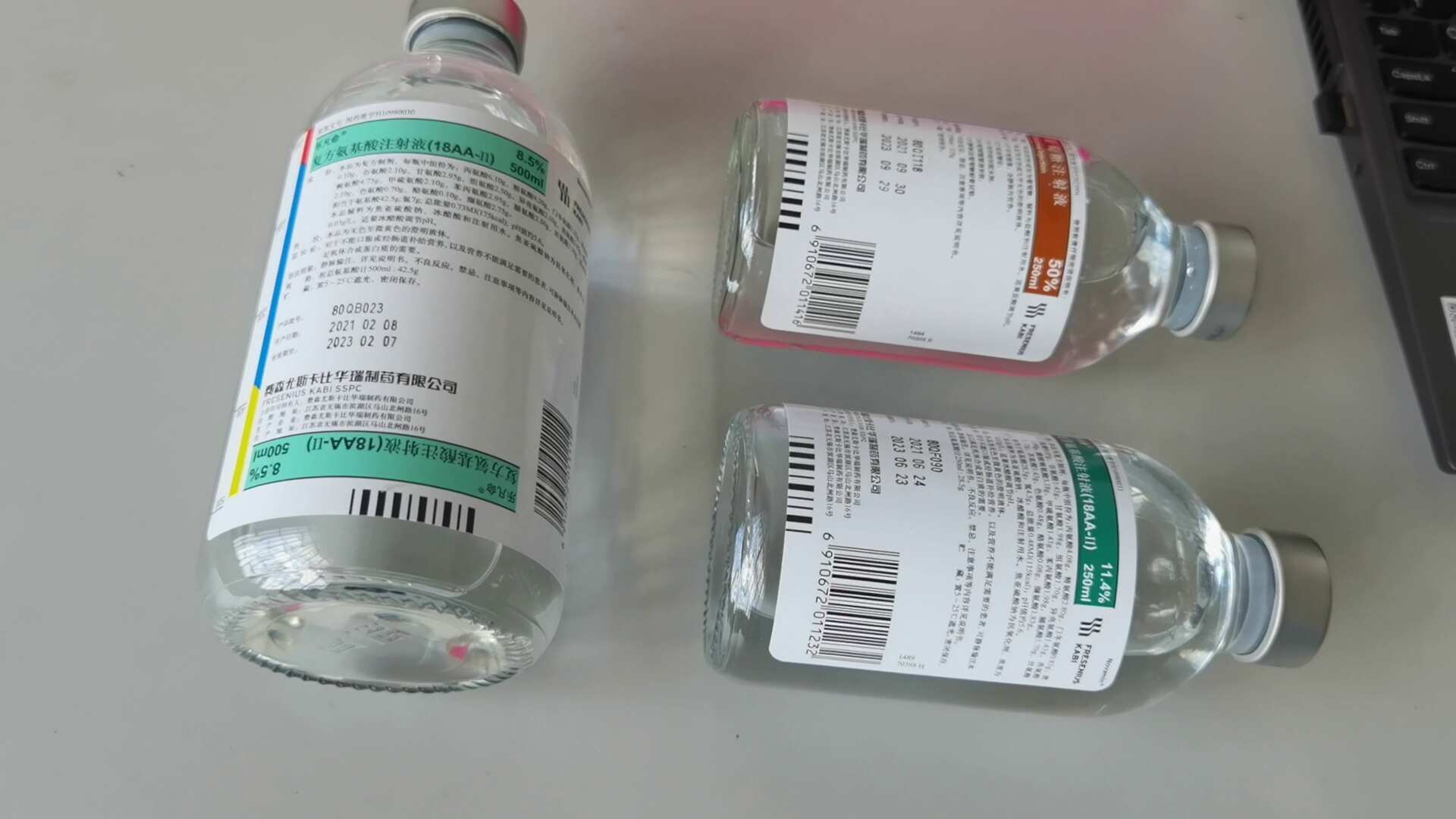

Supplement: S1 Dataset — (ZIP) [file pone.0298109.s001.zip › minimal data set/VOC2007/images/1101.jpg]

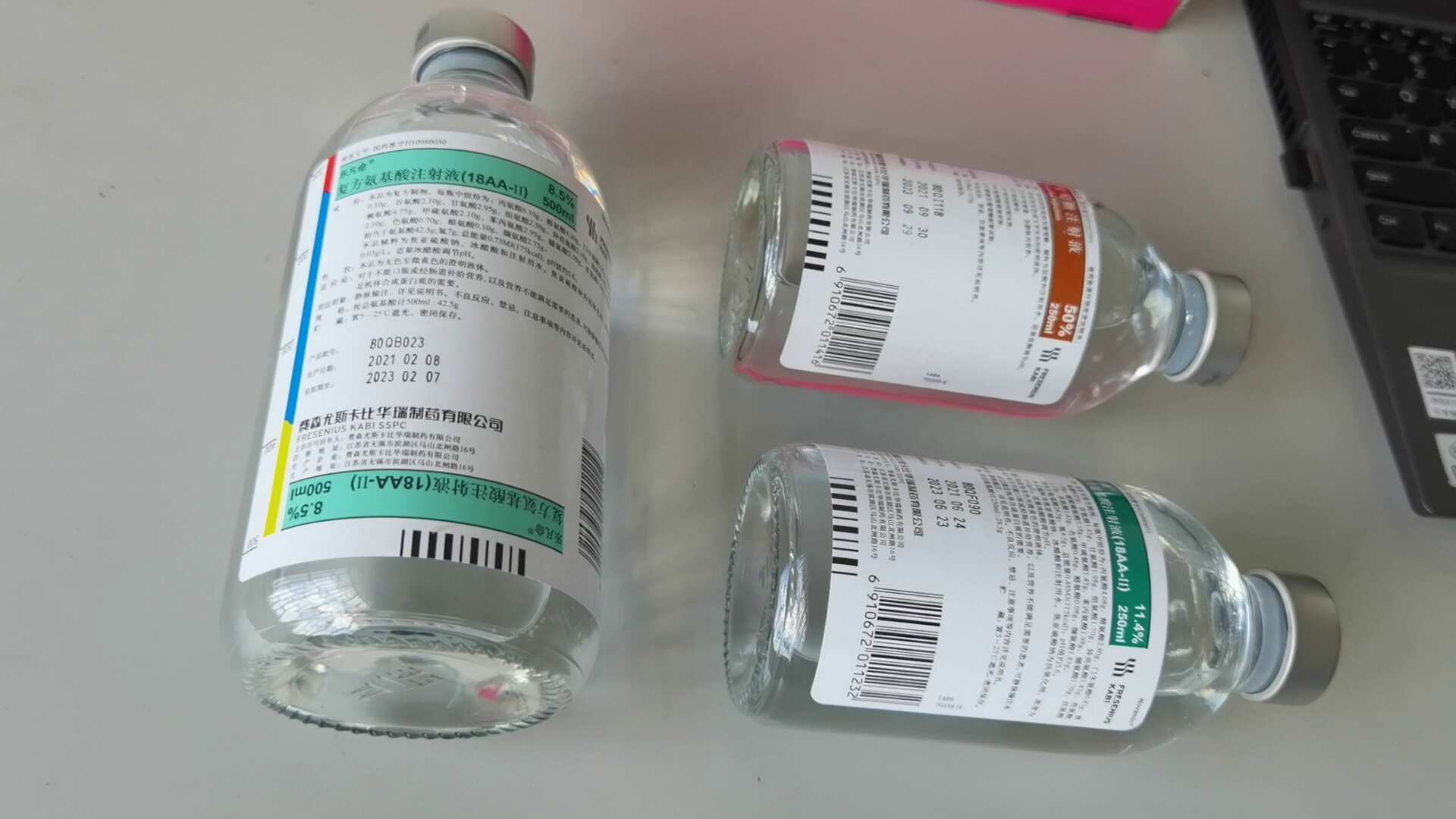

Supplement: S1 Dataset — (ZIP) [file pone.0298109.s001.zip › minimal data set/VOC2007/images/1102.jpg]

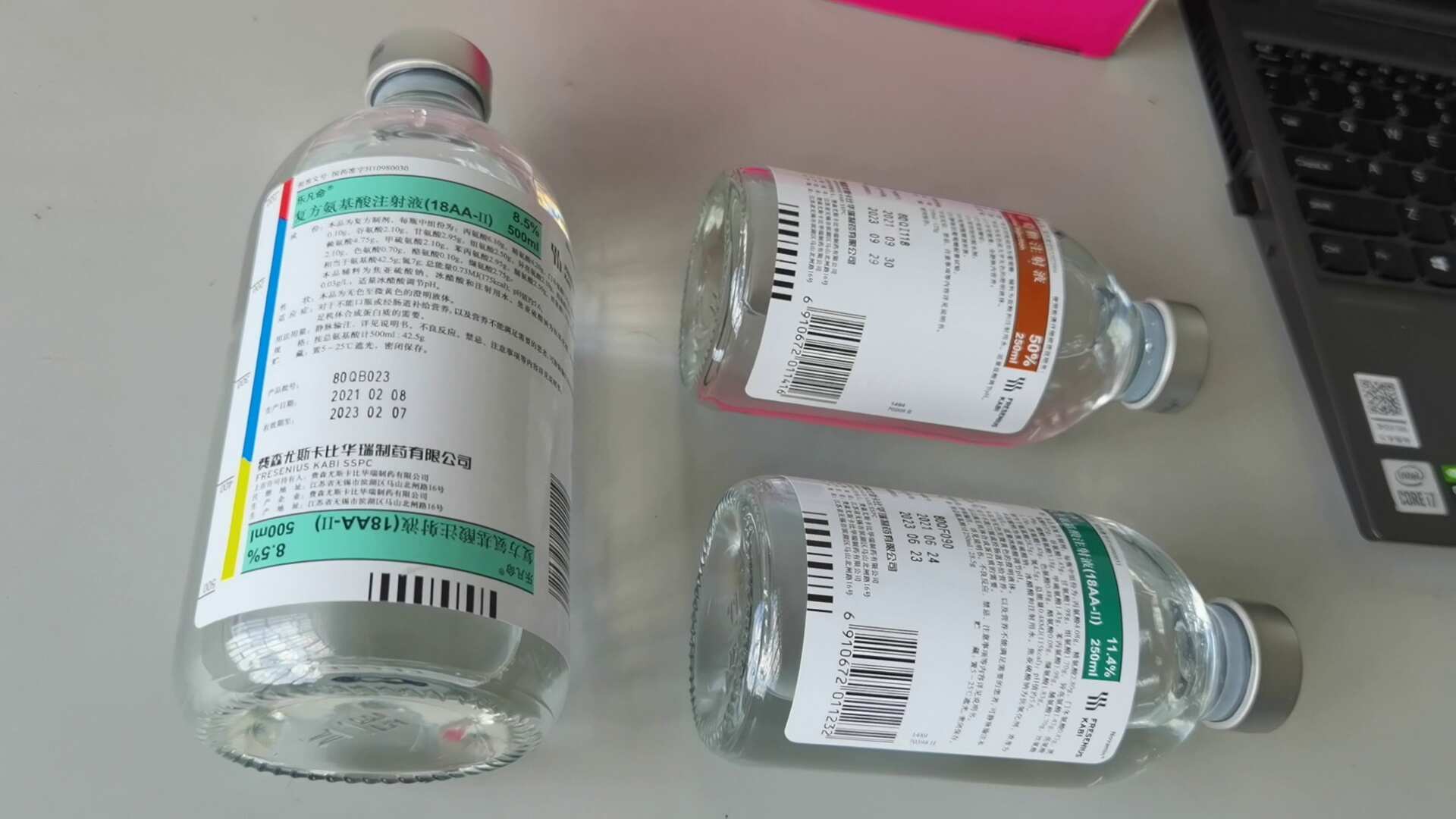

Supplement: S1 Dataset — (ZIP) [file pone.0298109.s001.zip › minimal data set/VOC2007/images/1103.jpg]

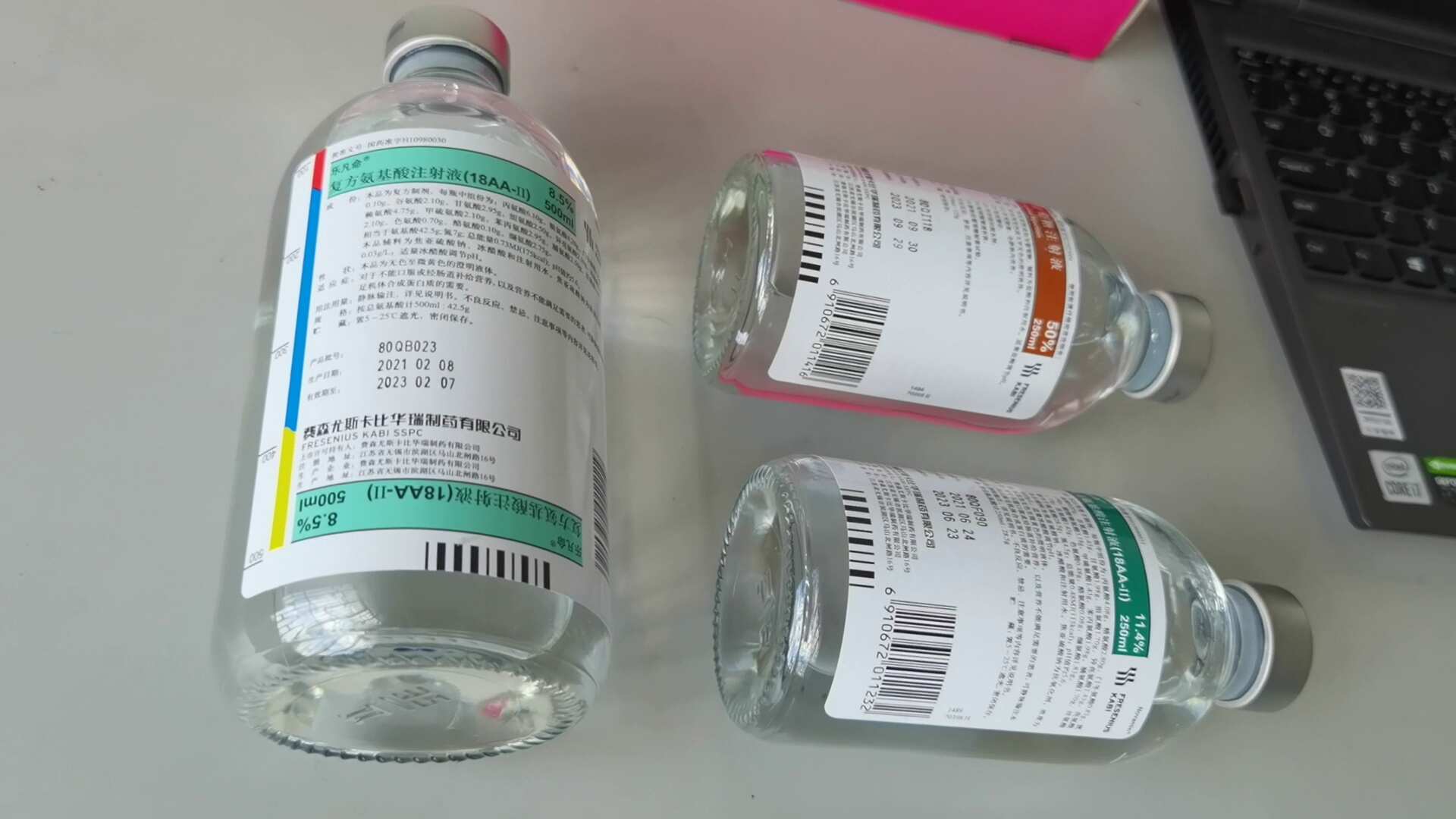

Supplement: S1 Dataset — (ZIP) [file pone.0298109.s001.zip › minimal data set/VOC2007/images/1104.jpg]

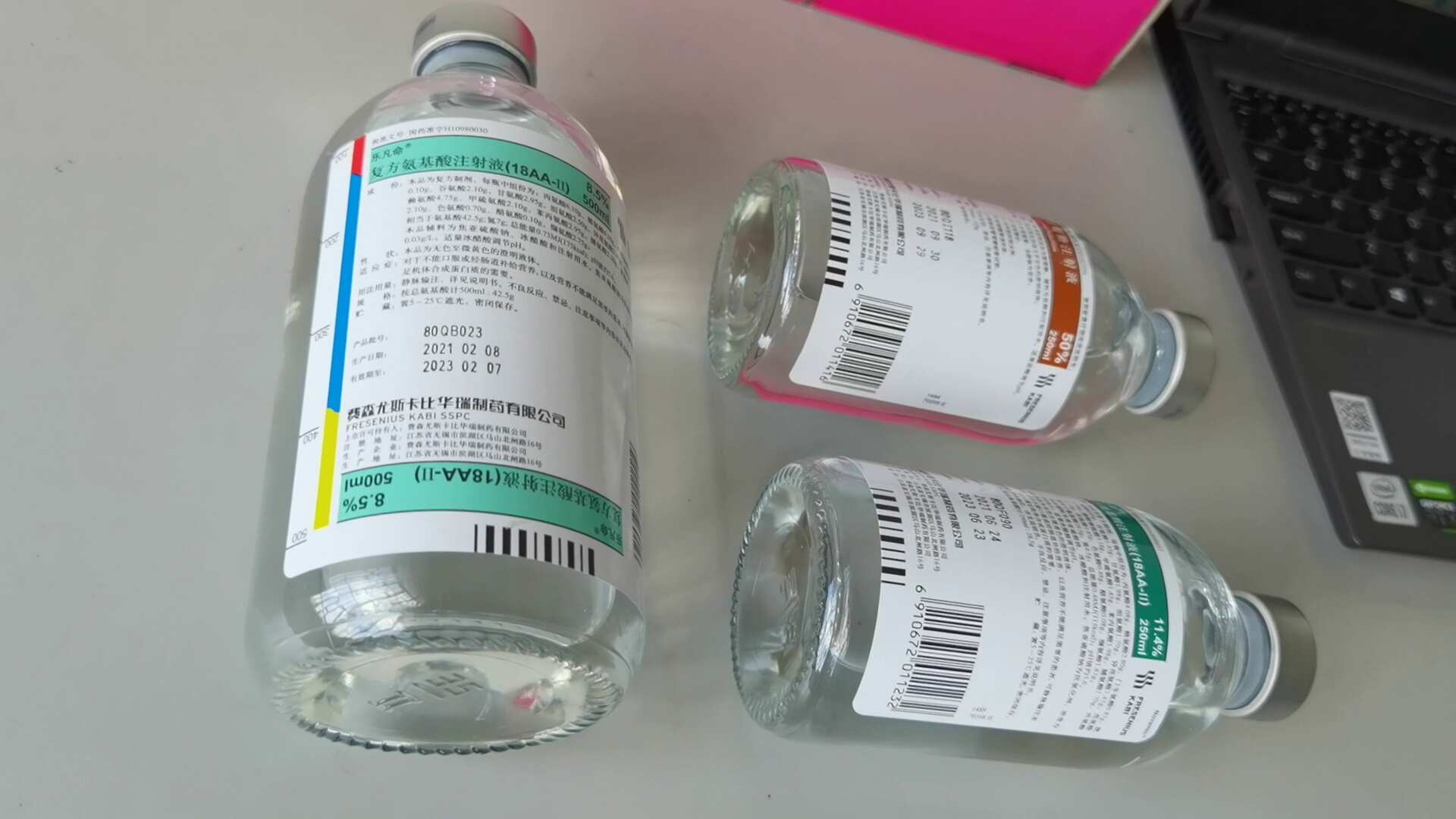

Supplement: S1 Dataset — (ZIP) [file pone.0298109.s001.zip › minimal data set/VOC2007/images/1105.jpg]

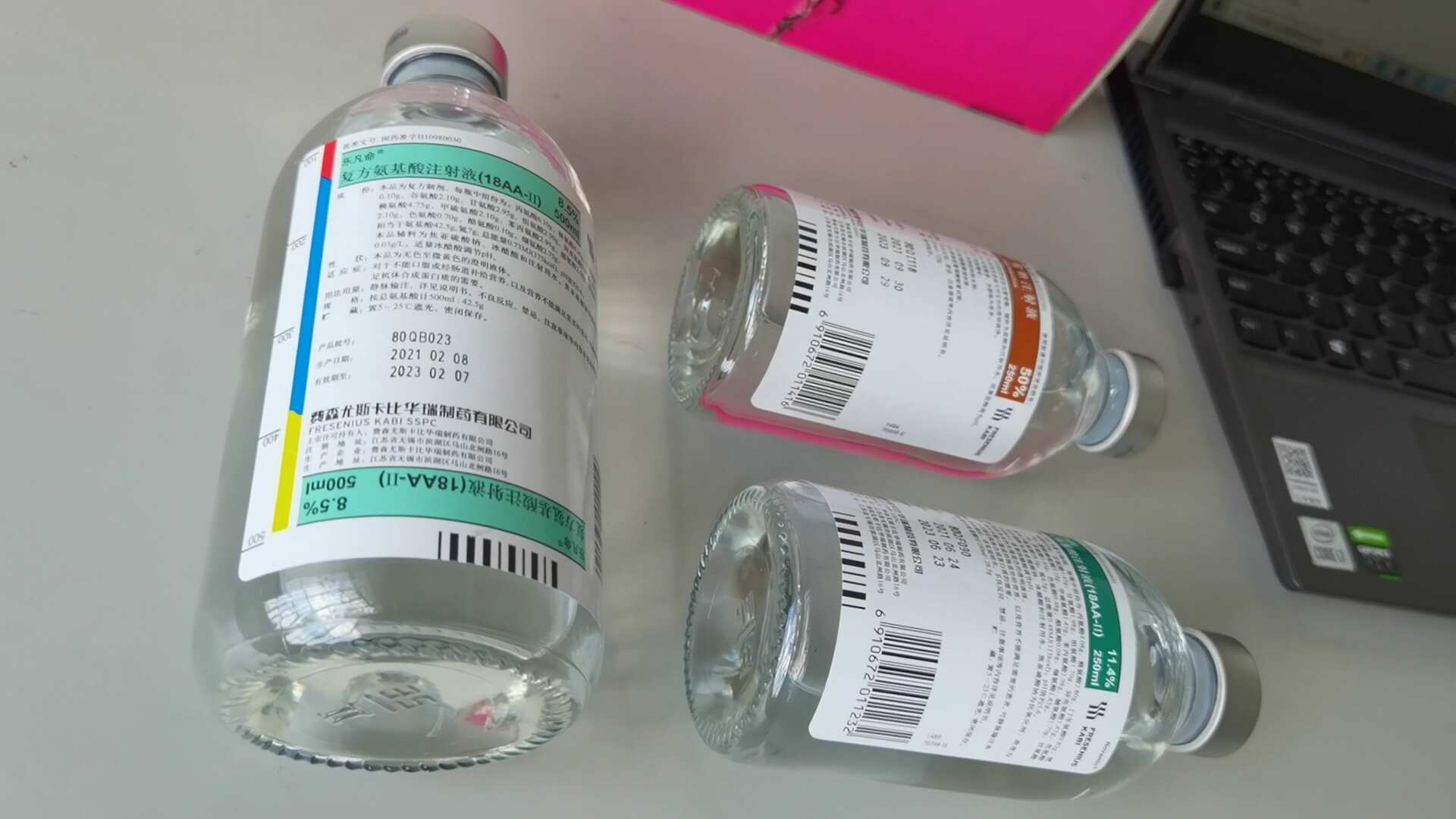

Supplement: S1 Dataset — (ZIP) [file pone.0298109.s001.zip › minimal data set/VOC2007/images/1106.jpg]

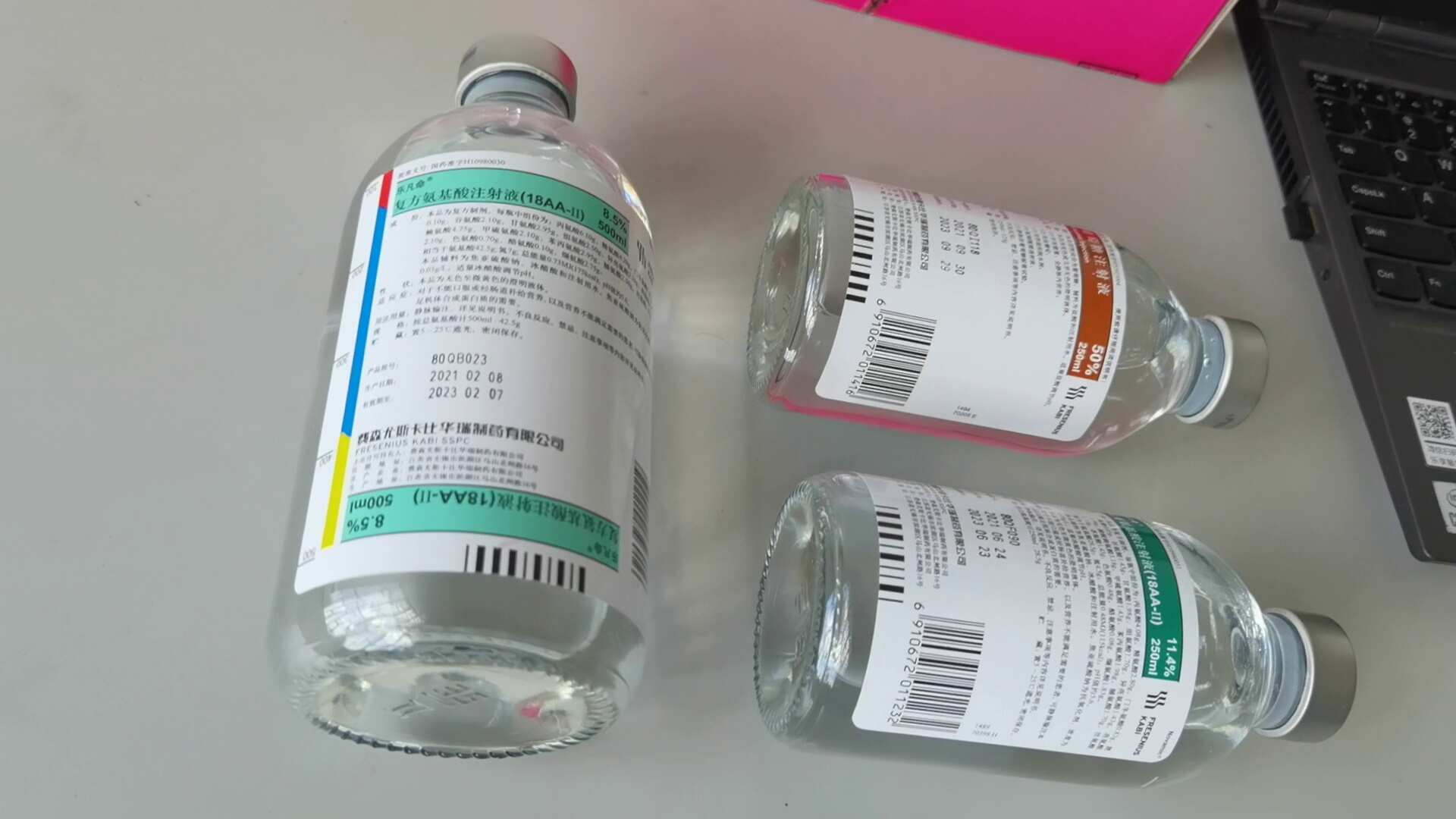

Supplement: S1 Dataset — (ZIP) [file pone.0298109.s001.zip › minimal data set/VOC2007/images/1107.jpg]

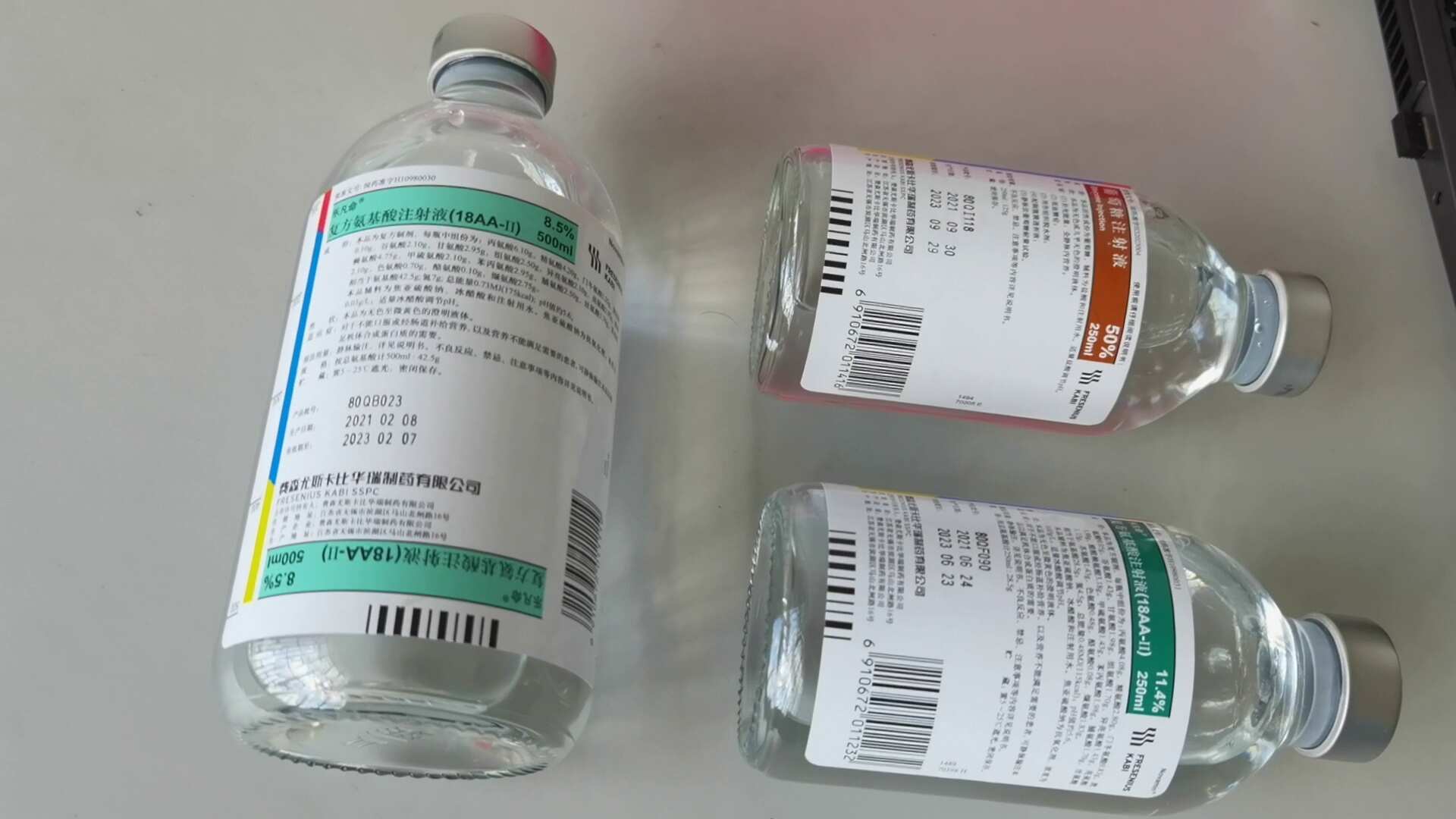

Supplement: S1 Dataset — (ZIP) [file pone.0298109.s001.zip › minimal data set/VOC2007/images/1108.jpg]

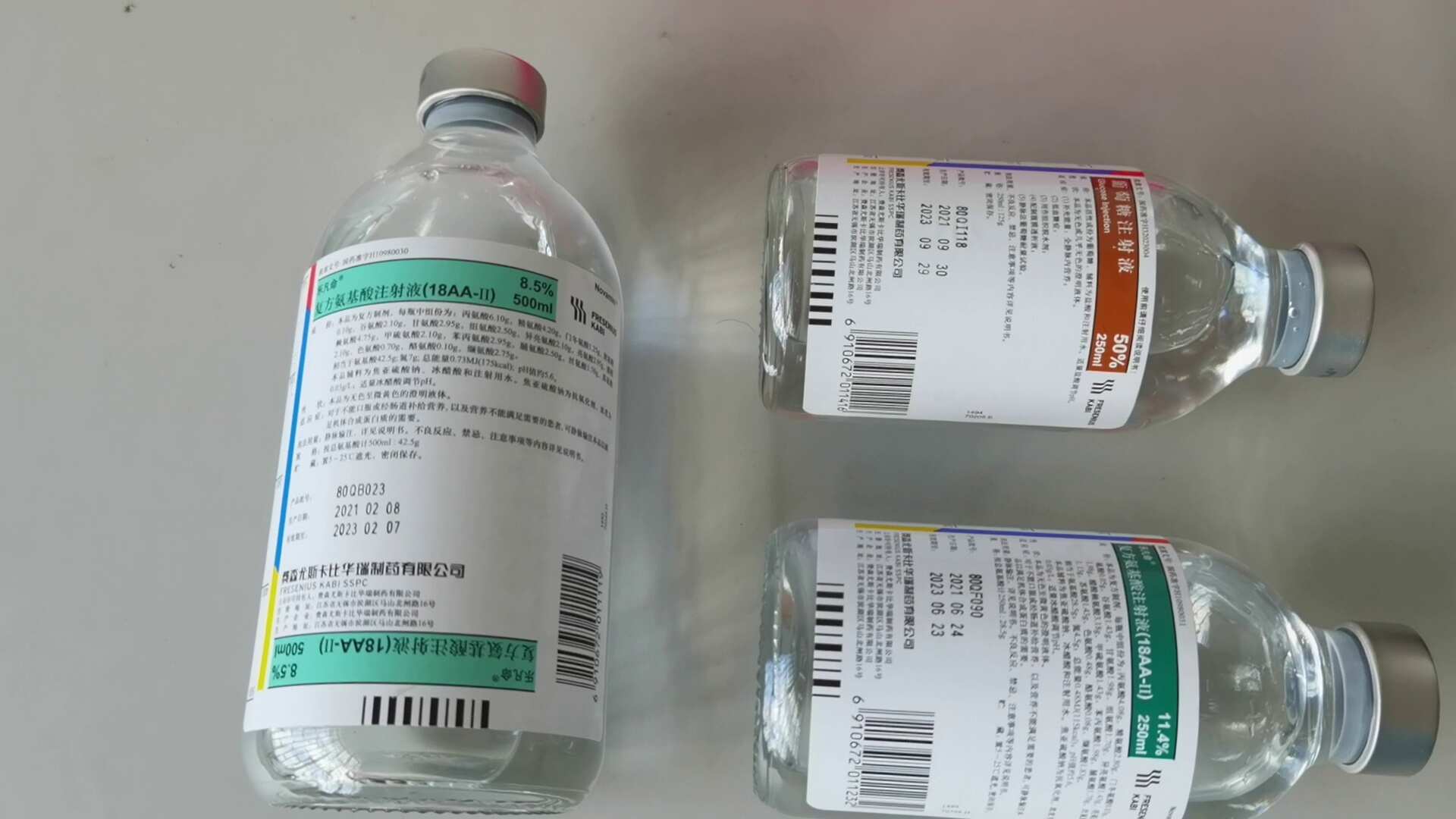

Supplement: S1 Dataset — (ZIP) [file pone.0298109.s001.zip › minimal data set/VOC2007/images/1109.jpg]

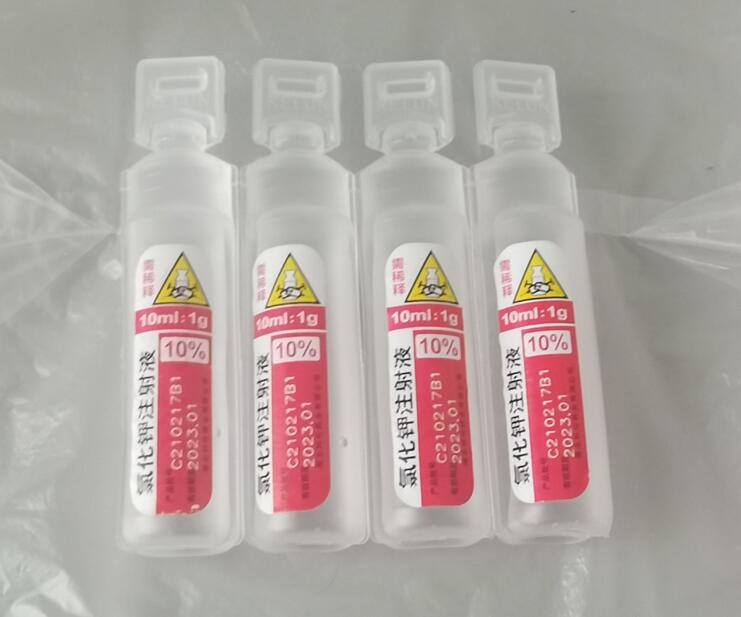

Supplement: S1 Dataset — (ZIP) [file pone.0298109.s001.zip › minimal data set/VOC2007/images/111.jpg]

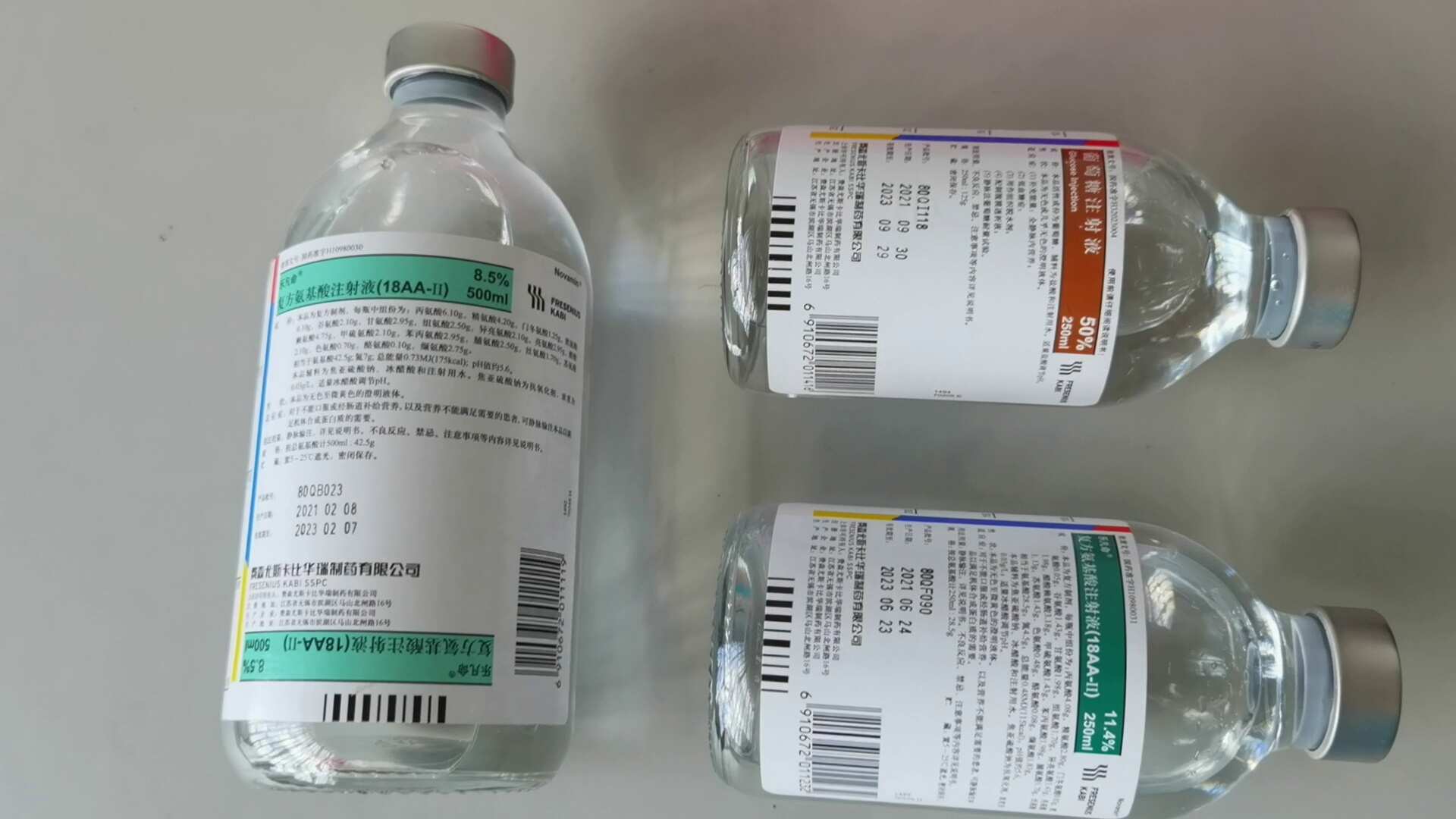

Supplement: S1 Dataset — (ZIP) [file pone.0298109.s001.zip › minimal data set/VOC2007/images/1110.jpg]

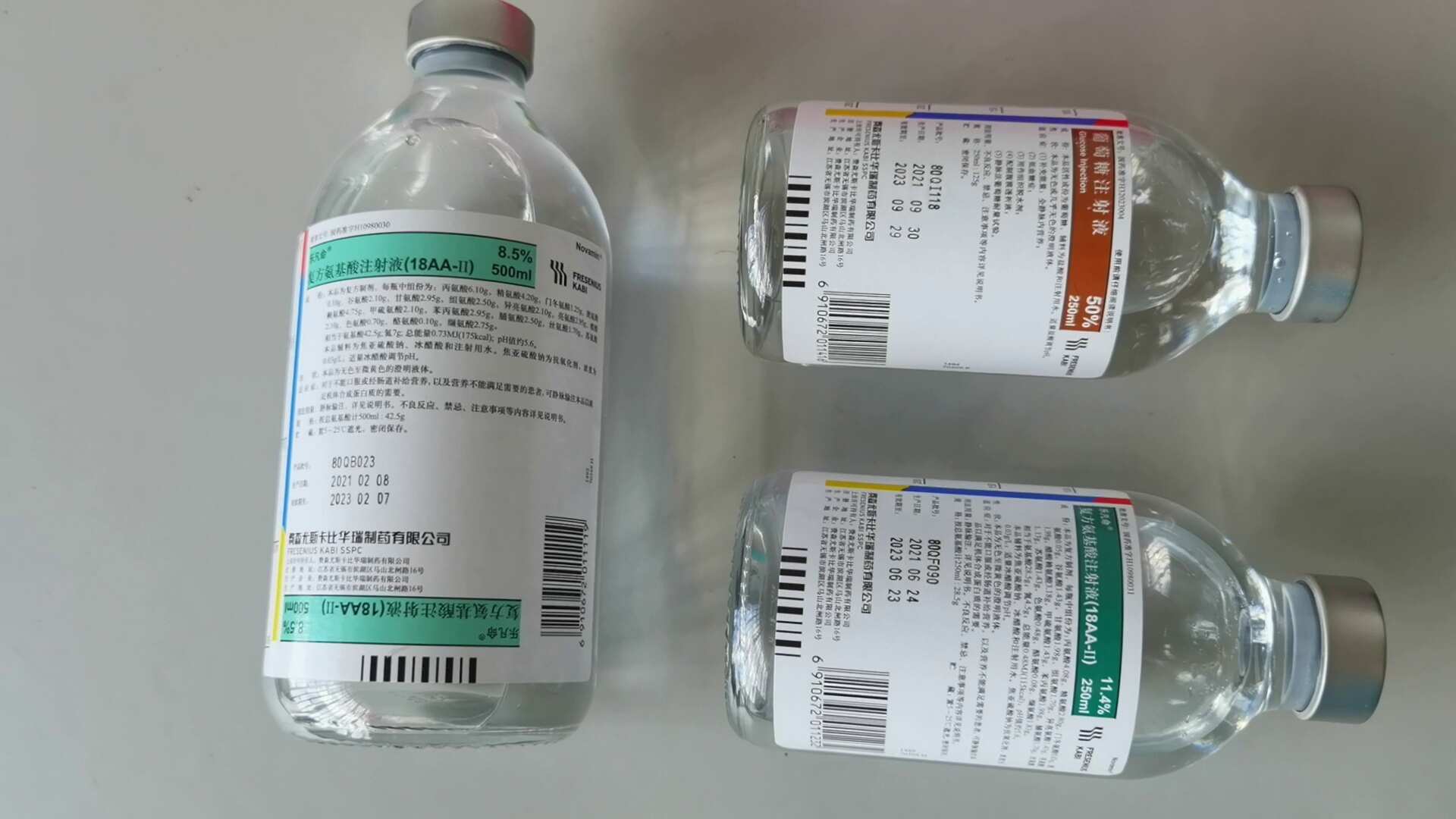

Supplement: S1 Dataset — (ZIP) [file pone.0298109.s001.zip › minimal data set/VOC2007/images/1111.jpg]

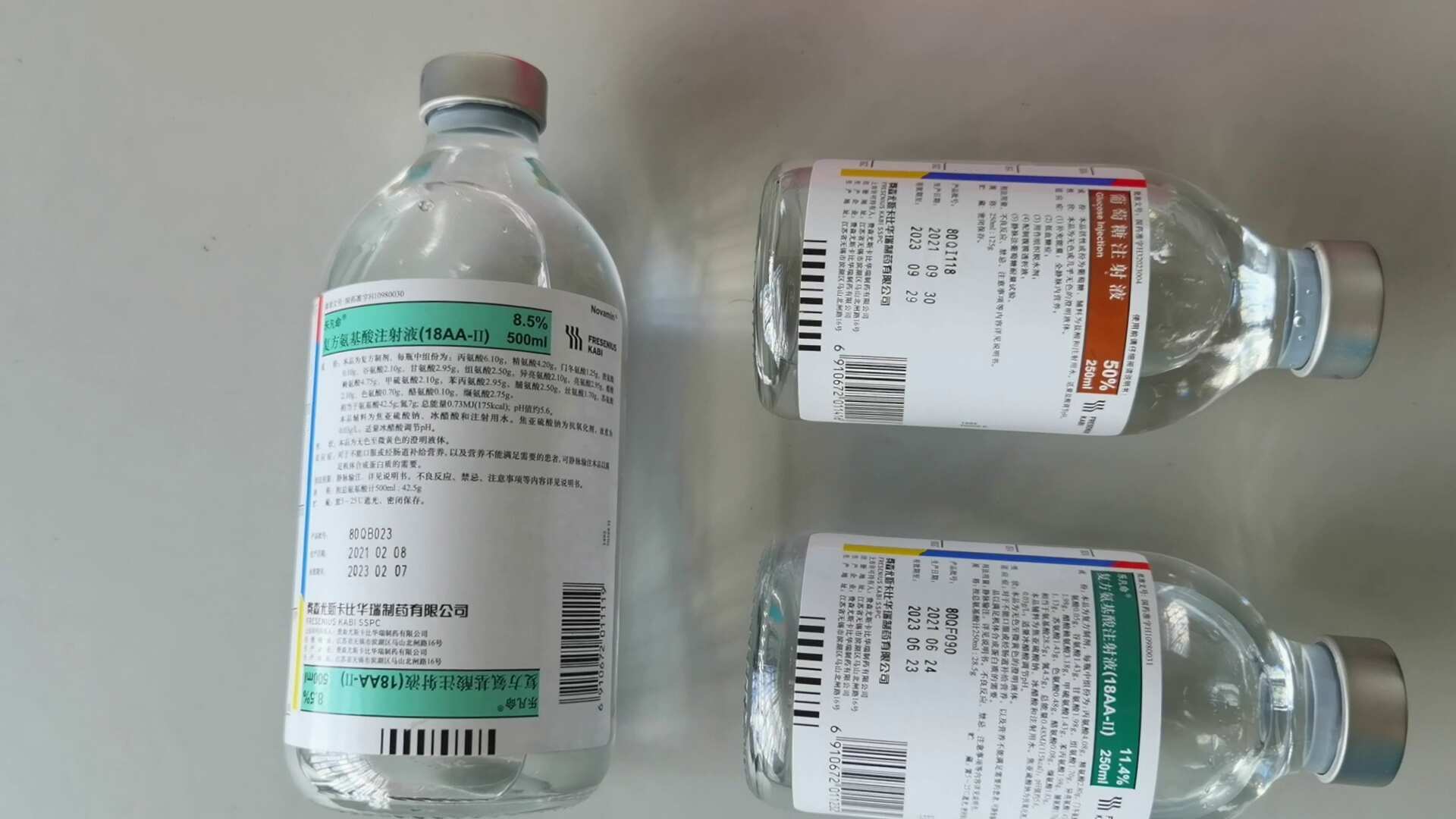

Supplement: S1 Dataset — (ZIP) [file pone.0298109.s001.zip › minimal data set/VOC2007/images/1112.jpg]

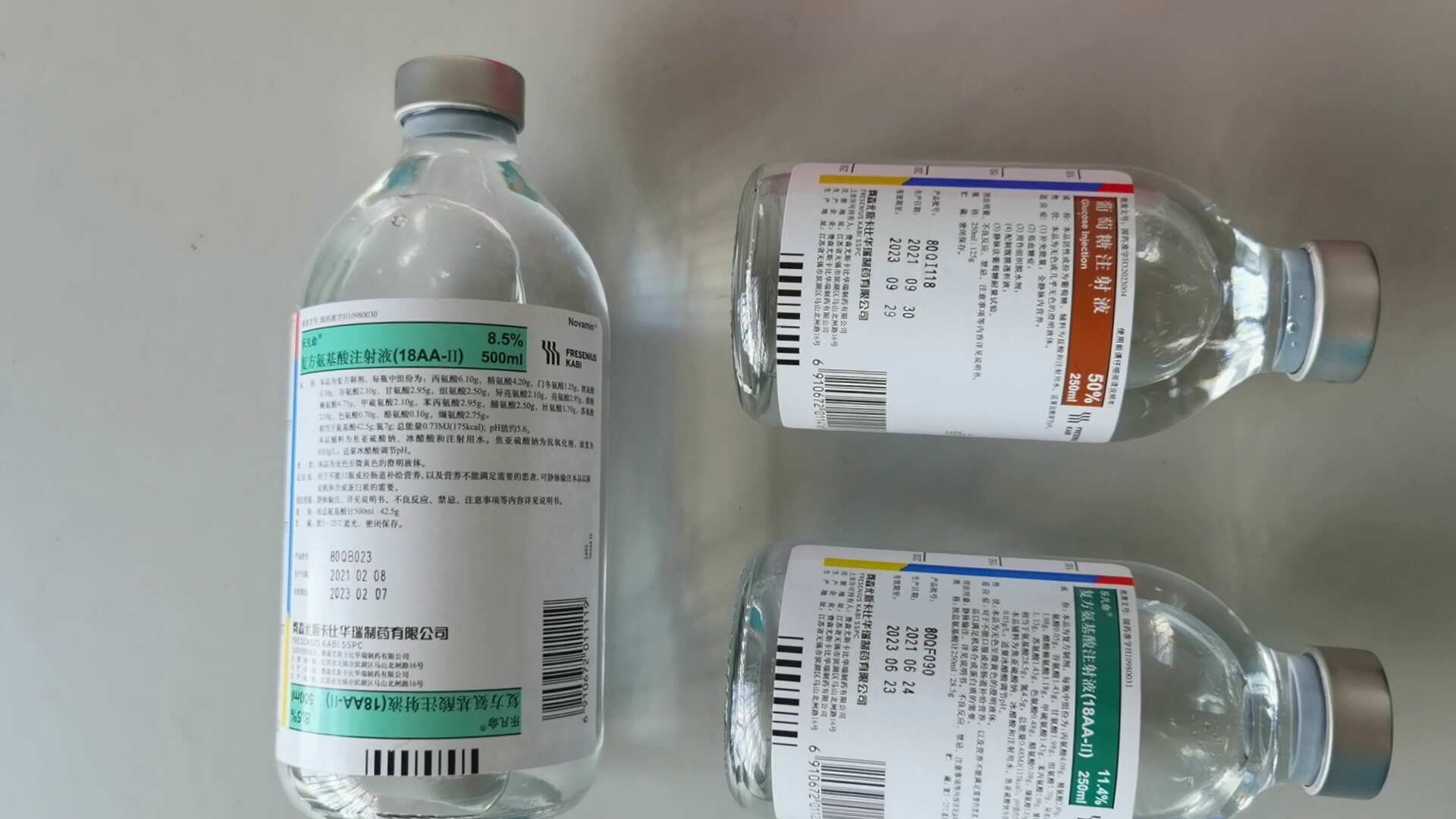

Supplement: S1 Dataset — (ZIP) [file pone.0298109.s001.zip › minimal data set/VOC2007/images/1113.jpg]

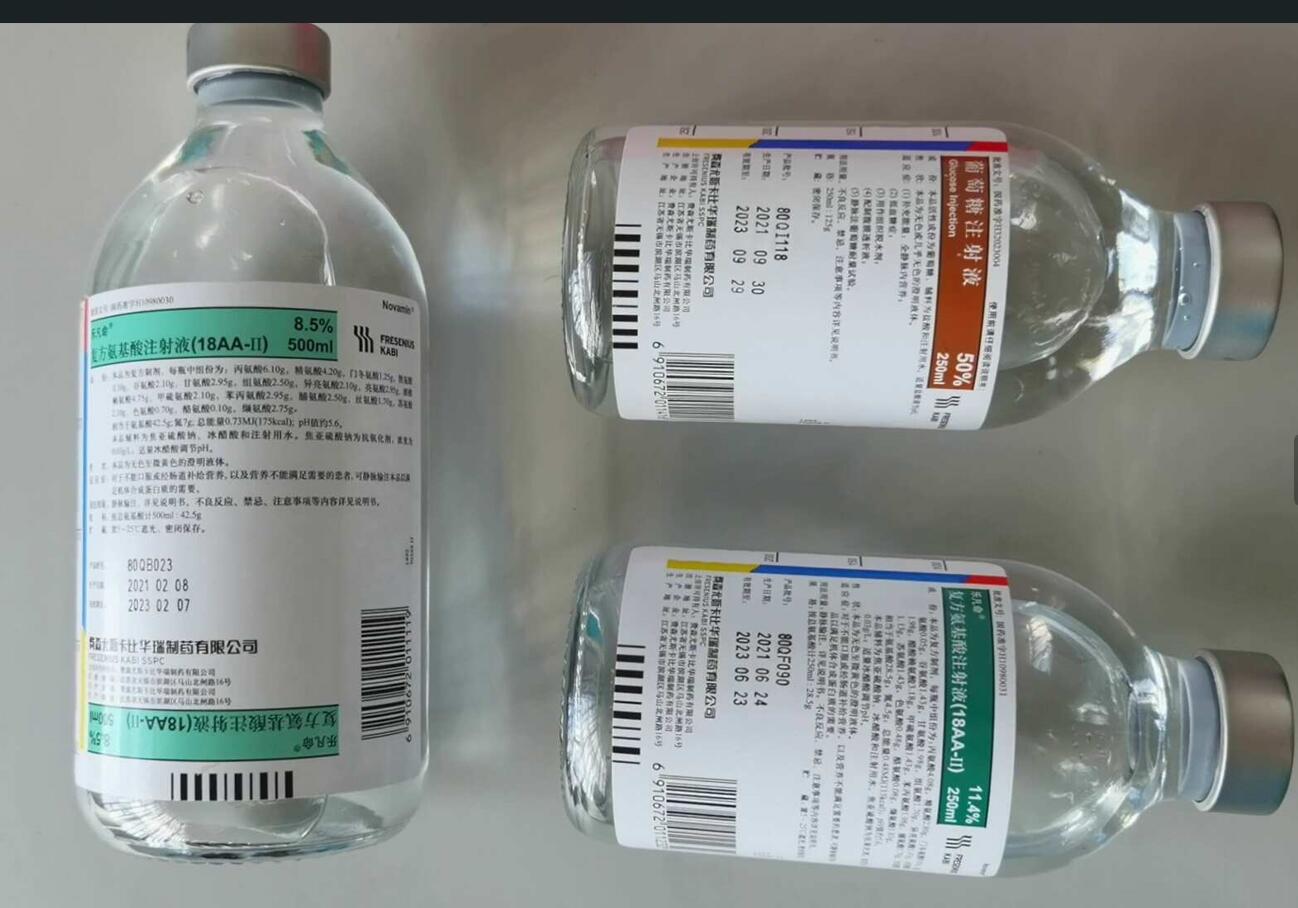

Supplement: S1 Dataset — (ZIP) [file pone.0298109.s001.zip › minimal data set/VOC2007/images/1114.jpg]

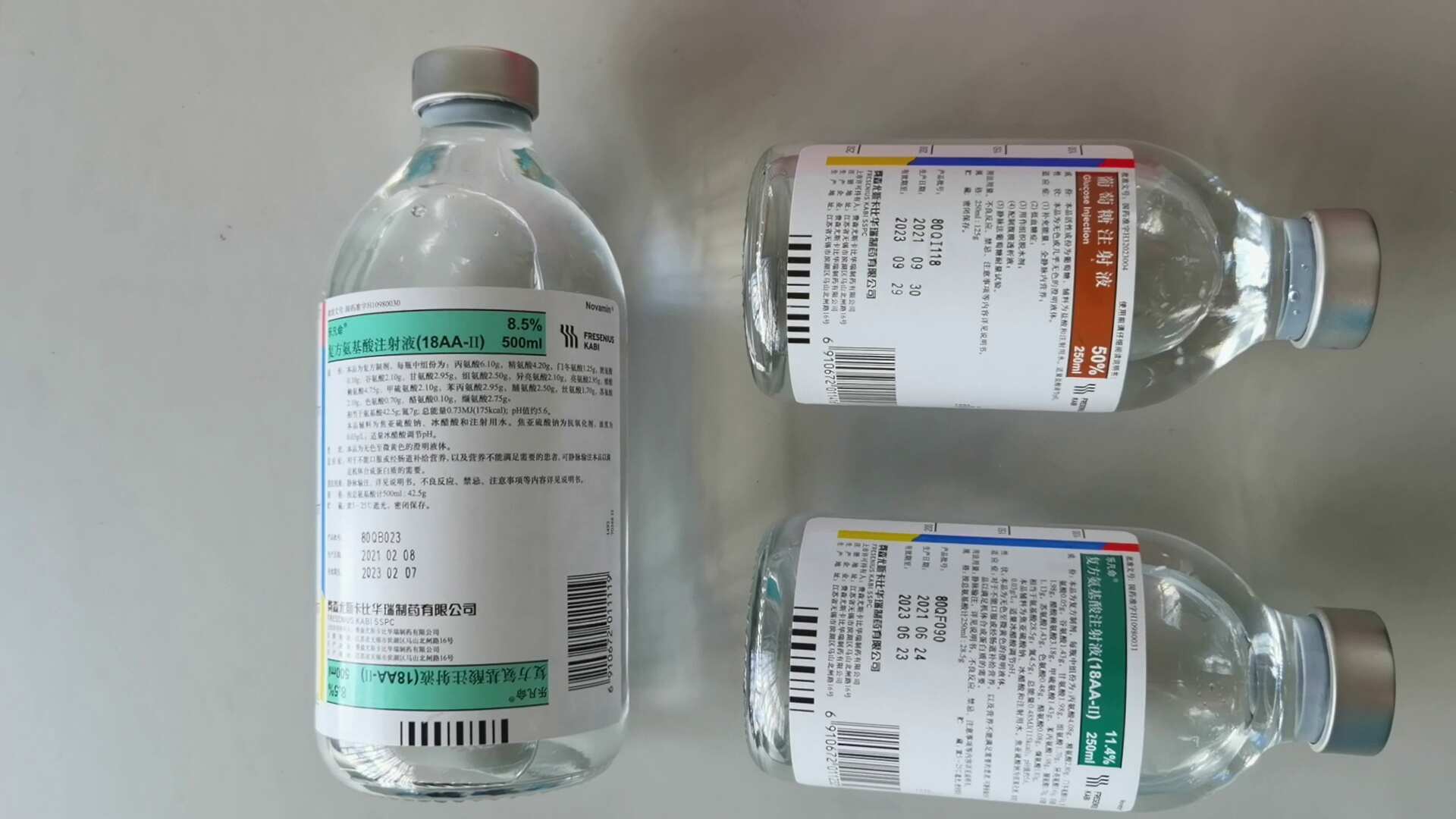

Supplement: S1 Dataset — (ZIP) [file pone.0298109.s001.zip › minimal data set/VOC2007/images/1115.jpg]

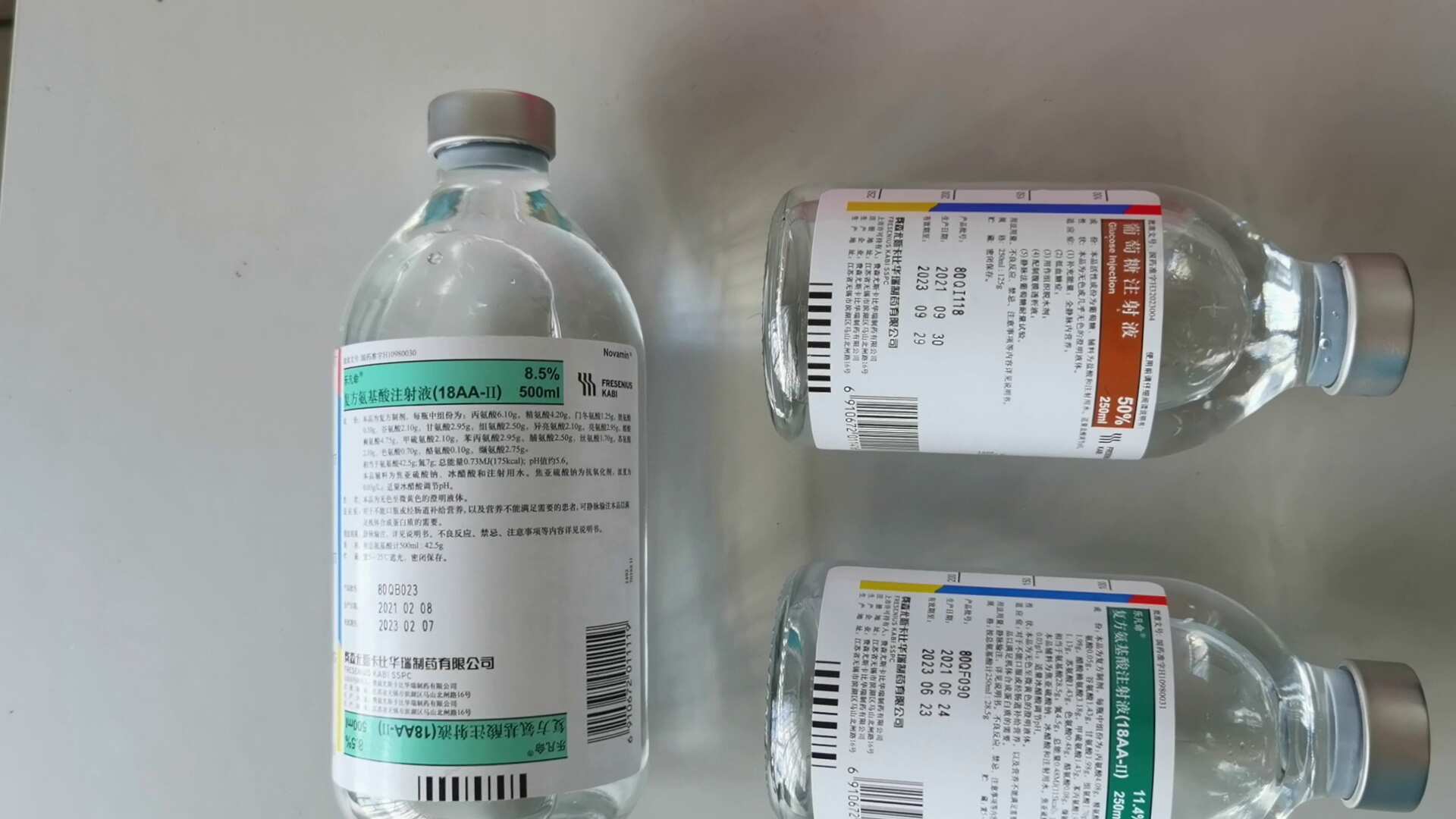

Supplement: S1 Dataset — (ZIP) [file pone.0298109.s001.zip › minimal data set/VOC2007/images/1116.jpg]

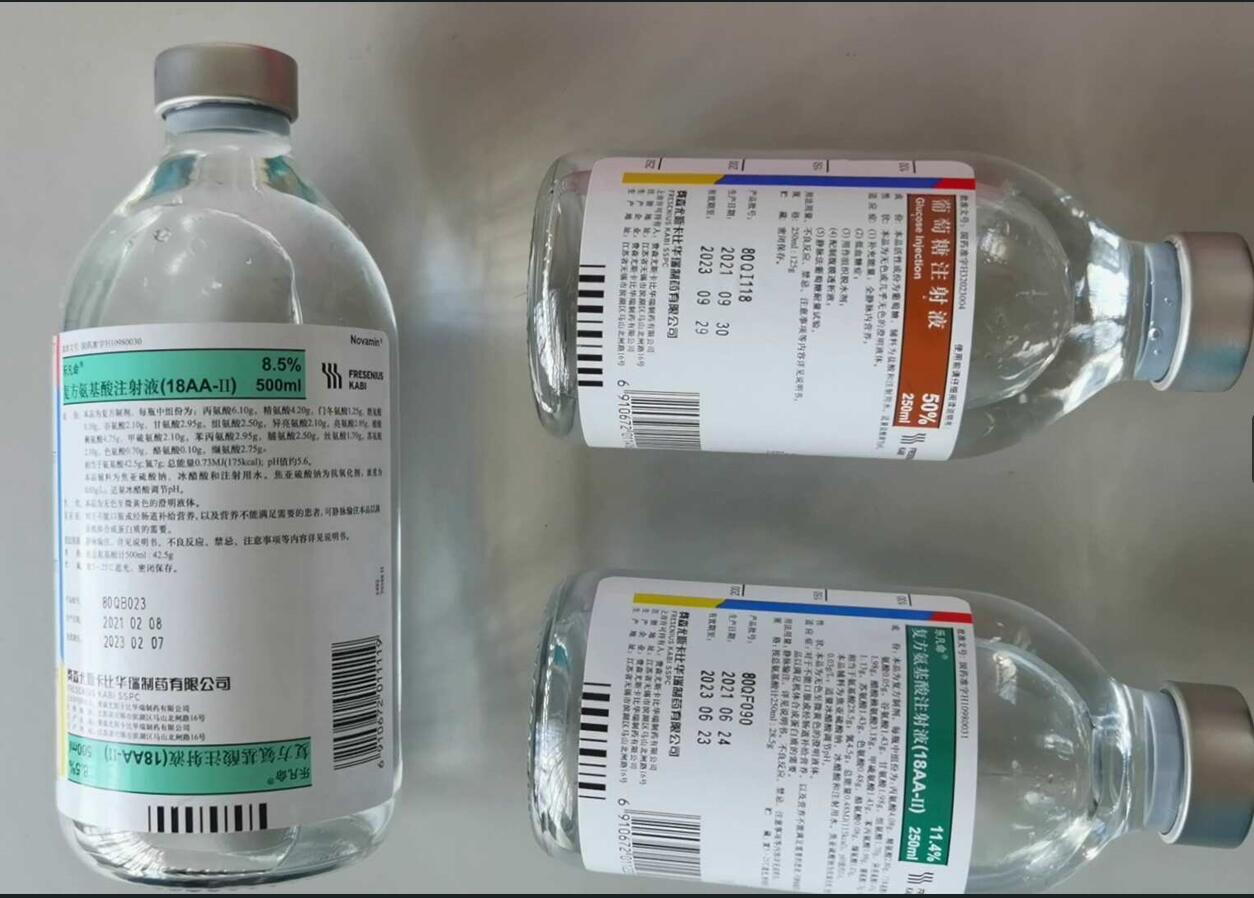

Supplement: S1 Dataset — (ZIP) [file pone.0298109.s001.zip › minimal data set/VOC2007/images/1117.jpg]

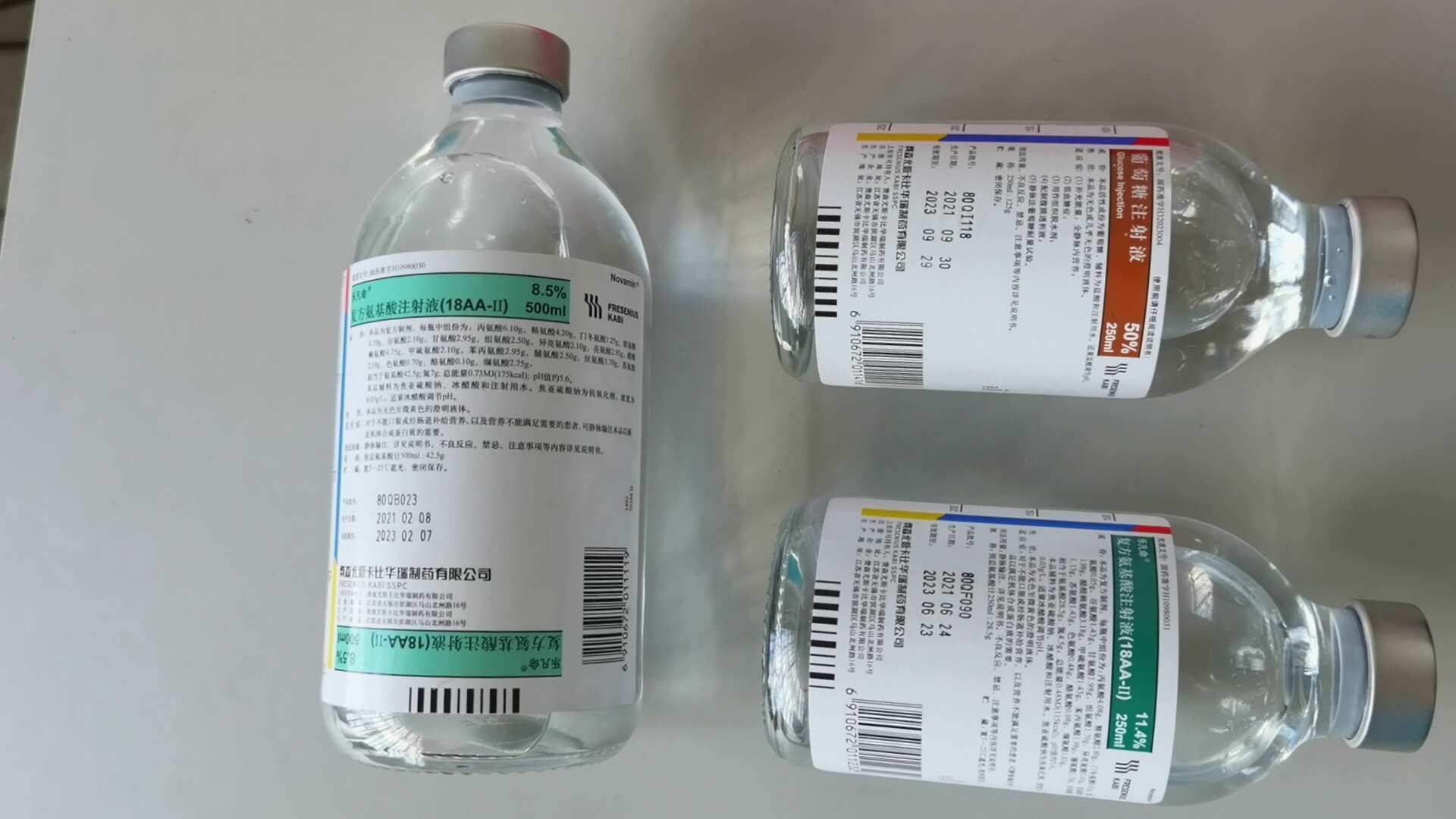

Supplement: S1 Dataset — (ZIP) [file pone.0298109.s001.zip › minimal data set/VOC2007/images/1118.jpg]

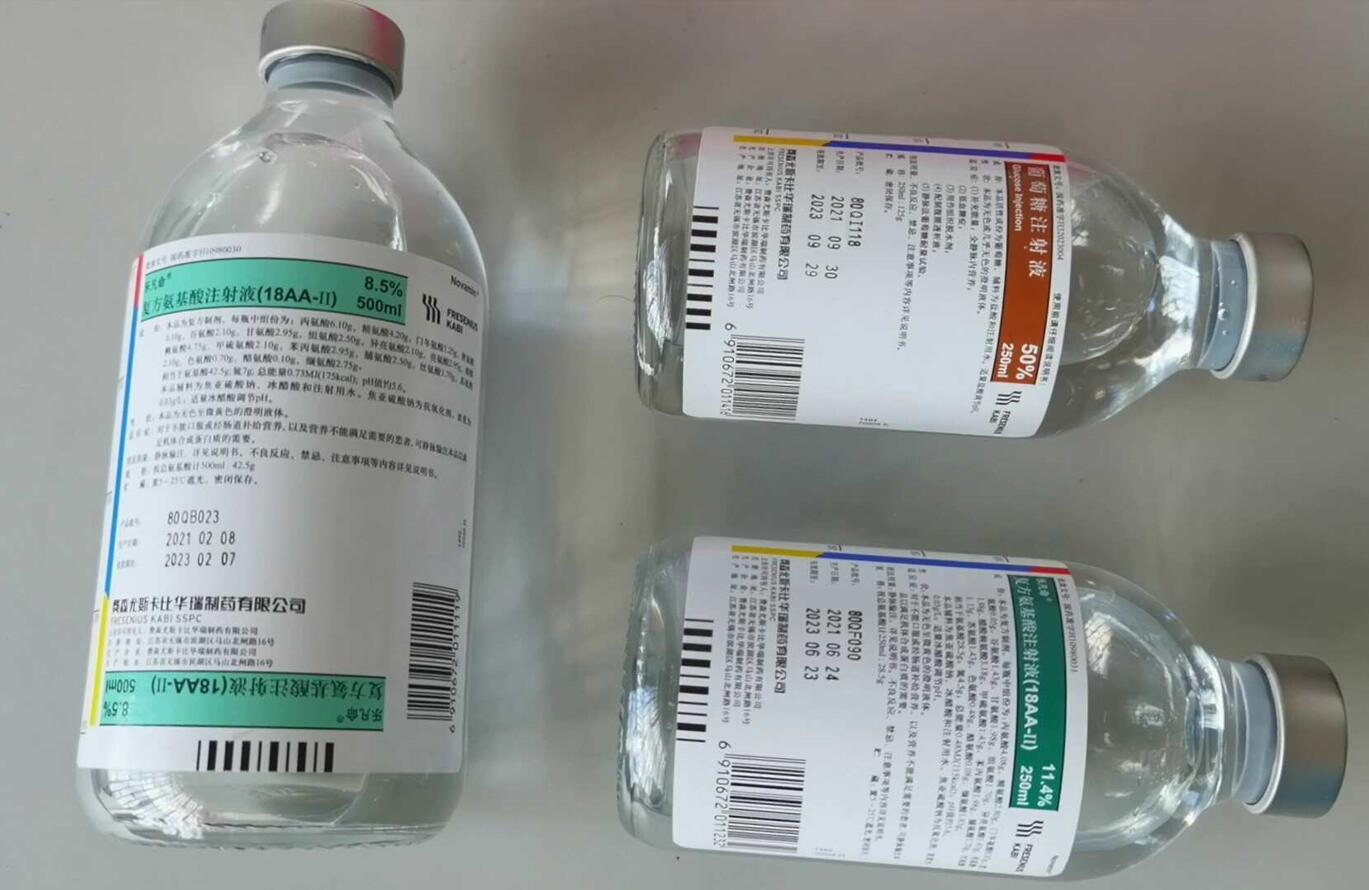

Supplement: S1 Dataset — (ZIP) [file pone.0298109.s001.zip › minimal data set/VOC2007/images/1119.jpg]

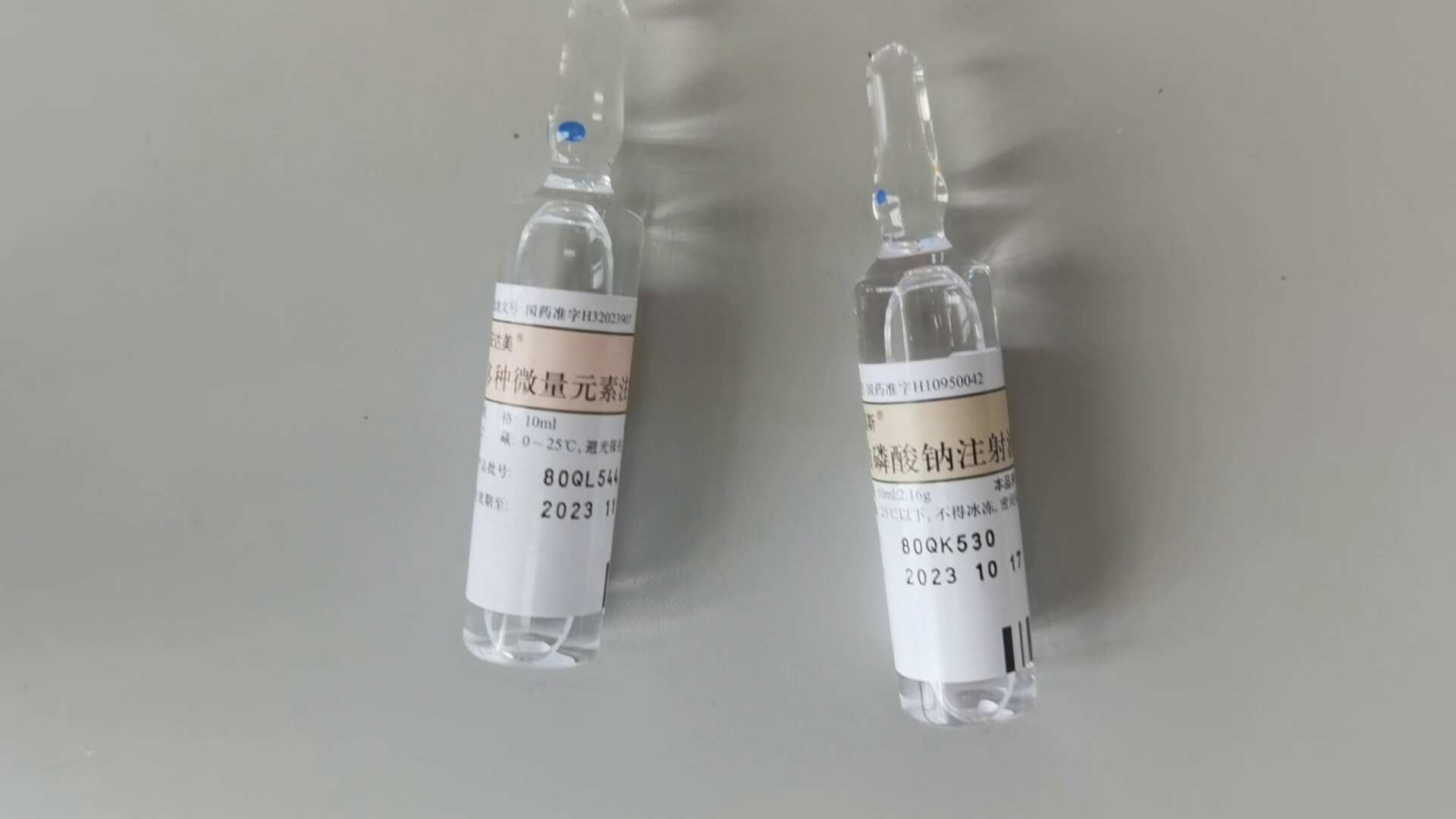

Supplement: S1 Dataset — (ZIP) [file pone.0298109.s001.zip › minimal data set/VOC2007/images/112.jpg]

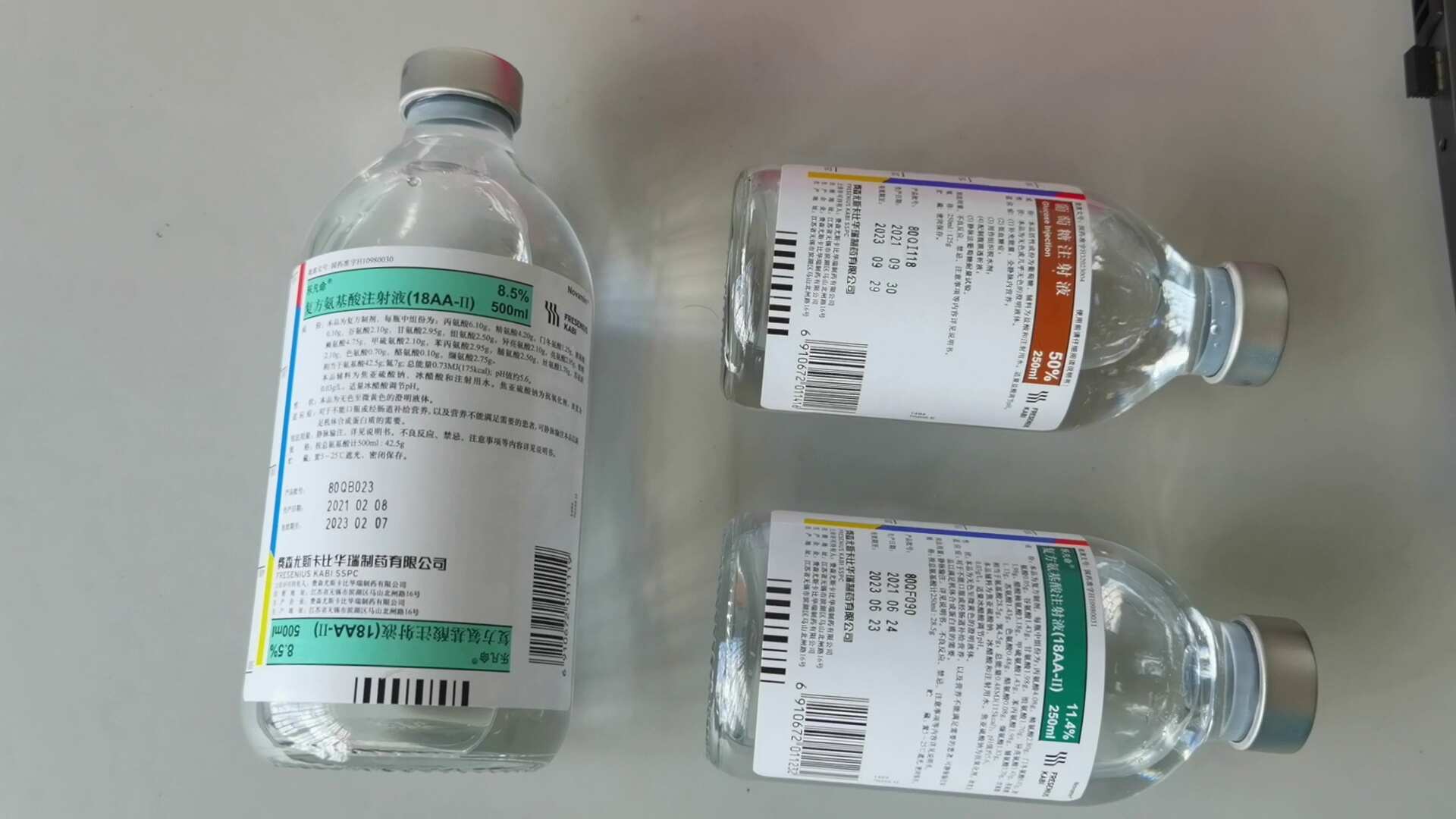

Supplement: S1 Dataset — (ZIP) [file pone.0298109.s001.zip › minimal data set/VOC2007/images/1120.jpg]

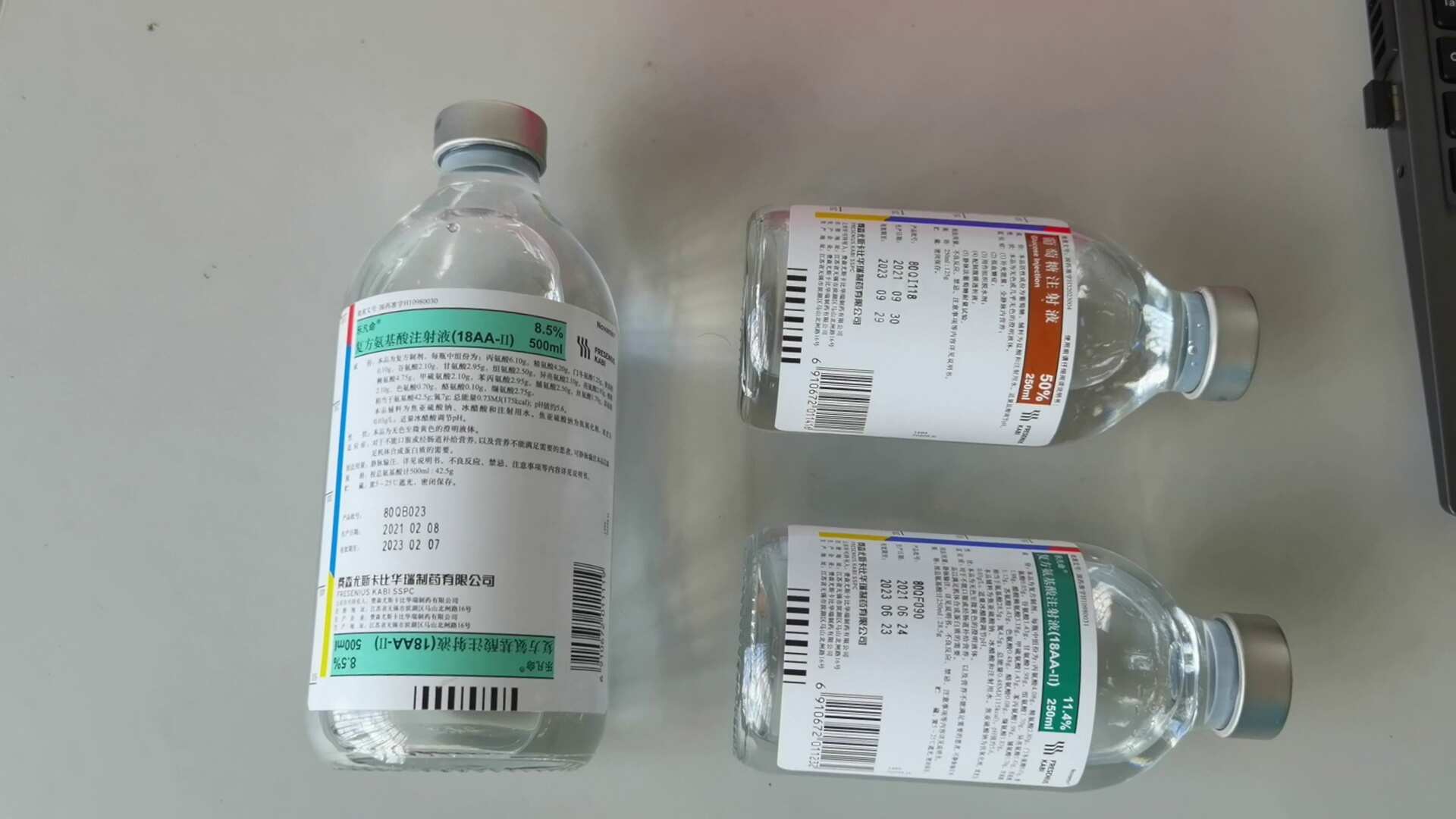

Supplement: S1 Dataset — (ZIP) [file pone.0298109.s001.zip › minimal data set/VOC2007/images/1121.jpg]

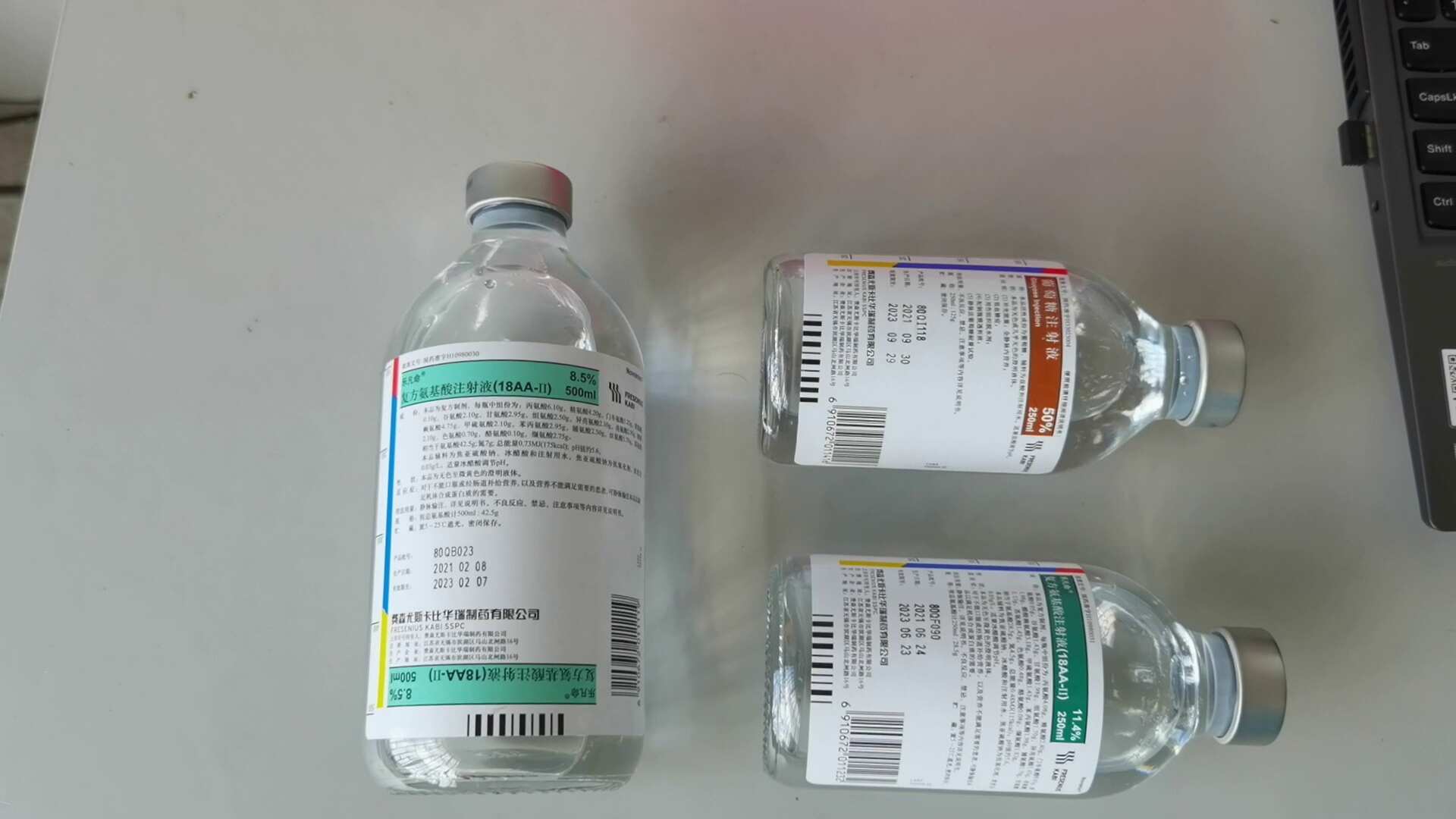

Supplement: S1 Dataset — (ZIP) [file pone.0298109.s001.zip › minimal data set/VOC2007/images/1122.jpg]

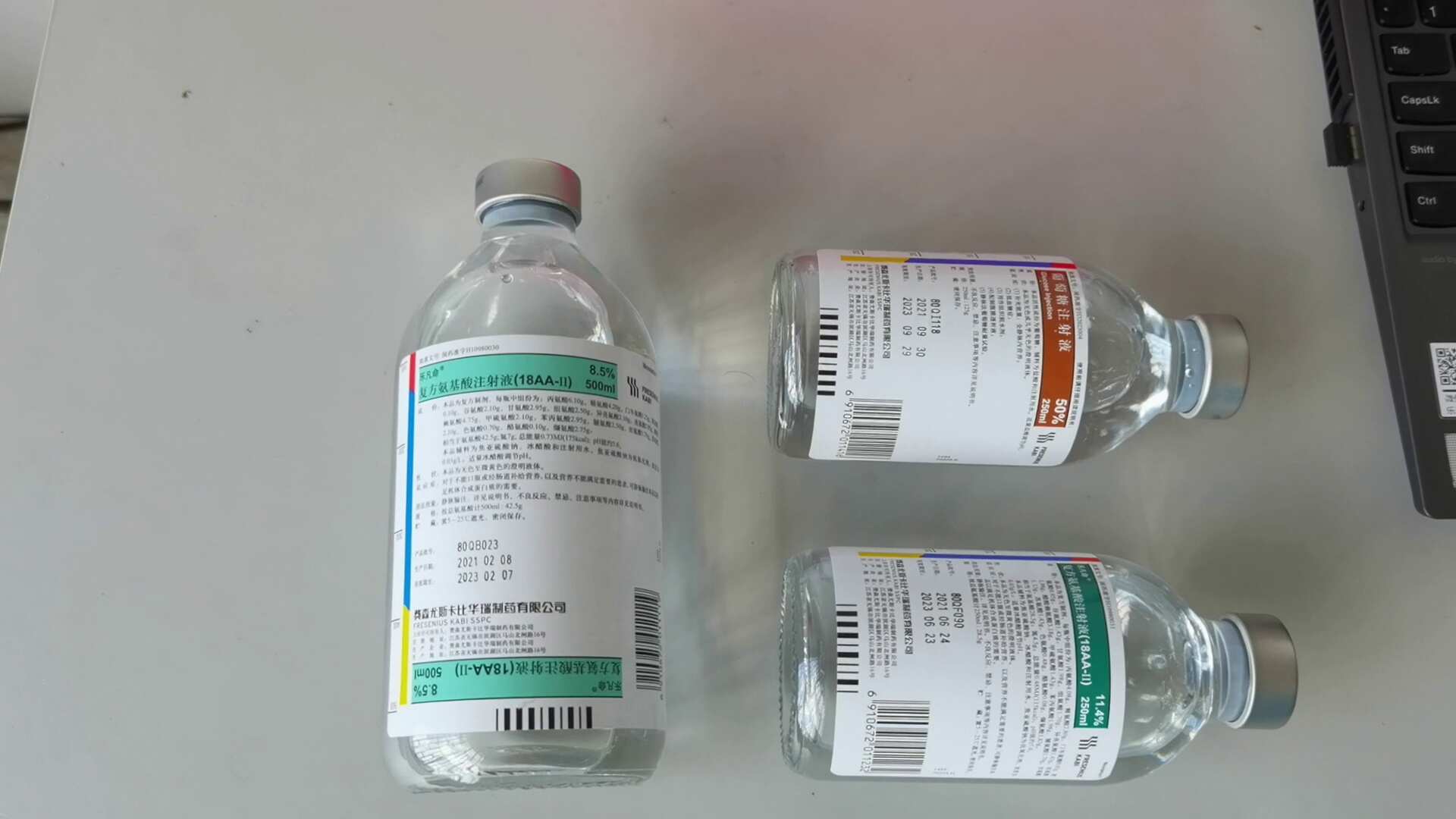

Supplement: S1 Dataset — (ZIP) [file pone.0298109.s001.zip › minimal data set/VOC2007/images/1123.jpg]

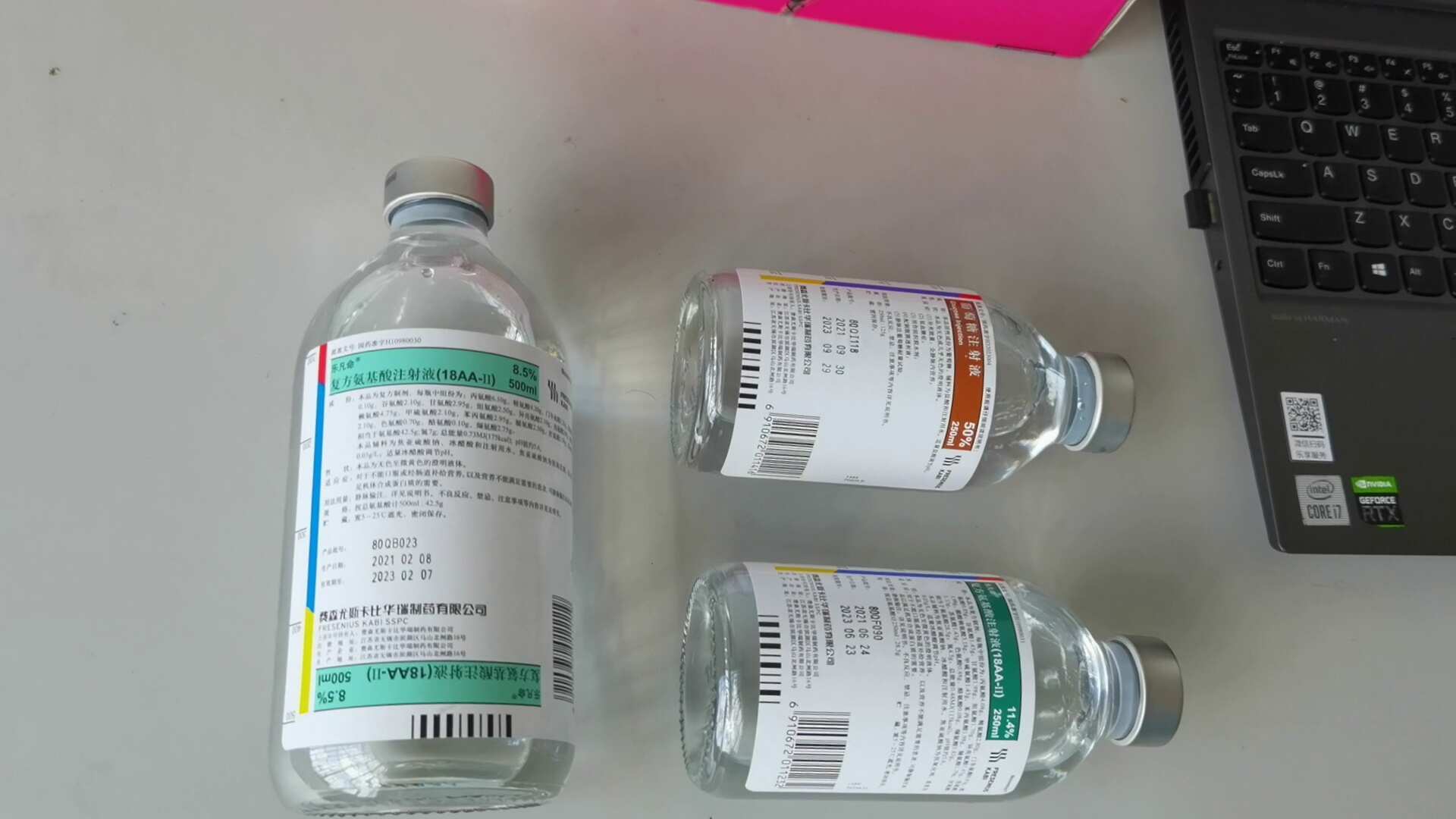

Supplement: S1 Dataset — (ZIP) [file pone.0298109.s001.zip › minimal data set/VOC2007/images/1124.jpg]

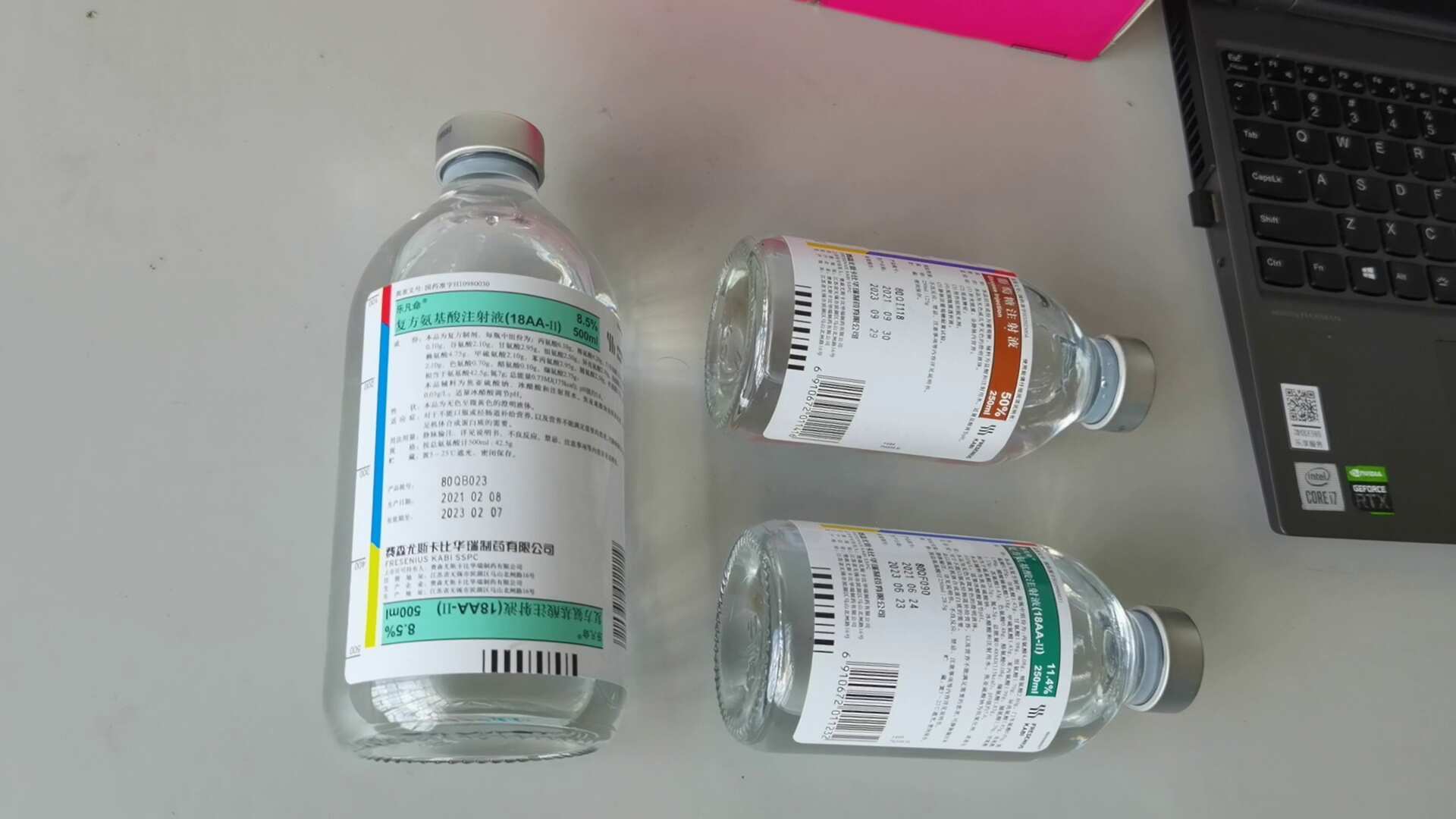

Supplement: S1 Dataset — (ZIP) [file pone.0298109.s001.zip › minimal data set/VOC2007/images/1125.jpg]

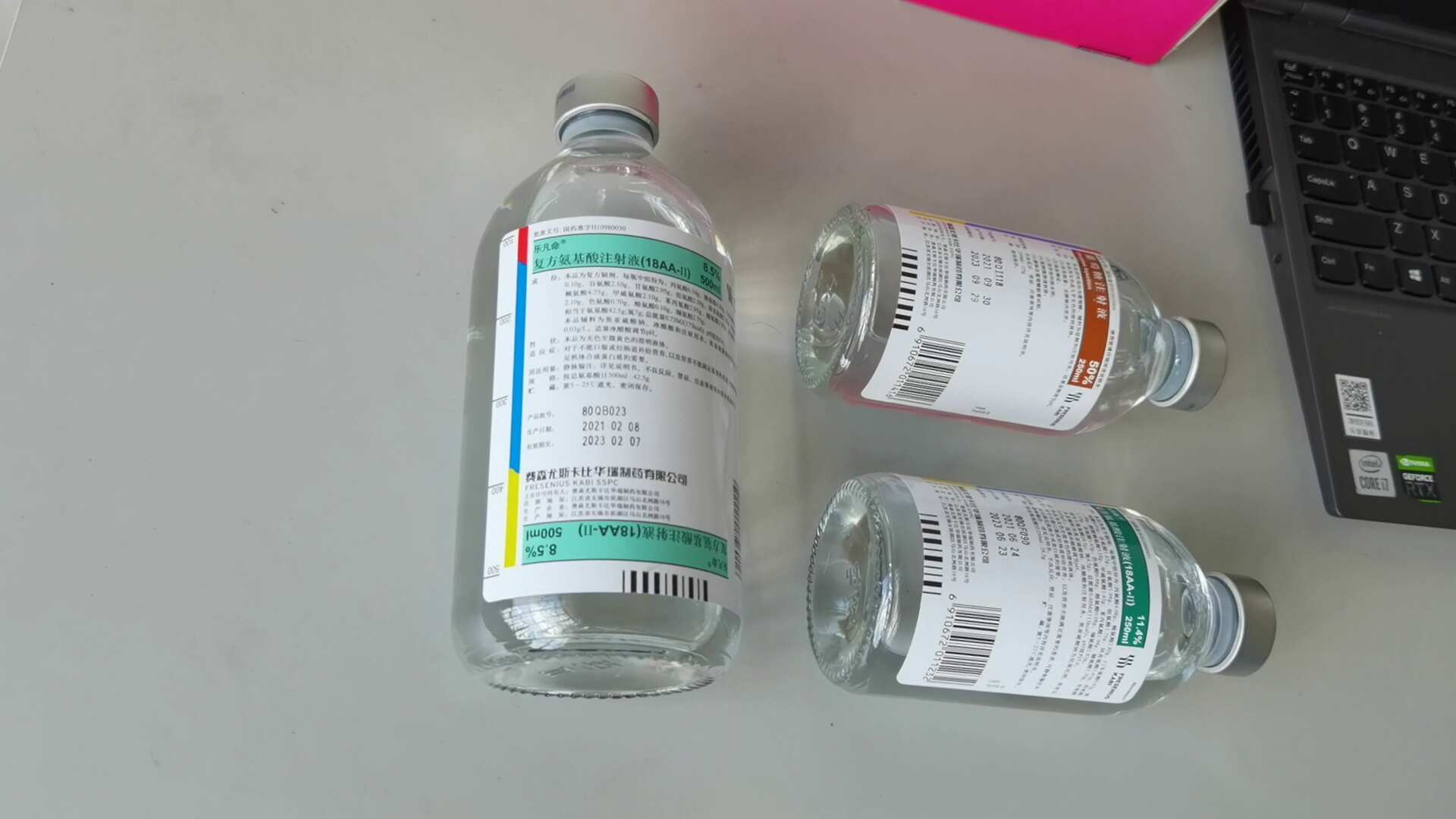

Supplement: S1 Dataset — (ZIP) [file pone.0298109.s001.zip › minimal data set/VOC2007/images/1126.jpg]

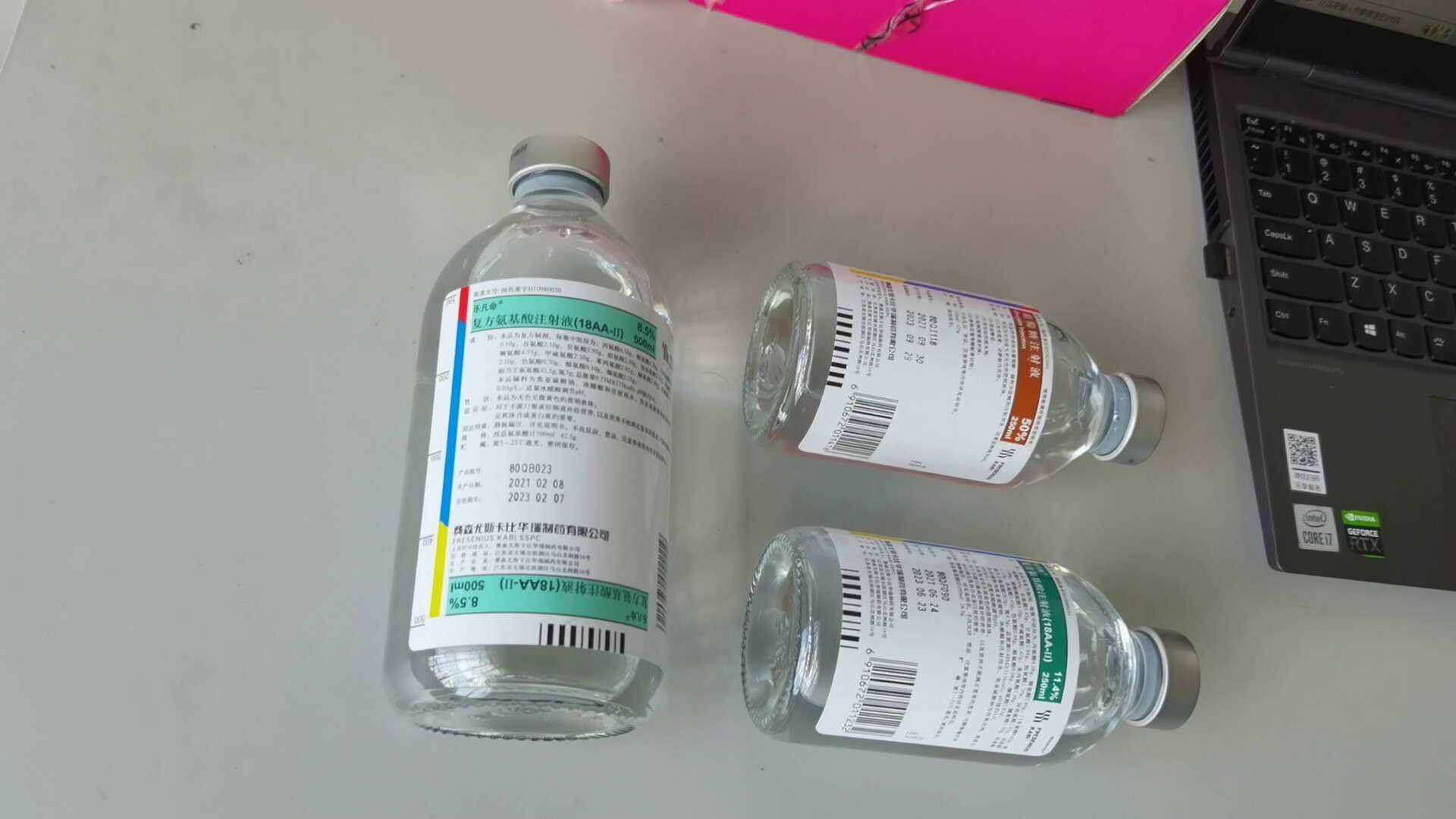

Supplement: S1 Dataset — (ZIP) [file pone.0298109.s001.zip › minimal data set/VOC2007/images/1127.jpg]

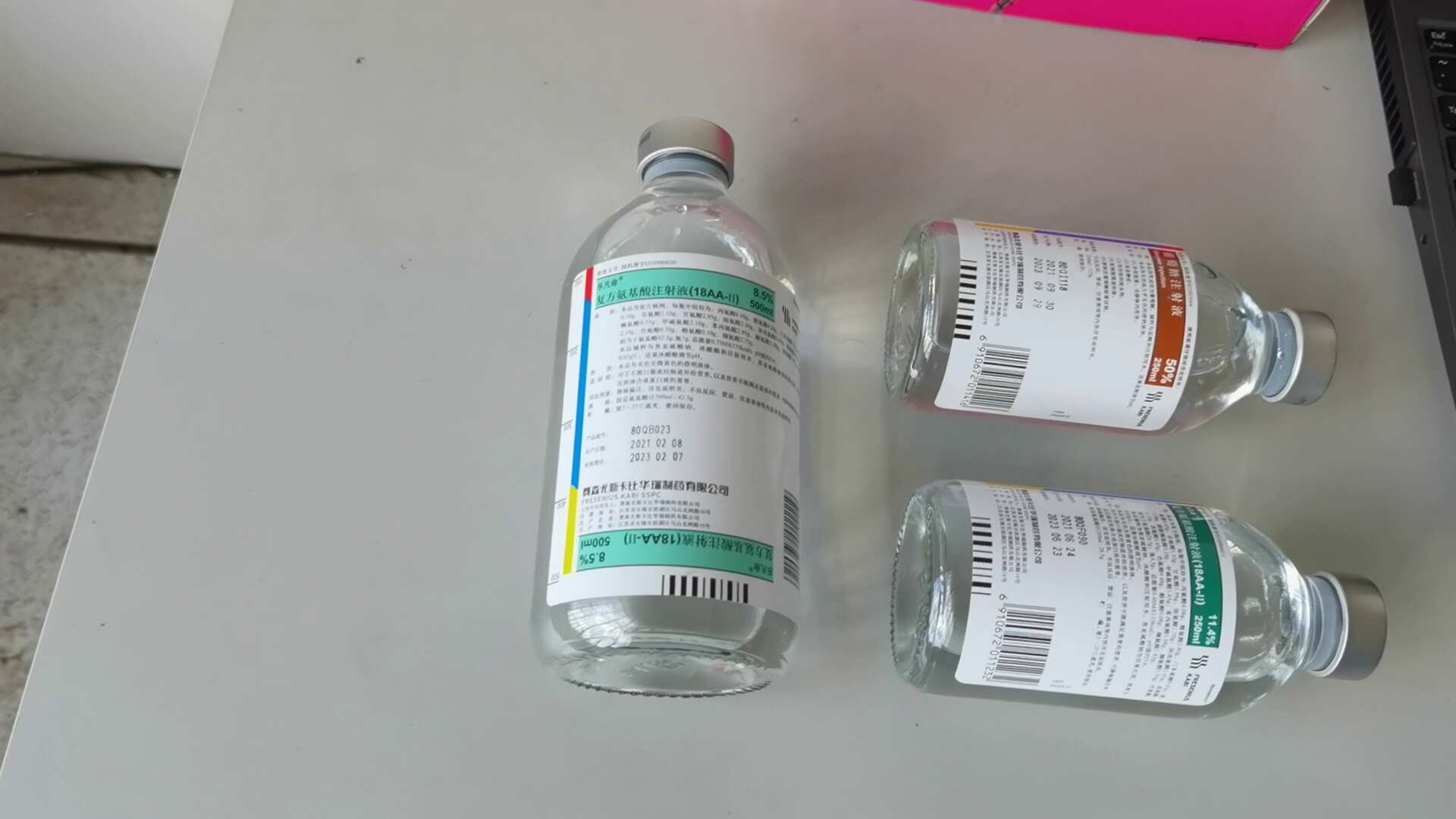

Supplement: S1 Dataset — (ZIP) [file pone.0298109.s001.zip › minimal data set/VOC2007/images/1128.jpg]

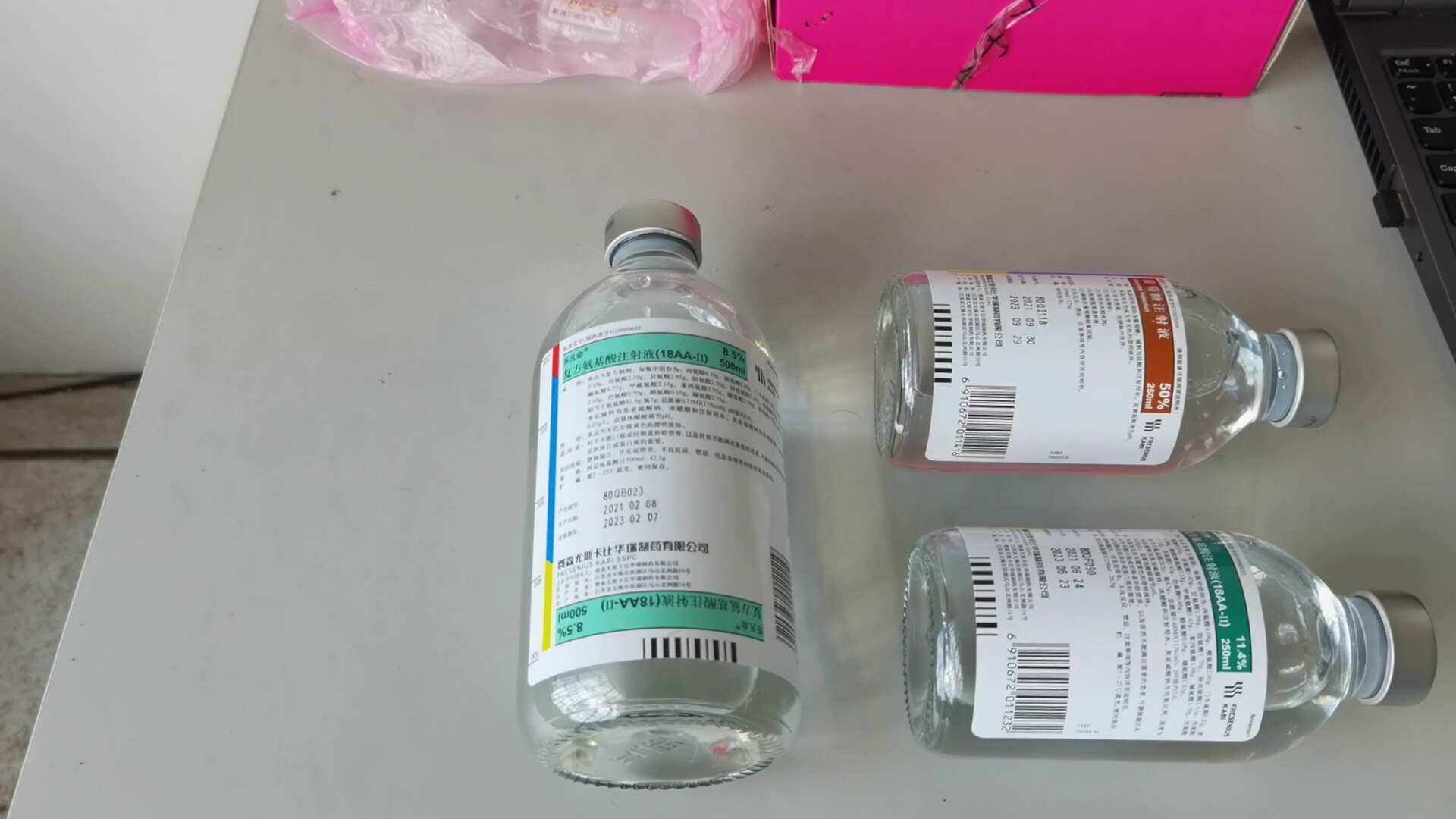

Supplement: S1 Dataset — (ZIP) [file pone.0298109.s001.zip › minimal data set/VOC2007/images/1129.jpg]

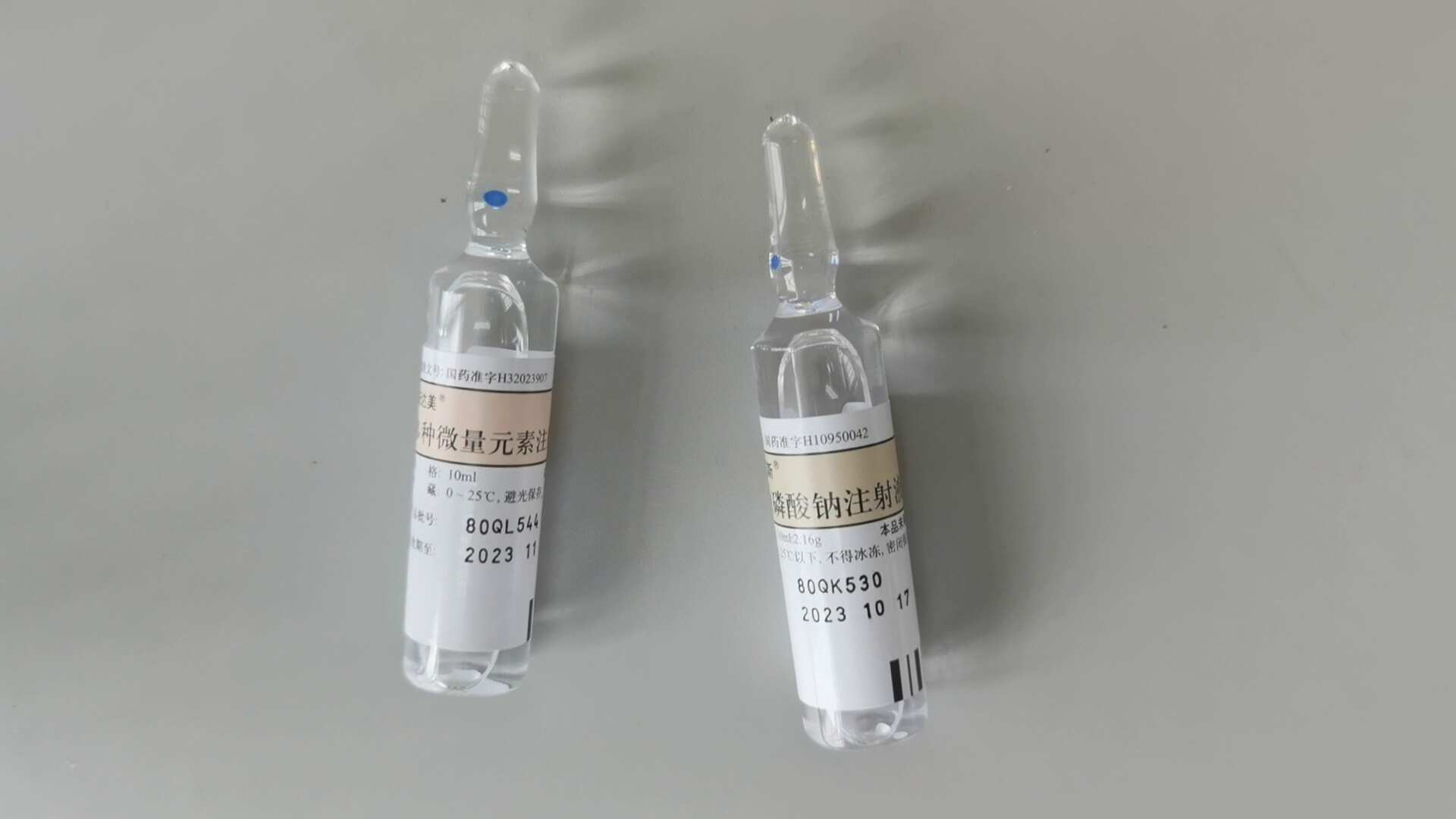

Supplement: S1 Dataset — (ZIP) [file pone.0298109.s001.zip › minimal data set/VOC2007/images/113.jpg]

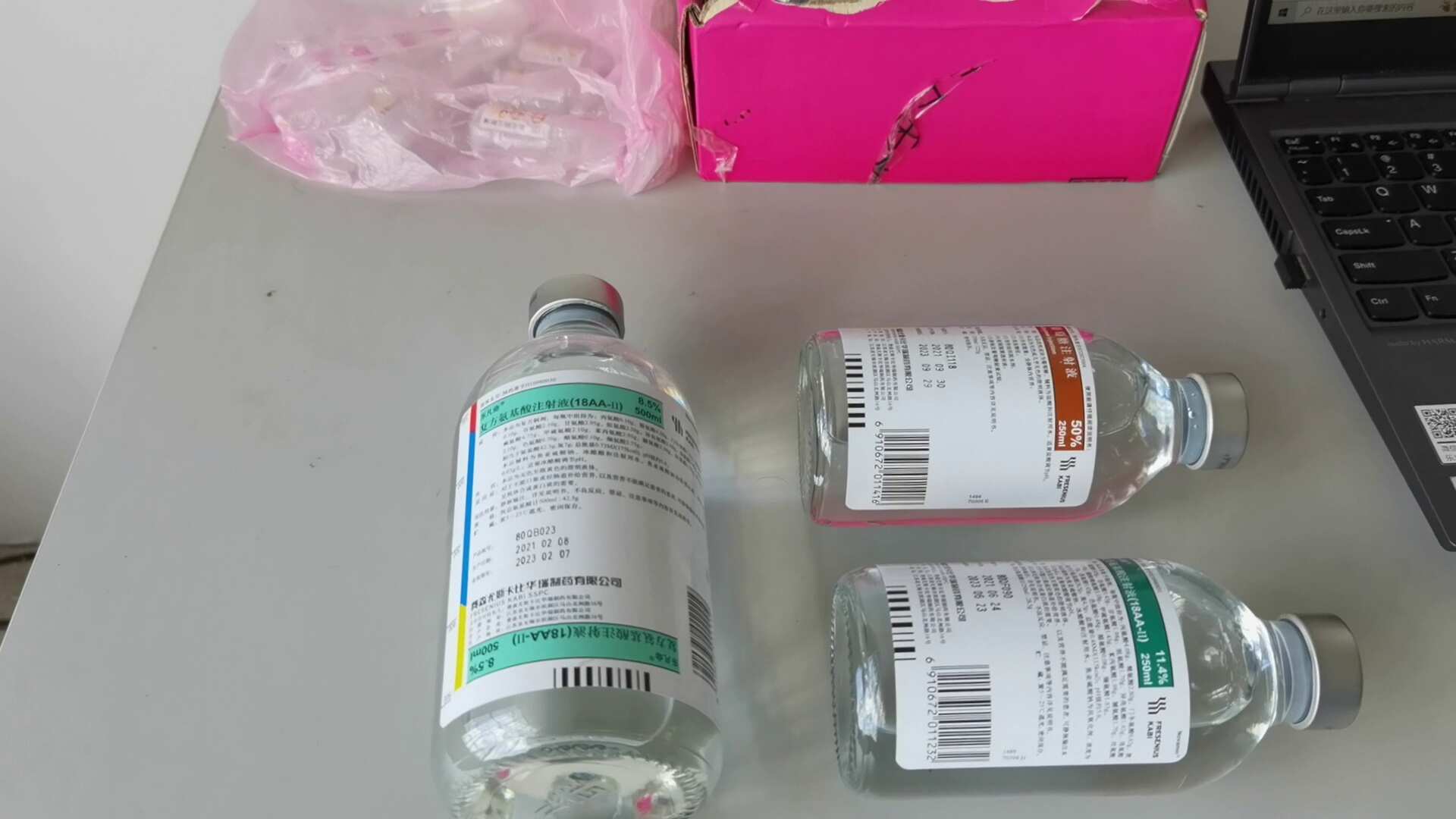

Supplement: S1 Dataset — (ZIP) [file pone.0298109.s001.zip › minimal data set/VOC2007/images/1130.jpg]

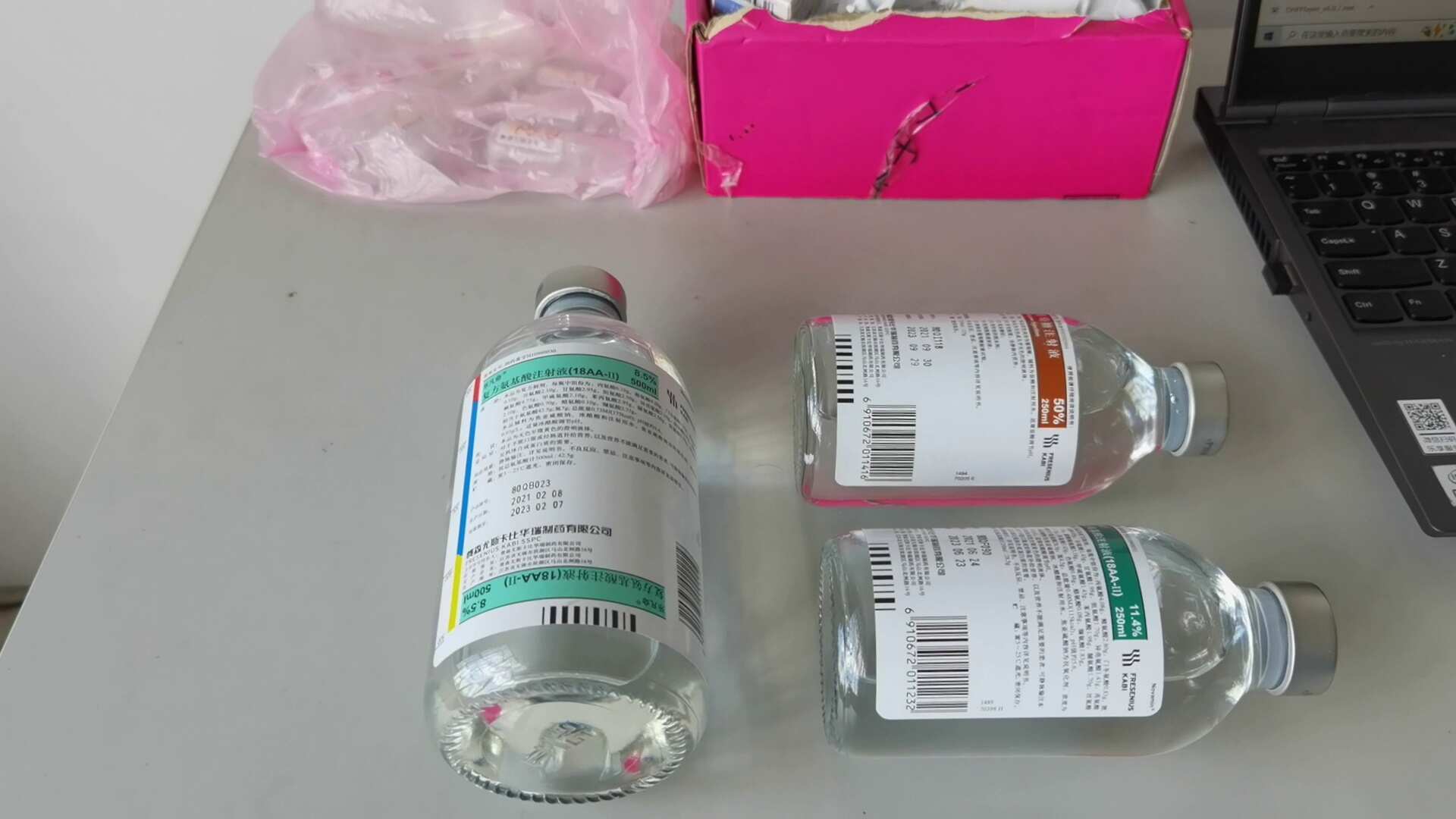

Supplement: S1 Dataset — (ZIP) [file pone.0298109.s001.zip › minimal data set/VOC2007/images/1131.jpg]

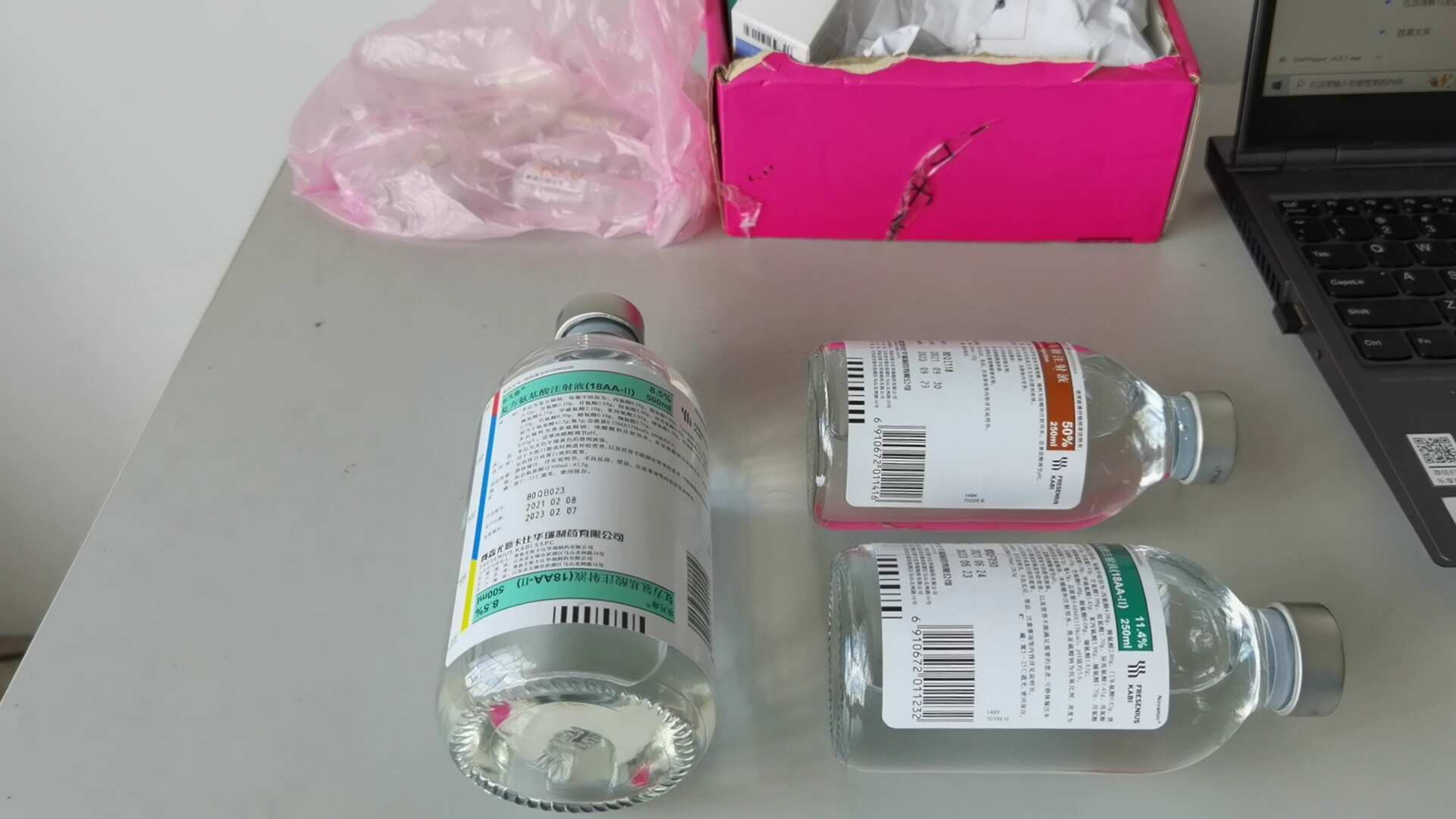

Supplement: S1 Dataset — (ZIP) [file pone.0298109.s001.zip › minimal data set/VOC2007/images/1132.jpg]

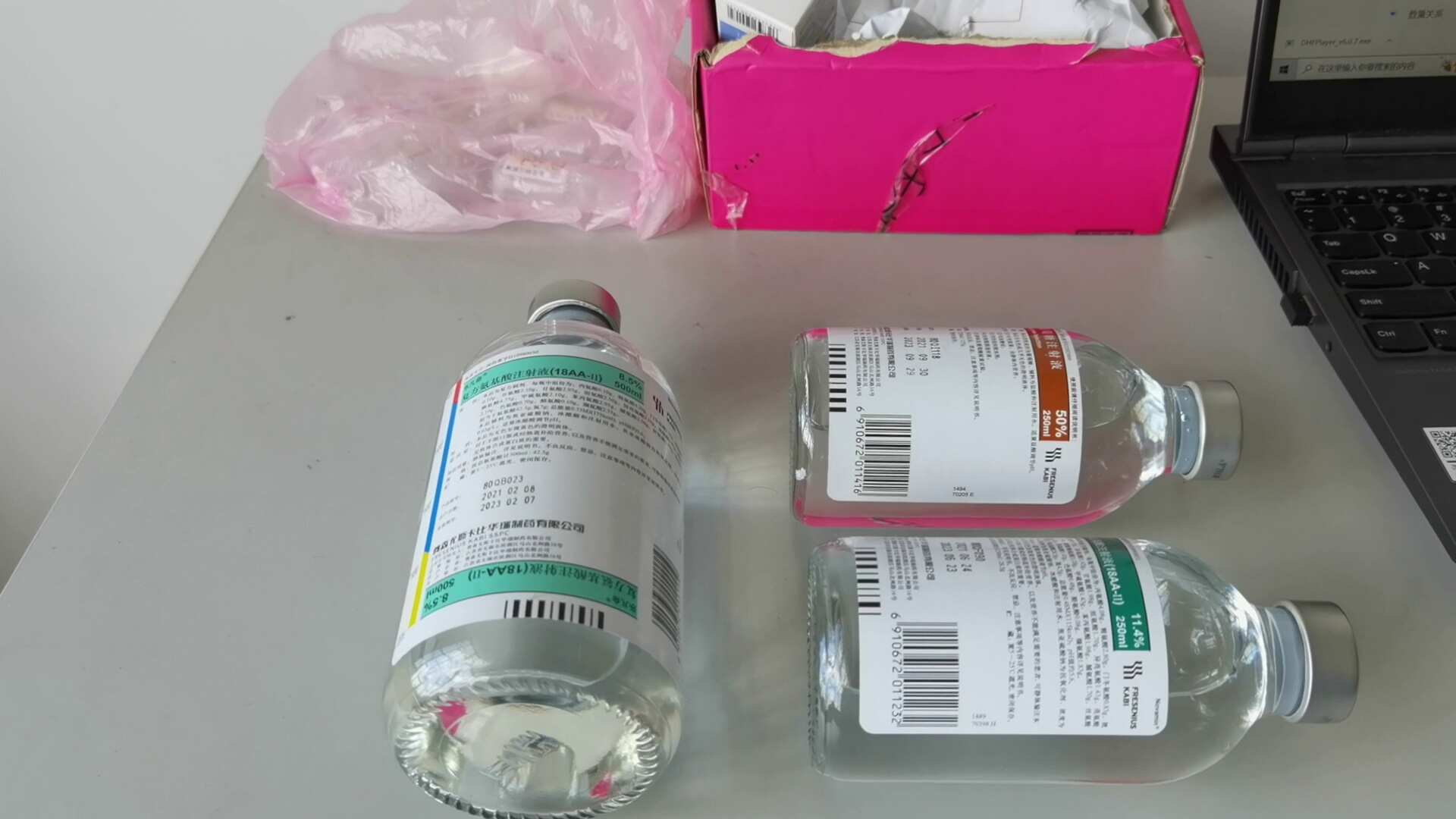

Supplement: S1 Dataset — (ZIP) [file pone.0298109.s001.zip › minimal data set/VOC2007/images/1133.jpg]

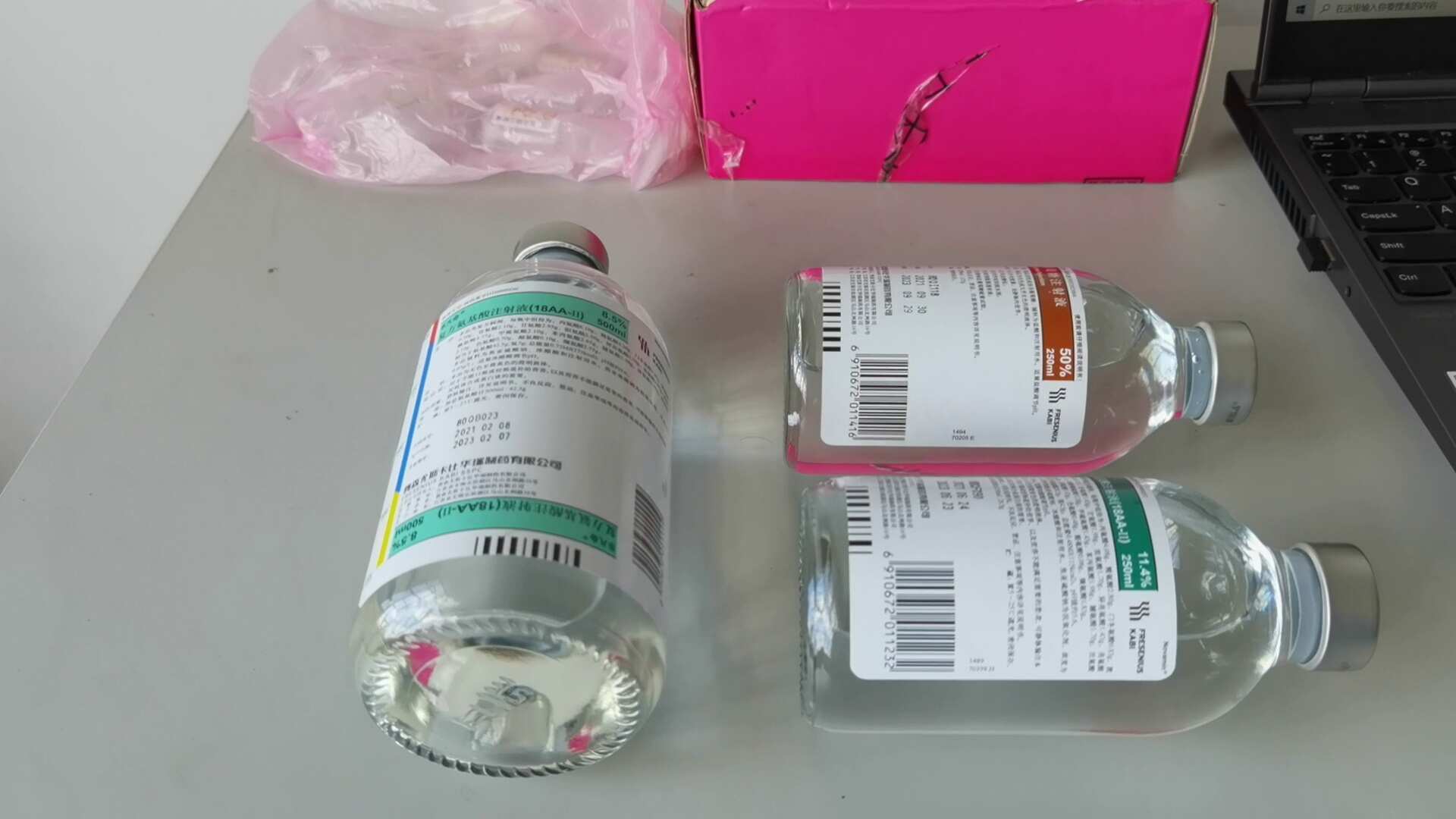

Supplement: S1 Dataset — (ZIP) [file pone.0298109.s001.zip › minimal data set/VOC2007/images/1134.jpg]

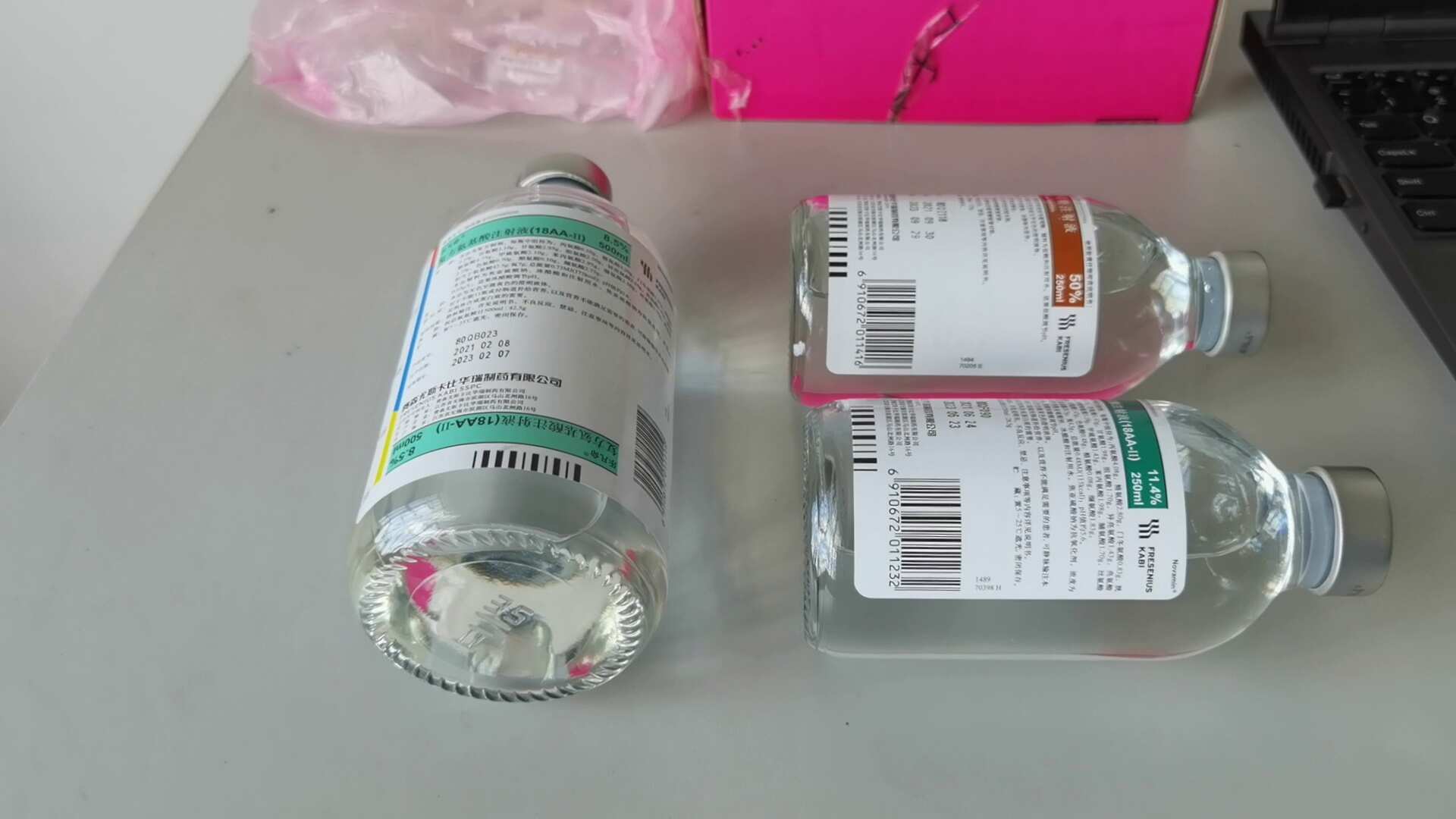

Supplement: S1 Dataset — (ZIP) [file pone.0298109.s001.zip › minimal data set/VOC2007/images/1135.jpg]

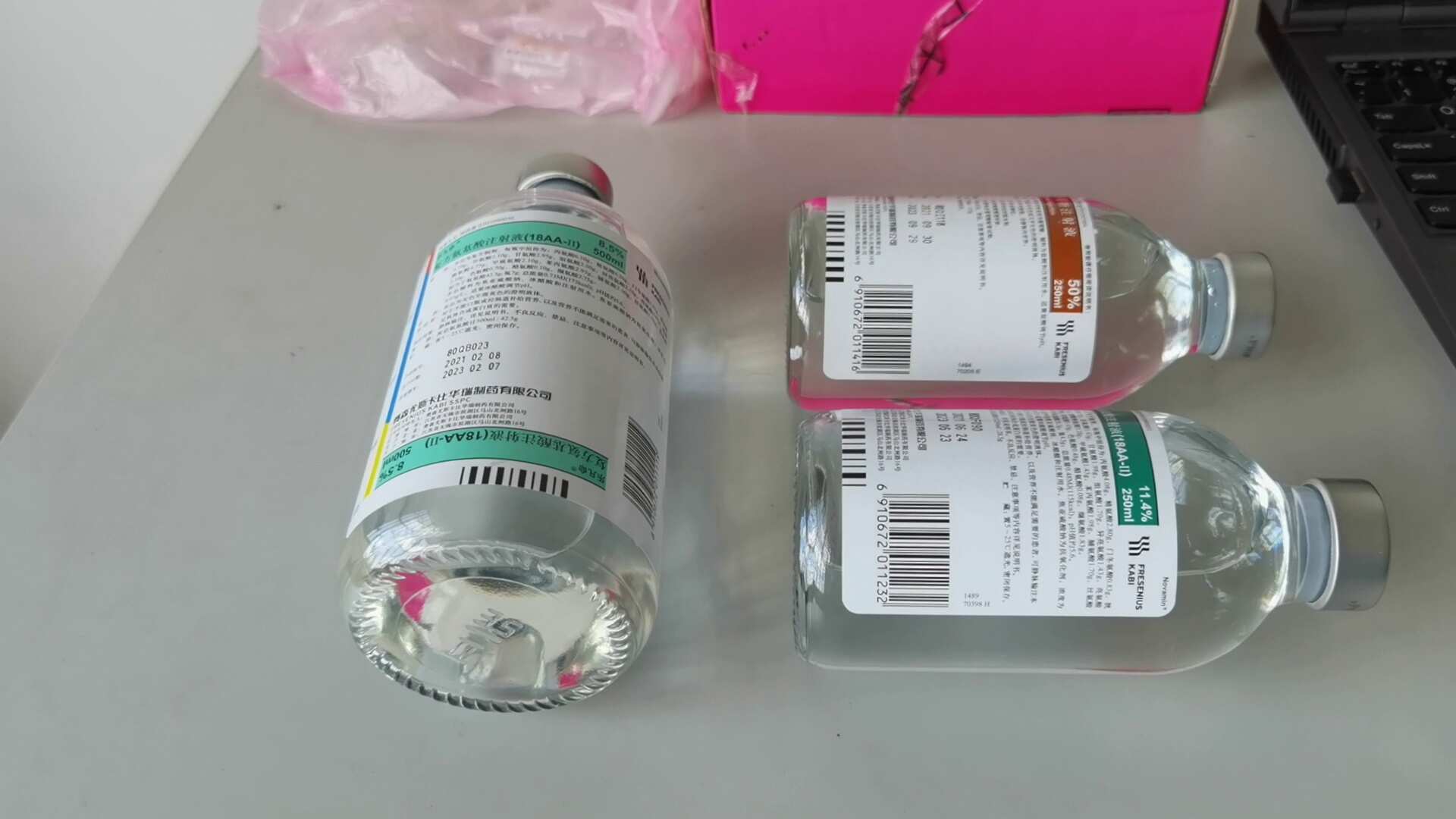

Supplement: S1 Dataset — (ZIP) [file pone.0298109.s001.zip › minimal data set/VOC2007/images/1136.jpg]

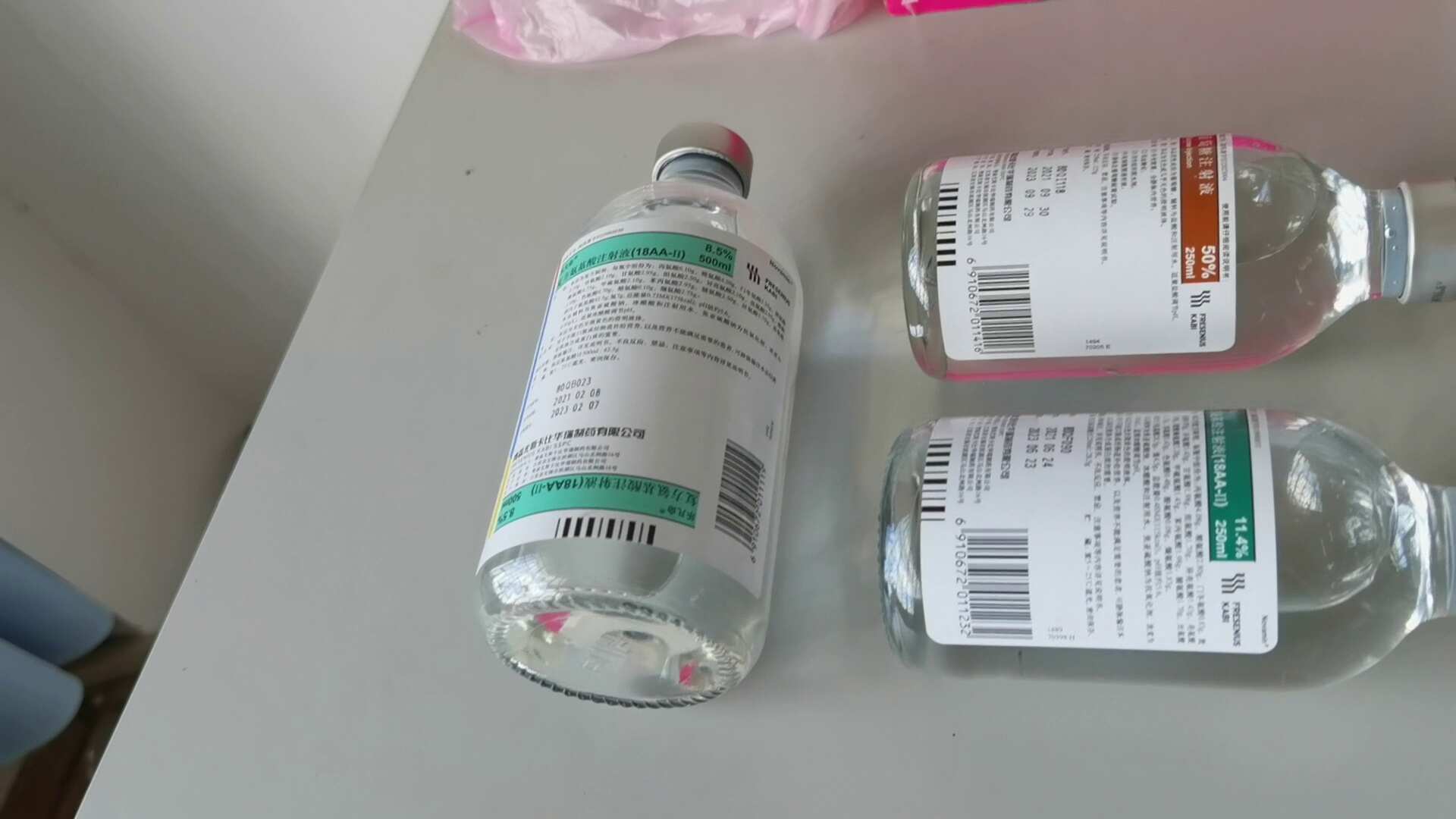

Supplement: S1 Dataset — (ZIP) [file pone.0298109.s001.zip › minimal data set/VOC2007/images/1137.jpg]

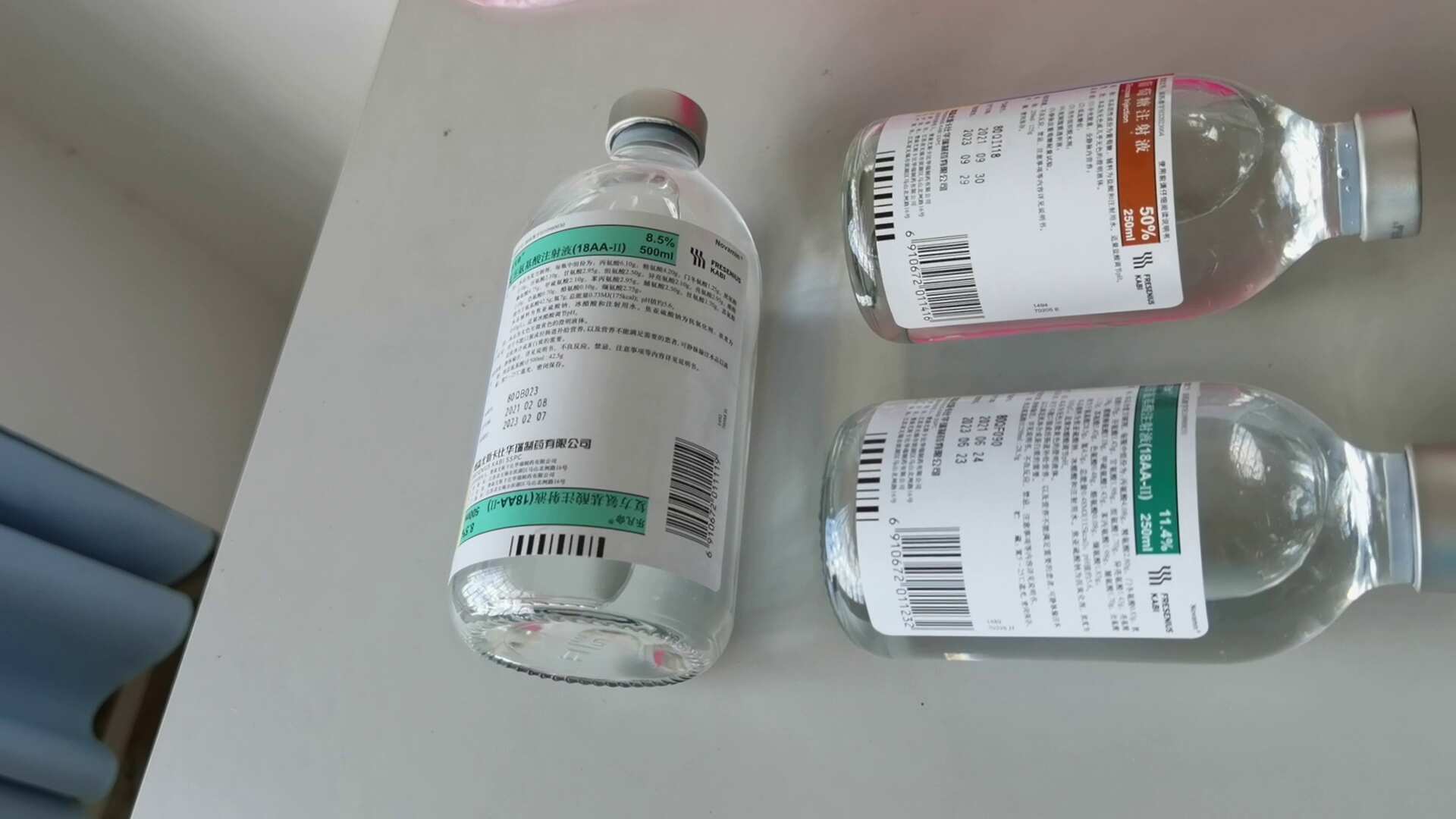

Supplement: S1 Dataset — (ZIP) [file pone.0298109.s001.zip › minimal data set/VOC2007/images/1138.jpg]

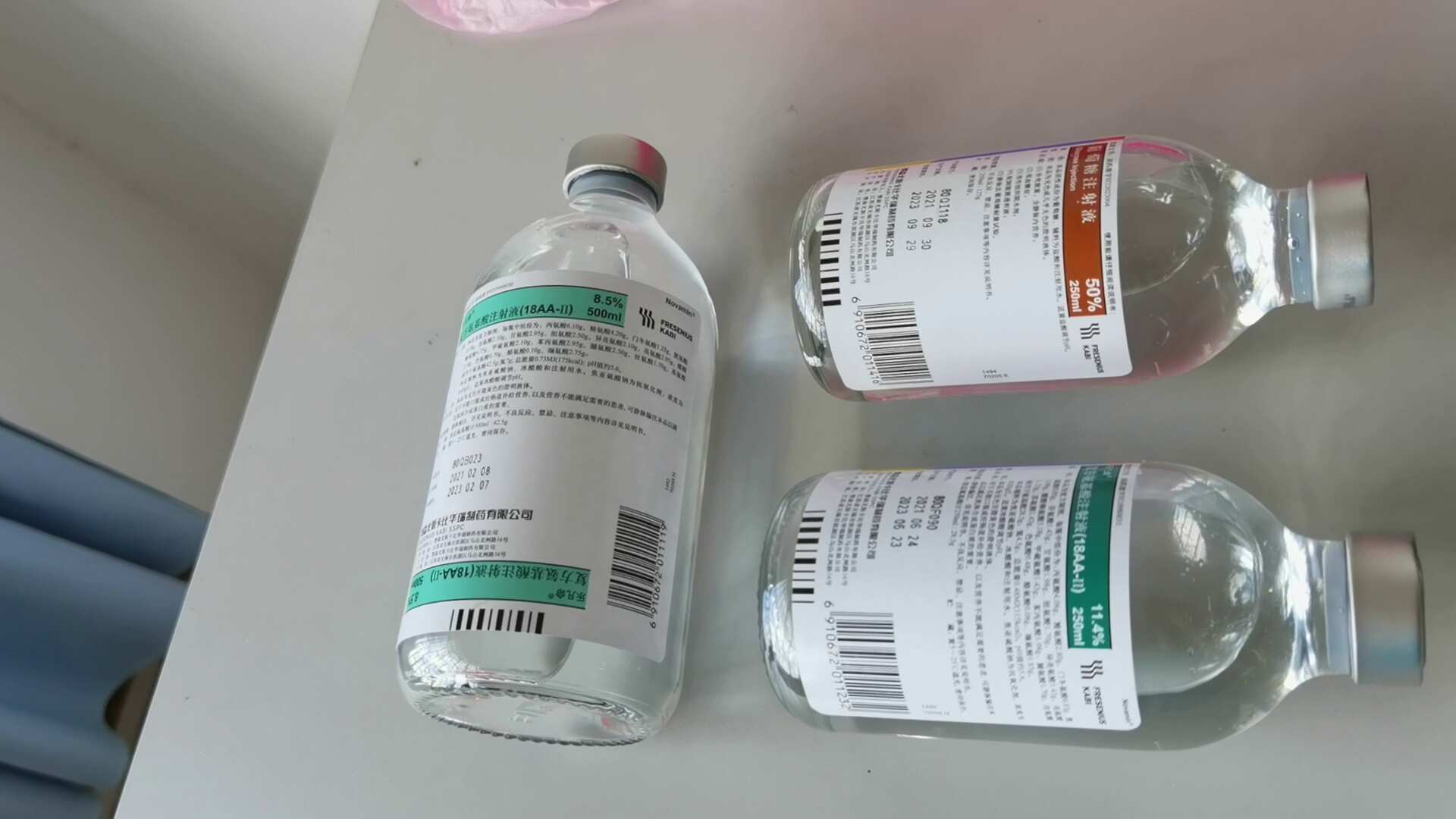

Supplement: S1 Dataset — (ZIP) [file pone.0298109.s001.zip › minimal data set/VOC2007/images/1139.jpg]

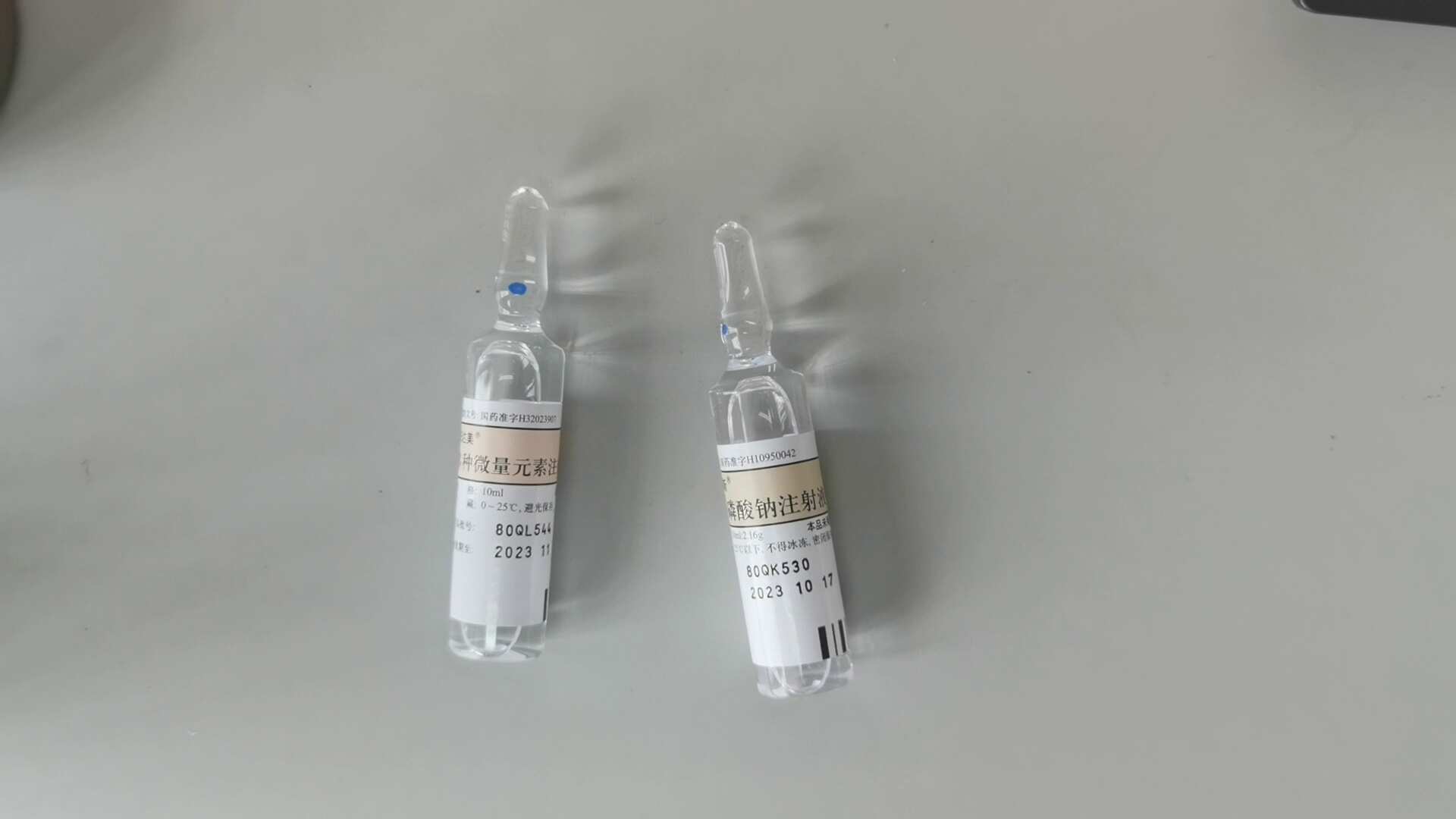

Supplement: S1 Dataset — (ZIP) [file pone.0298109.s001.zip › minimal data set/VOC2007/images/114.jpg]

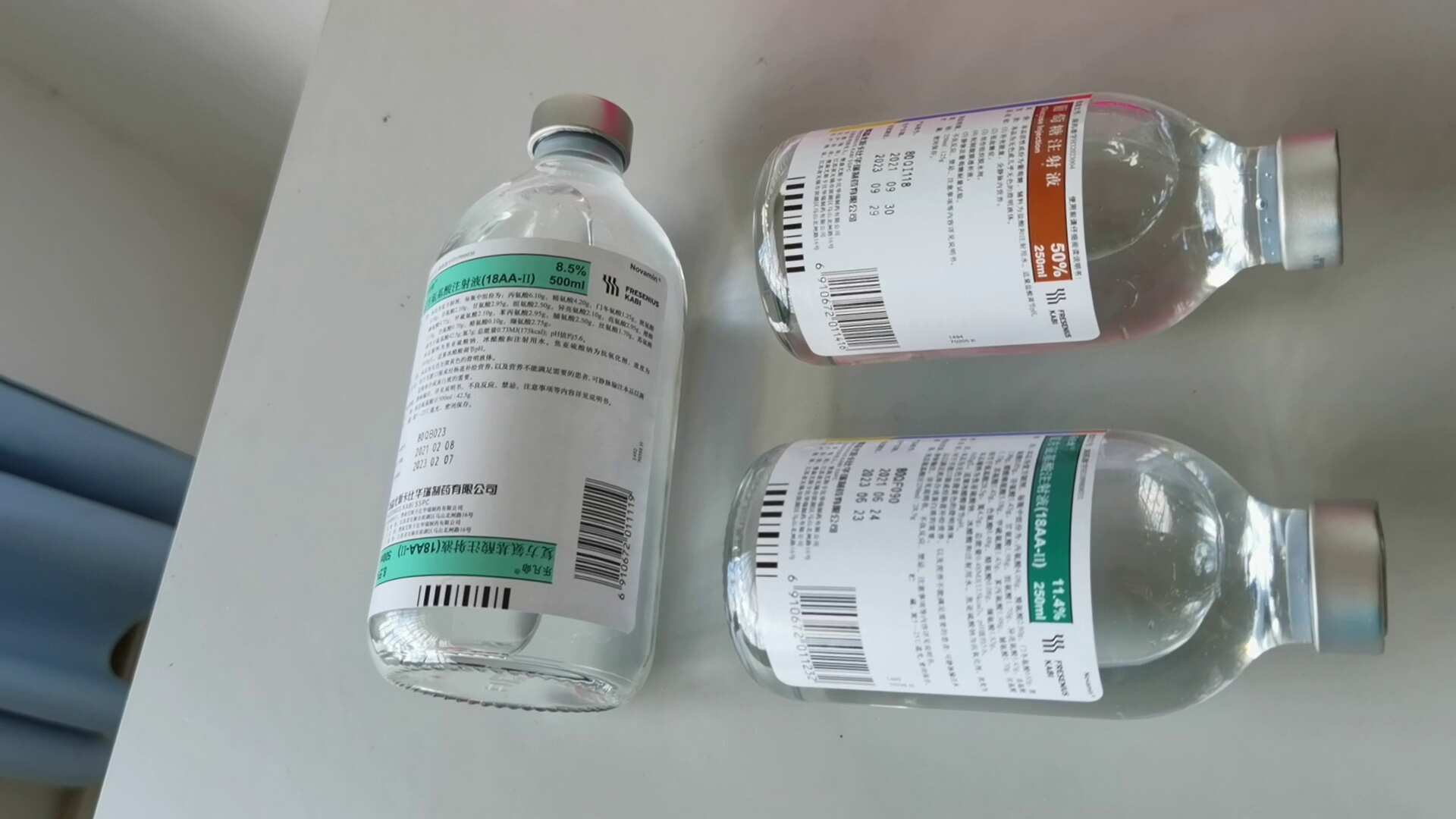

Supplement: S1 Dataset — (ZIP) [file pone.0298109.s001.zip › minimal data set/VOC2007/images/1140.jpg]

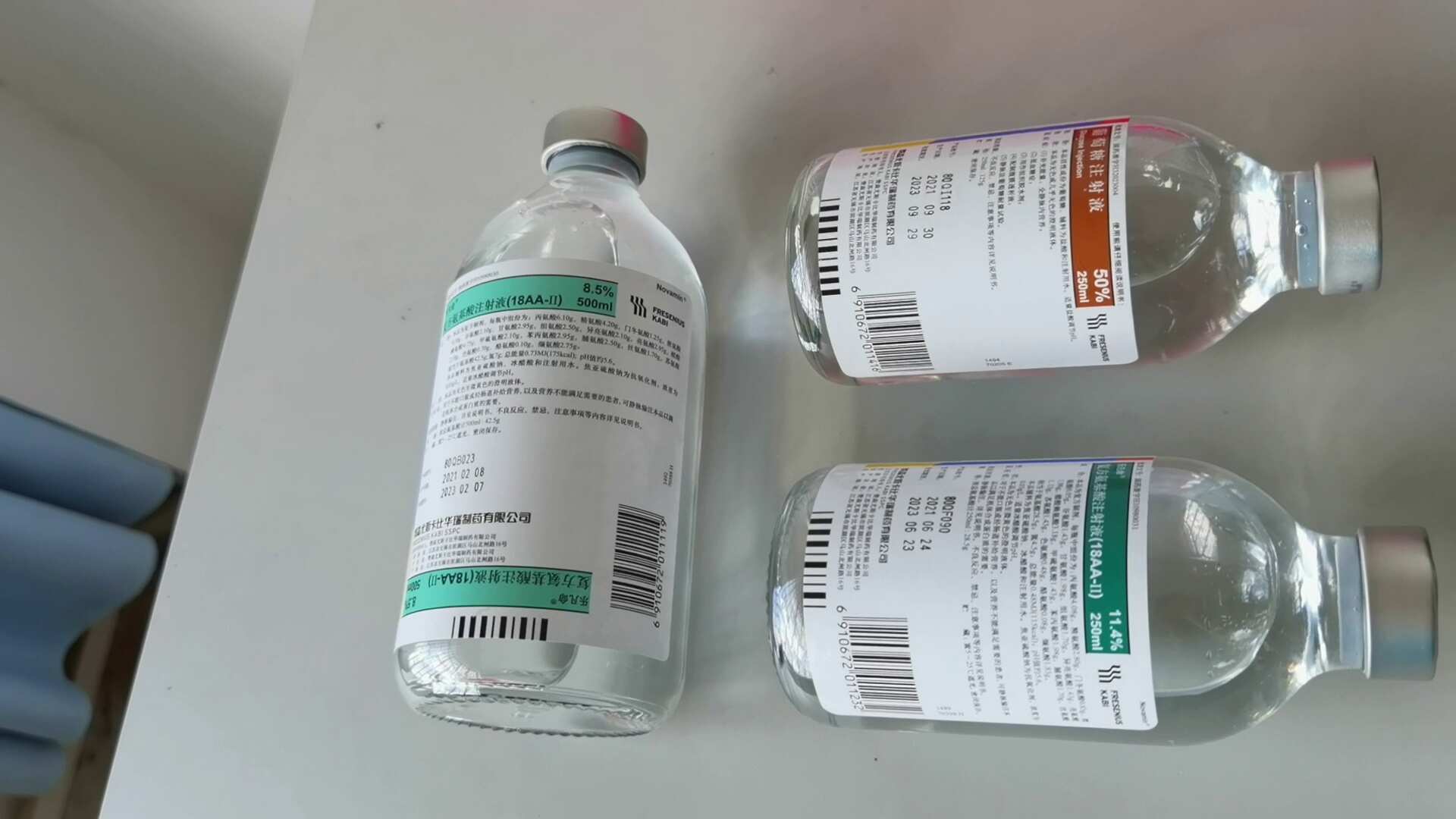

Supplement: S1 Dataset — (ZIP) [file pone.0298109.s001.zip › minimal data set/VOC2007/images/1141.jpg]

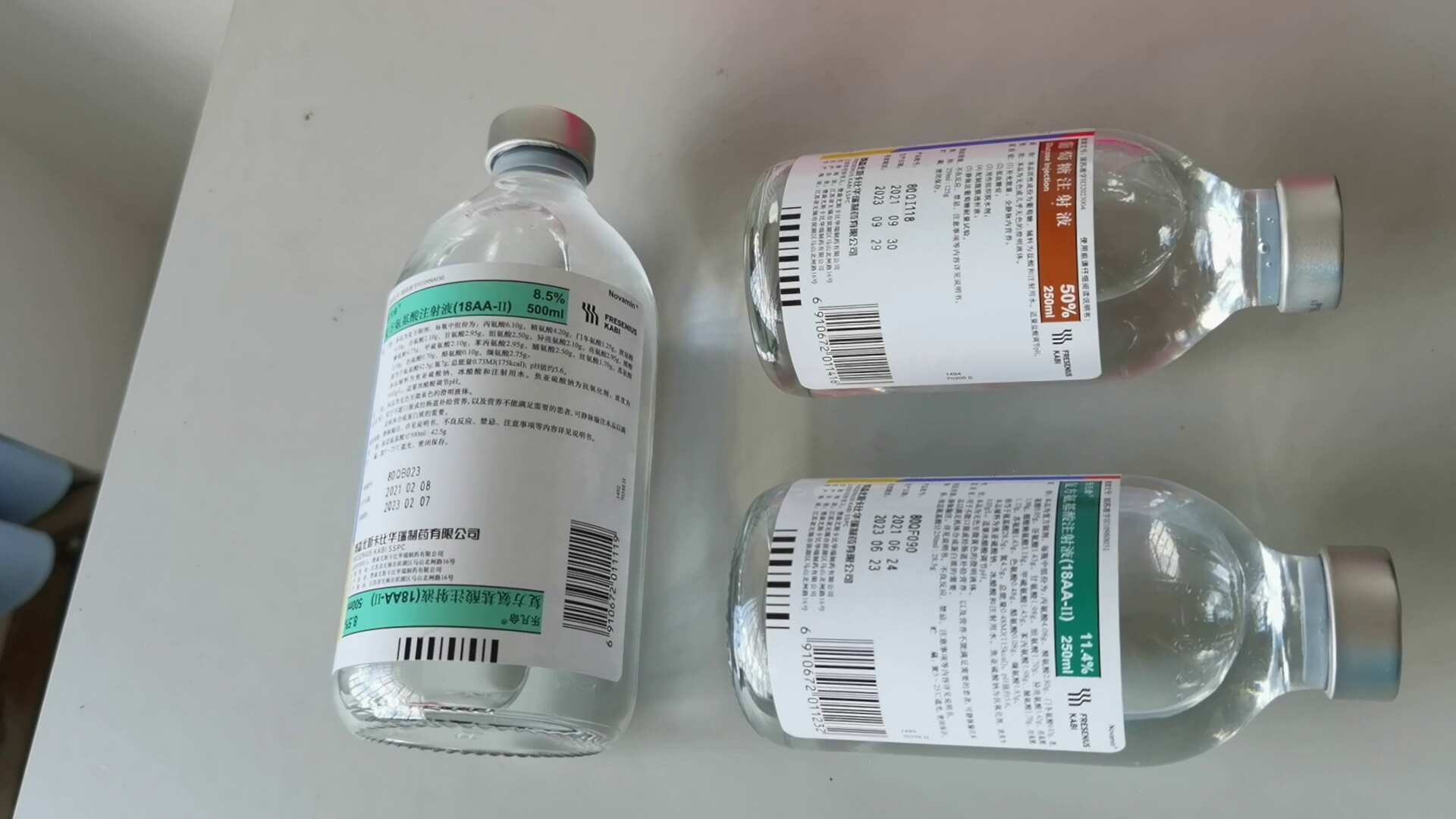

Supplement: S1 Dataset — (ZIP) [file pone.0298109.s001.zip › minimal data set/VOC2007/images/1142.jpg]

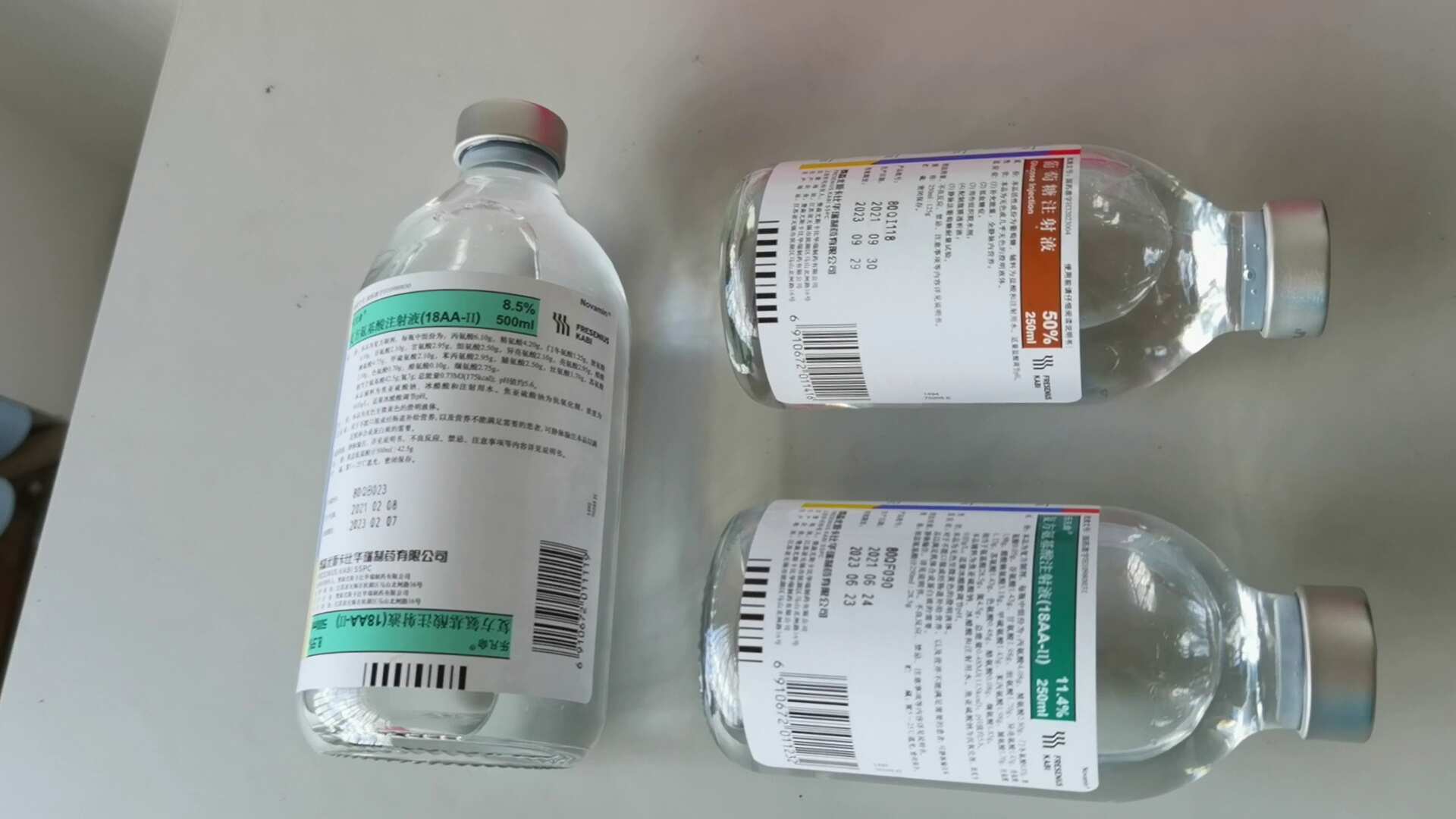

Supplement: S1 Dataset — (ZIP) [file pone.0298109.s001.zip › minimal data set/VOC2007/images/1143.jpg]

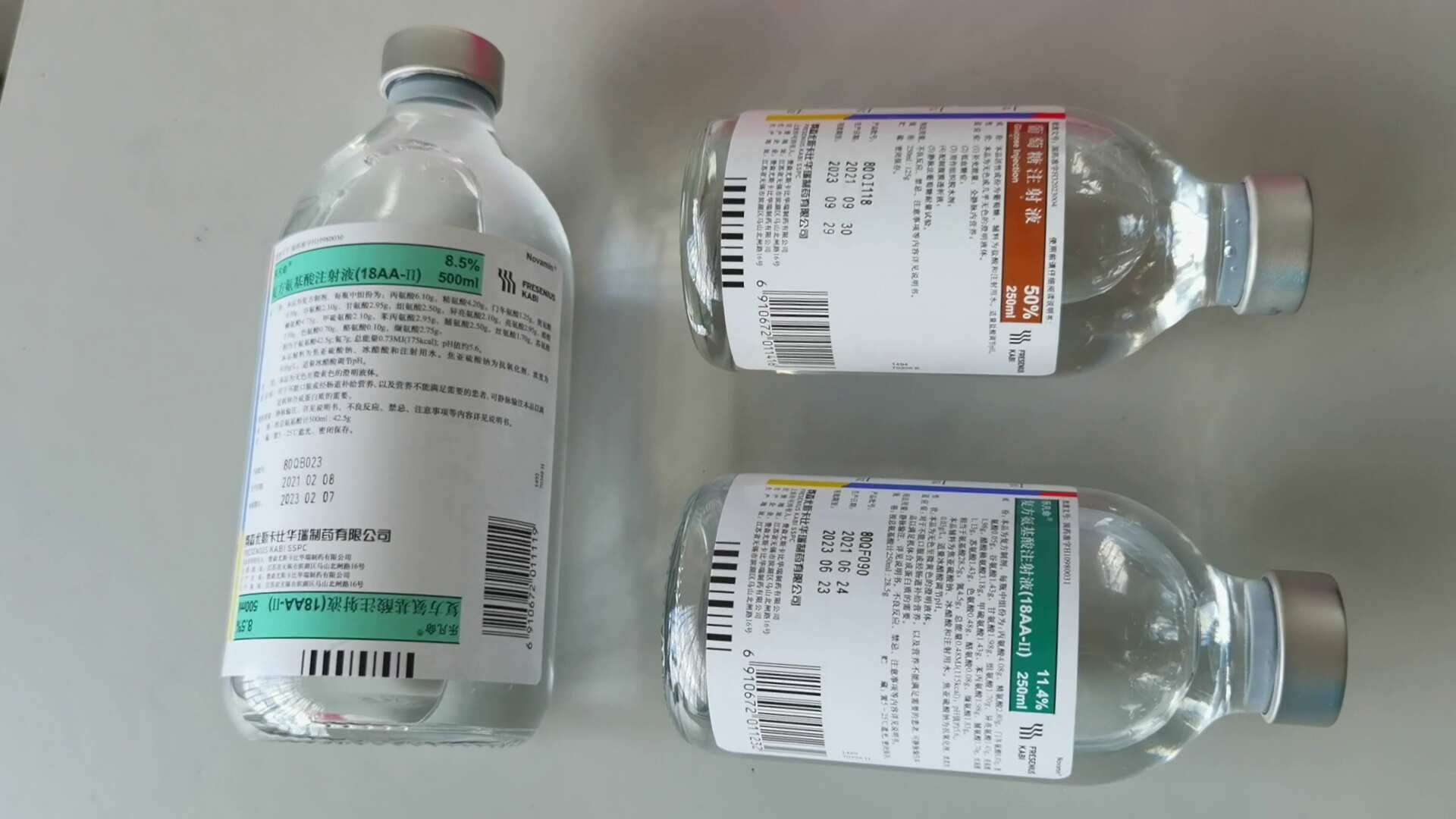

Supplement: S1 Dataset — (ZIP) [file pone.0298109.s001.zip › minimal data set/VOC2007/images/1144.jpg]

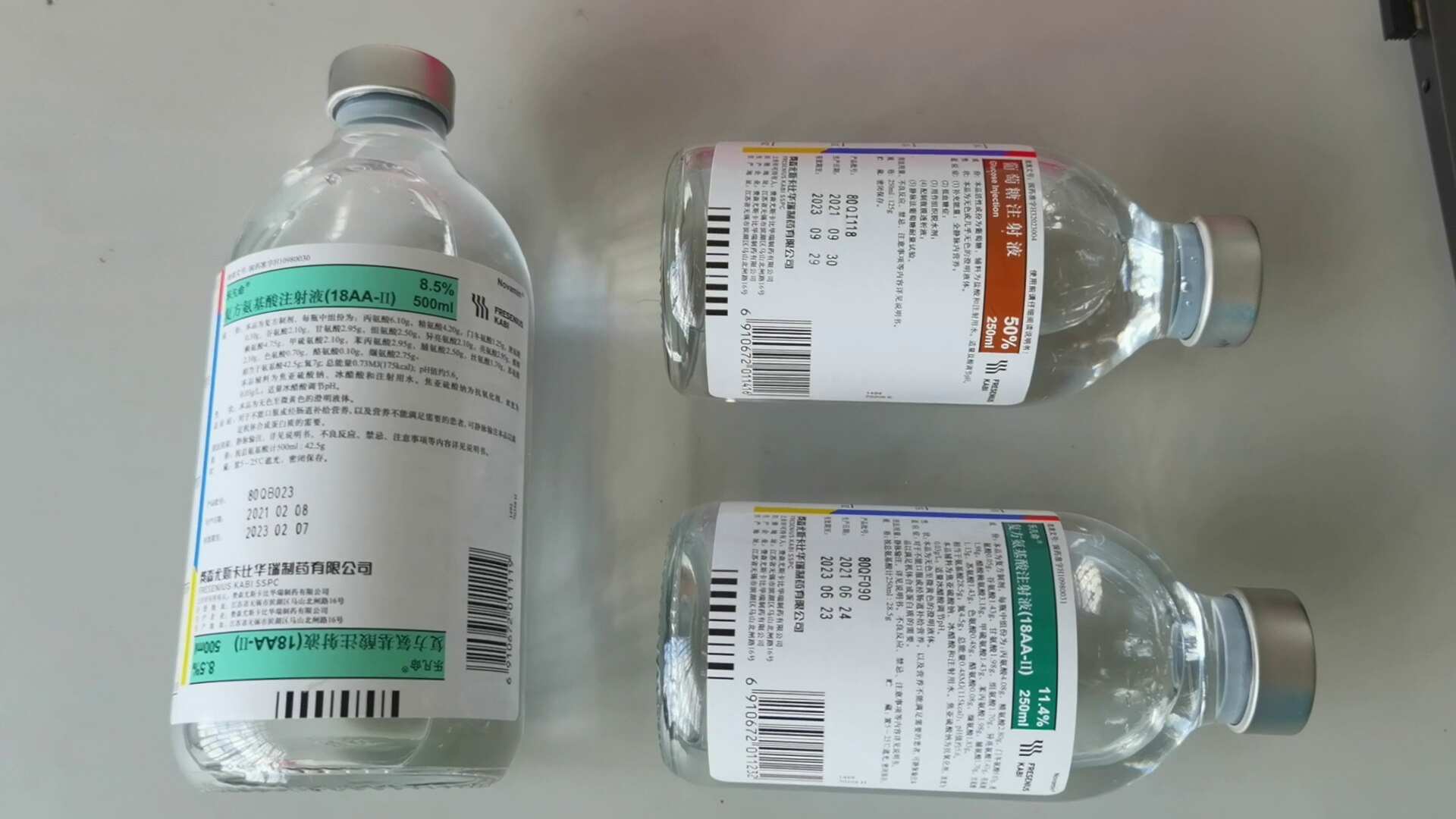

Supplement: S1 Dataset — (ZIP) [file pone.0298109.s001.zip › minimal data set/VOC2007/images/1145.jpg]

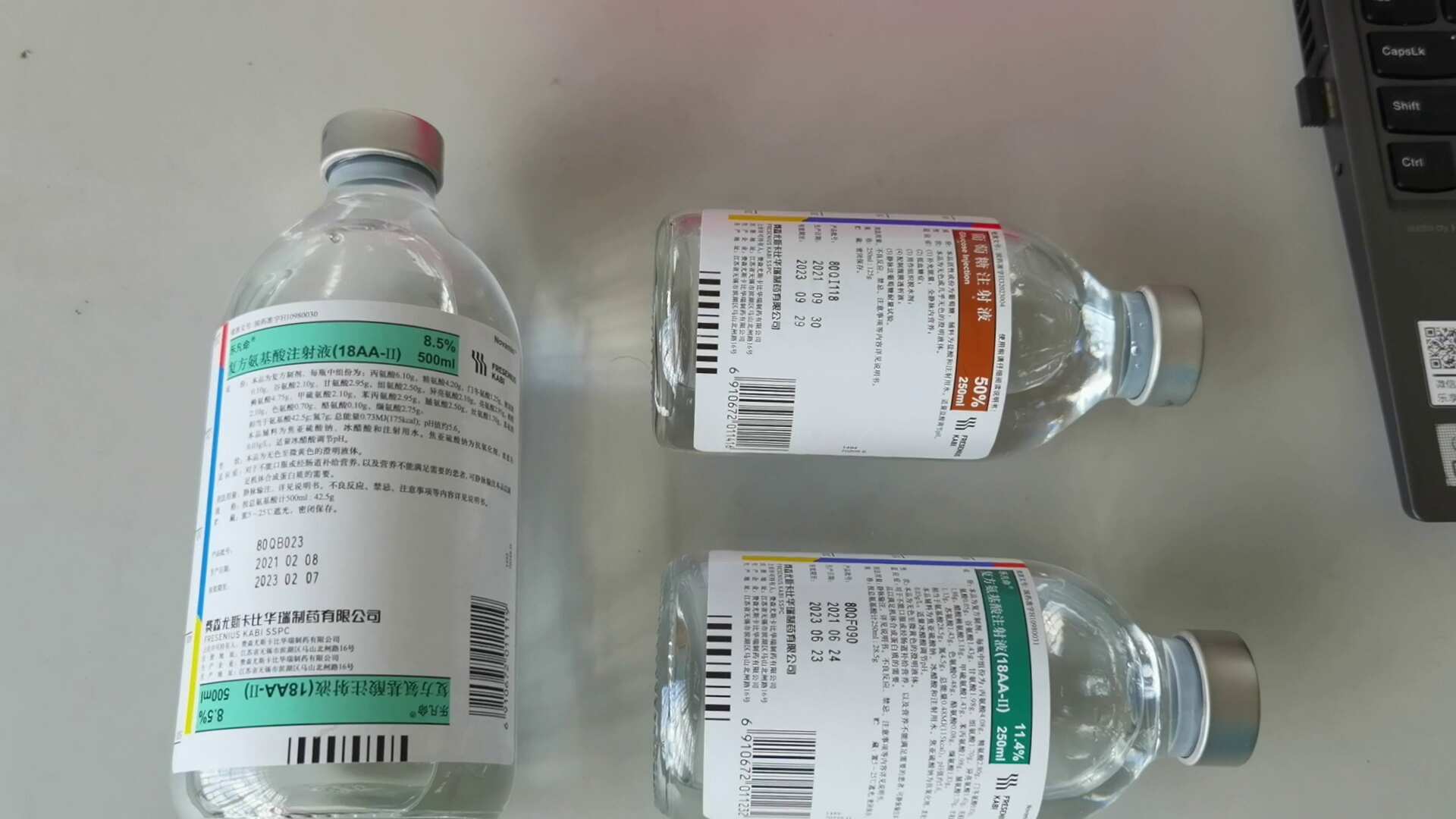

Supplement: S1 Dataset — (ZIP) [file pone.0298109.s001.zip › minimal data set/VOC2007/images/1146.jpg]

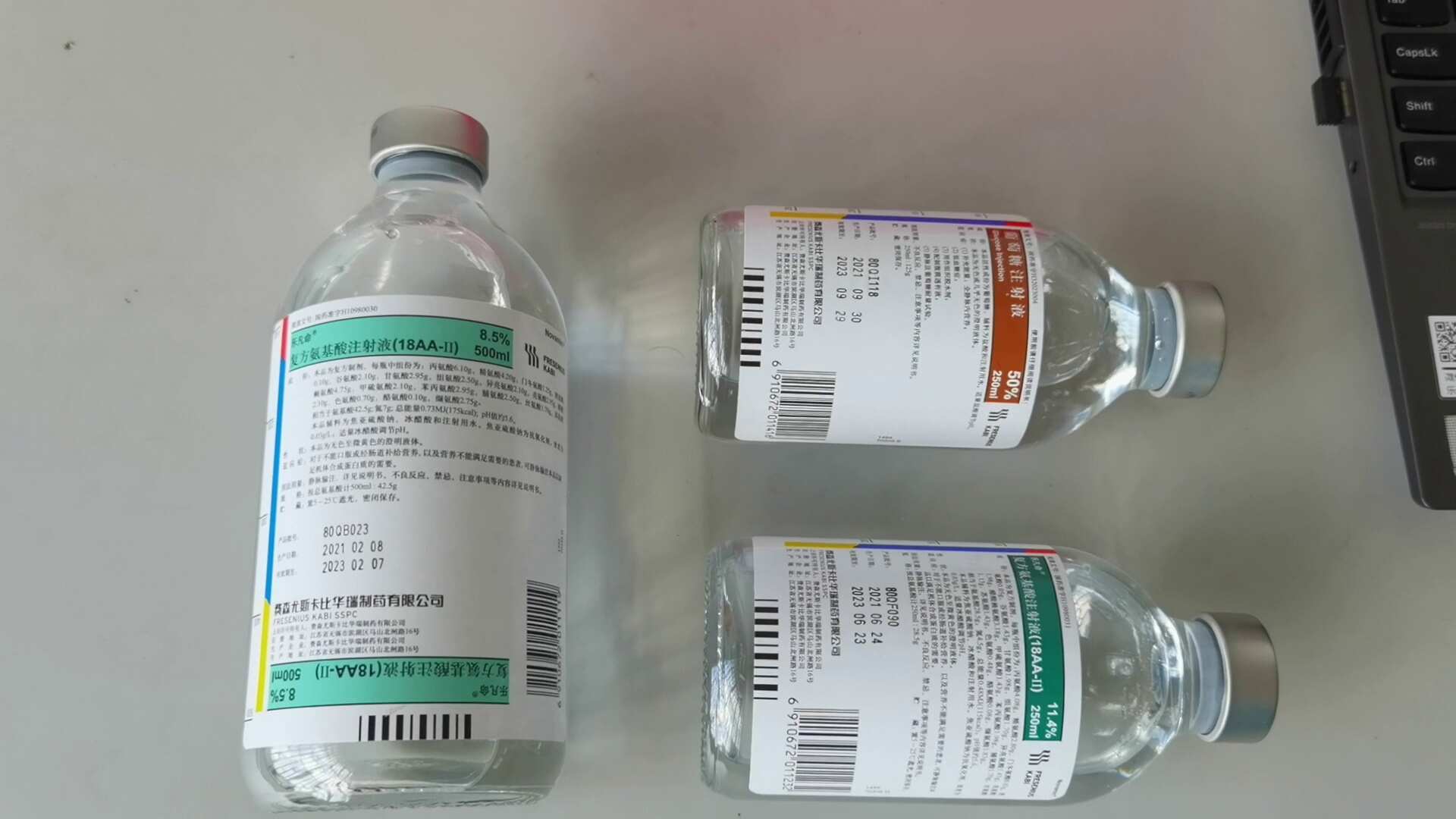

Supplement: S1 Dataset — (ZIP) [file pone.0298109.s001.zip › minimal data set/VOC2007/images/1147.jpg]

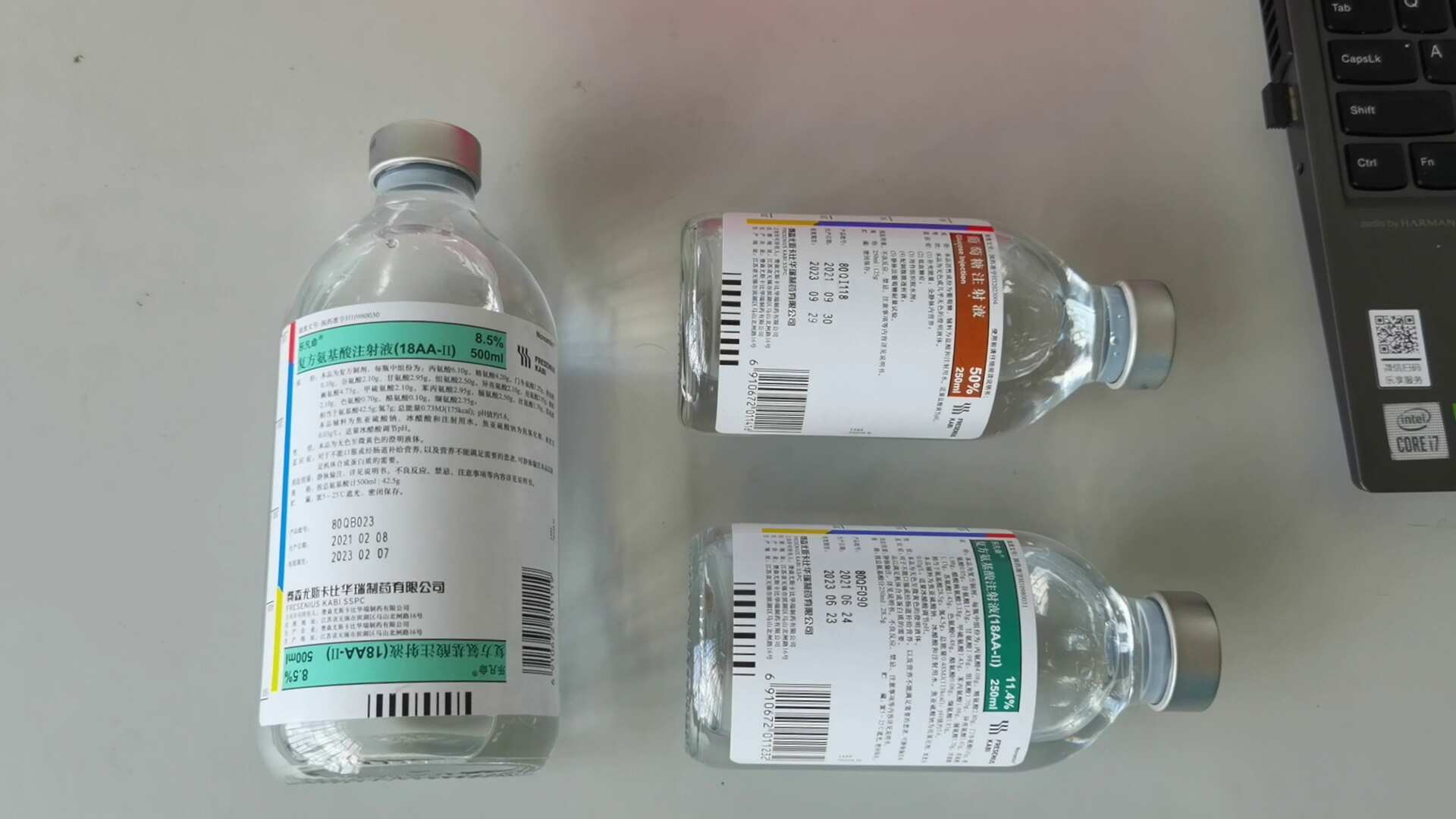

Supplement: S1 Dataset — (ZIP) [file pone.0298109.s001.zip › minimal data set/VOC2007/images/1148.jpg]

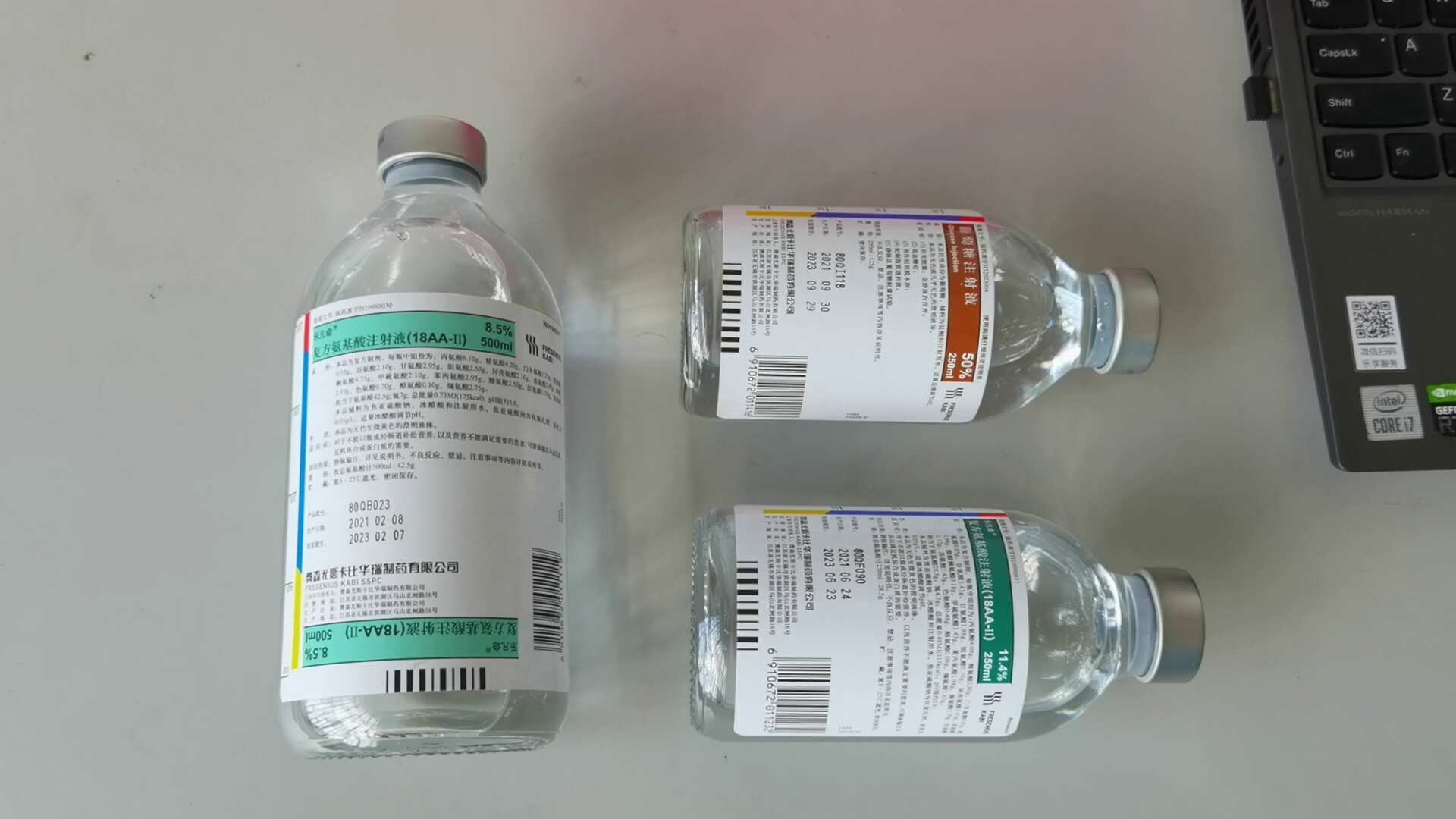

Supplement: S1 Dataset — (ZIP) [file pone.0298109.s001.zip › minimal data set/VOC2007/images/1149.jpg]

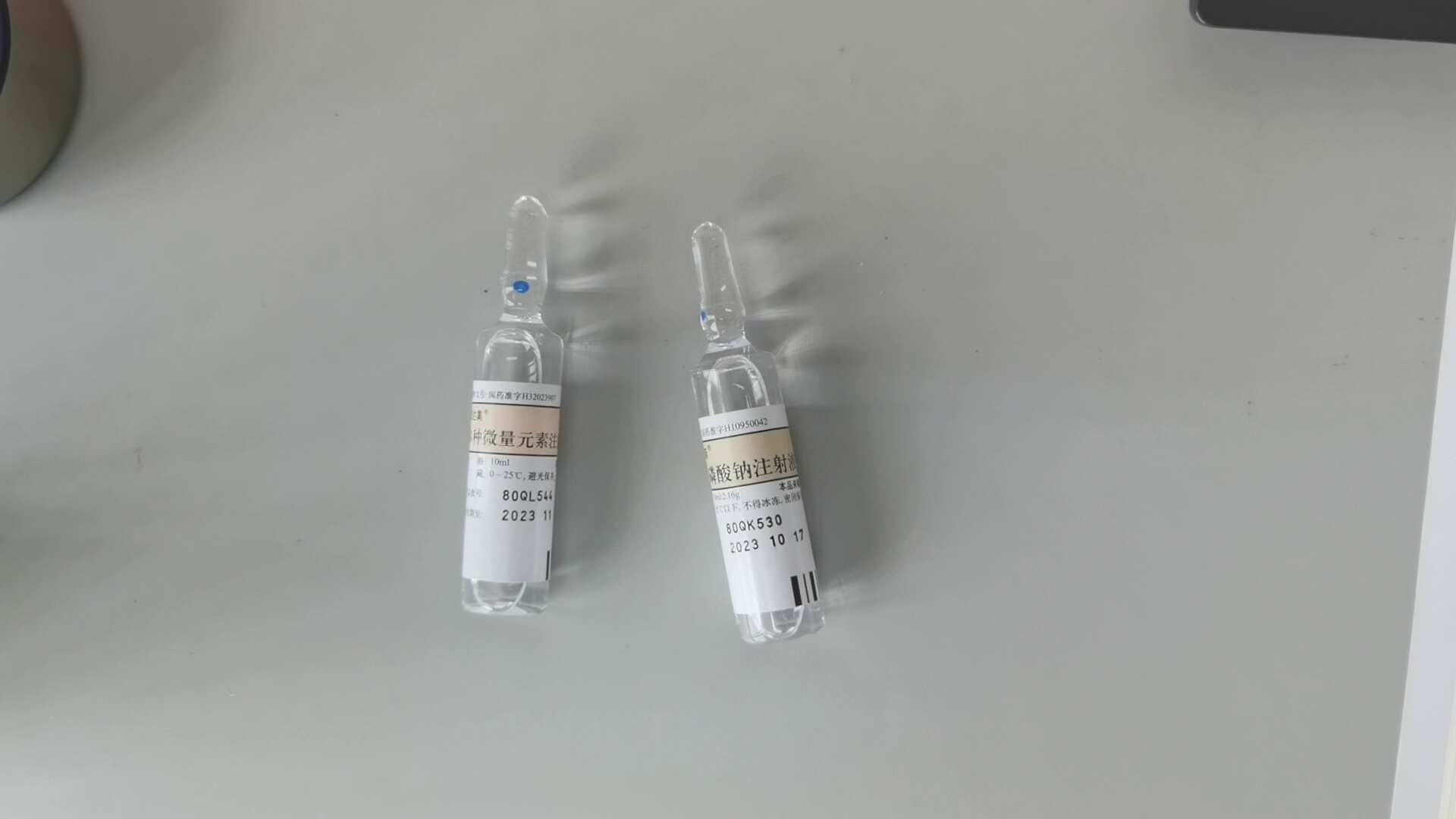

Supplement: S1 Dataset — (ZIP) [file pone.0298109.s001.zip › minimal data set/VOC2007/images/115.jpg]

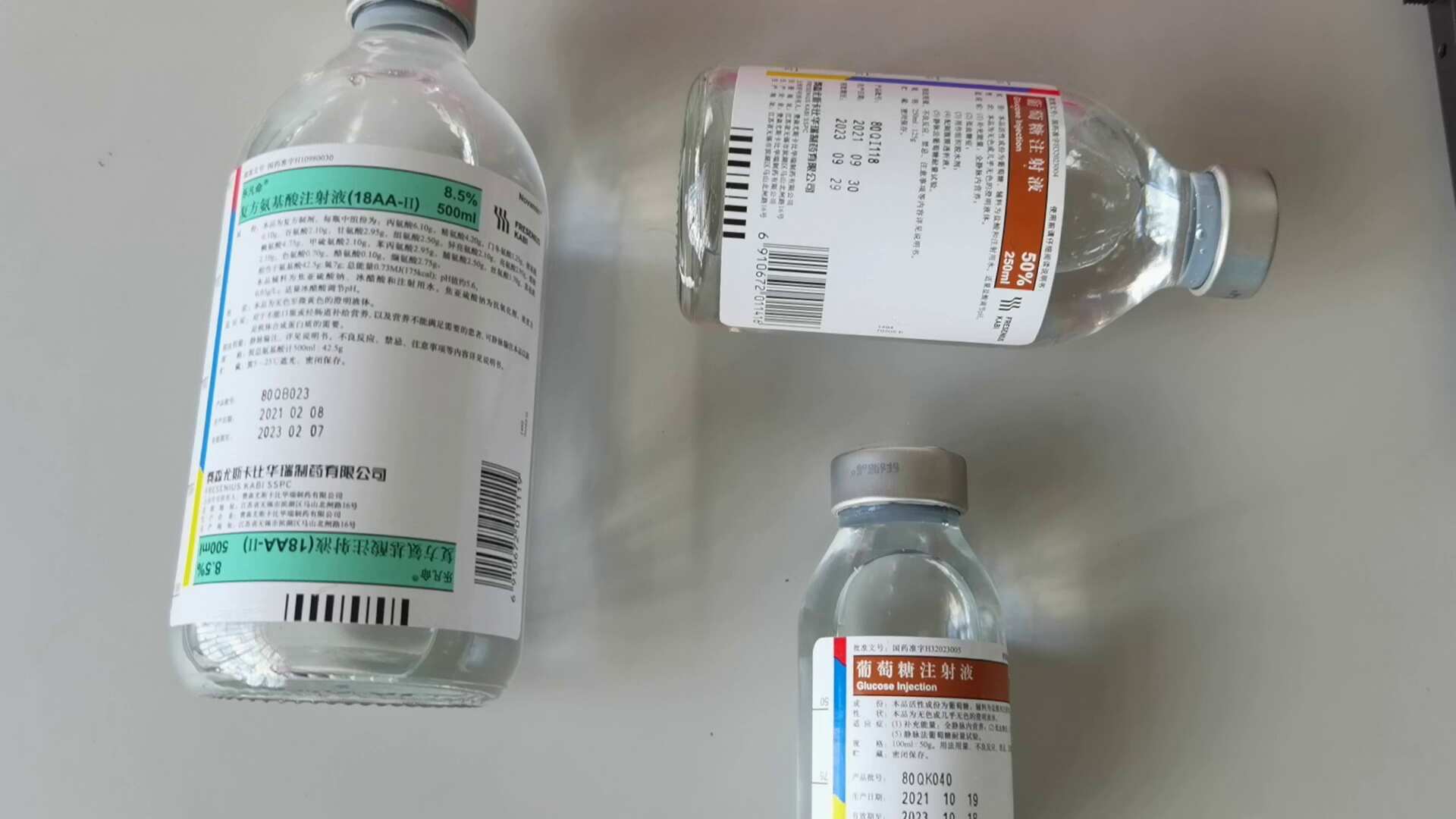

Supplement: S1 Dataset — (ZIP) [file pone.0298109.s001.zip › minimal data set/VOC2007/images/1150.jpg]

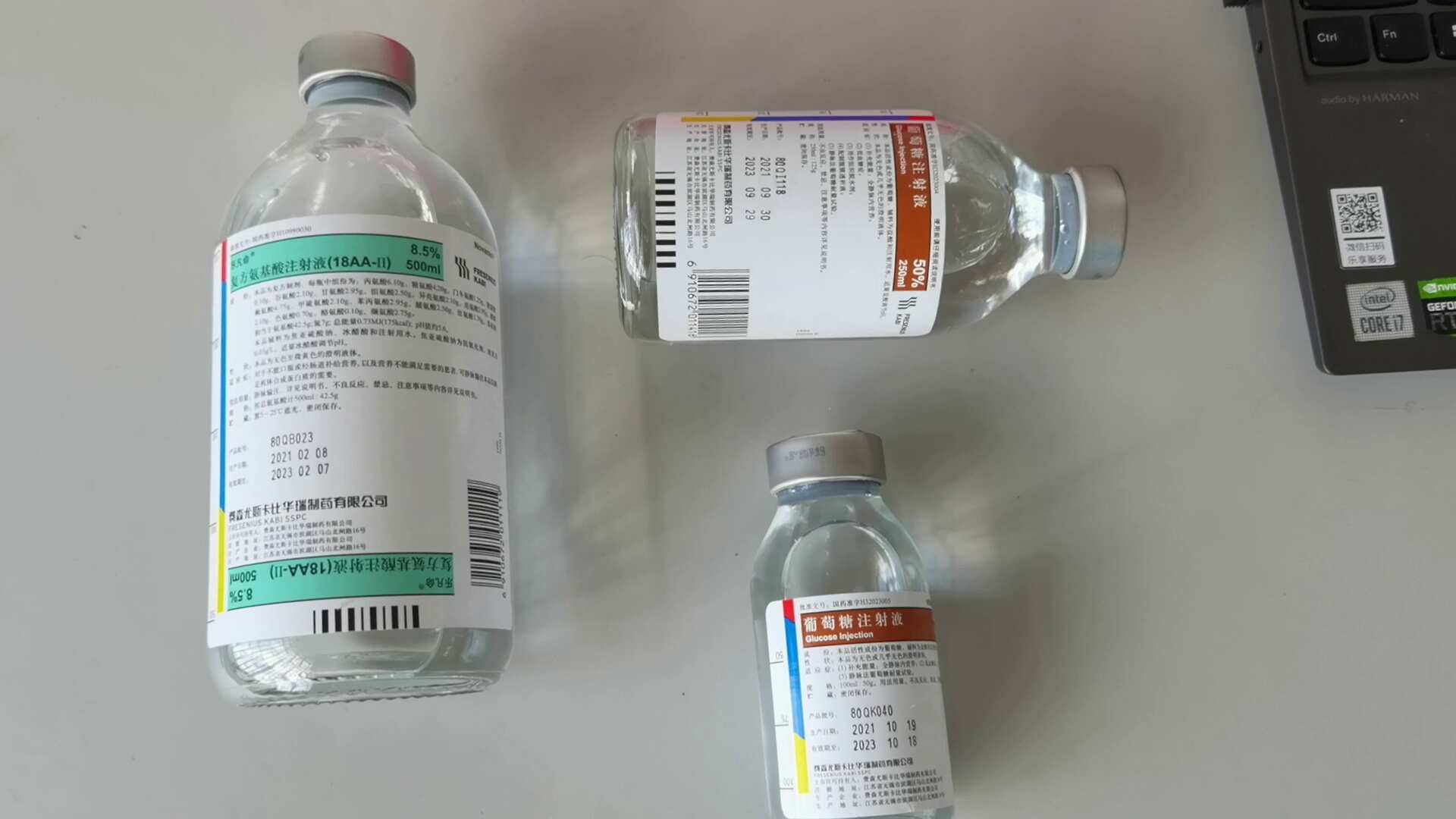

Supplement: S1 Dataset — (ZIP) [file pone.0298109.s001.zip › minimal data set/VOC2007/images/1151.jpg]

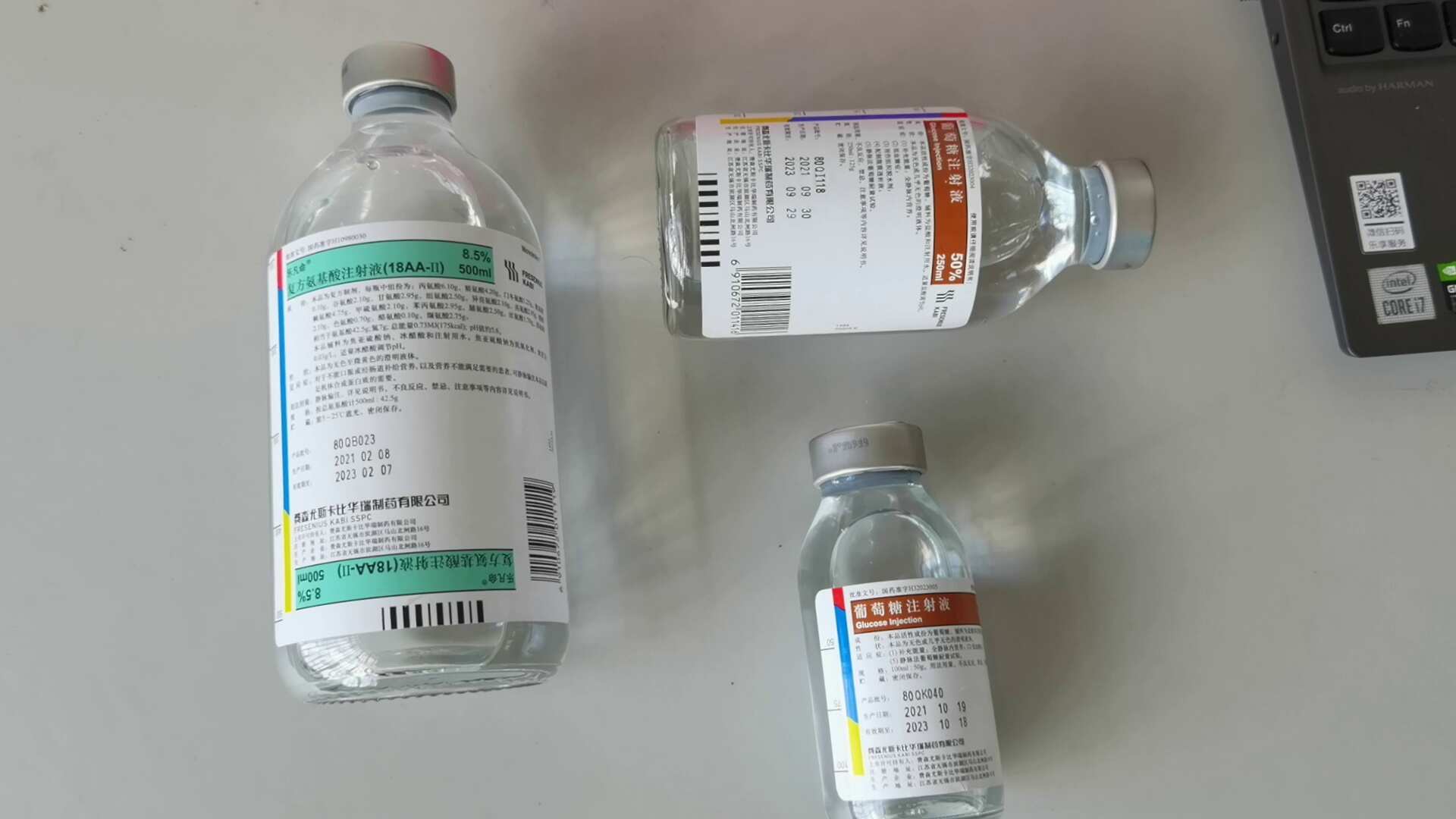

Supplement: S1 Dataset — (ZIP) [file pone.0298109.s001.zip › minimal data set/VOC2007/images/1152.jpg]

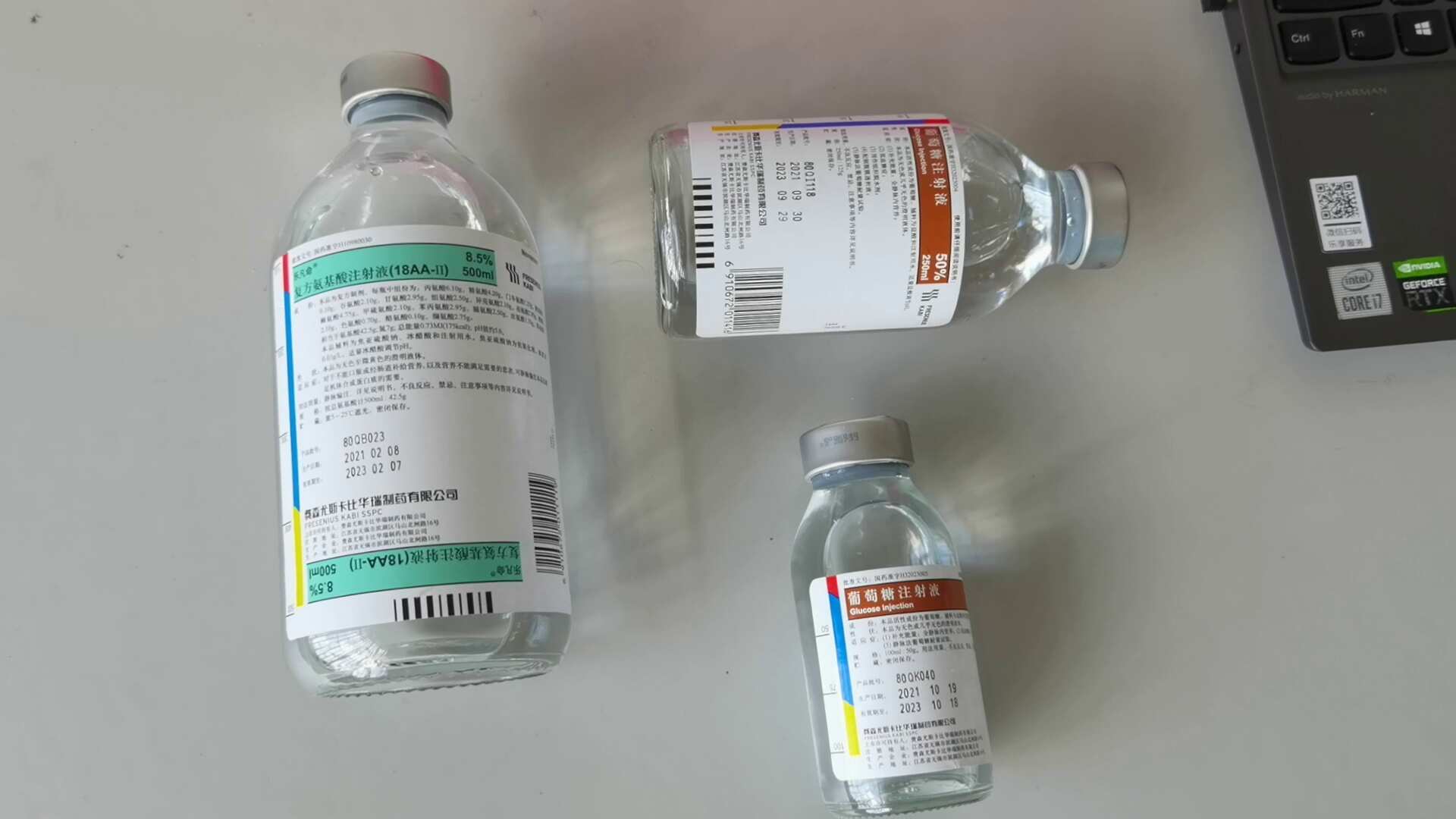

Supplement: S1 Dataset — (ZIP) [file pone.0298109.s001.zip › minimal data set/VOC2007/images/1153.jpg]

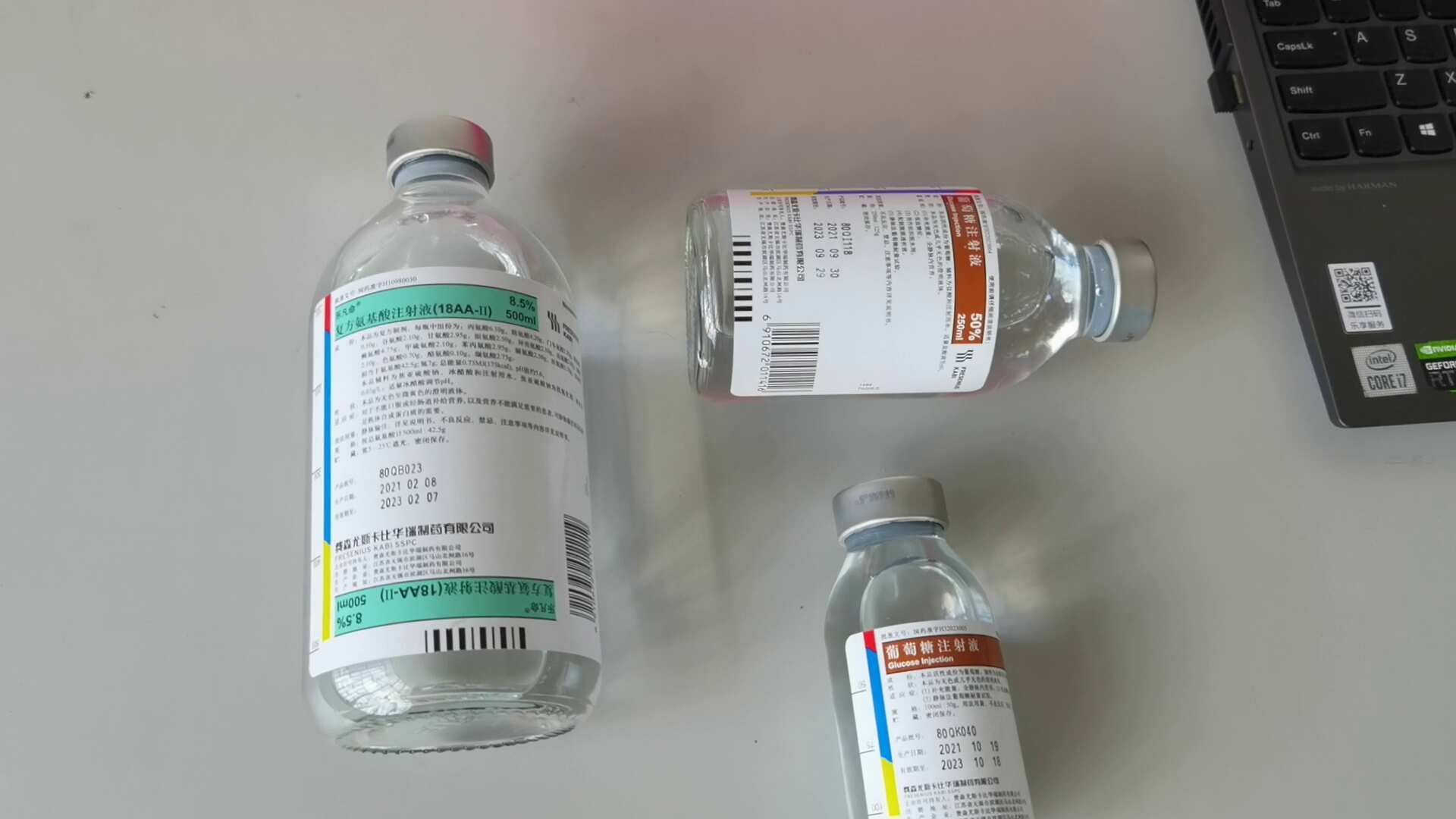

Supplement: S1 Dataset — (ZIP) [file pone.0298109.s001.zip › minimal data set/VOC2007/images/1154.jpg]

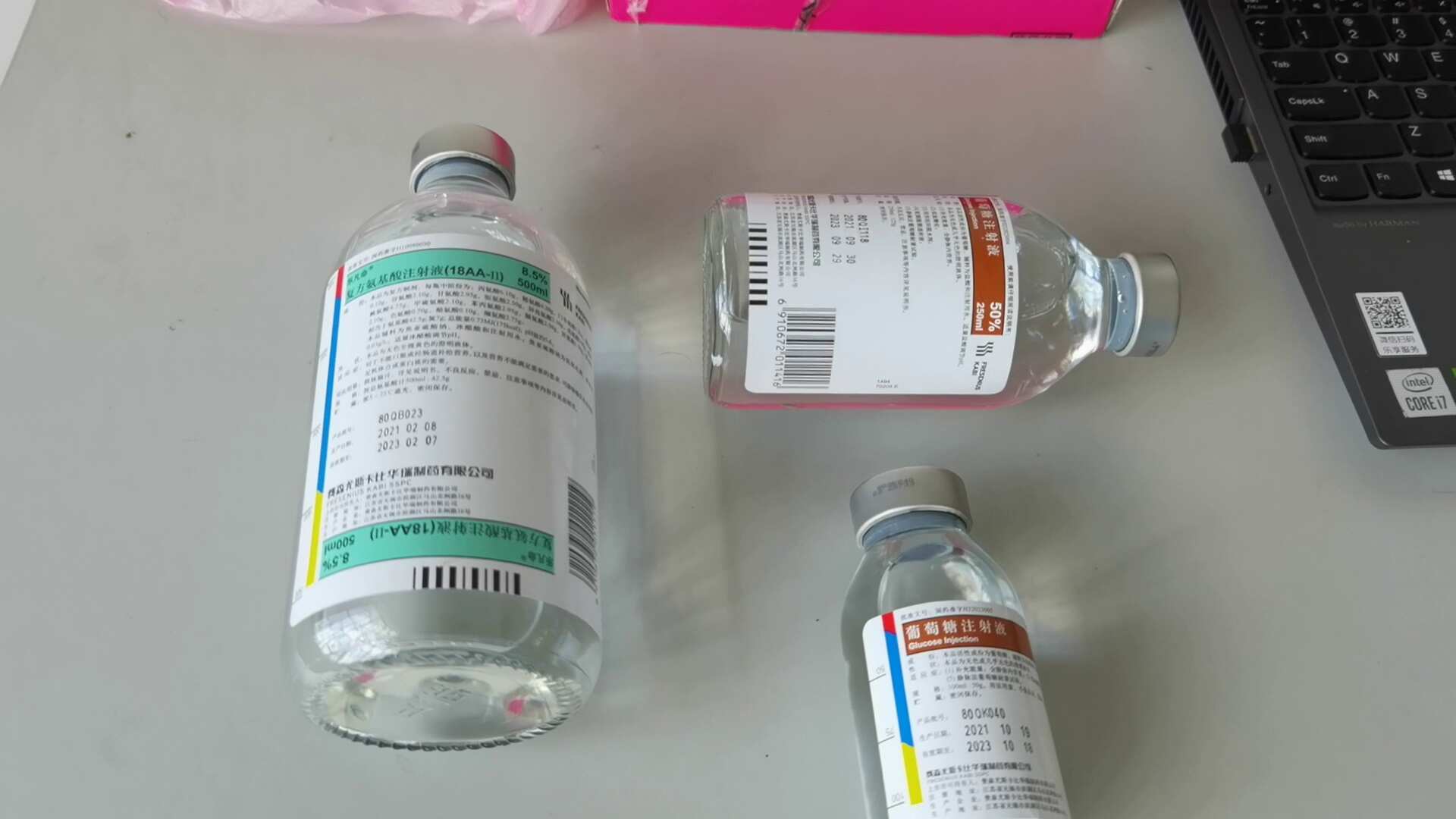

Supplement: S1 Dataset — (ZIP) [file pone.0298109.s001.zip › minimal data set/VOC2007/images/1155.jpg]

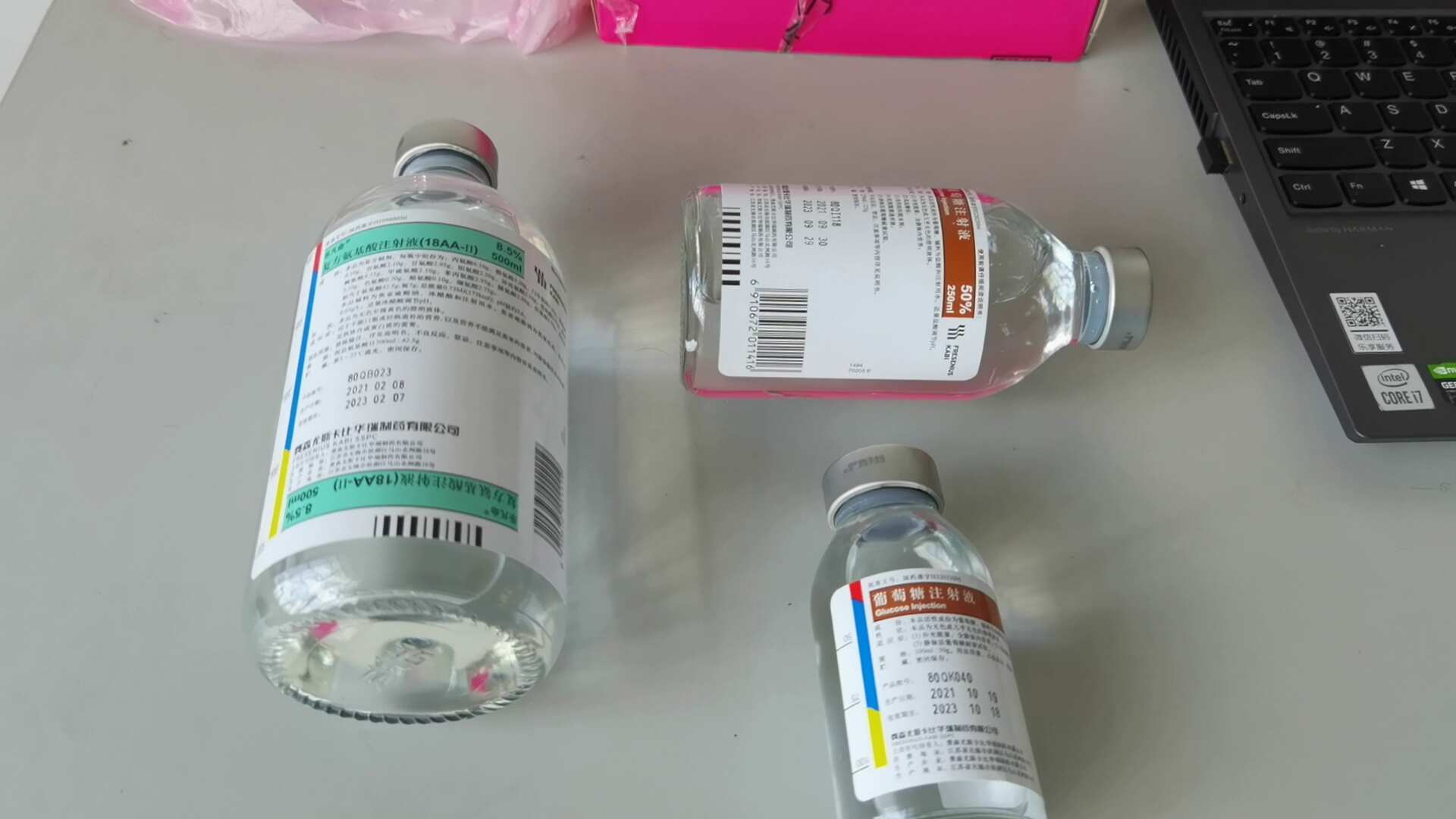

Supplement: S1 Dataset — (ZIP) [file pone.0298109.s001.zip › minimal data set/VOC2007/images/1156.jpg]

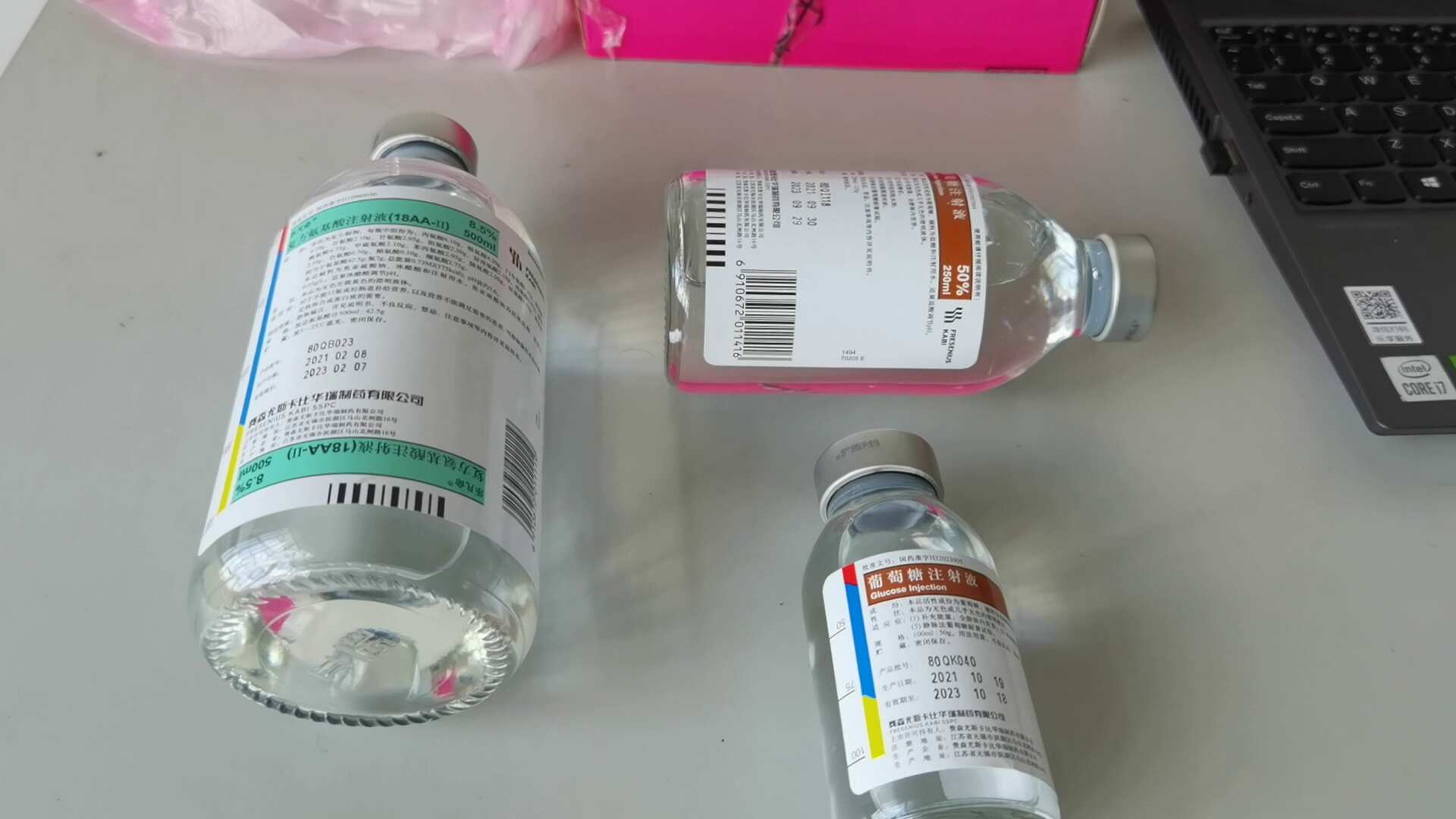

Supplement: S1 Dataset — (ZIP) [file pone.0298109.s001.zip › minimal data set/VOC2007/images/1157.jpg]

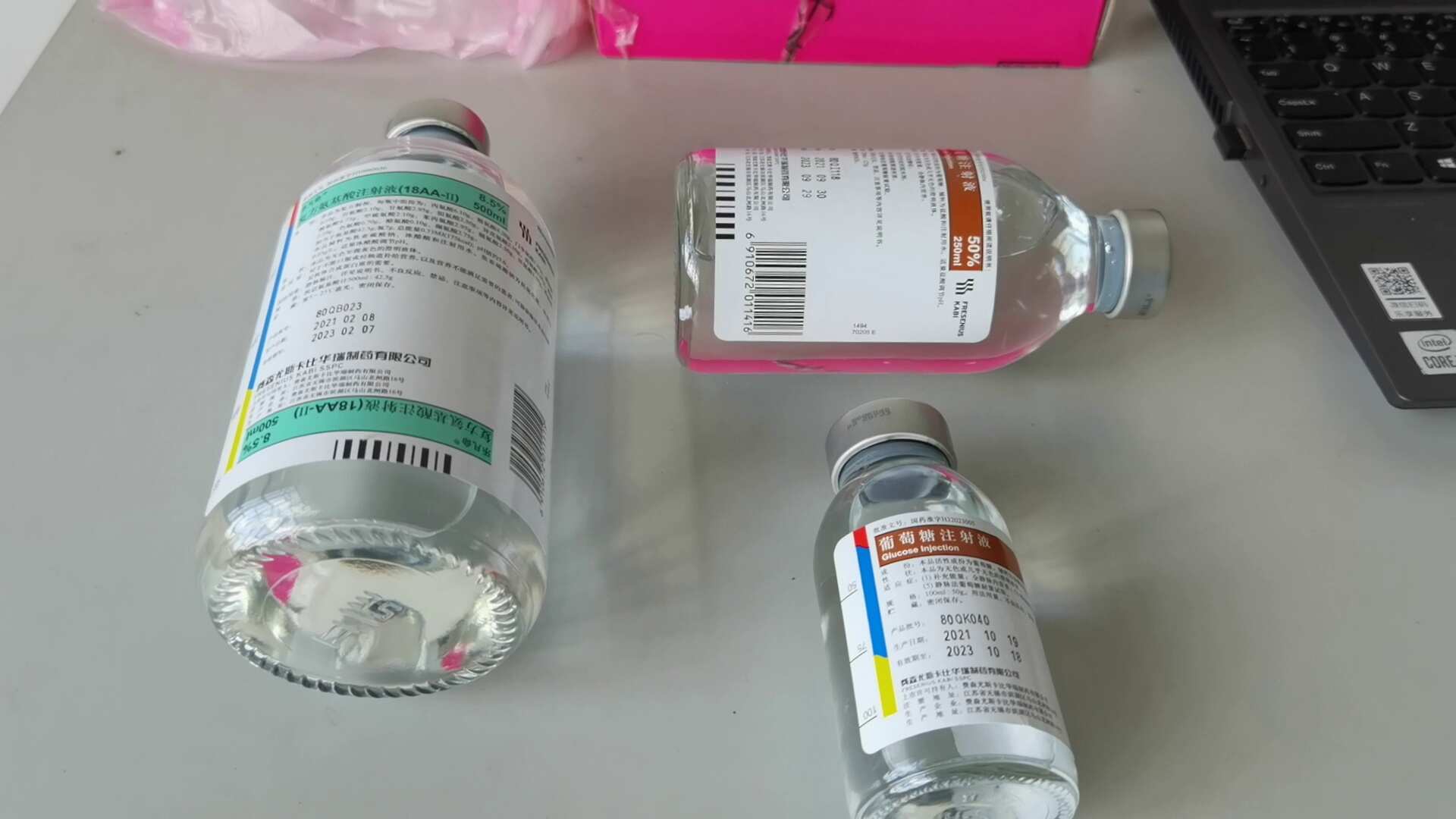

Supplement: S1 Dataset — (ZIP) [file pone.0298109.s001.zip › minimal data set/VOC2007/images/1158.jpg]

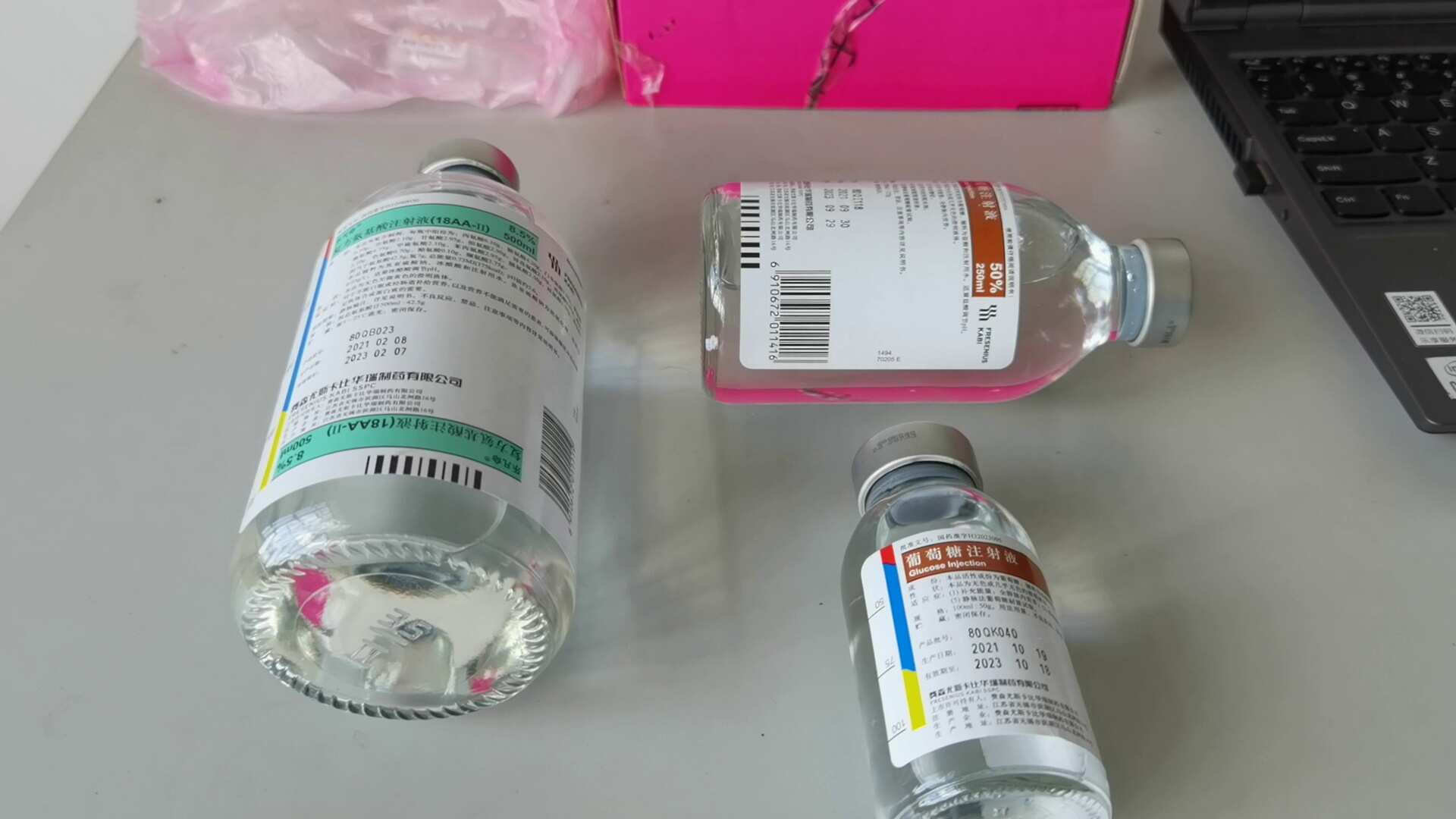

Supplement: S1 Dataset — (ZIP) [file pone.0298109.s001.zip › minimal data set/VOC2007/images/1159.jpg]

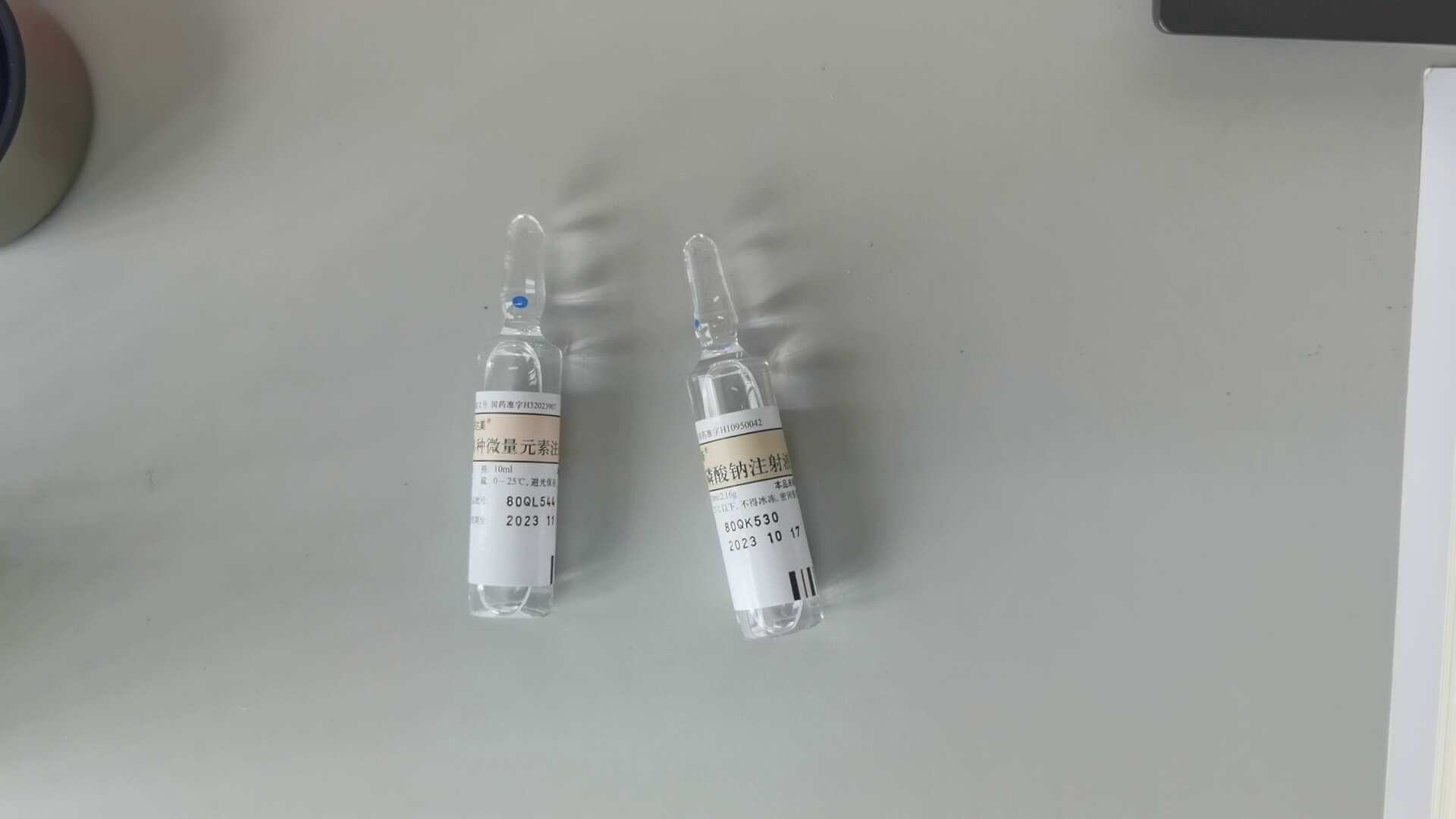

Supplement: S1 Dataset — (ZIP) [file pone.0298109.s001.zip › minimal data set/VOC2007/images/116.jpg]

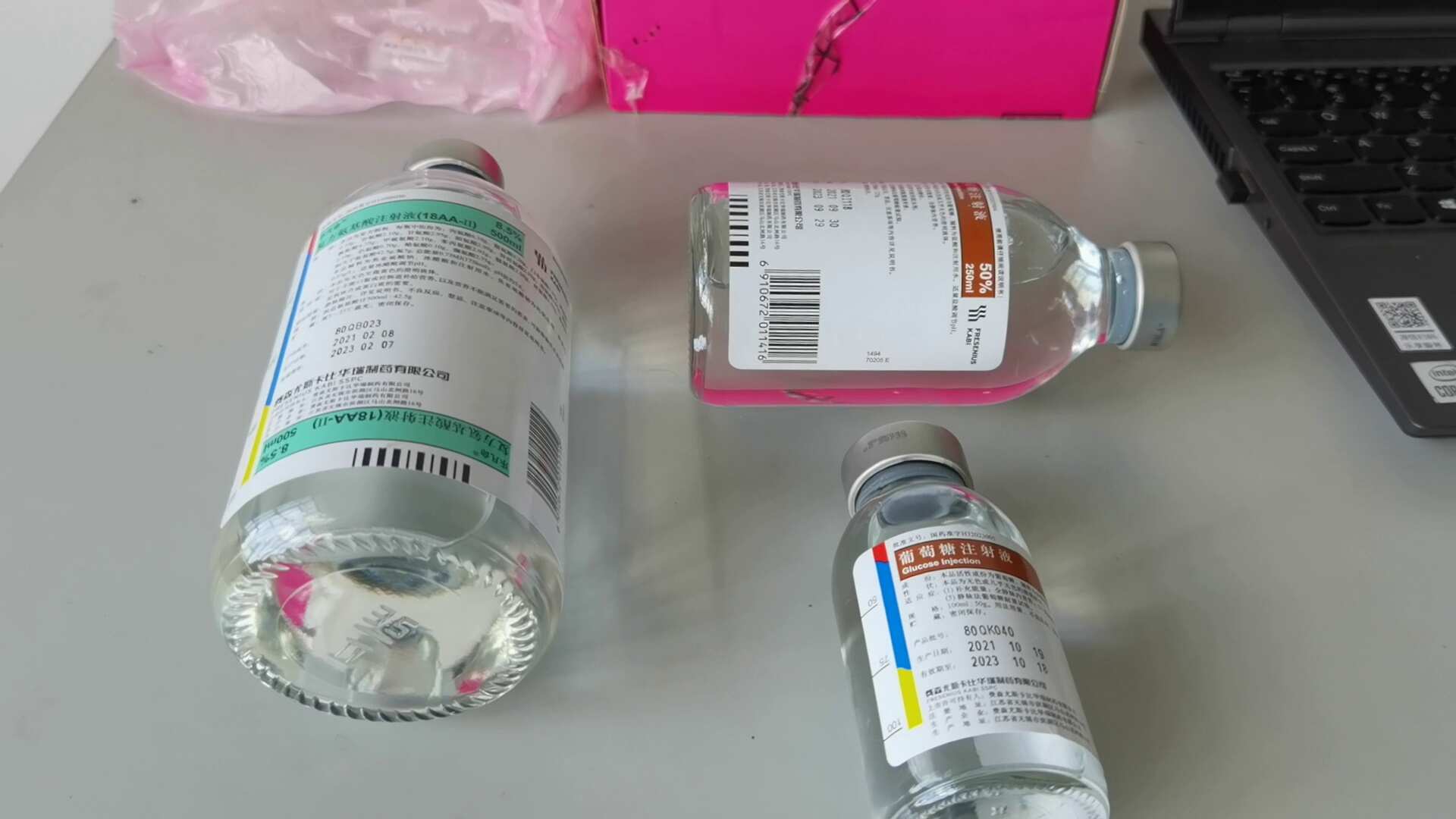

Supplement: S1 Dataset — (ZIP) [file pone.0298109.s001.zip › minimal data set/VOC2007/images/1160.jpg]

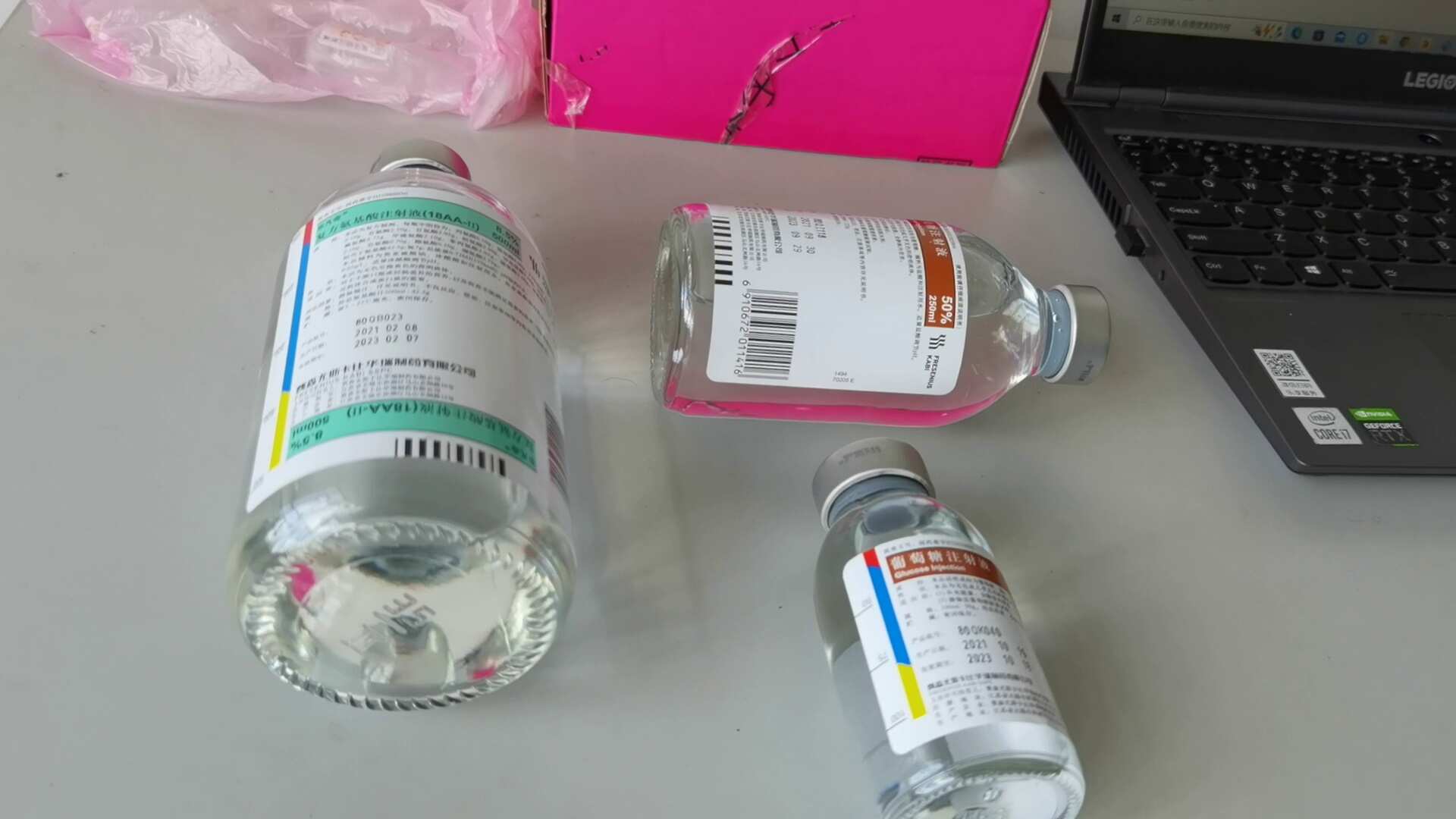

Supplement: S1 Dataset — (ZIP) [file pone.0298109.s001.zip › minimal data set/VOC2007/images/1161.jpg]

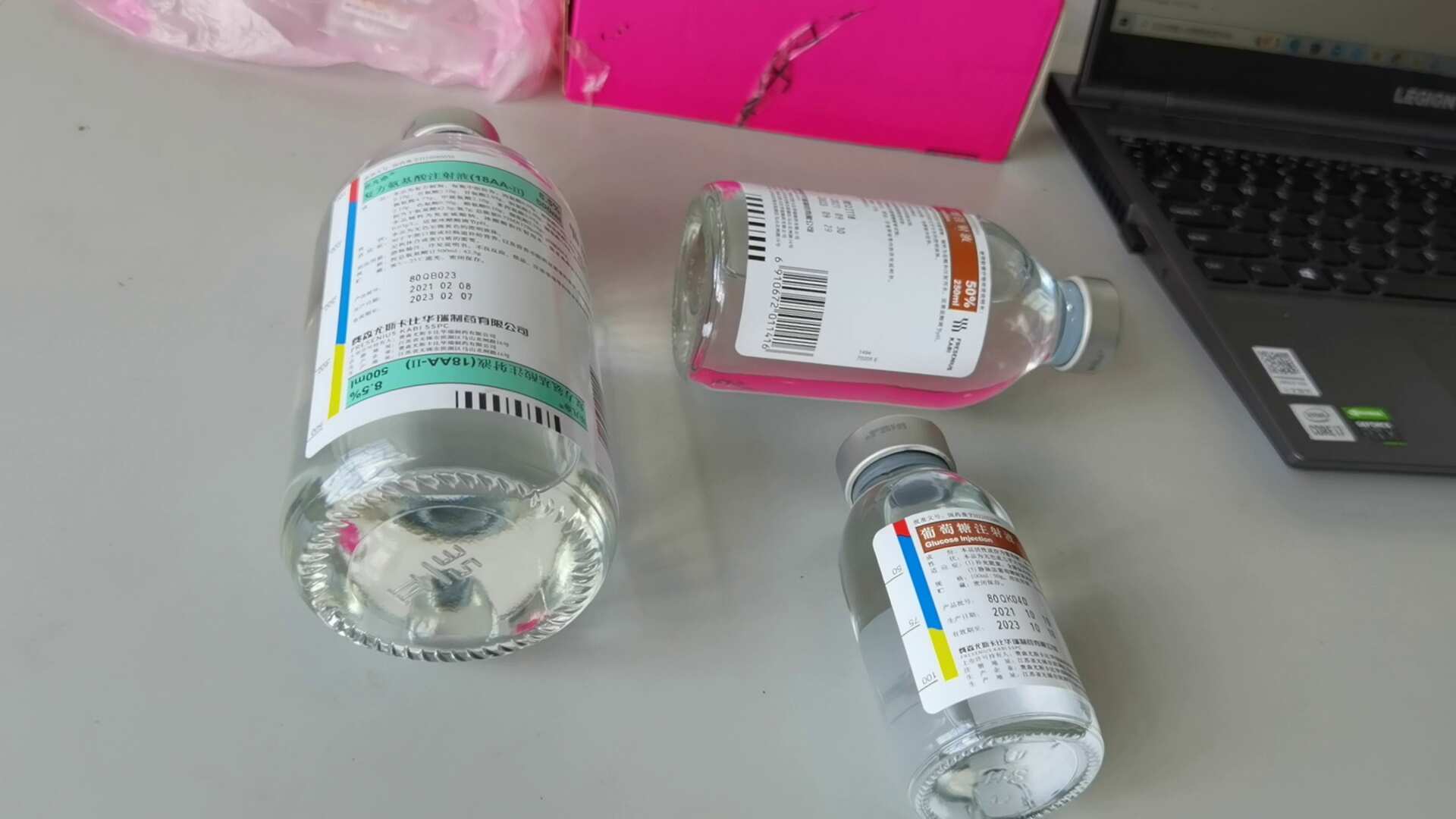

Supplement: S1 Dataset — (ZIP) [file pone.0298109.s001.zip › minimal data set/VOC2007/images/1162.jpg]

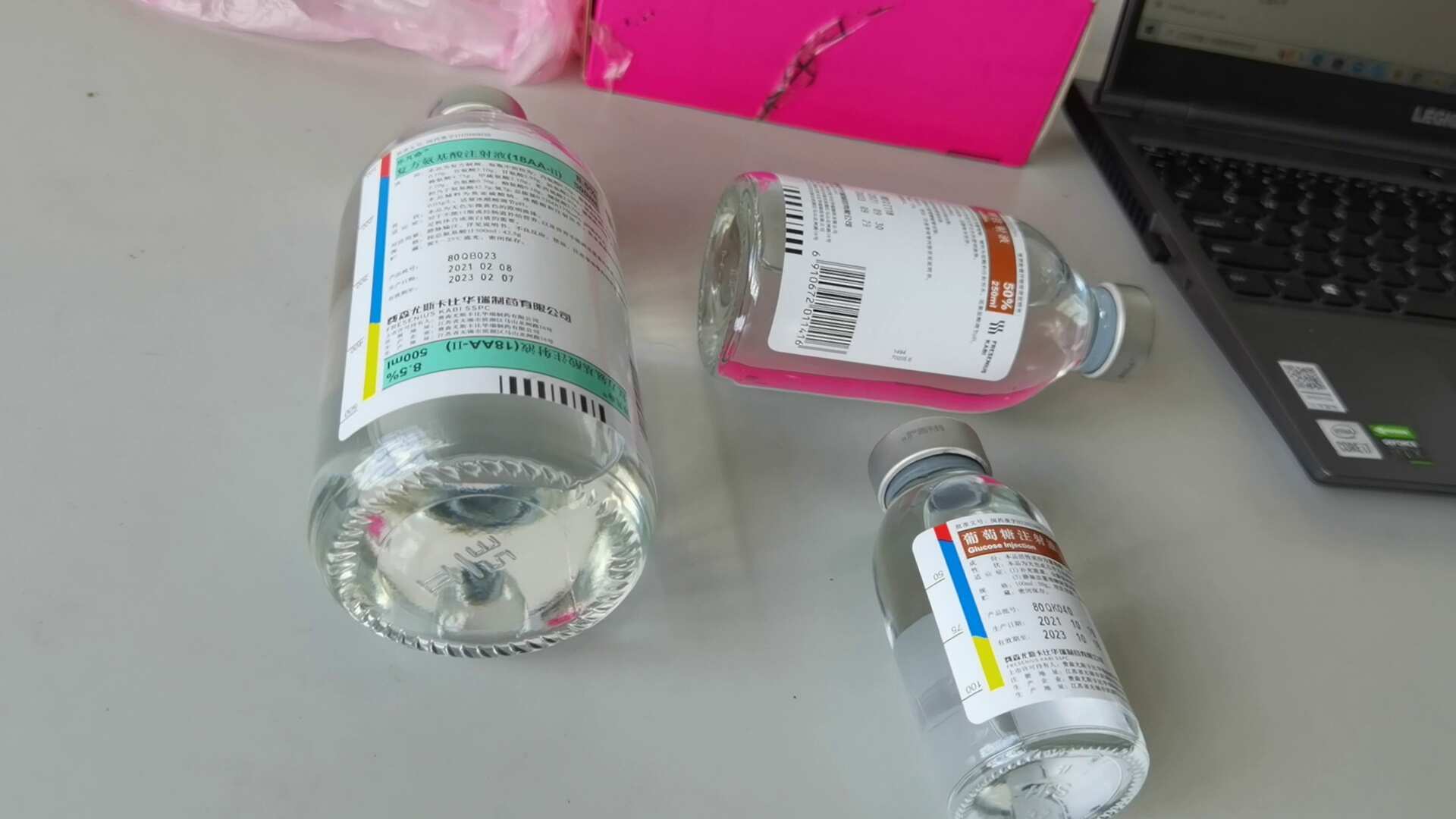

Supplement: S1 Dataset — (ZIP) [file pone.0298109.s001.zip › minimal data set/VOC2007/images/1163.jpg]

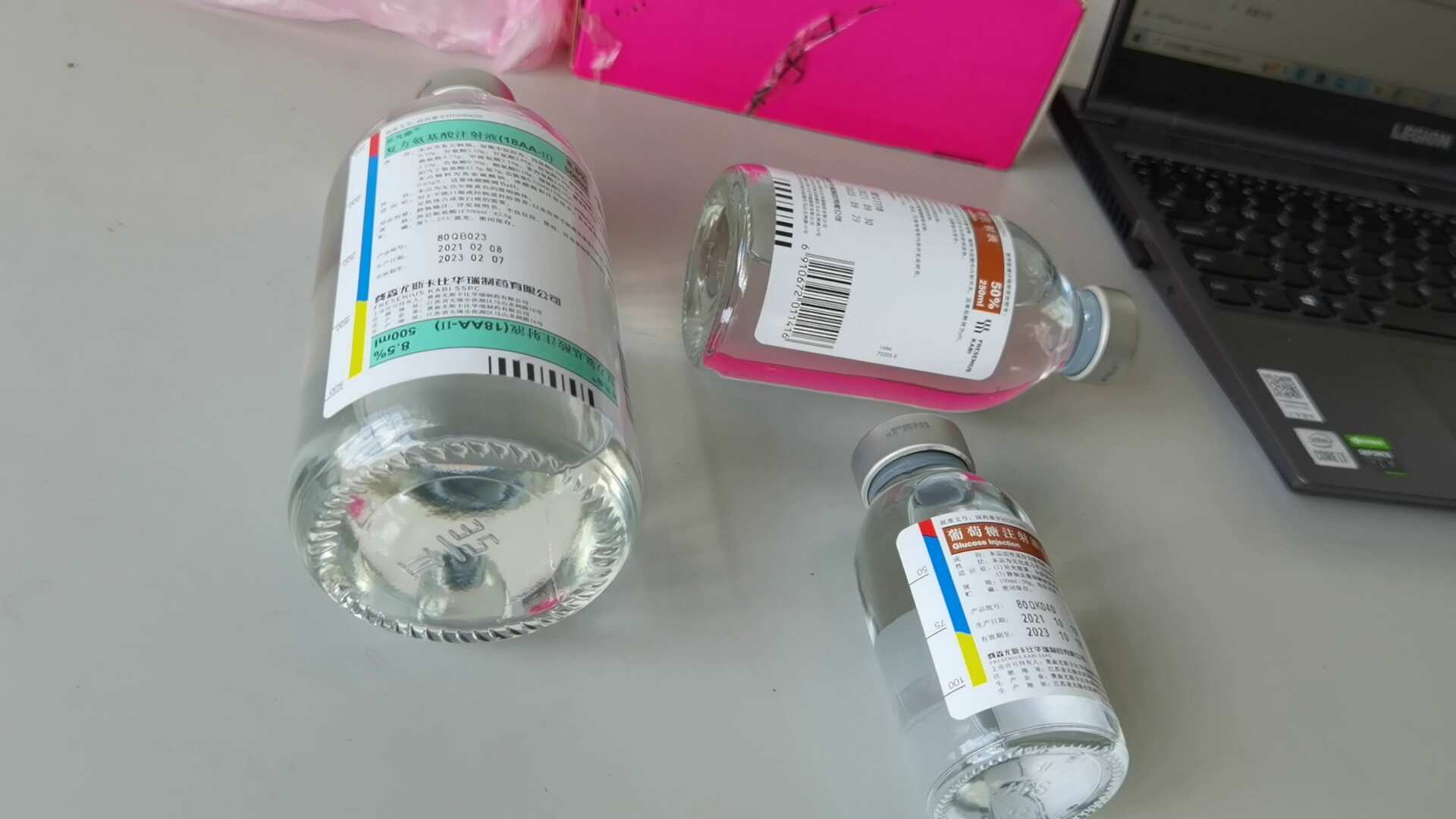

Supplement: S1 Dataset — (ZIP) [file pone.0298109.s001.zip › minimal data set/VOC2007/images/1164.jpg]

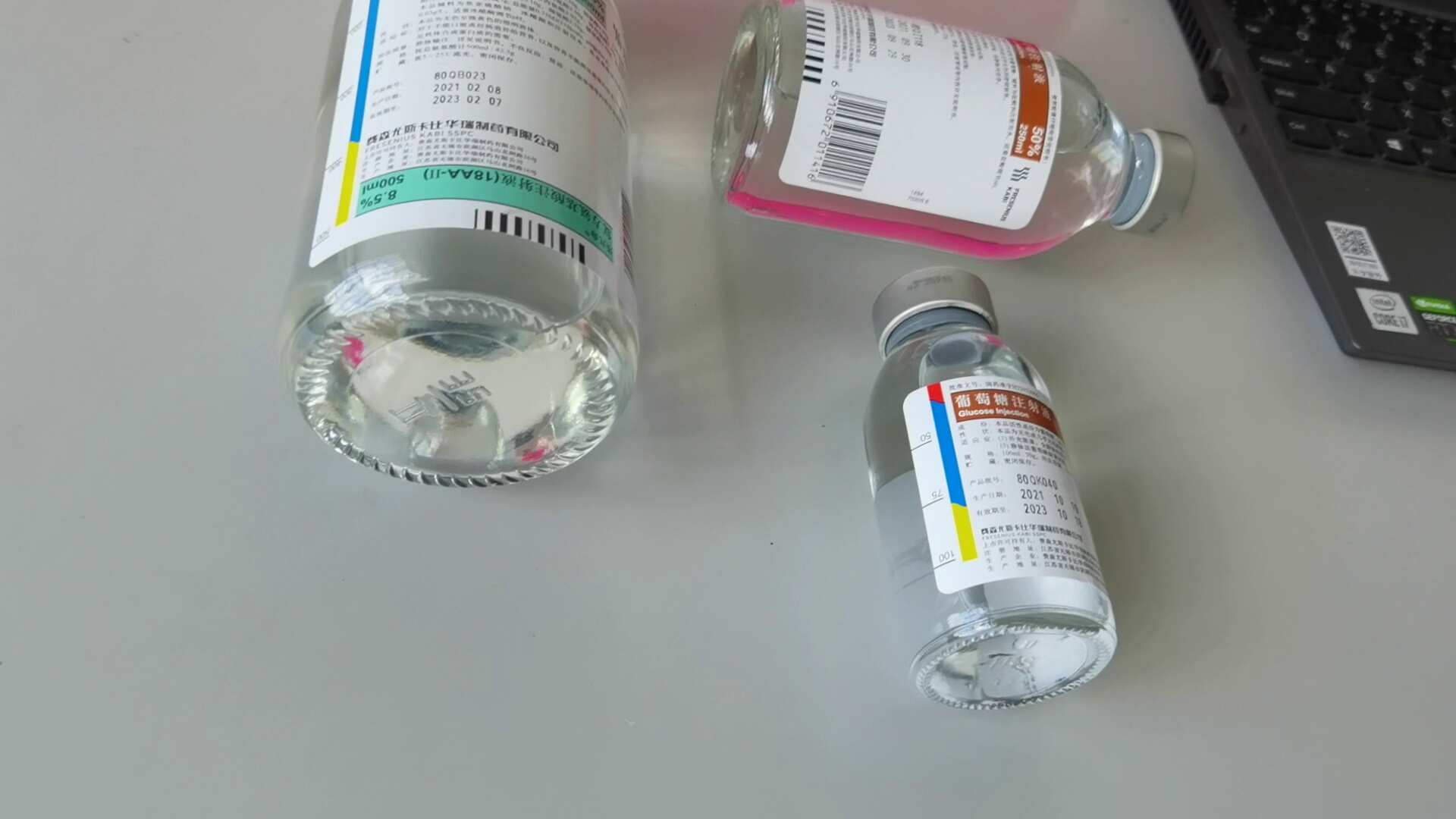

Supplement: S1 Dataset — (ZIP) [file pone.0298109.s001.zip › minimal data set/VOC2007/images/1165.jpg]

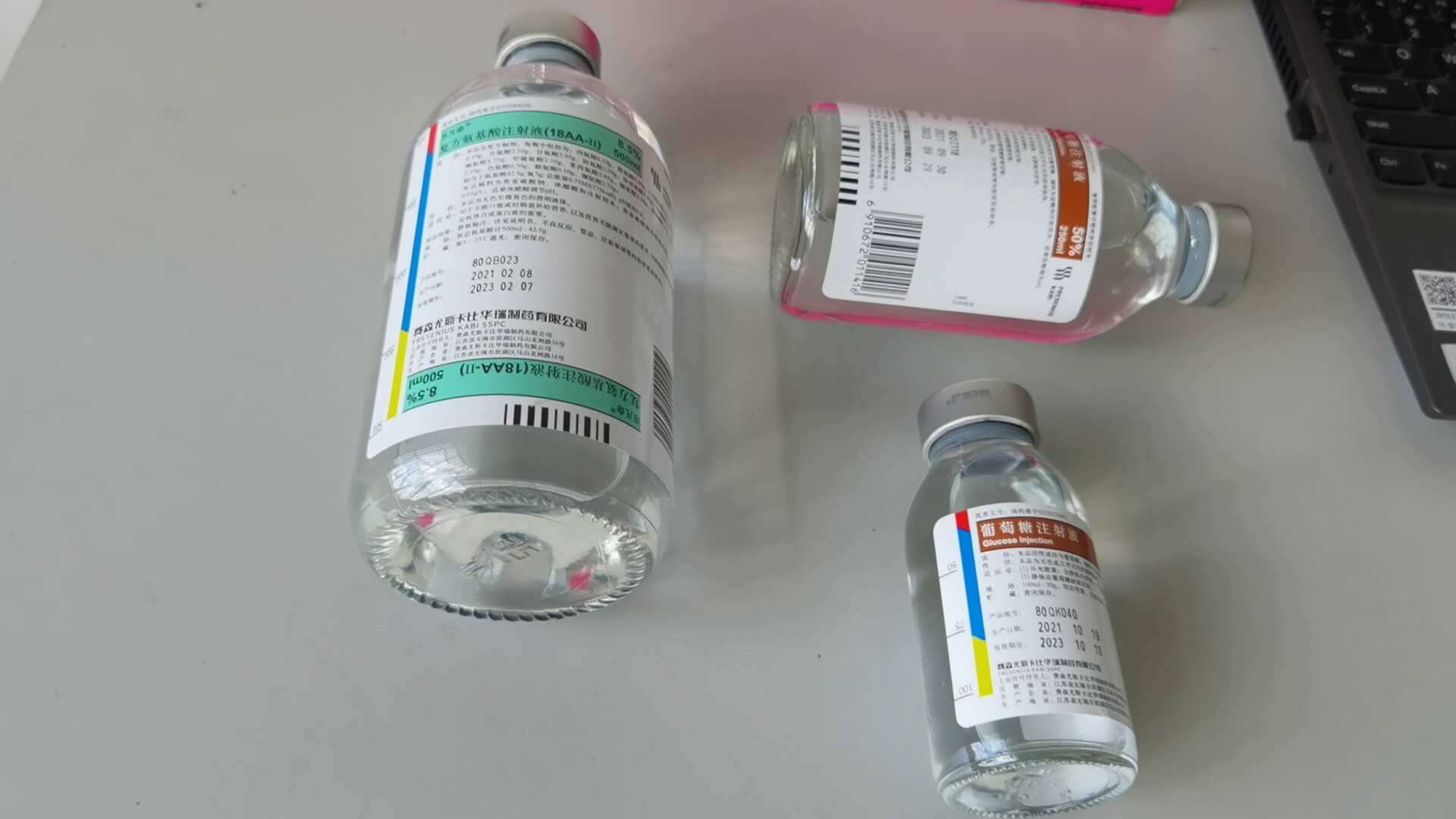

Supplement: S1 Dataset — (ZIP) [file pone.0298109.s001.zip › minimal data set/VOC2007/images/1166.jpg]

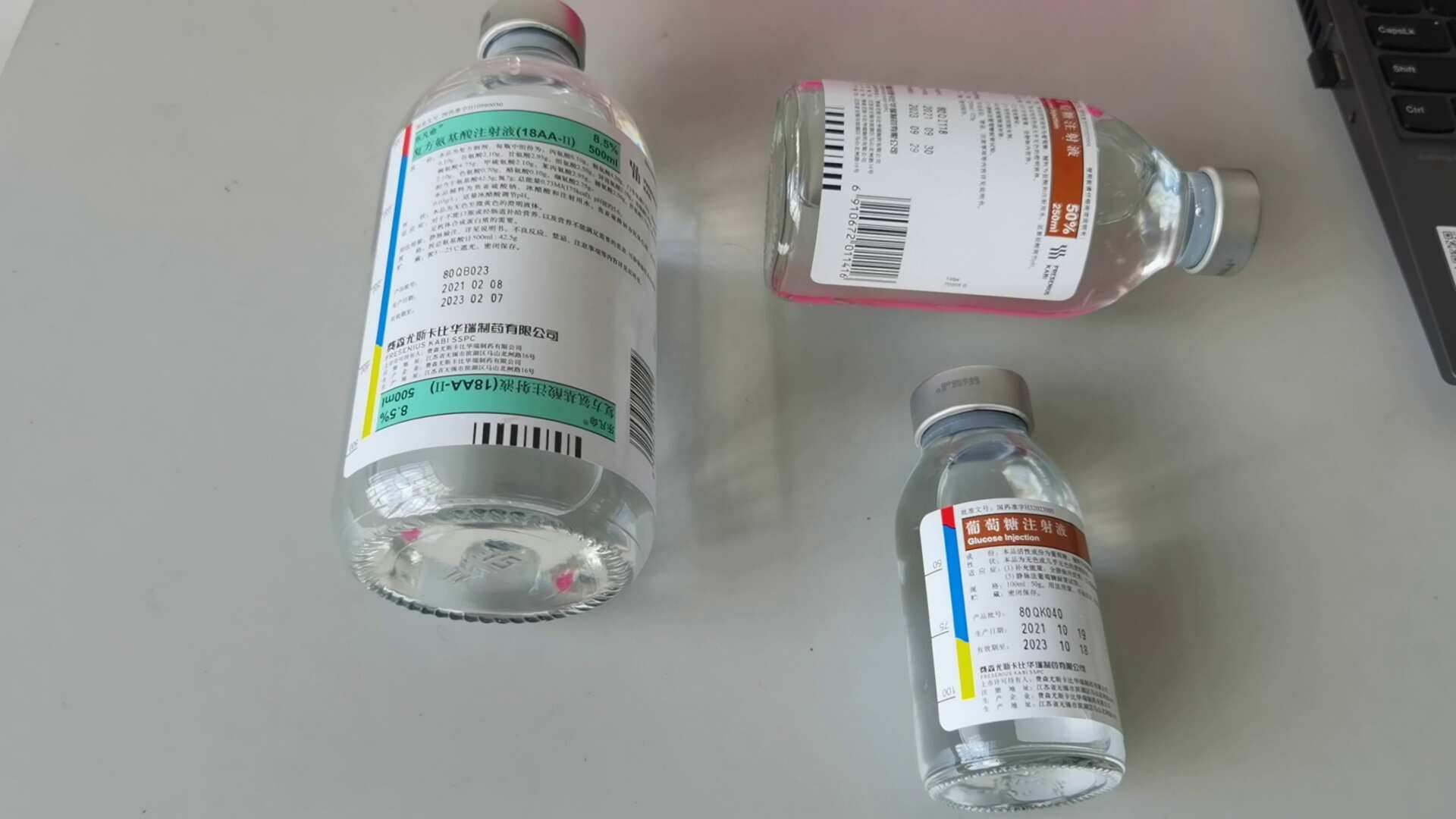

Supplement: S1 Dataset — (ZIP) [file pone.0298109.s001.zip › minimal data set/VOC2007/images/1167.jpg]

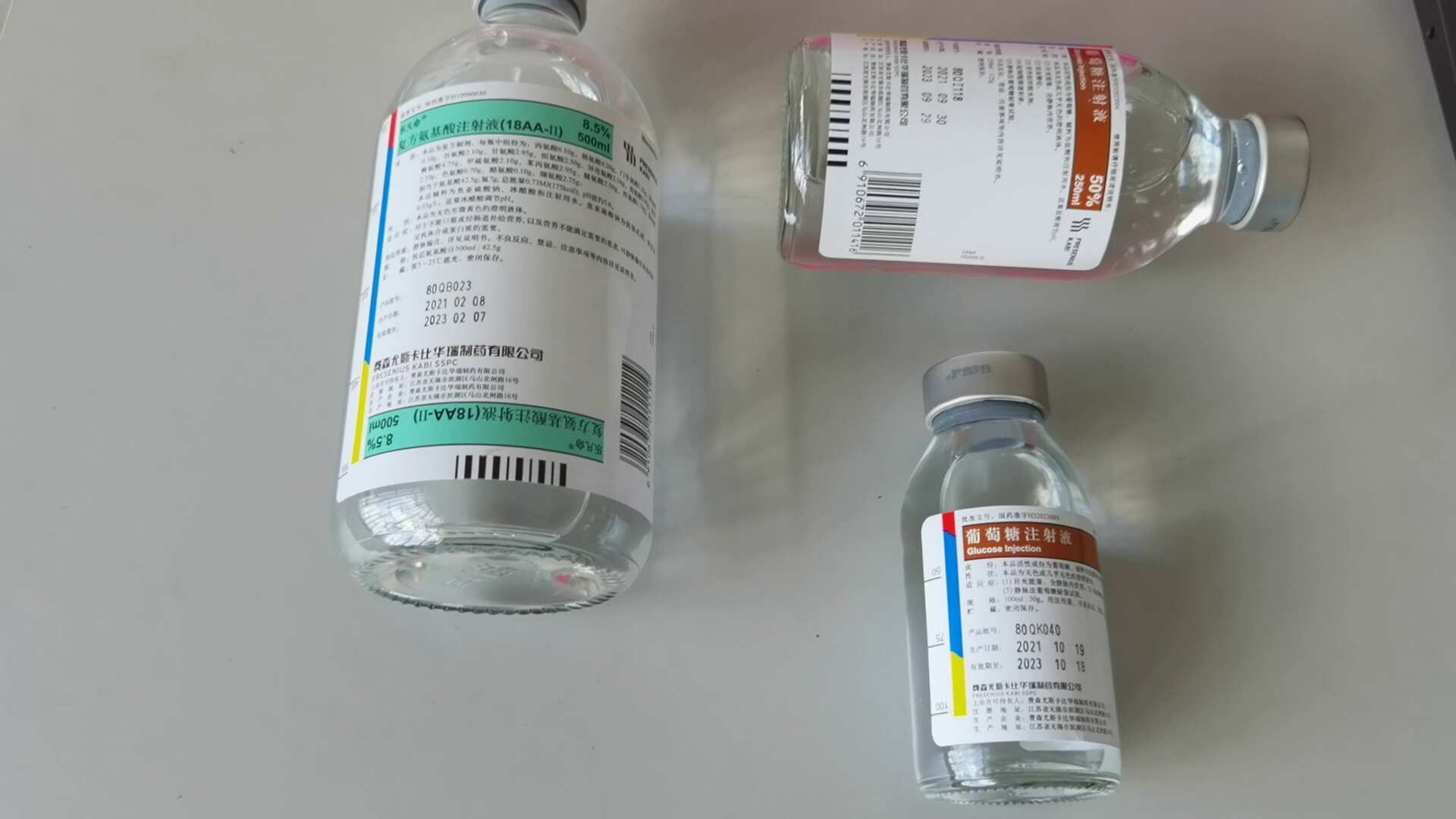

Supplement: S1 Dataset — (ZIP) [file pone.0298109.s001.zip › minimal data set/VOC2007/images/1168.jpg]

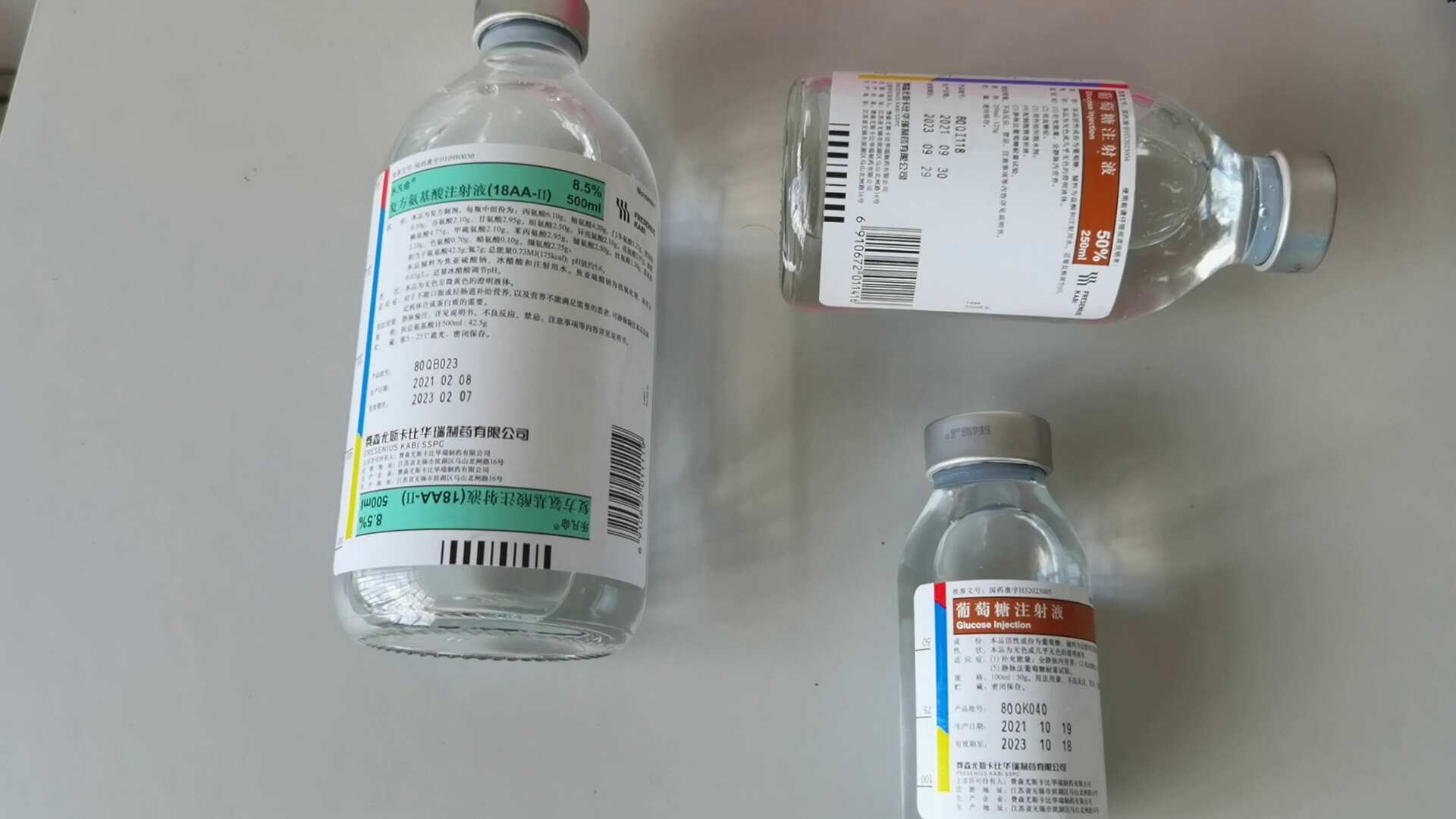

Supplement: S1 Dataset — (ZIP) [file pone.0298109.s001.zip › minimal data set/VOC2007/images/1169.jpg]

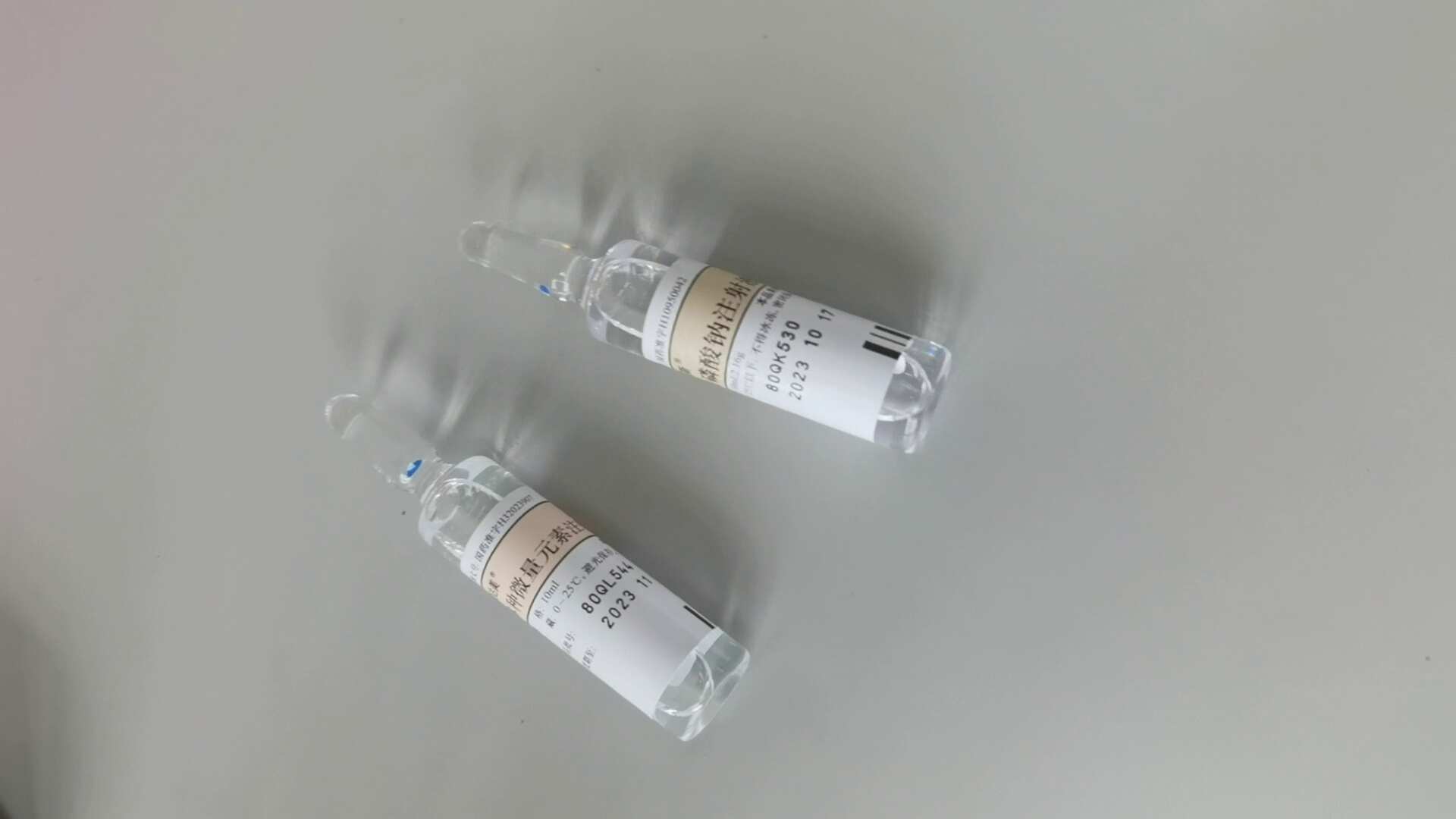

Supplement: S1 Dataset — (ZIP) [file pone.0298109.s001.zip › minimal data set/VOC2007/images/117.jpg]

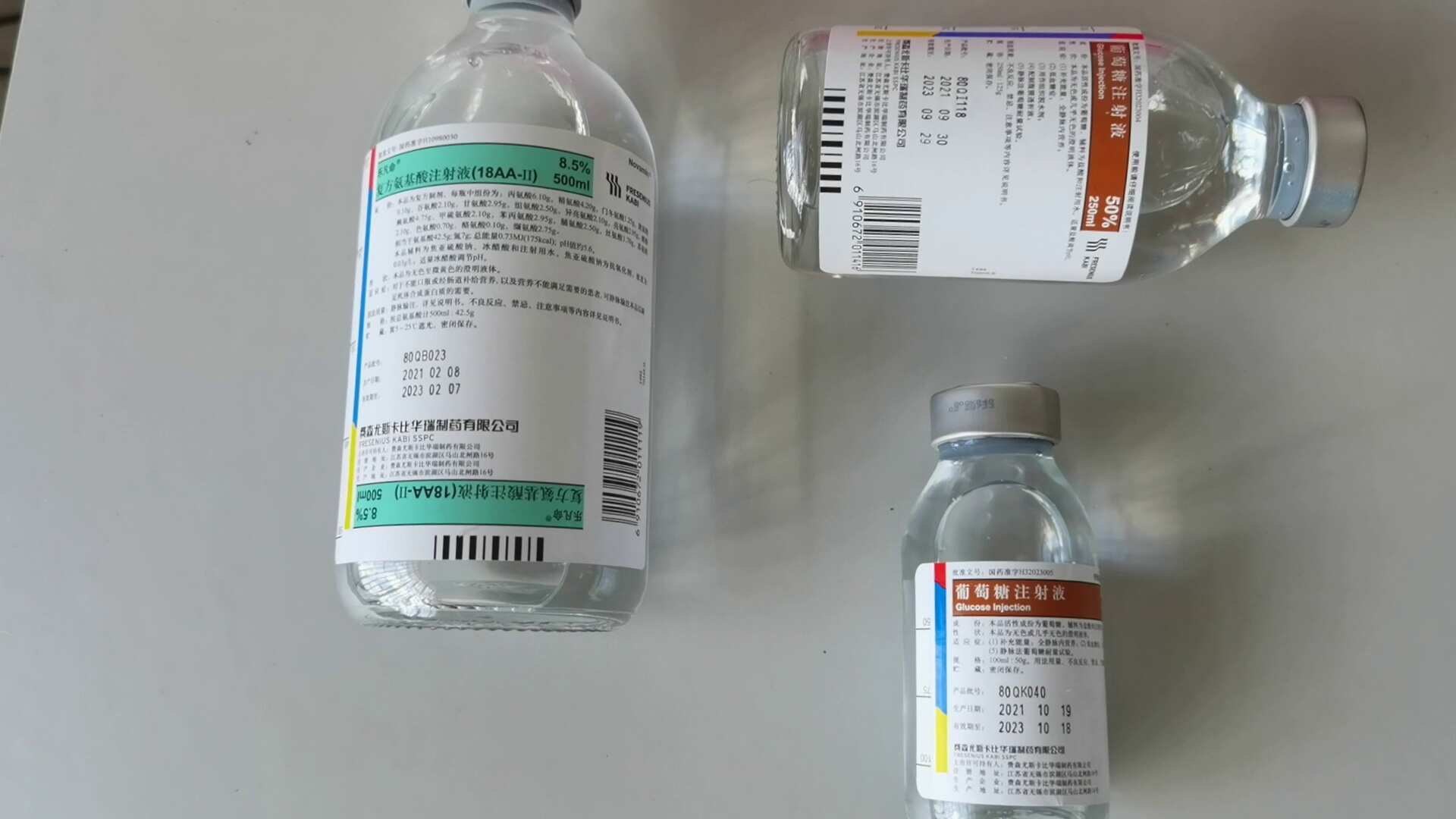

Supplement: S1 Dataset — (ZIP) [file pone.0298109.s001.zip › minimal data set/VOC2007/images/1170.jpg]

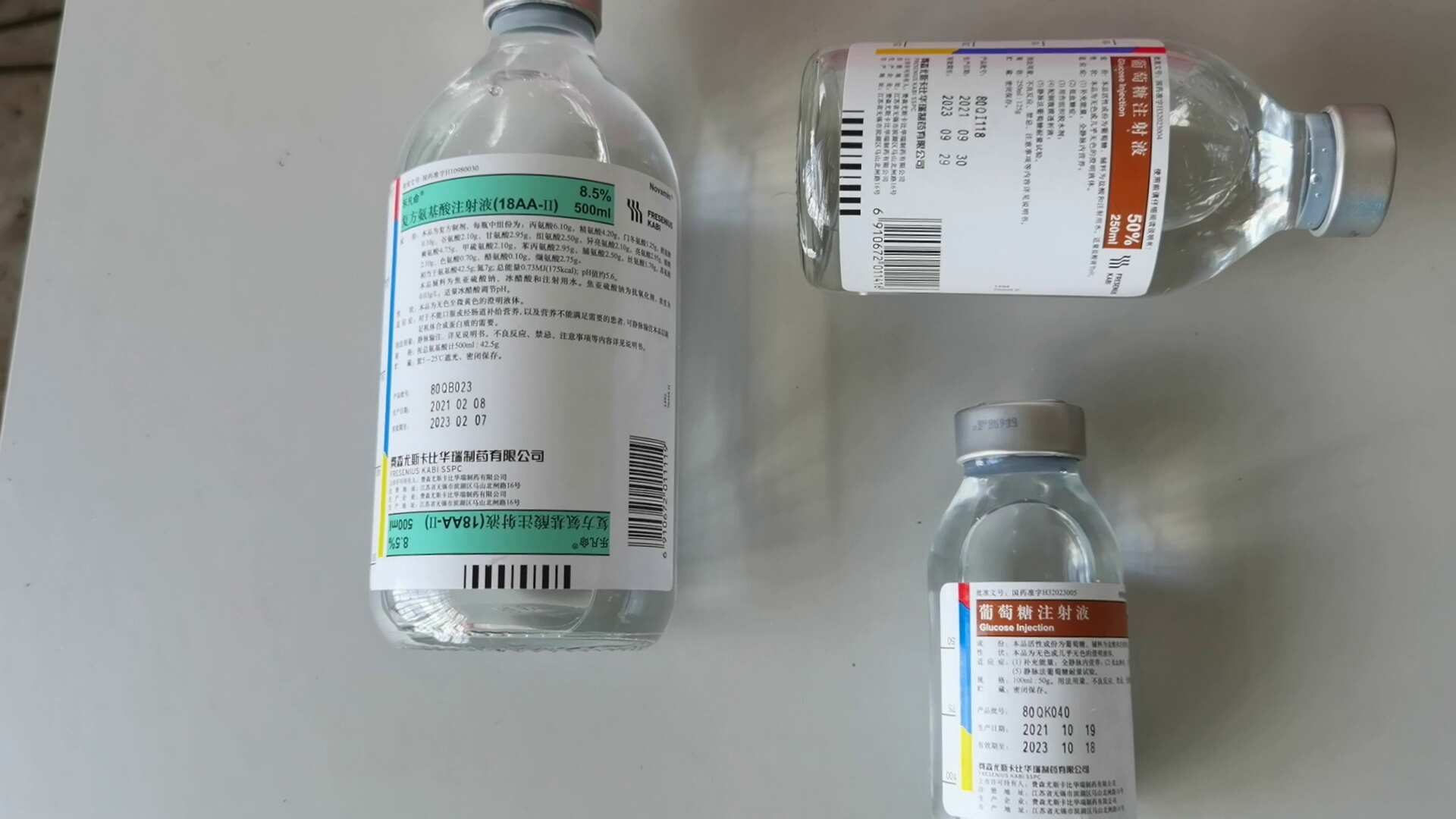

Supplement: S1 Dataset — (ZIP) [file pone.0298109.s001.zip › minimal data set/VOC2007/images/1171.jpg]

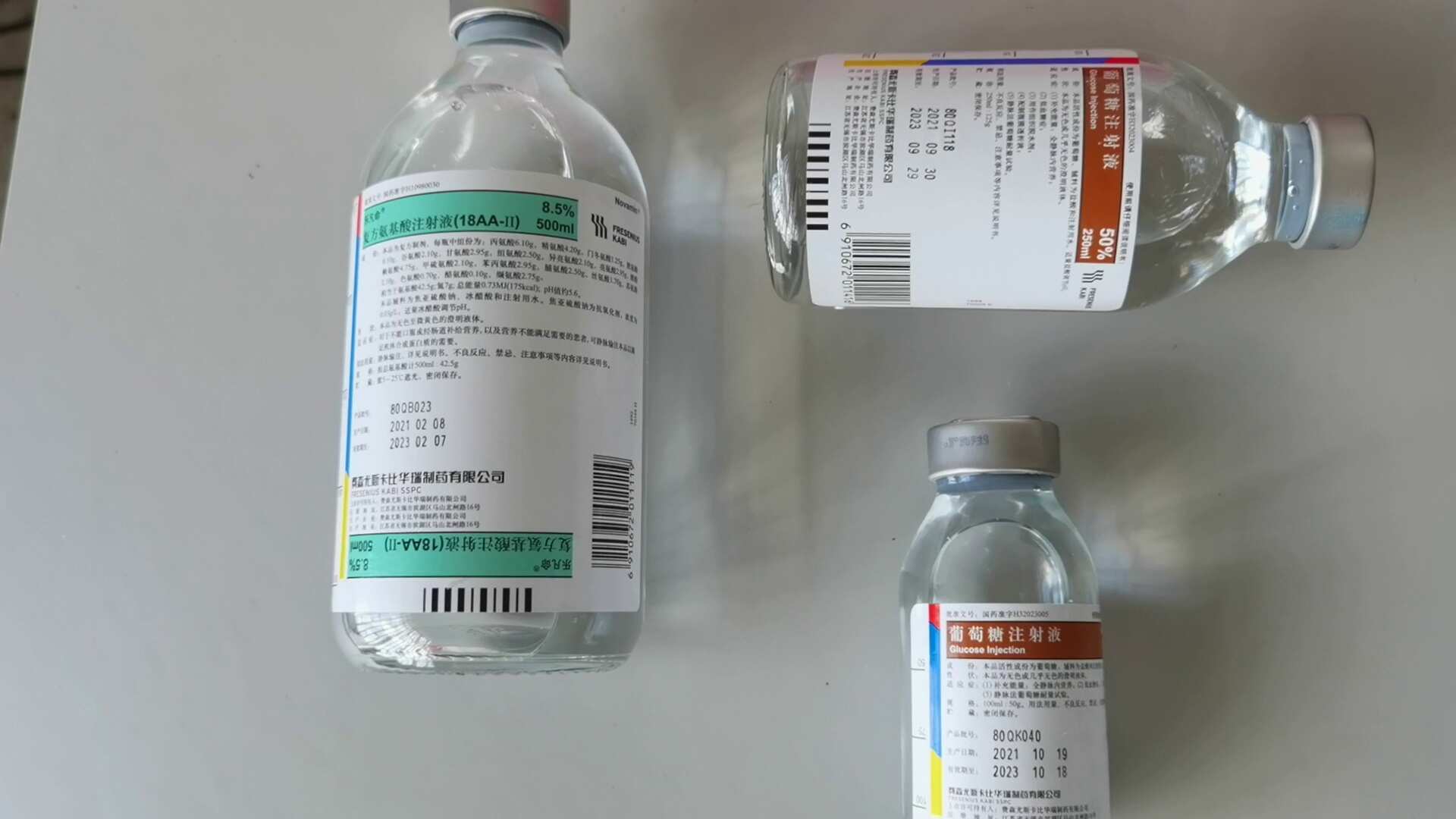

Supplement: S1 Dataset — (ZIP) [file pone.0298109.s001.zip › minimal data set/VOC2007/images/1172.jpg]

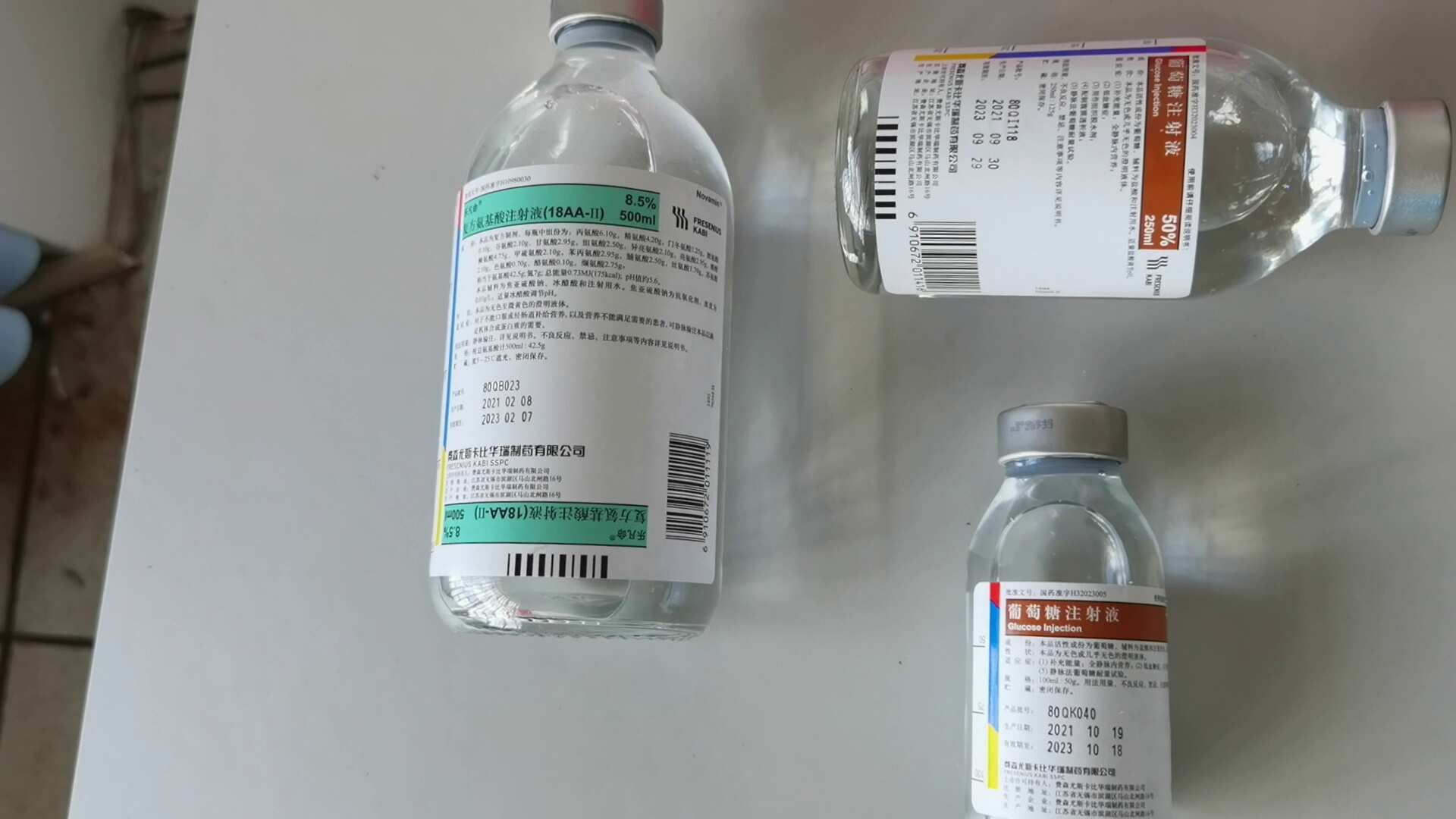

Supplement: S1 Dataset — (ZIP) [file pone.0298109.s001.zip › minimal data set/VOC2007/images/1173.jpg]

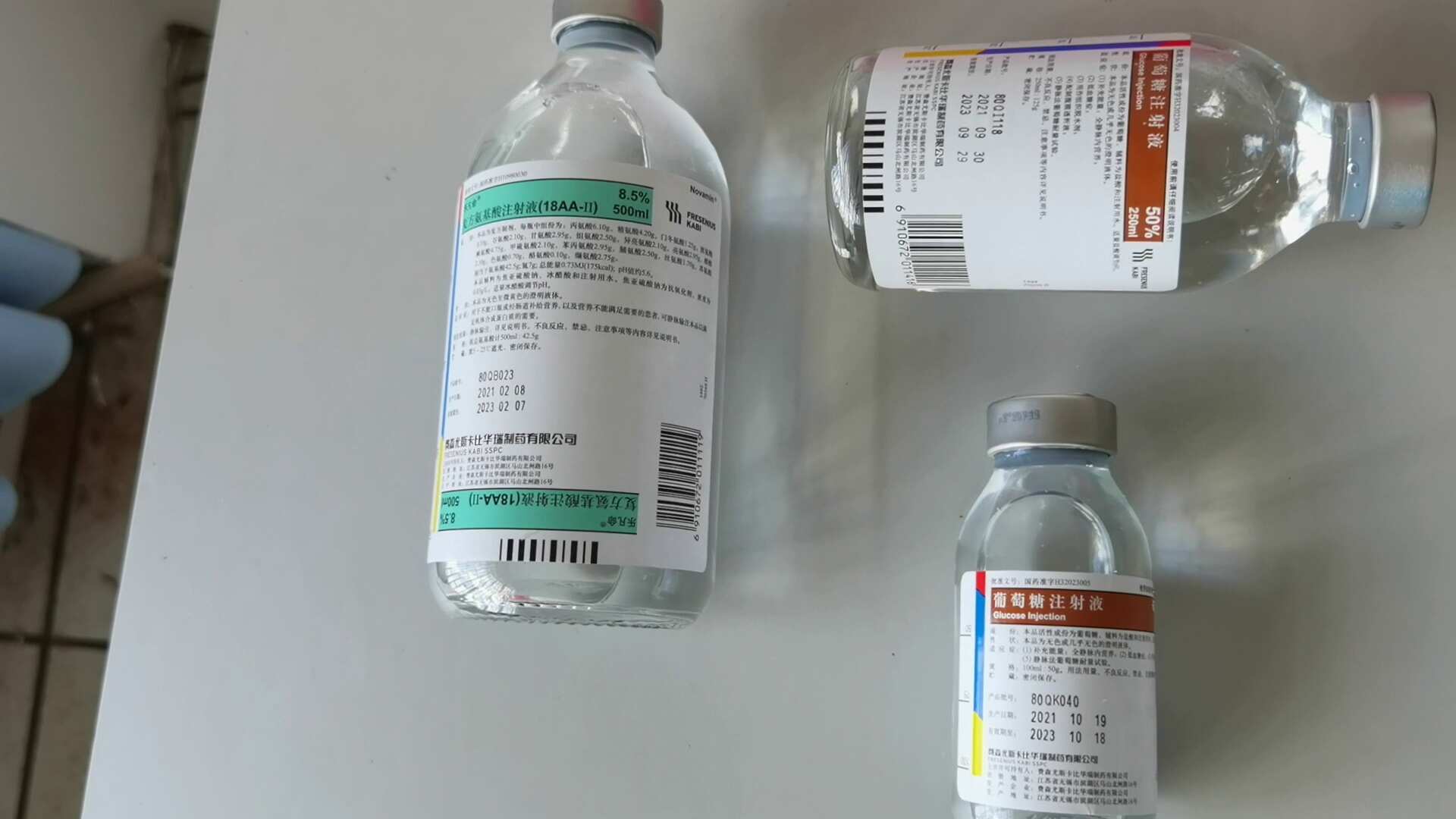

Supplement: S1 Dataset — (ZIP) [file pone.0298109.s001.zip › minimal data set/VOC2007/images/1174.jpg]

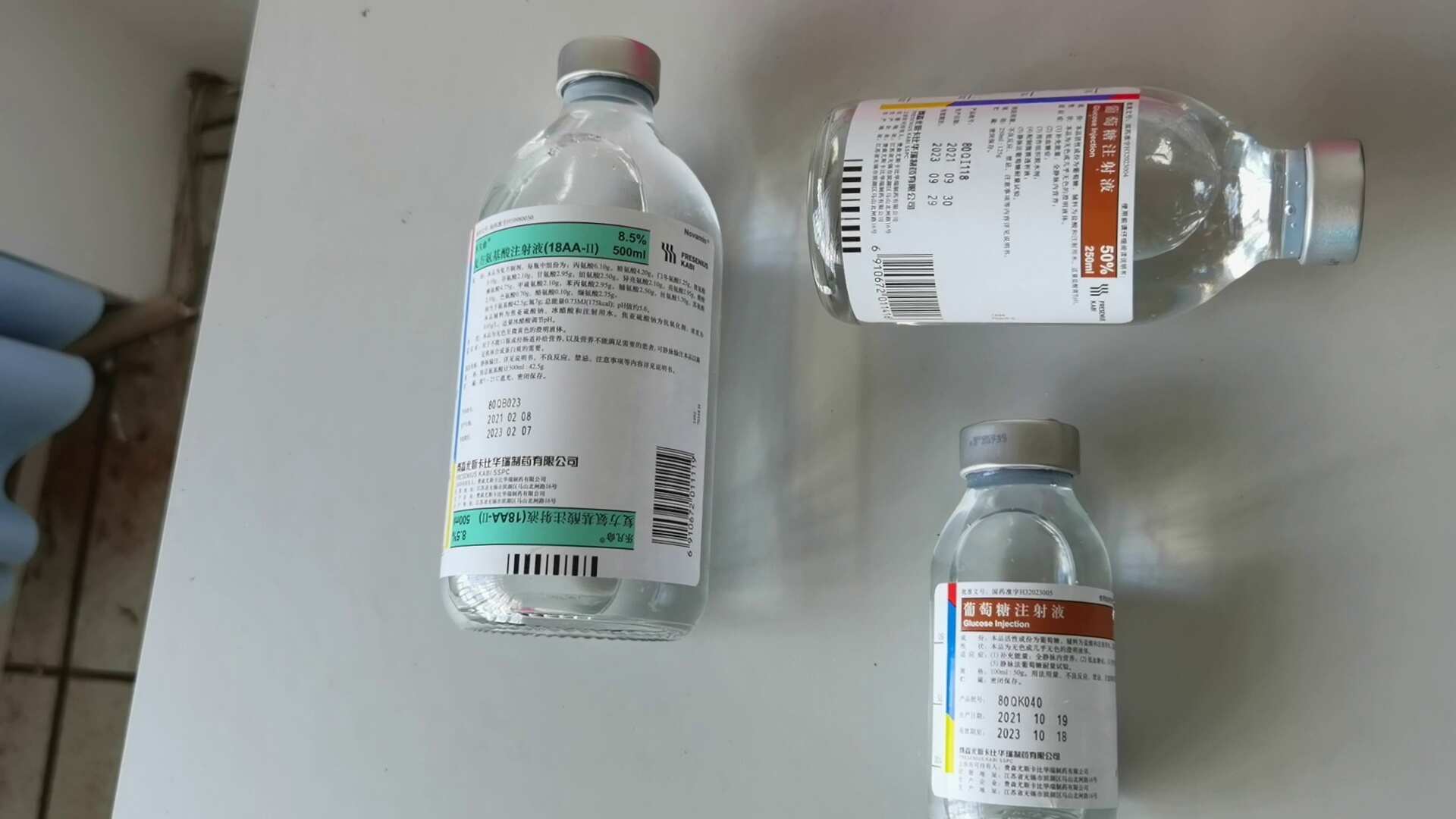

Supplement: S1 Dataset — (ZIP) [file pone.0298109.s001.zip › minimal data set/VOC2007/images/1175.jpg]

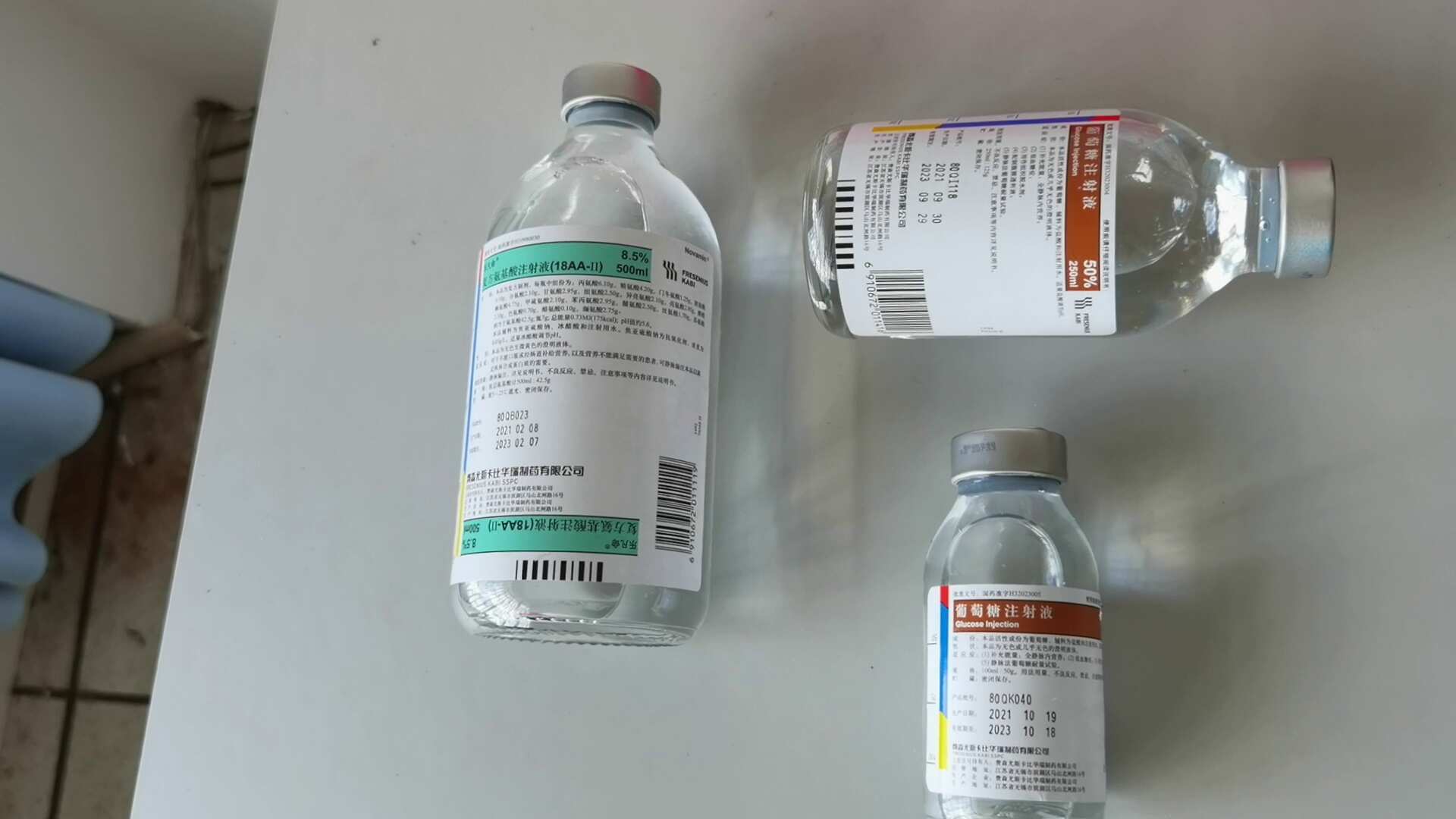

Supplement: S1 Dataset — (ZIP) [file pone.0298109.s001.zip › minimal data set/VOC2007/images/1176.jpg]

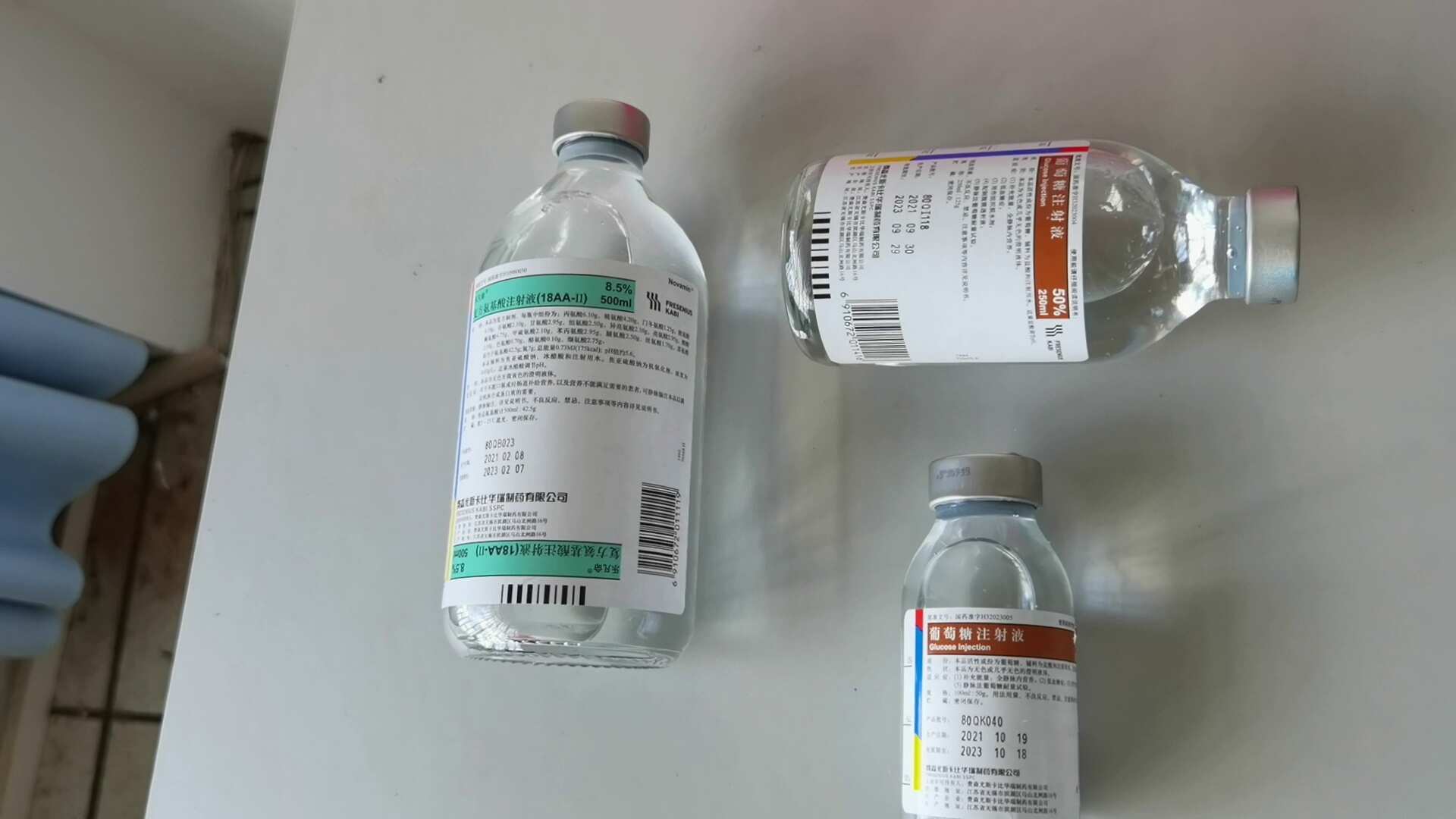

Supplement: S1 Dataset — (ZIP) [file pone.0298109.s001.zip › minimal data set/VOC2007/images/1177.jpg]

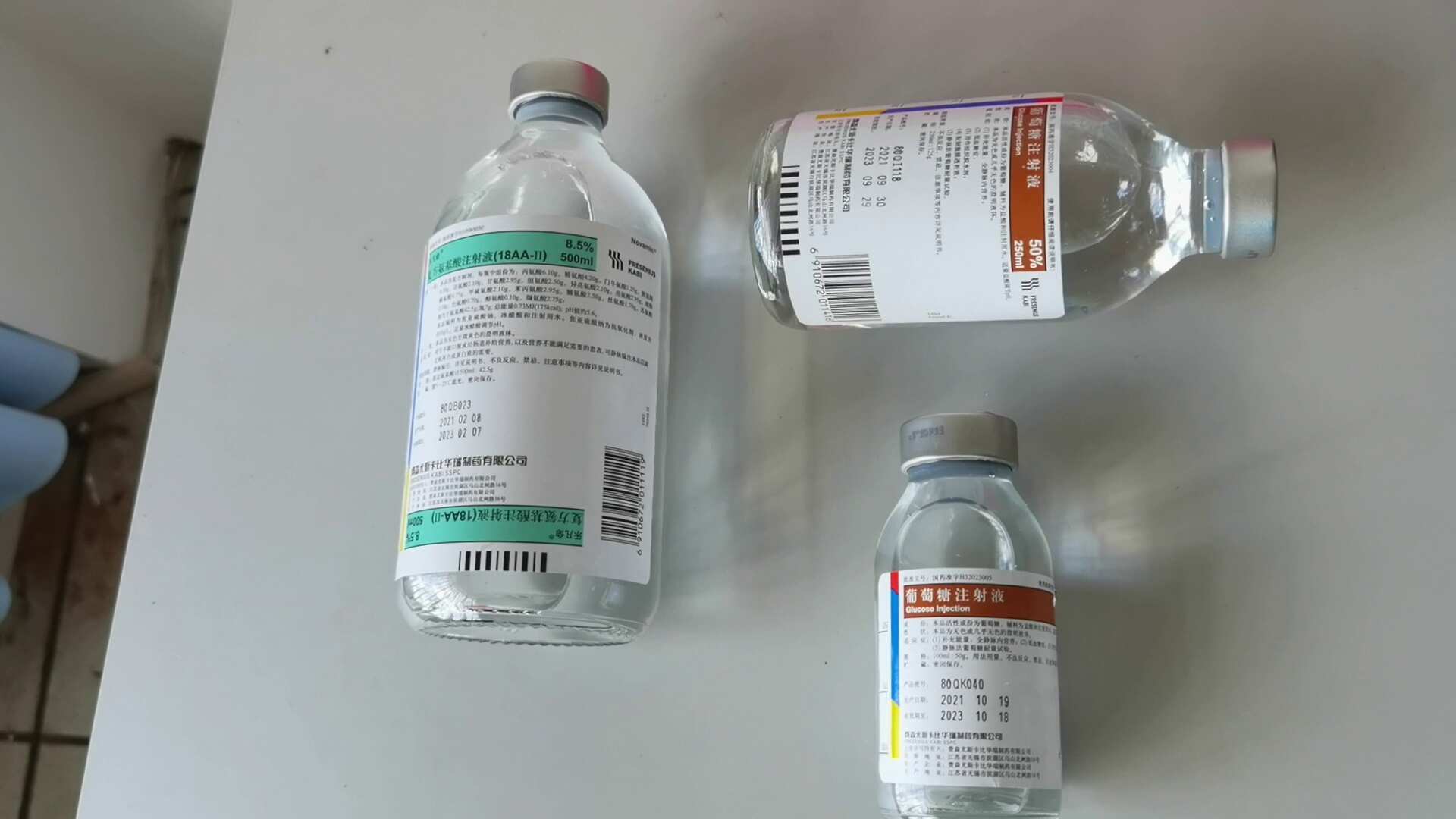

Supplement: S1 Dataset — (ZIP) [file pone.0298109.s001.zip › minimal data set/VOC2007/images/1178.jpg]
