# Supplementary figures and images for: Intelligent supervision of PIVAS drug dispensing based on image recognition technology (part 1 of 2)
Source: PLoS One. 2024 Apr 4;19(4):e0298109. doi: 10.1371/journal.pone.0298109 (PMC10994394; doi:10.1371/journal.pone.0298109)

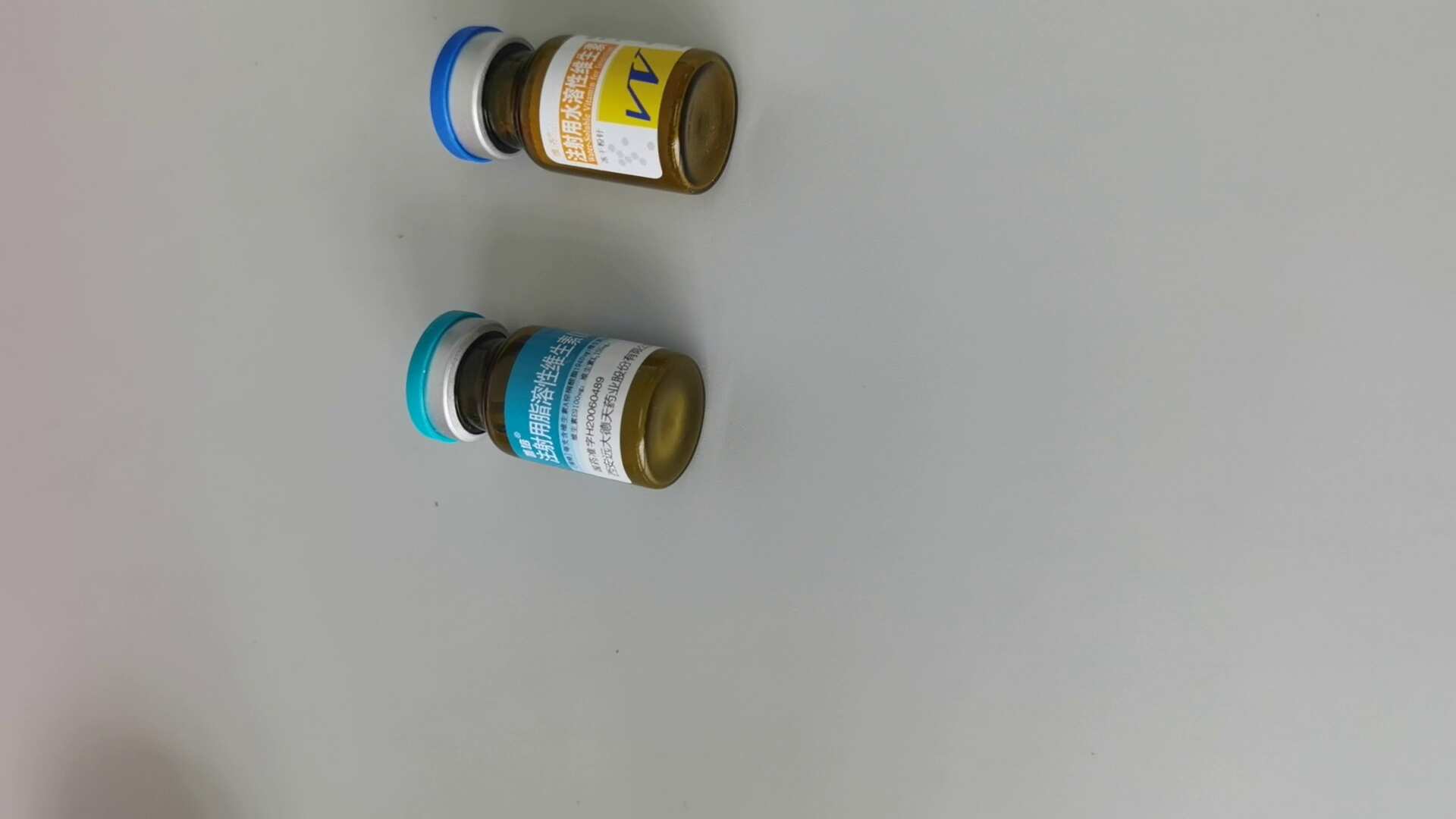

Supplement: S1 Dataset — (ZIP) [file pone.0298109.s001.zip › minimal data set/VOC2007/images/1.jpg]

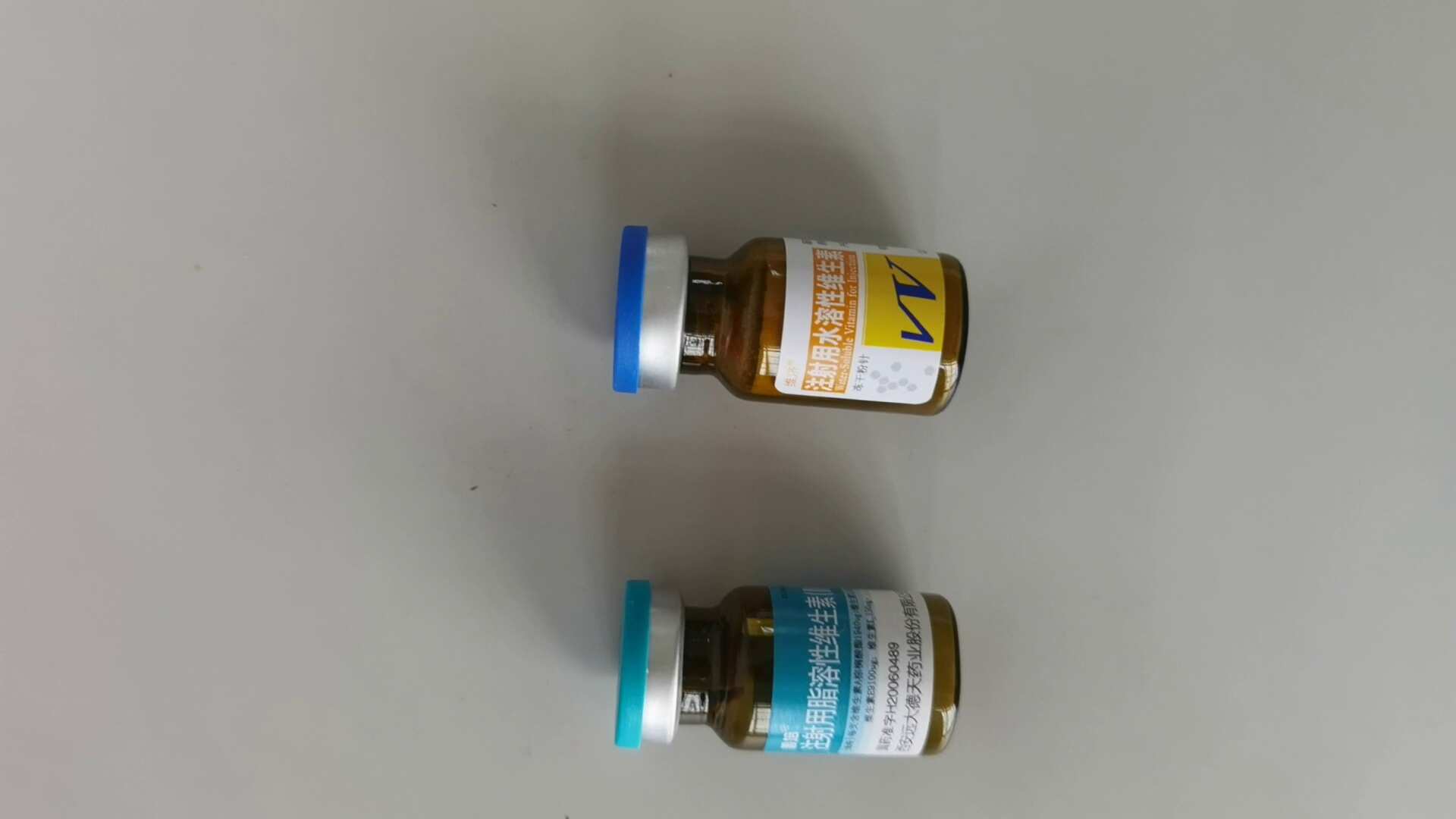

Supplement: S1 Dataset — (ZIP) [file pone.0298109.s001.zip › minimal data set/VOC2007/images/10.jpg]

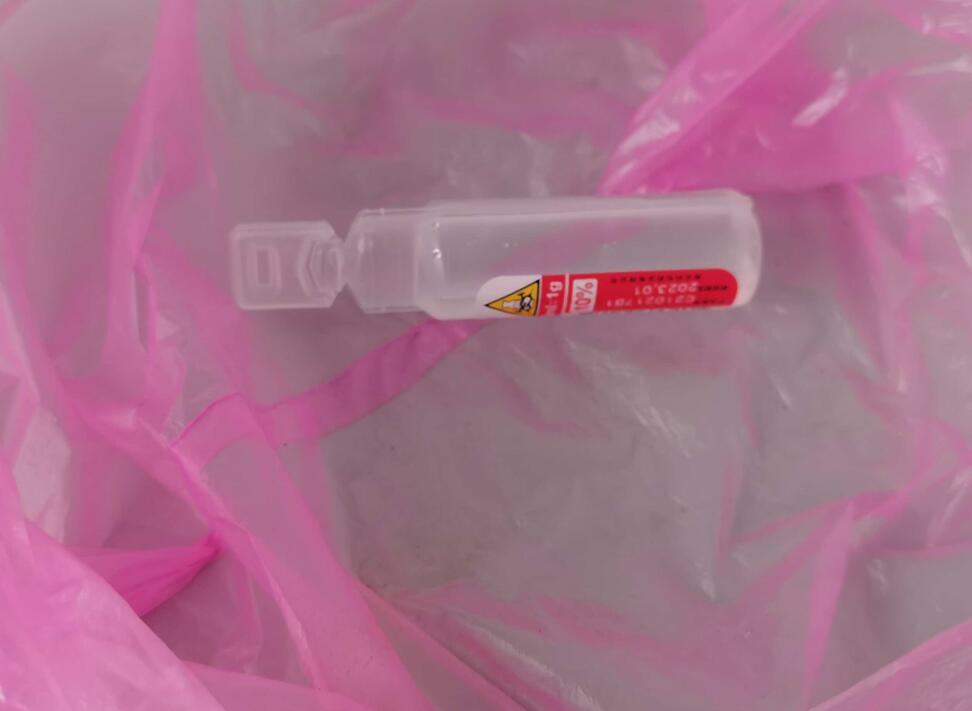

Supplement: S1 Dataset — (ZIP) [file pone.0298109.s001.zip › minimal data set/VOC2007/images/100.jpg]

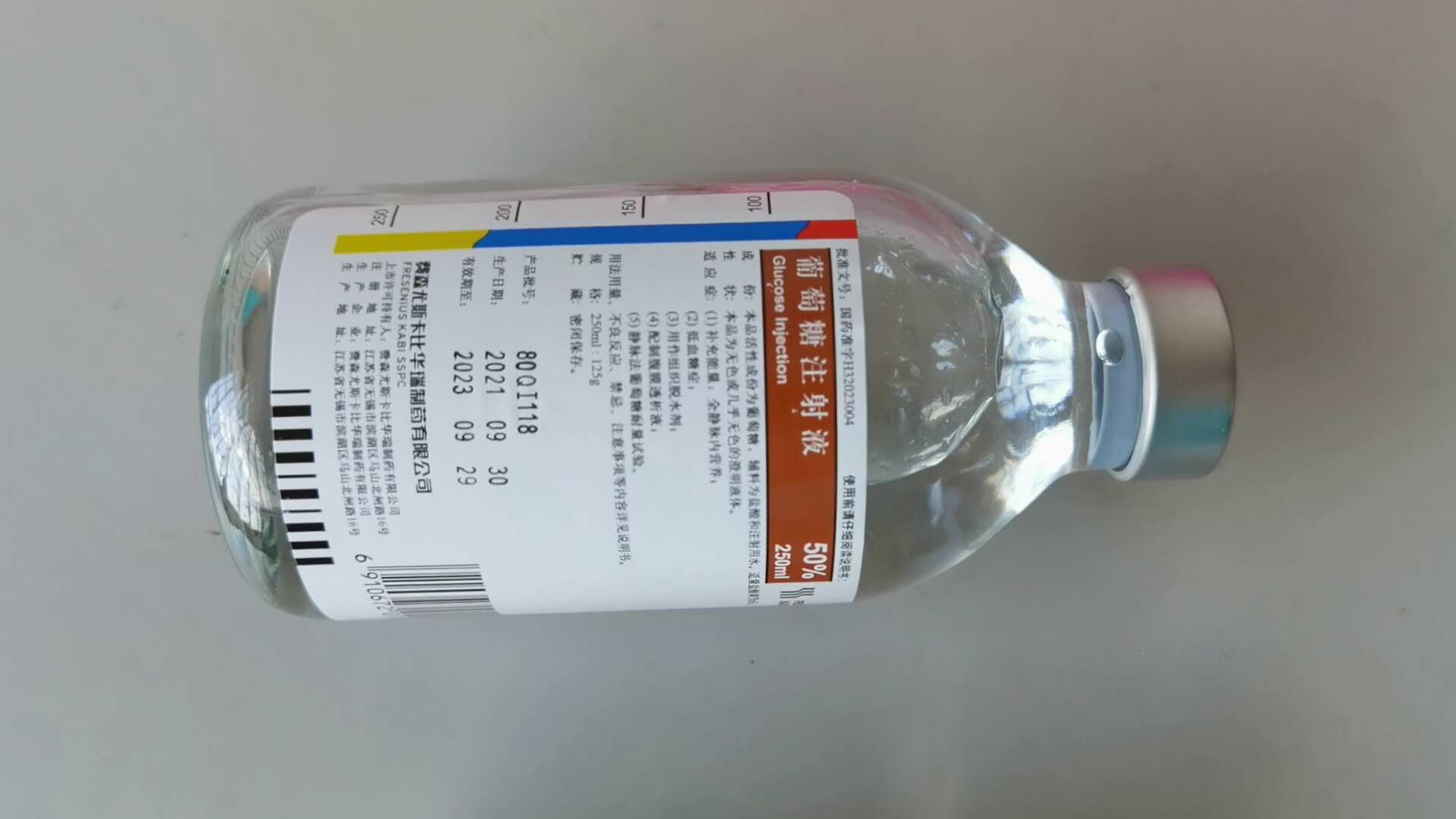

Supplement: S1 Dataset — (ZIP) [file pone.0298109.s001.zip › minimal data set/VOC2007/images/1000.jpg]

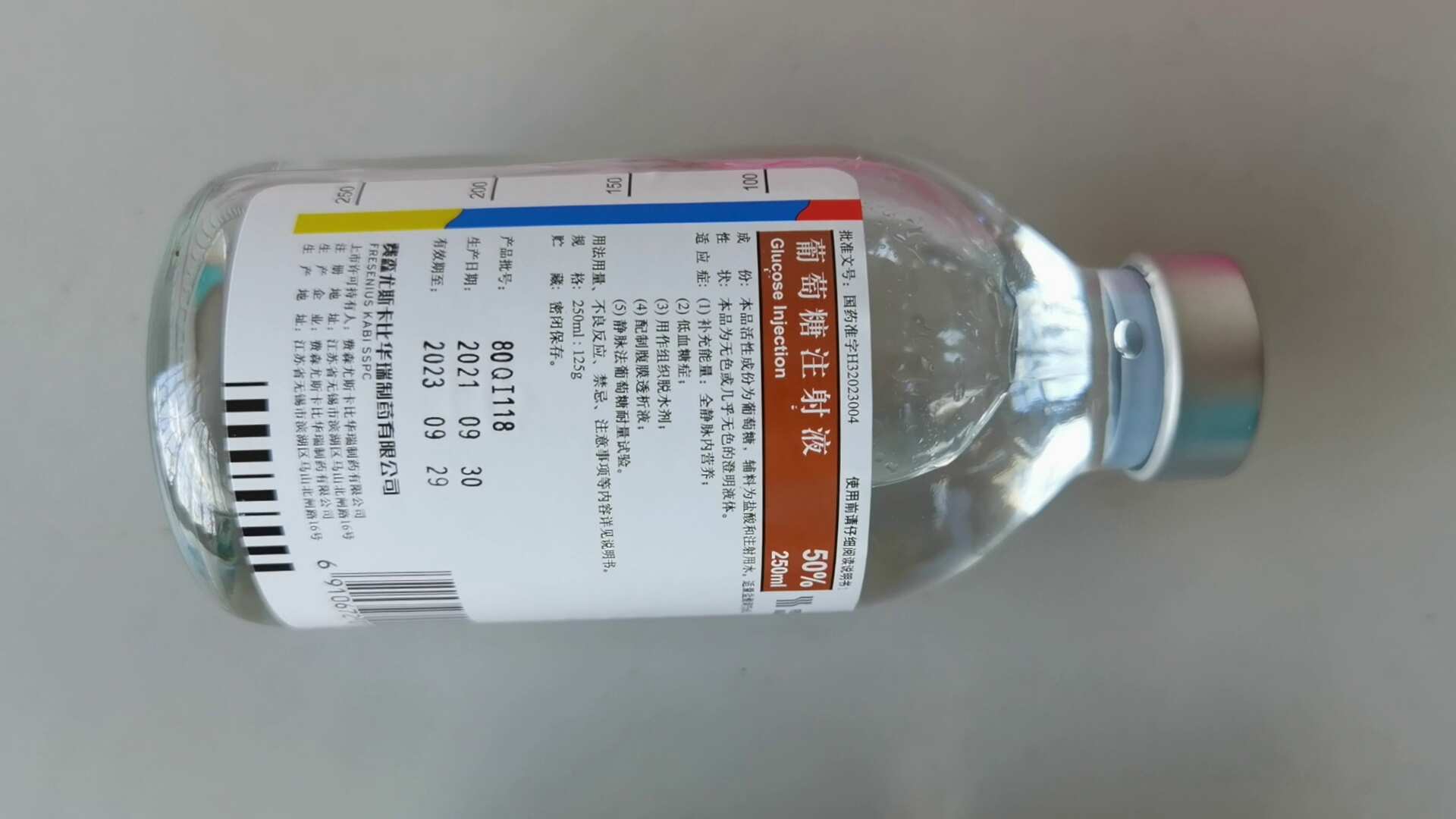

Supplement: S1 Dataset — (ZIP) [file pone.0298109.s001.zip › minimal data set/VOC2007/images/1001.jpg]

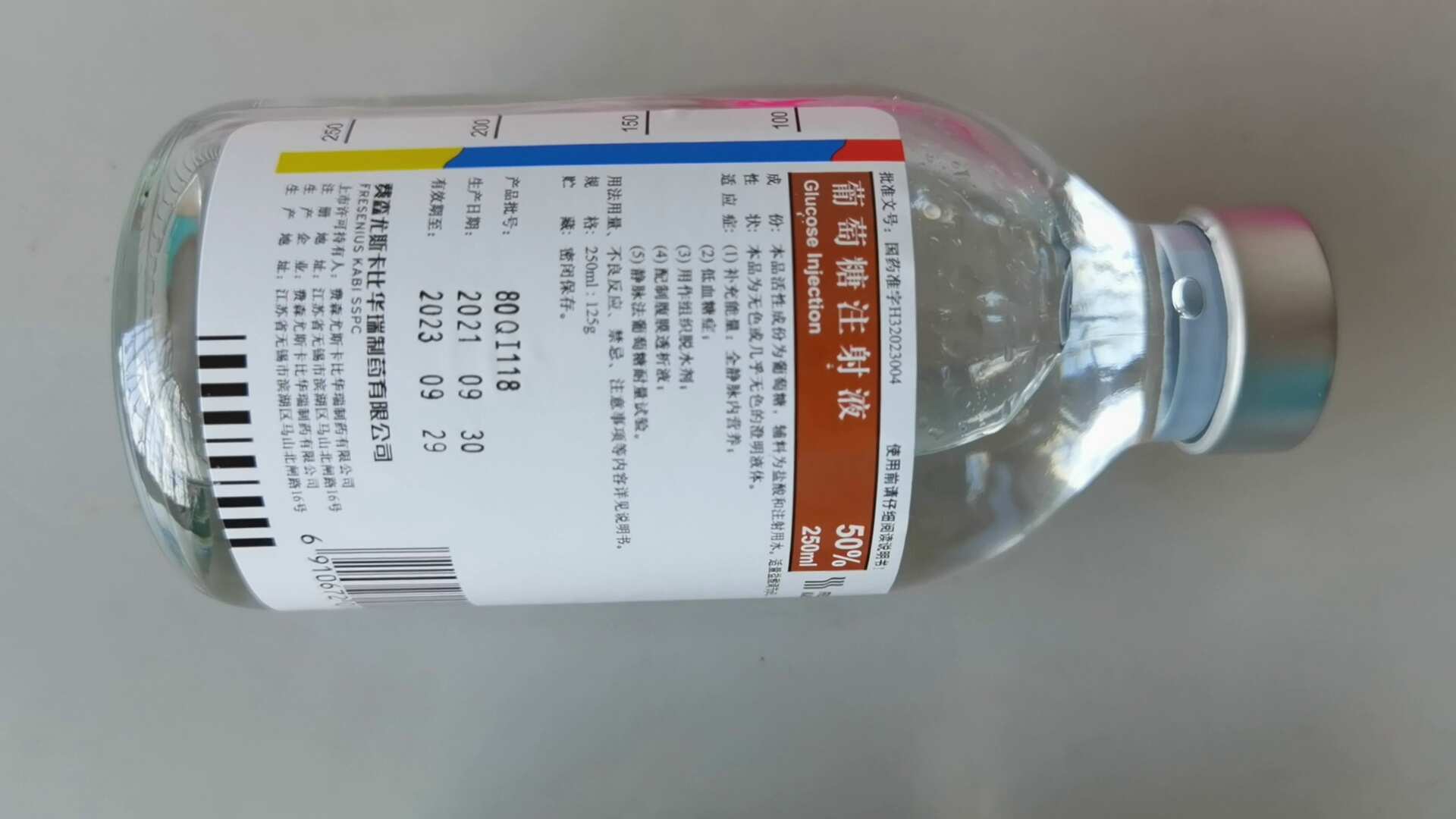

Supplement: S1 Dataset — (ZIP) [file pone.0298109.s001.zip › minimal data set/VOC2007/images/1002.jpg]

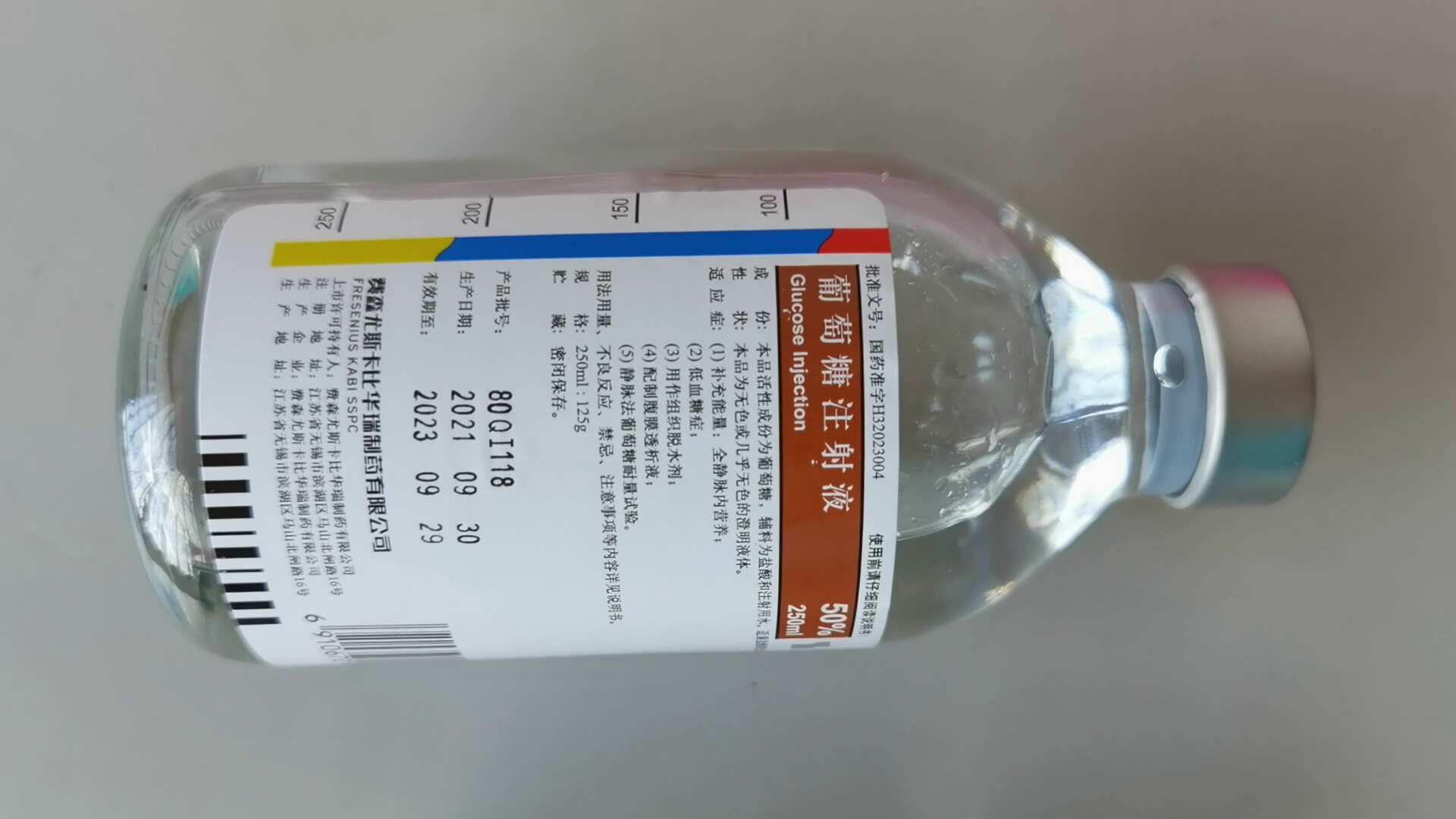

Supplement: S1 Dataset — (ZIP) [file pone.0298109.s001.zip › minimal data set/VOC2007/images/1003.jpg]

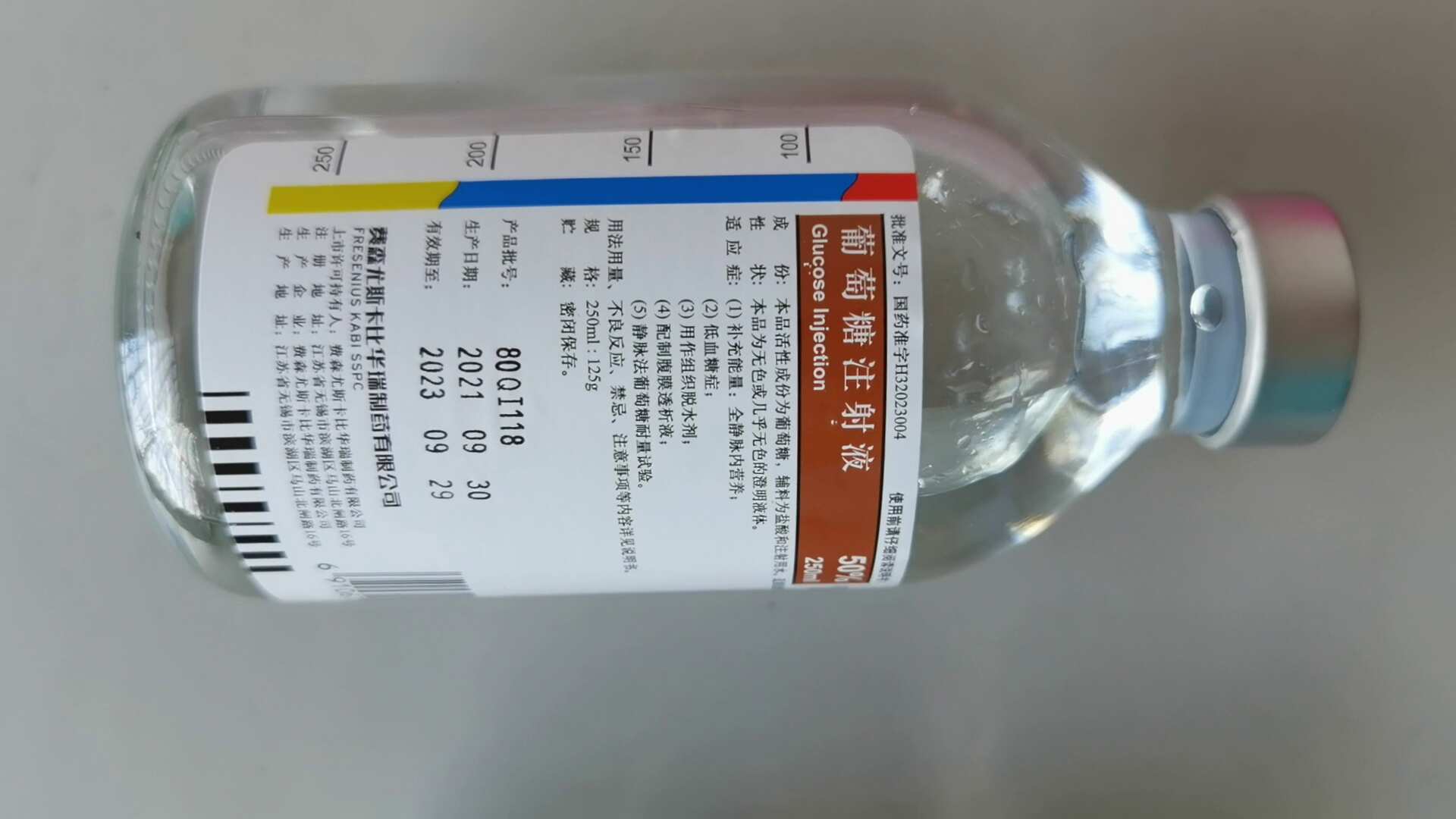

Supplement: S1 Dataset — (ZIP) [file pone.0298109.s001.zip › minimal data set/VOC2007/images/1004.jpg]

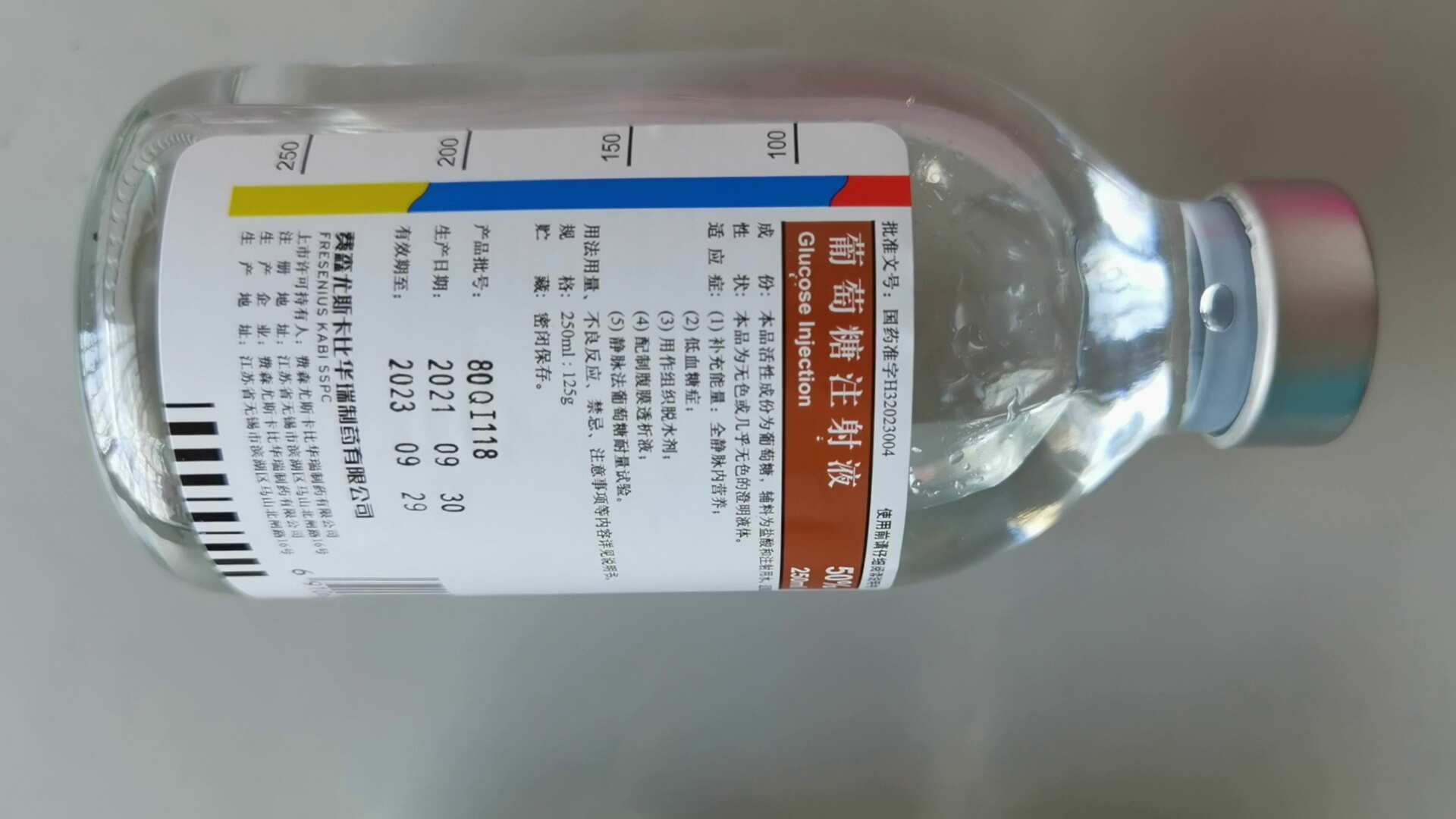

Supplement: S1 Dataset — (ZIP) [file pone.0298109.s001.zip › minimal data set/VOC2007/images/1005.jpg]

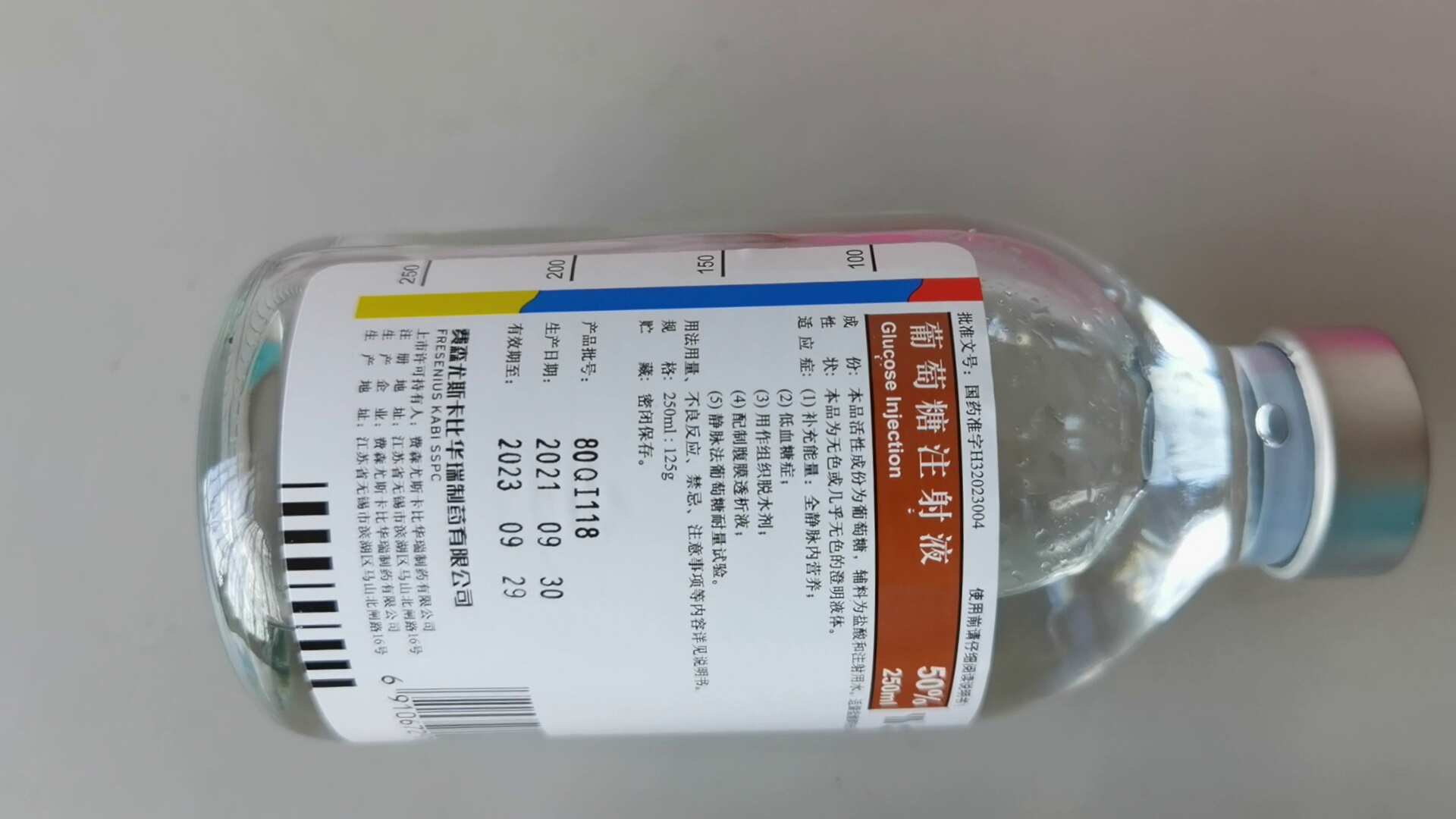

Supplement: S1 Dataset — (ZIP) [file pone.0298109.s001.zip › minimal data set/VOC2007/images/1006.jpg]

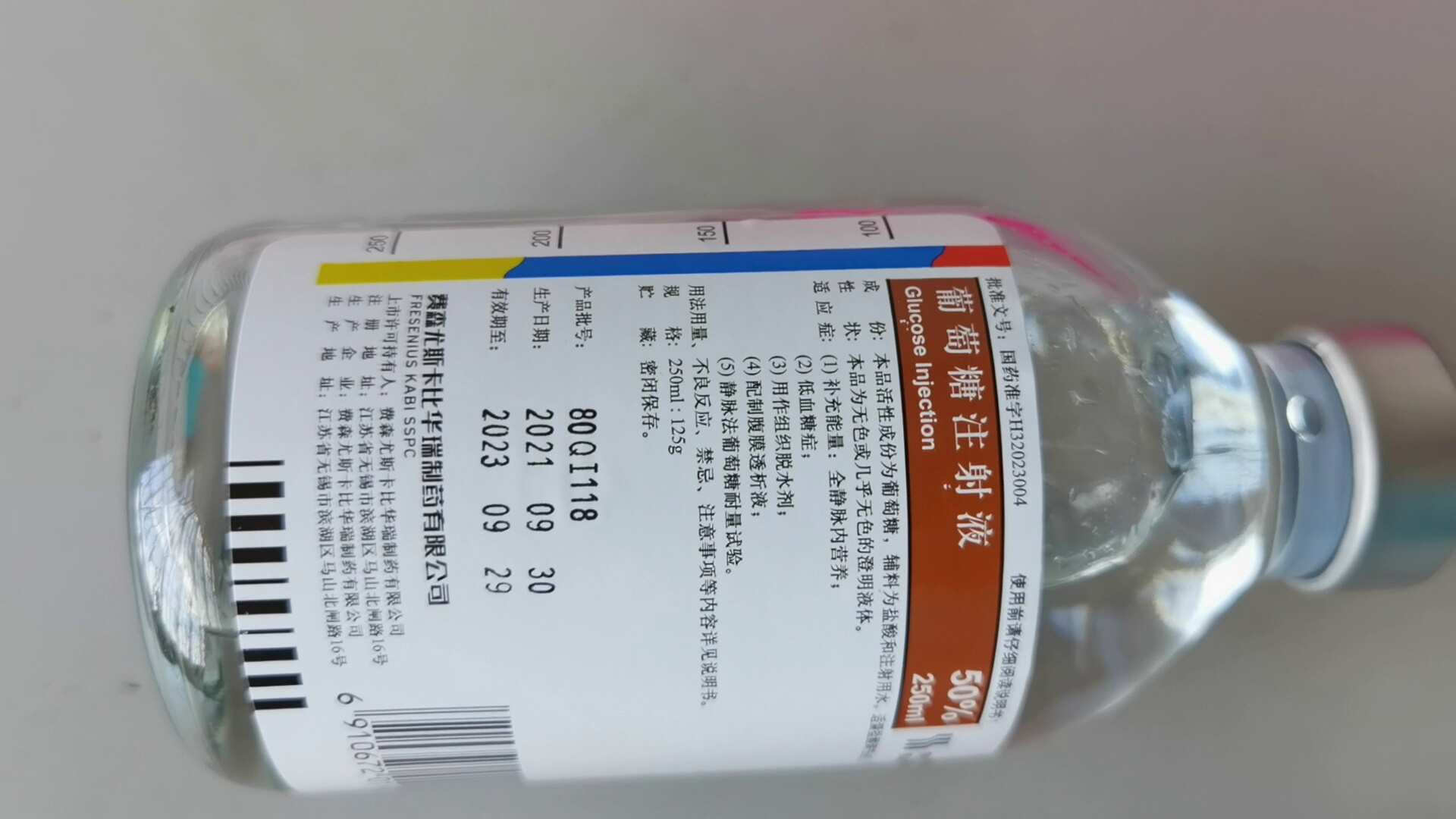

Supplement: S1 Dataset — (ZIP) [file pone.0298109.s001.zip › minimal data set/VOC2007/images/1007.jpg]

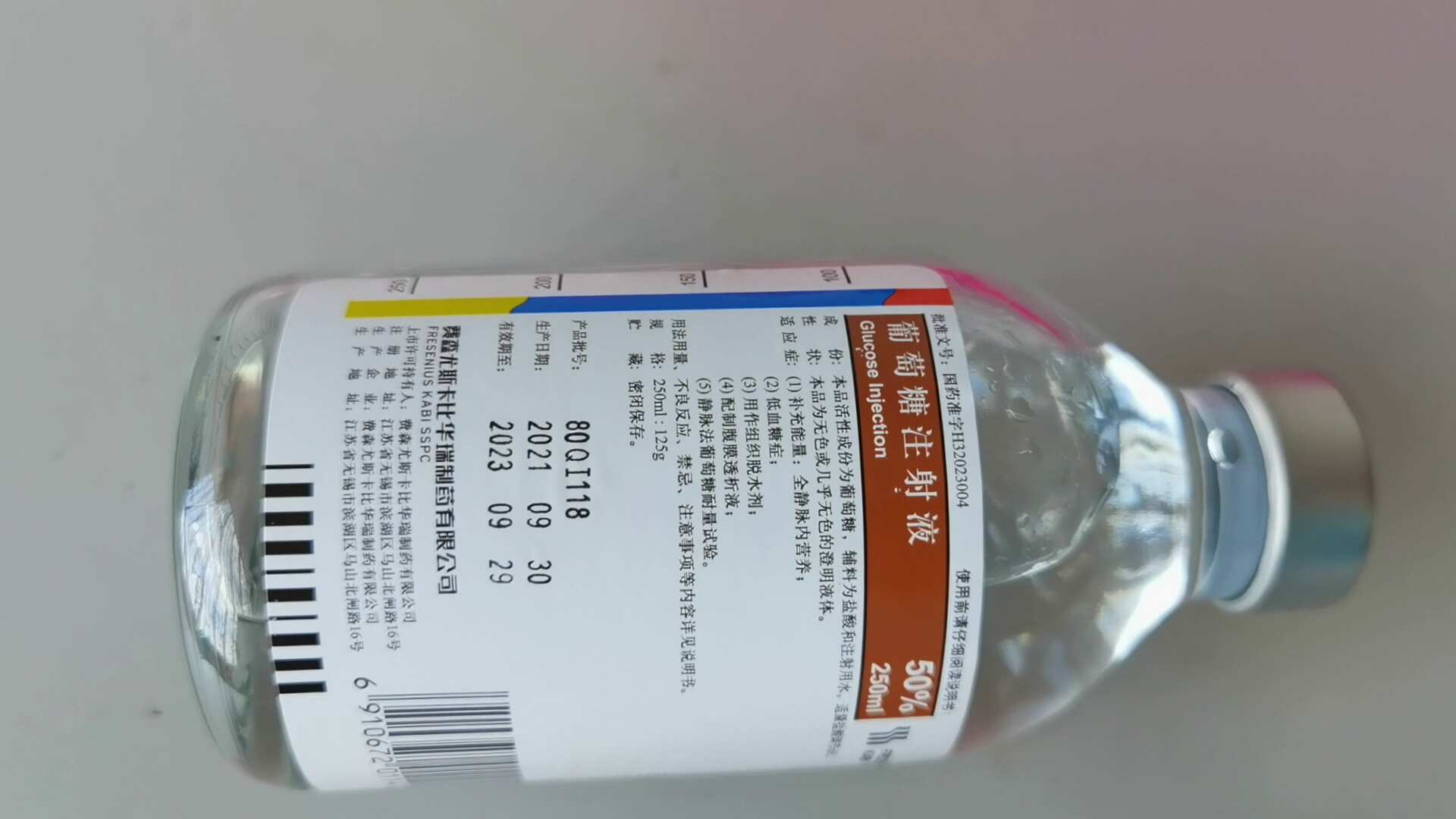

Supplement: S1 Dataset — (ZIP) [file pone.0298109.s001.zip › minimal data set/VOC2007/images/1008.jpg]

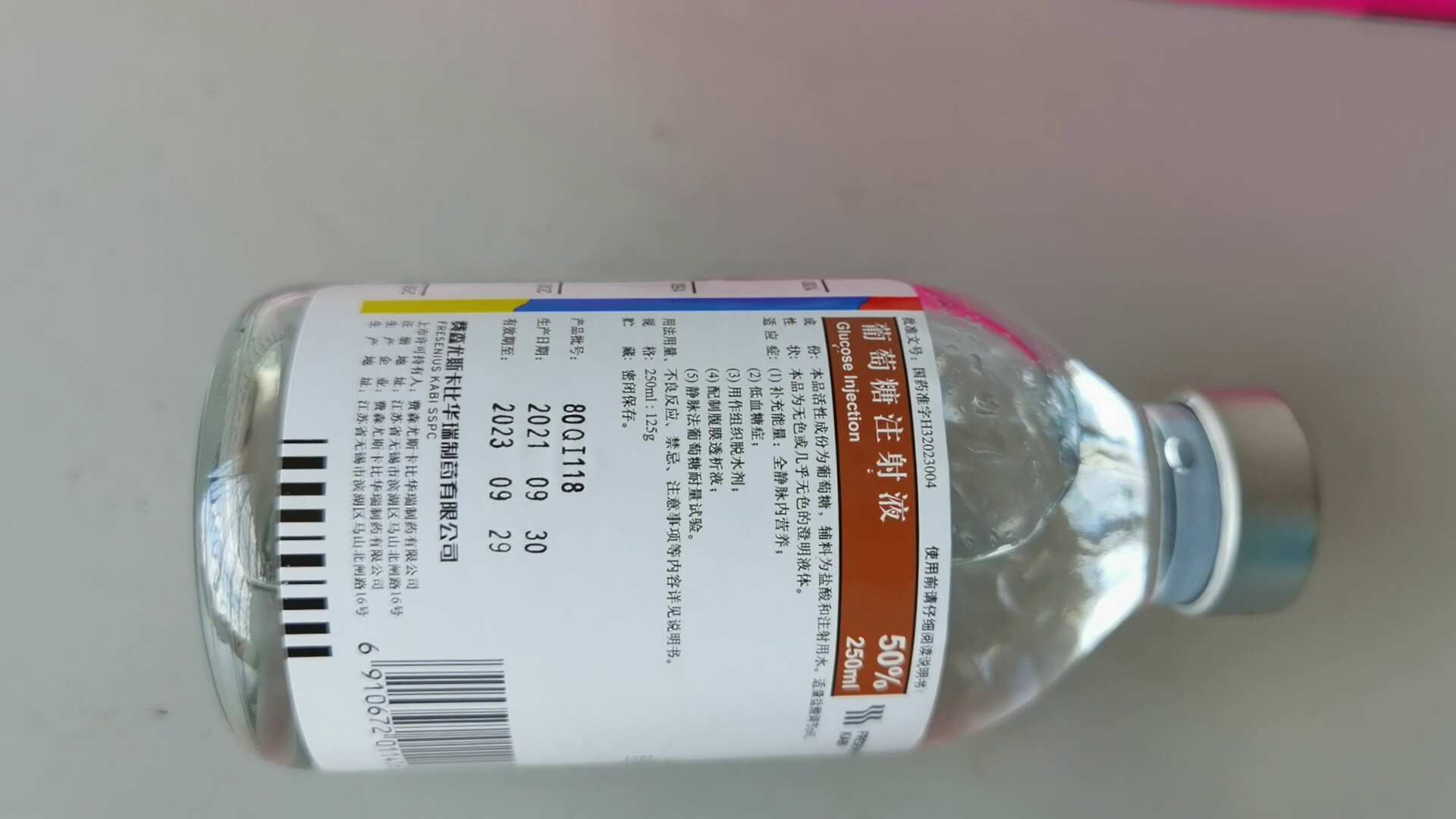

Supplement: S1 Dataset — (ZIP) [file pone.0298109.s001.zip › minimal data set/VOC2007/images/1009.jpg]

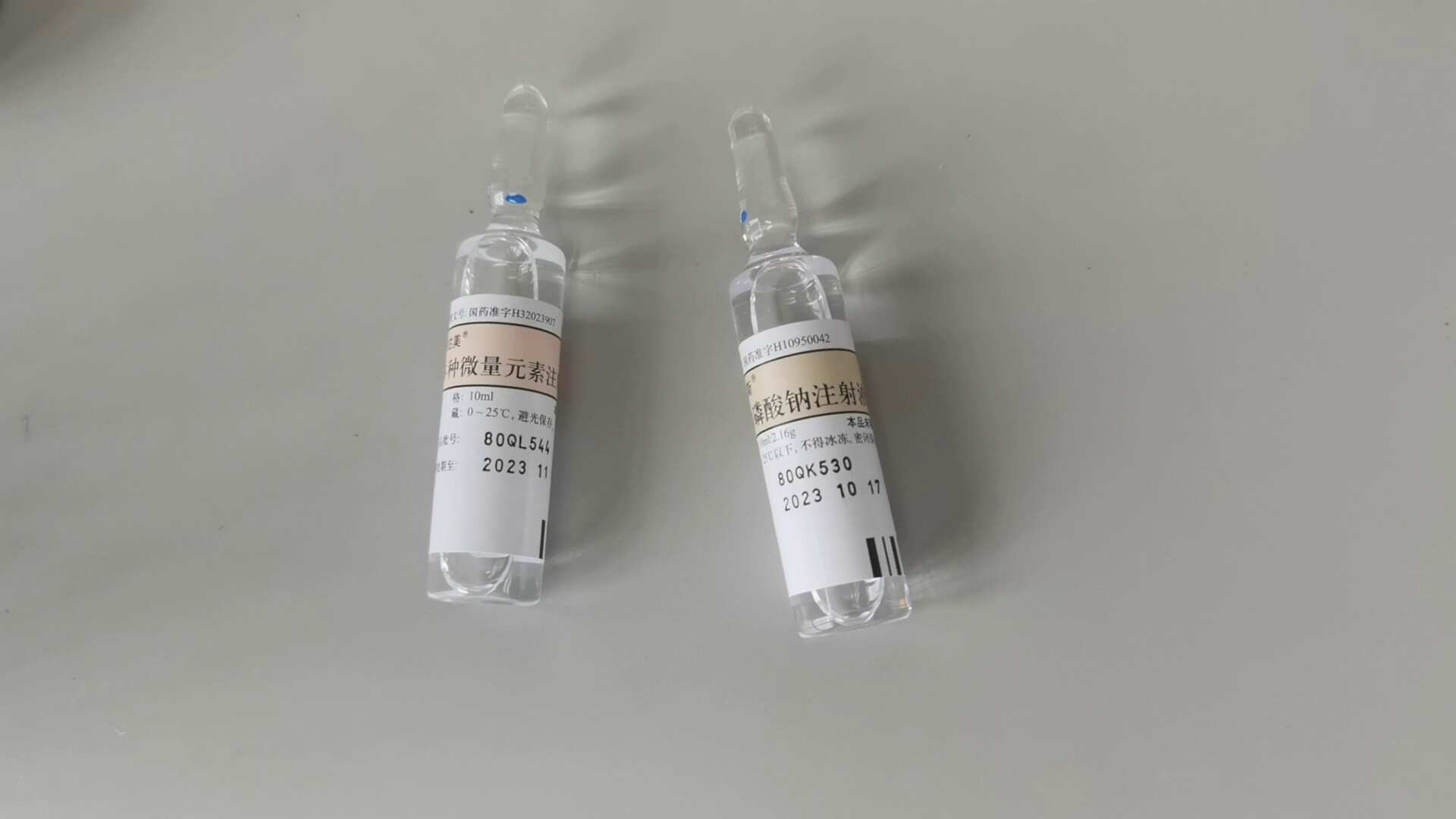

Supplement: S1 Dataset — (ZIP) [file pone.0298109.s001.zip › minimal data set/VOC2007/images/101.jpg]

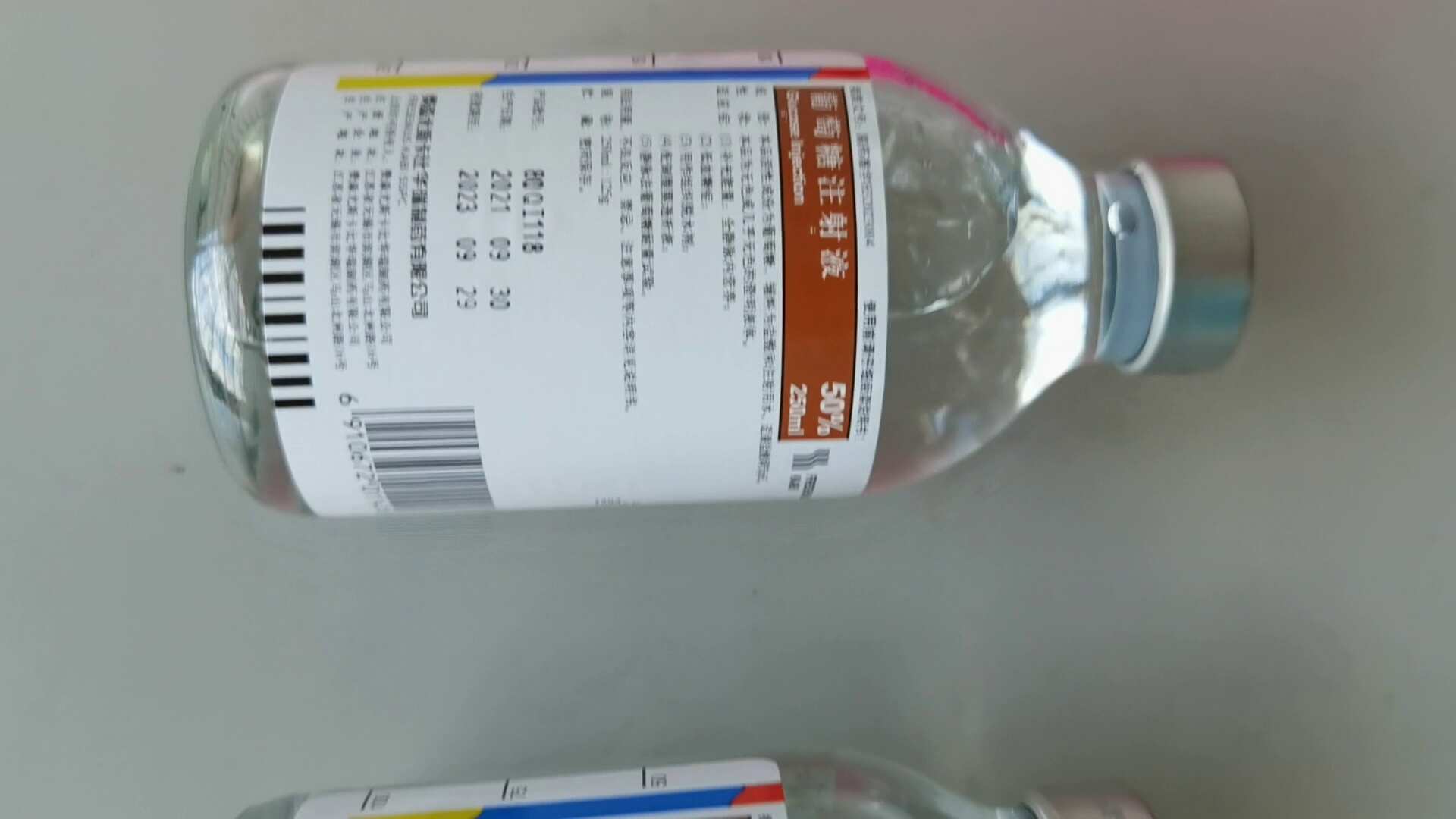

Supplement: S1 Dataset — (ZIP) [file pone.0298109.s001.zip › minimal data set/VOC2007/images/1010.jpg]

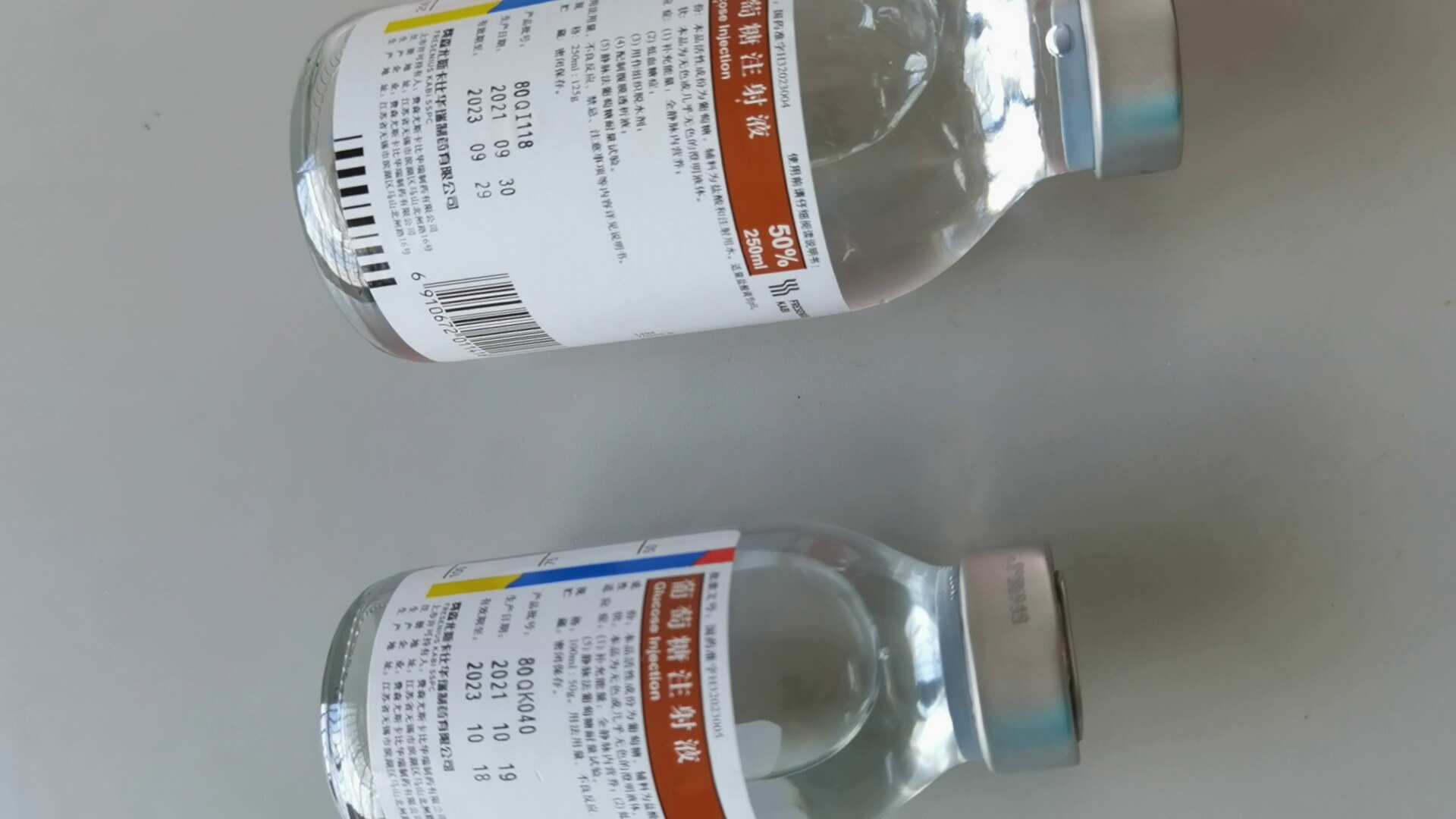

Supplement: S1 Dataset — (ZIP) [file pone.0298109.s001.zip › minimal data set/VOC2007/images/1011.jpg]

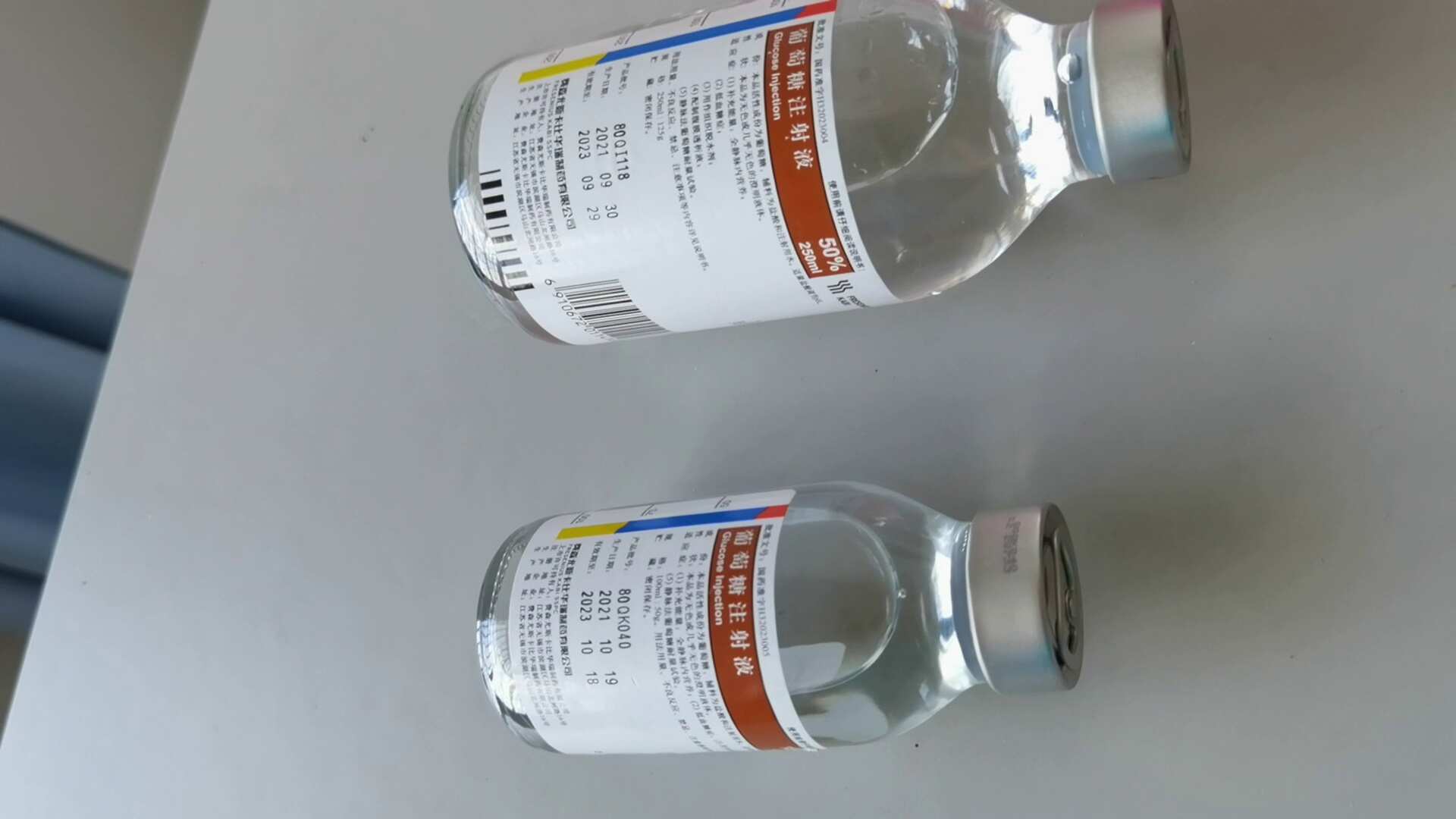

Supplement: S1 Dataset — (ZIP) [file pone.0298109.s001.zip › minimal data set/VOC2007/images/1012.jpg]

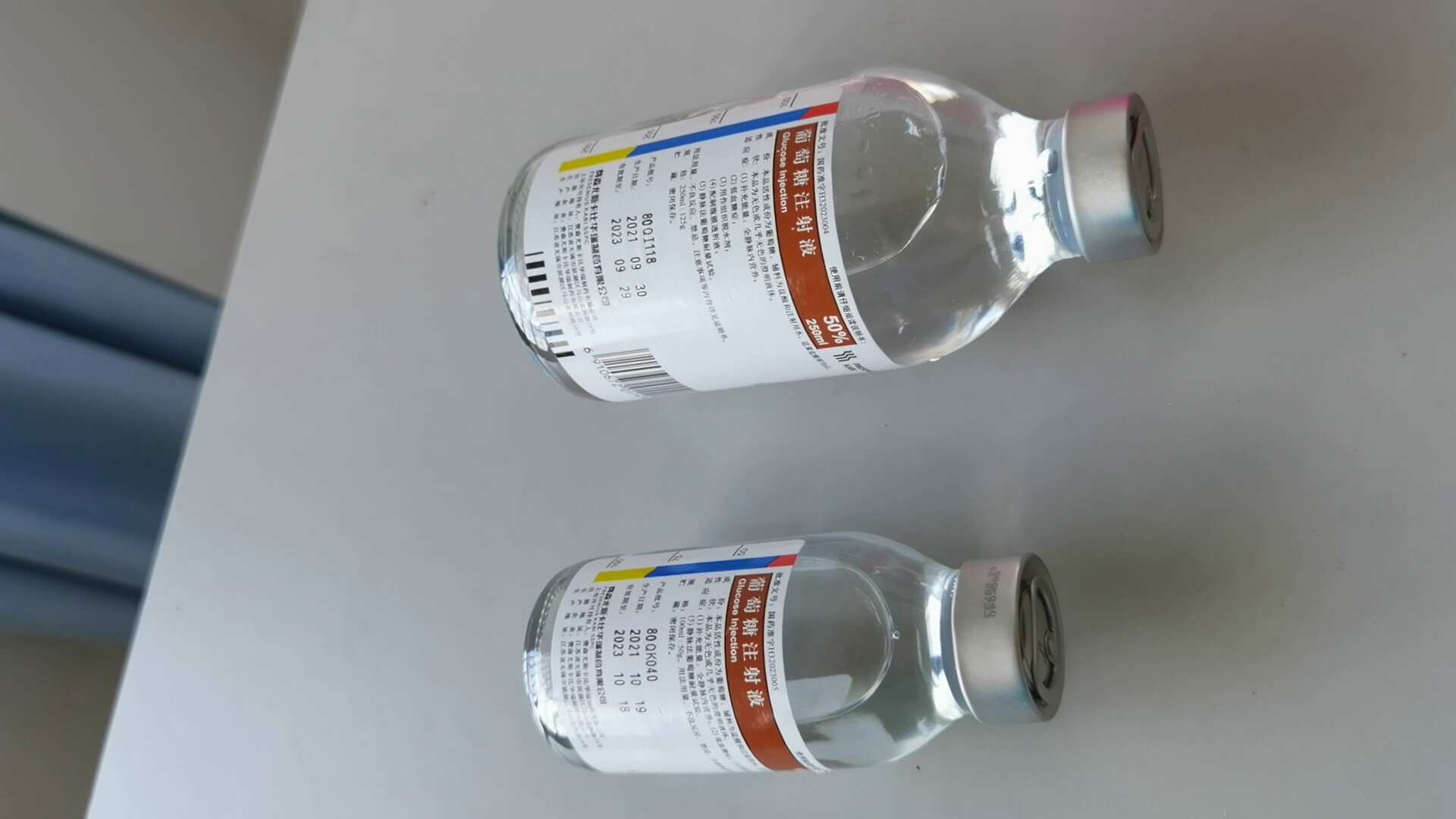

Supplement: S1 Dataset — (ZIP) [file pone.0298109.s001.zip › minimal data set/VOC2007/images/1013.jpg]

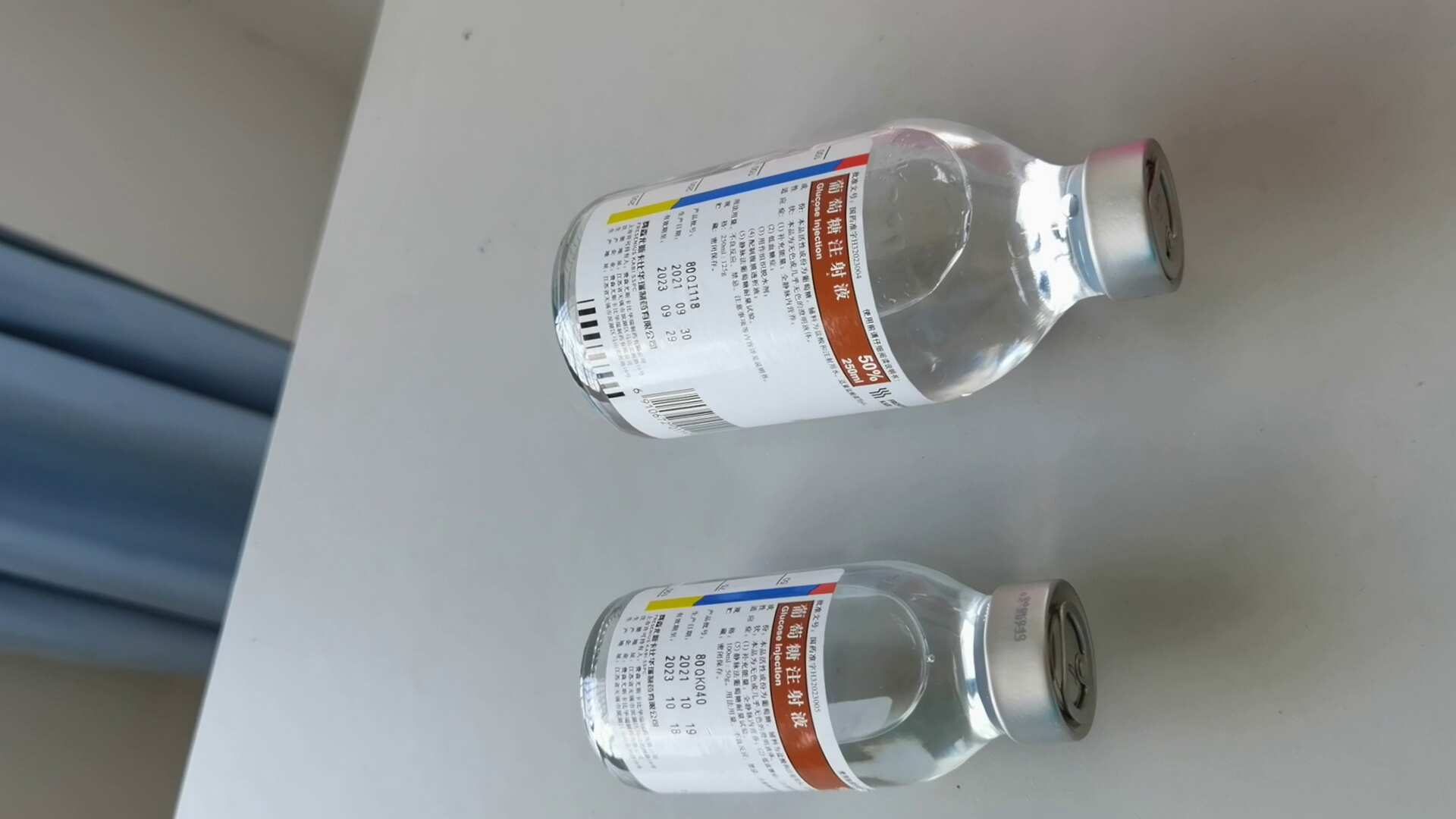

Supplement: S1 Dataset — (ZIP) [file pone.0298109.s001.zip › minimal data set/VOC2007/images/1014.jpg]

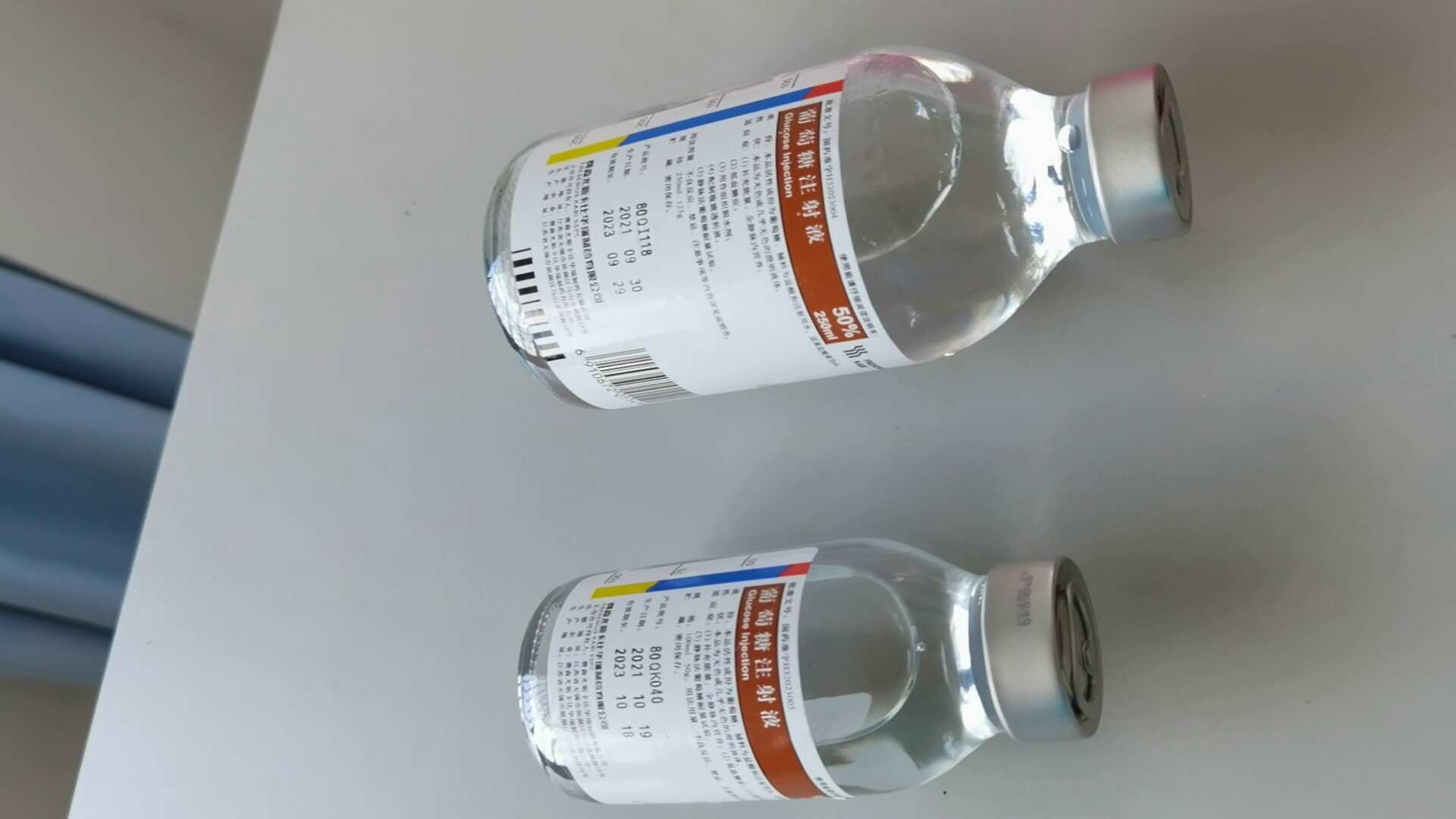

Supplement: S1 Dataset — (ZIP) [file pone.0298109.s001.zip › minimal data set/VOC2007/images/1015.jpg]

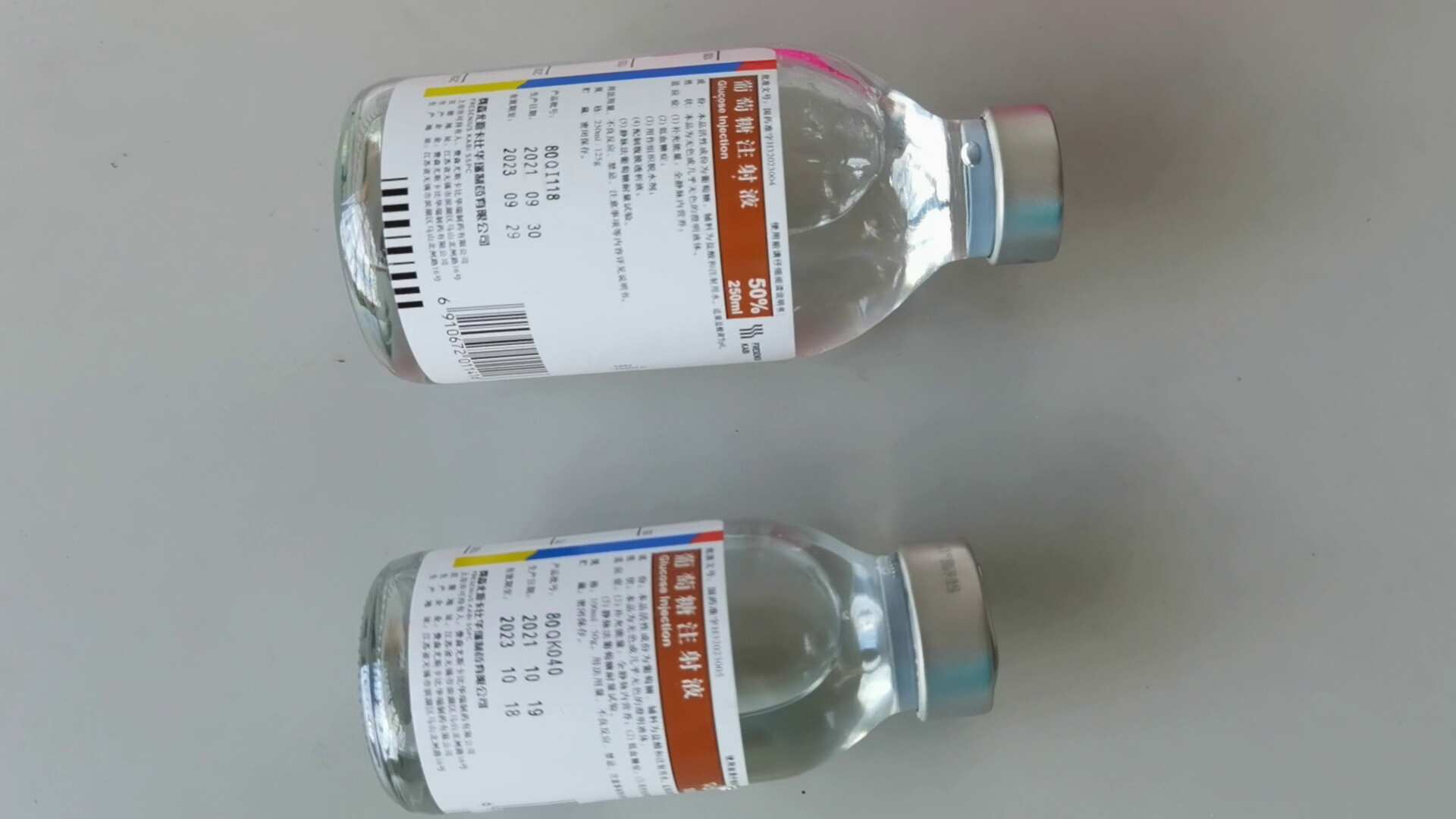

Supplement: S1 Dataset — (ZIP) [file pone.0298109.s001.zip › minimal data set/VOC2007/images/1016.jpg]

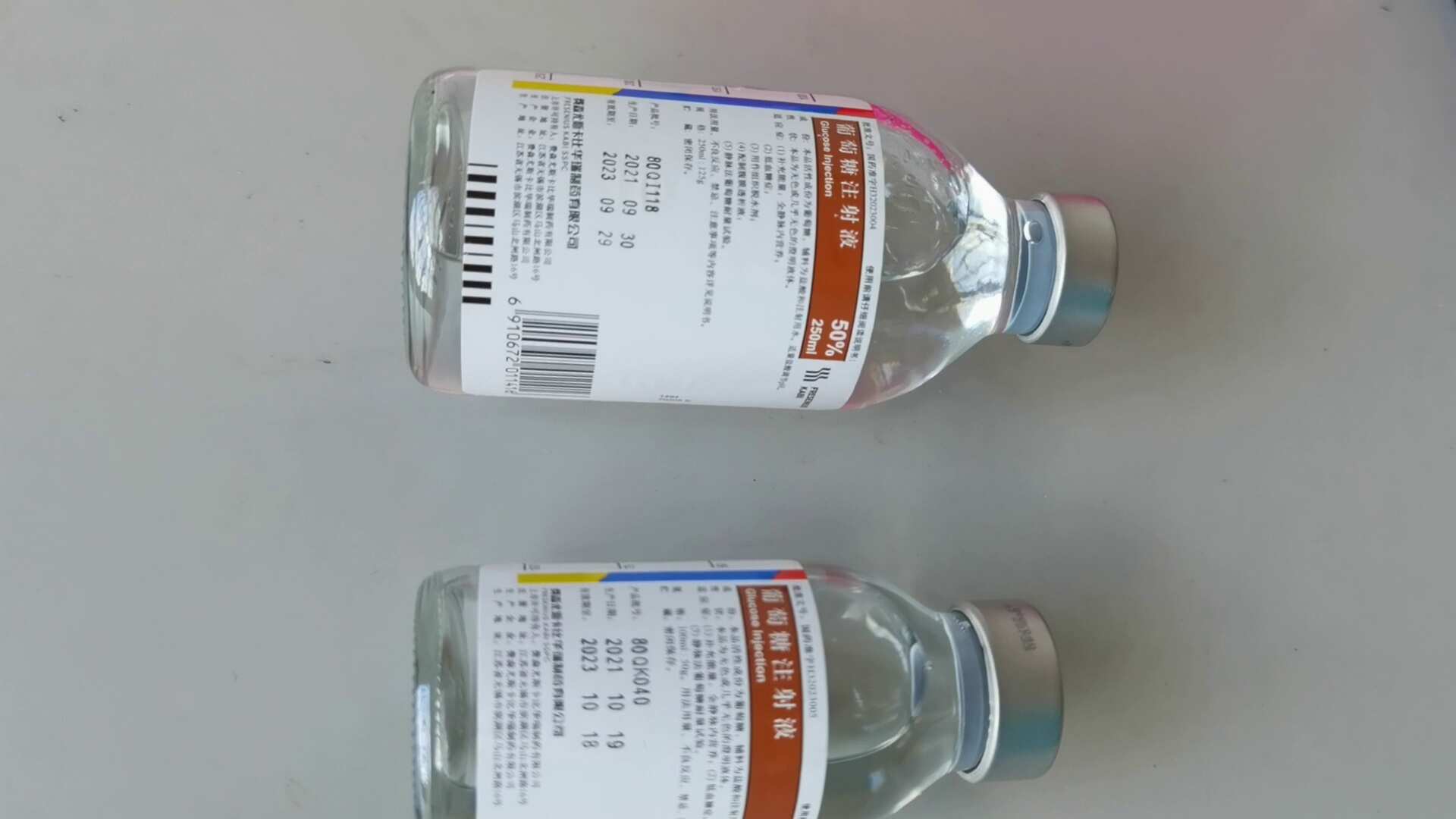

Supplement: S1 Dataset — (ZIP) [file pone.0298109.s001.zip › minimal data set/VOC2007/images/1017.jpg]

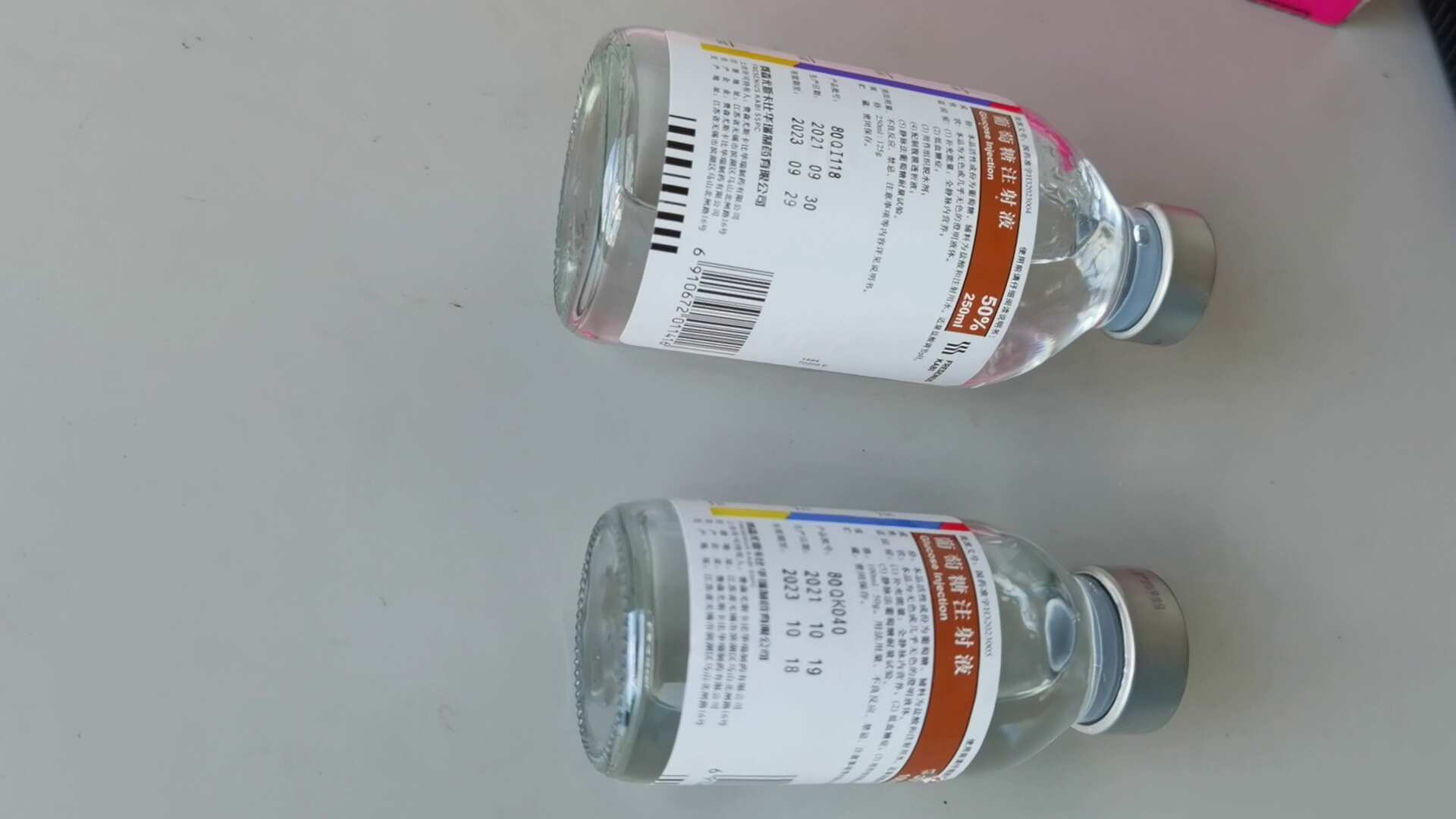

Supplement: S1 Dataset — (ZIP) [file pone.0298109.s001.zip › minimal data set/VOC2007/images/1018.jpg]

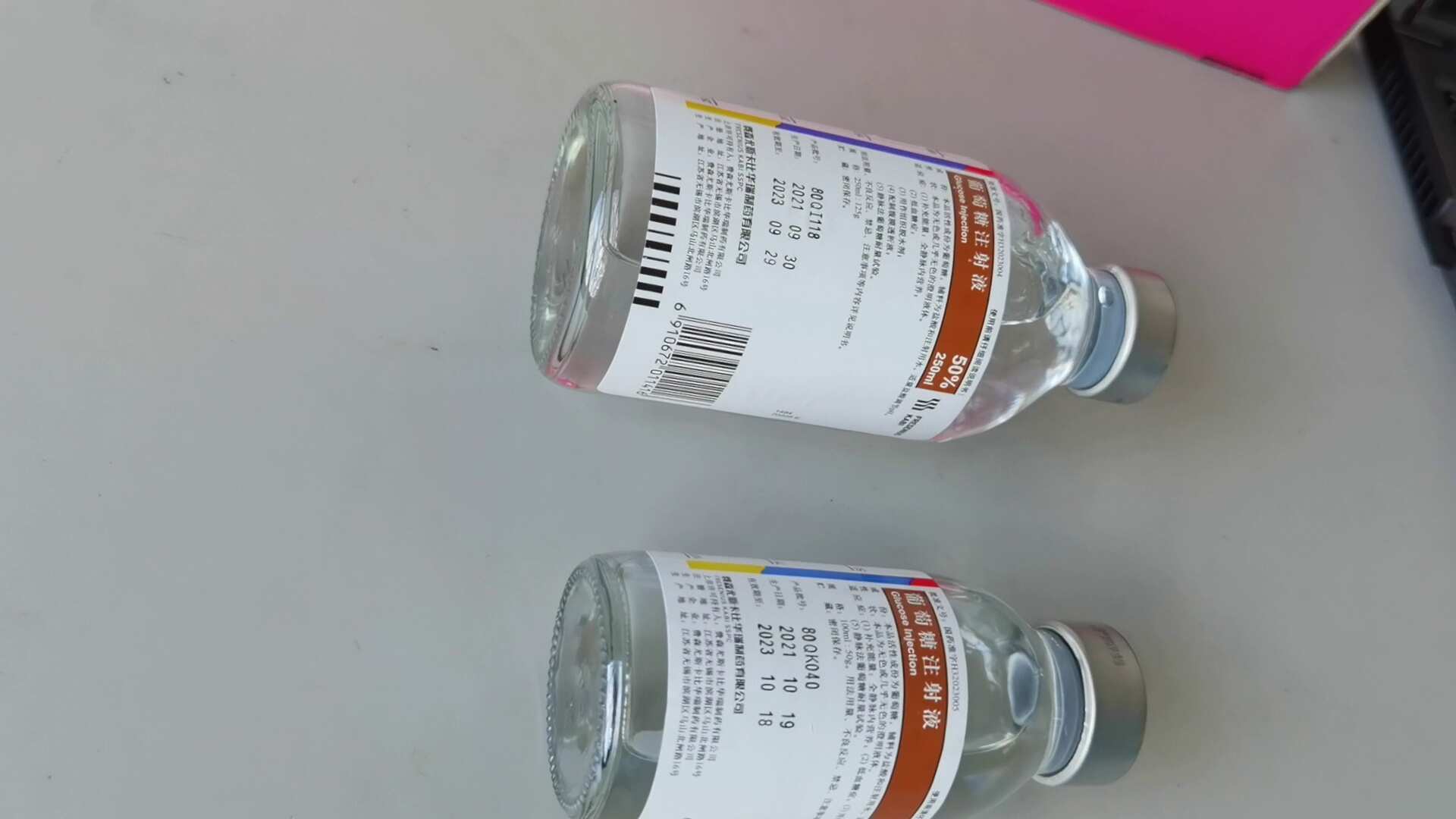

Supplement: S1 Dataset — (ZIP) [file pone.0298109.s001.zip › minimal data set/VOC2007/images/1019.jpg]

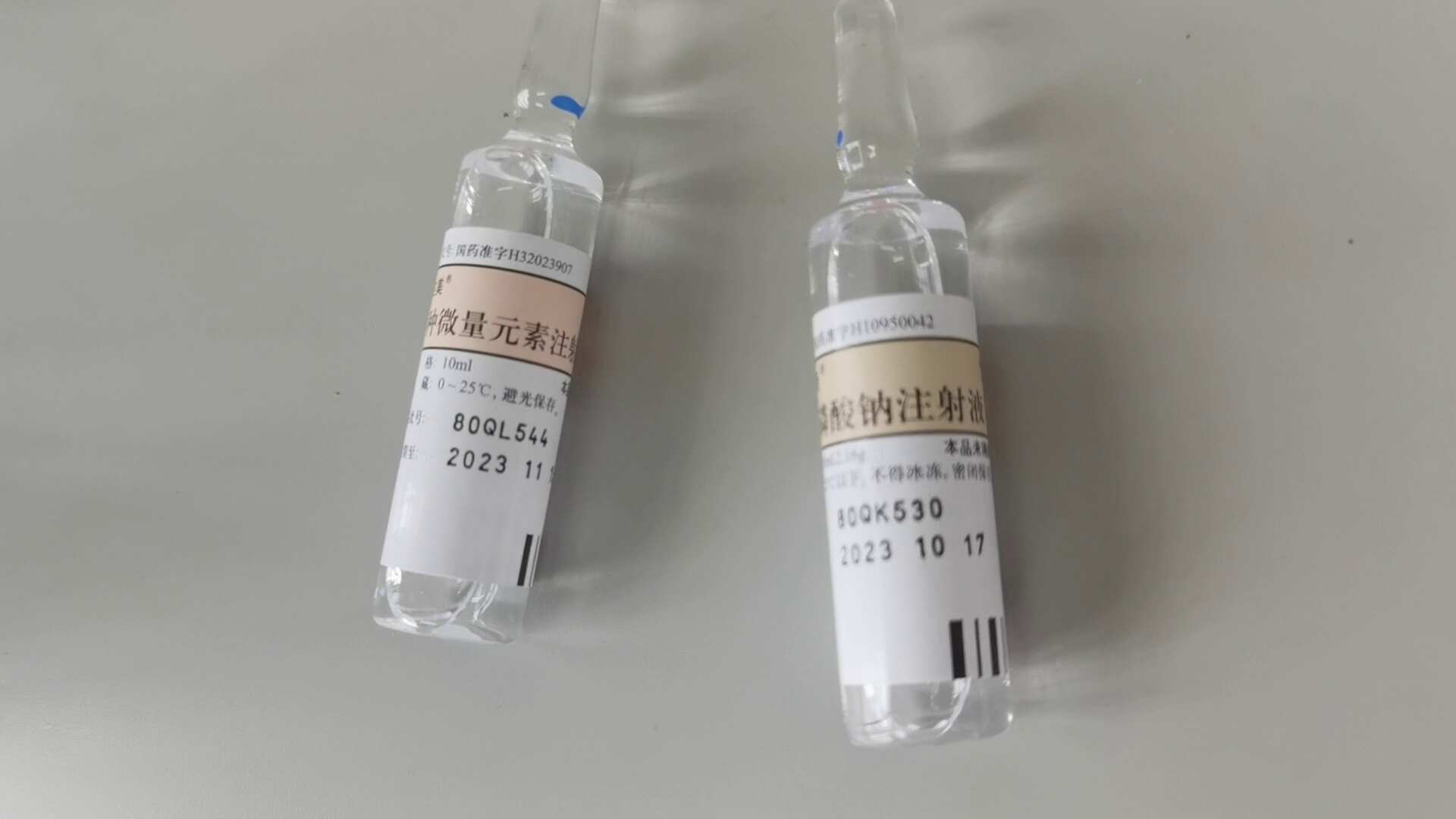

Supplement: S1 Dataset — (ZIP) [file pone.0298109.s001.zip › minimal data set/VOC2007/images/102.jpg]

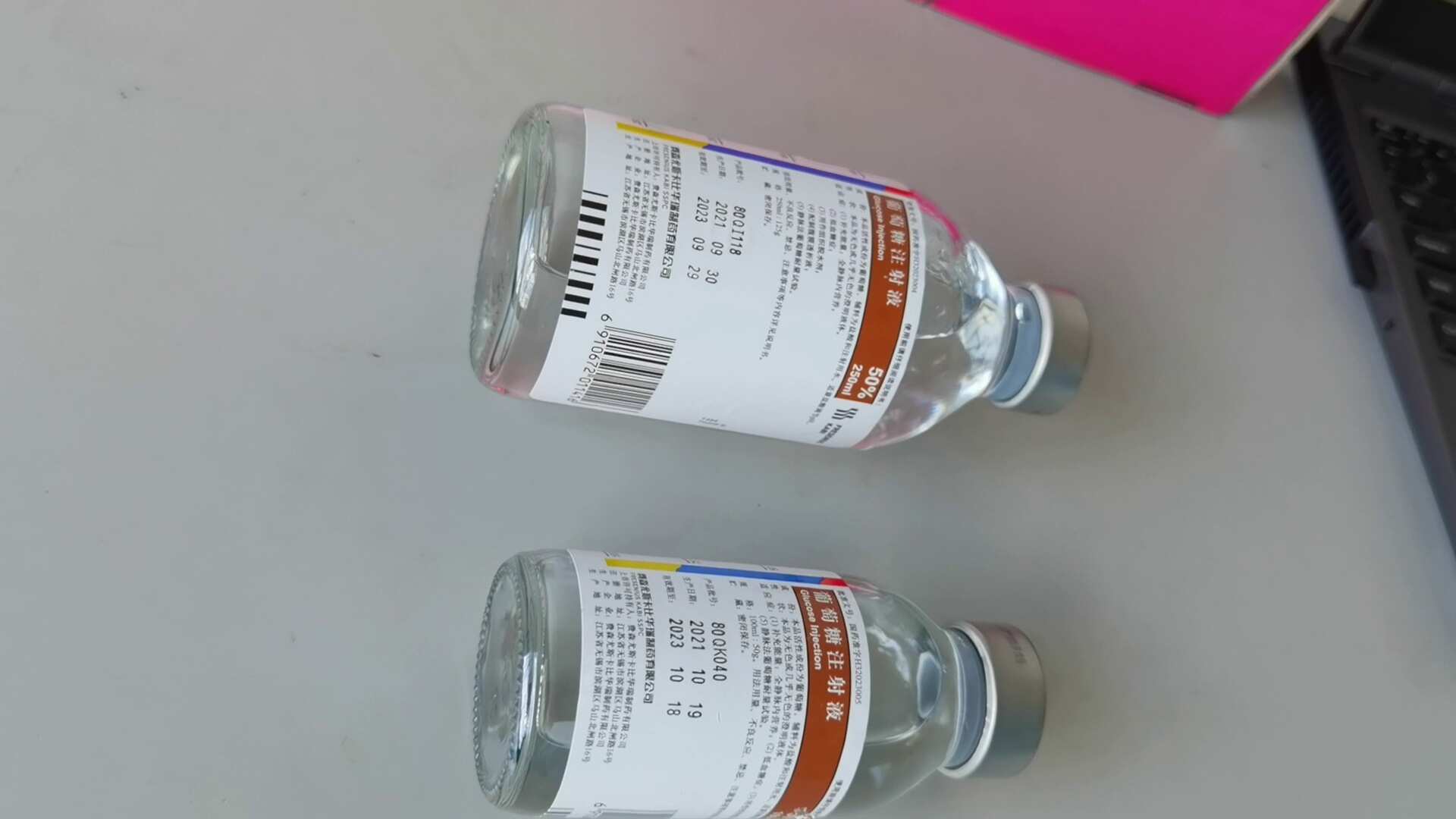

Supplement: S1 Dataset — (ZIP) [file pone.0298109.s001.zip › minimal data set/VOC2007/images/1020.jpg]

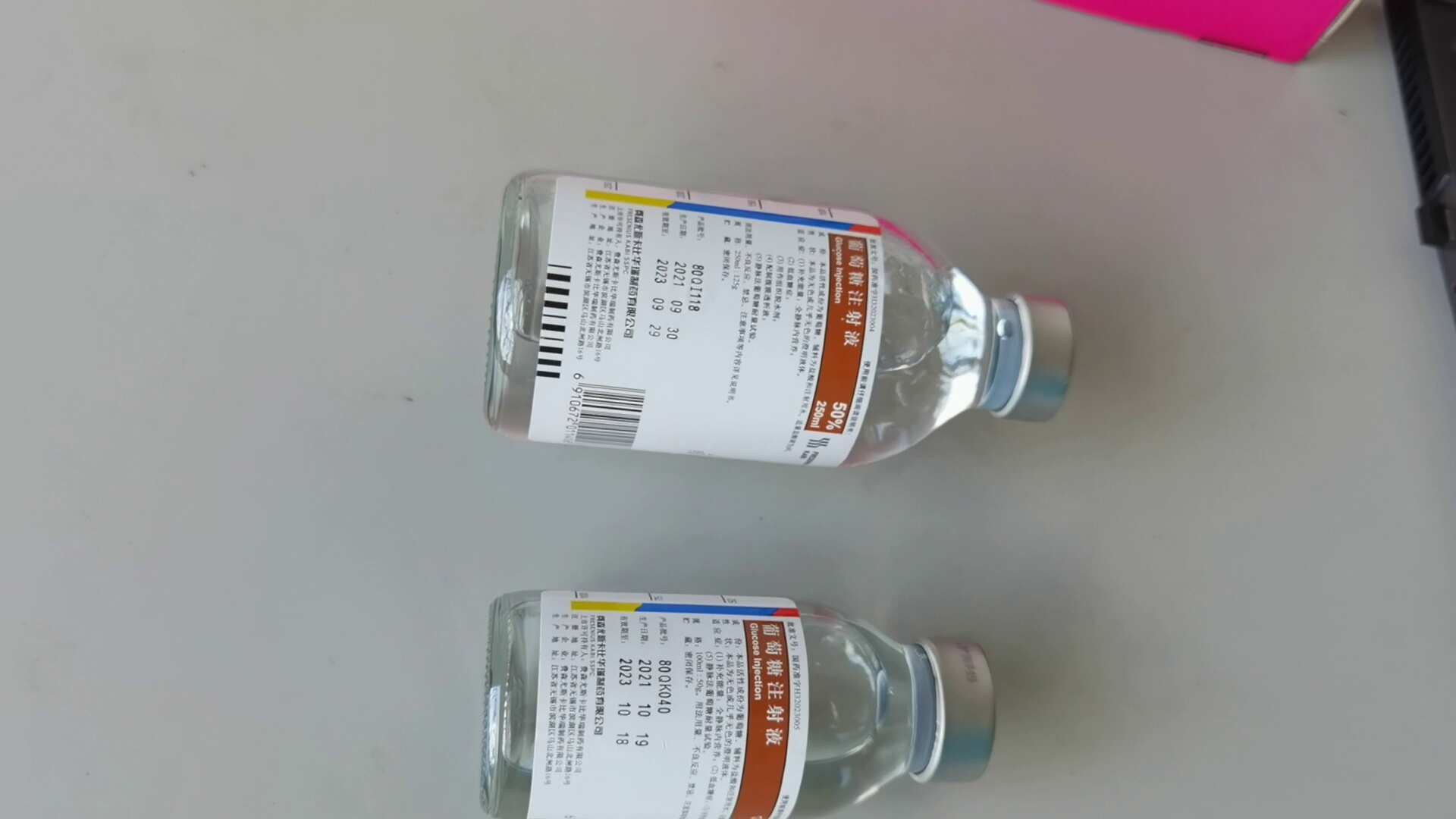

Supplement: S1 Dataset — (ZIP) [file pone.0298109.s001.zip › minimal data set/VOC2007/images/1021.jpg]

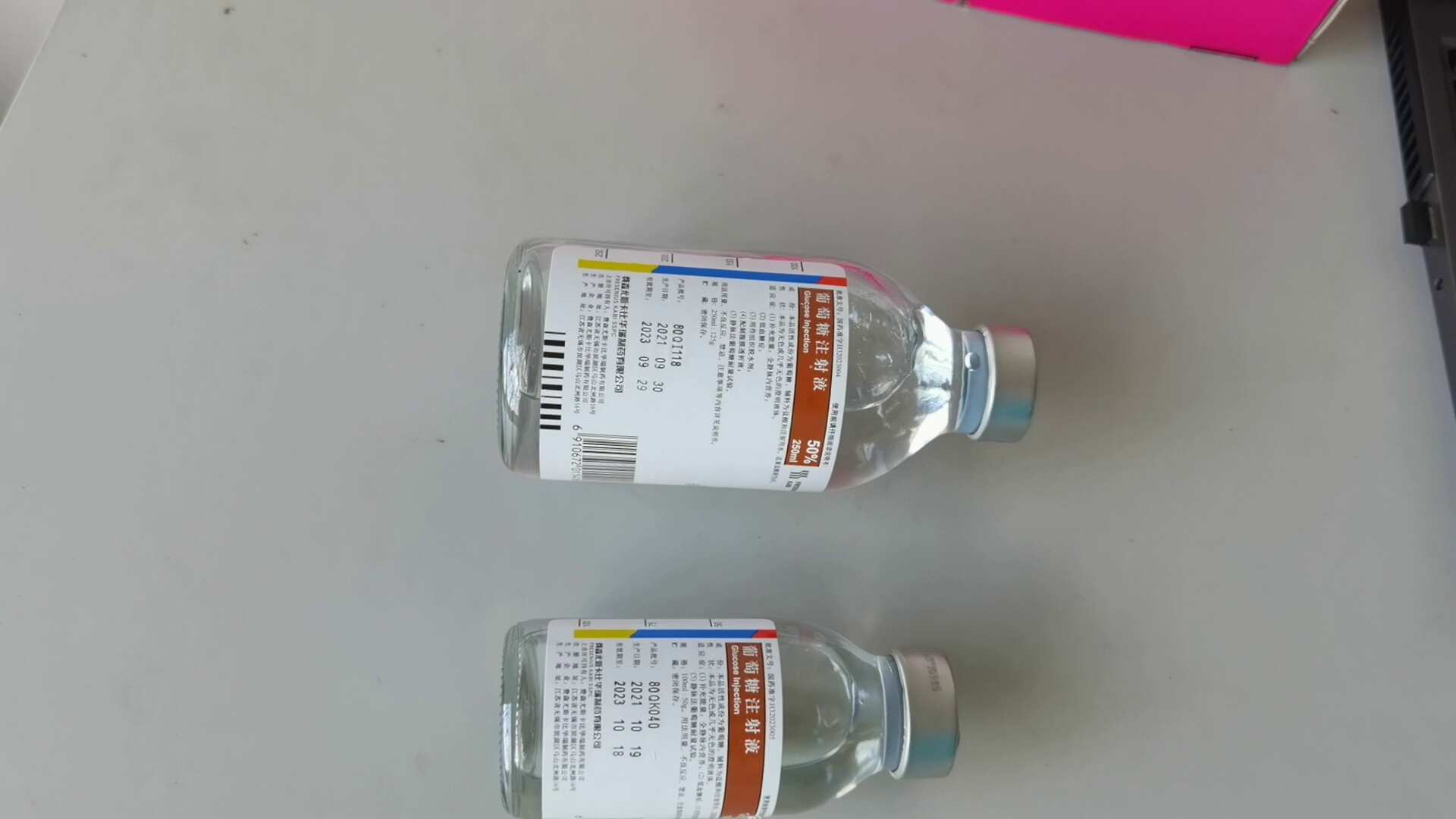

Supplement: S1 Dataset — (ZIP) [file pone.0298109.s001.zip › minimal data set/VOC2007/images/1022.jpg]

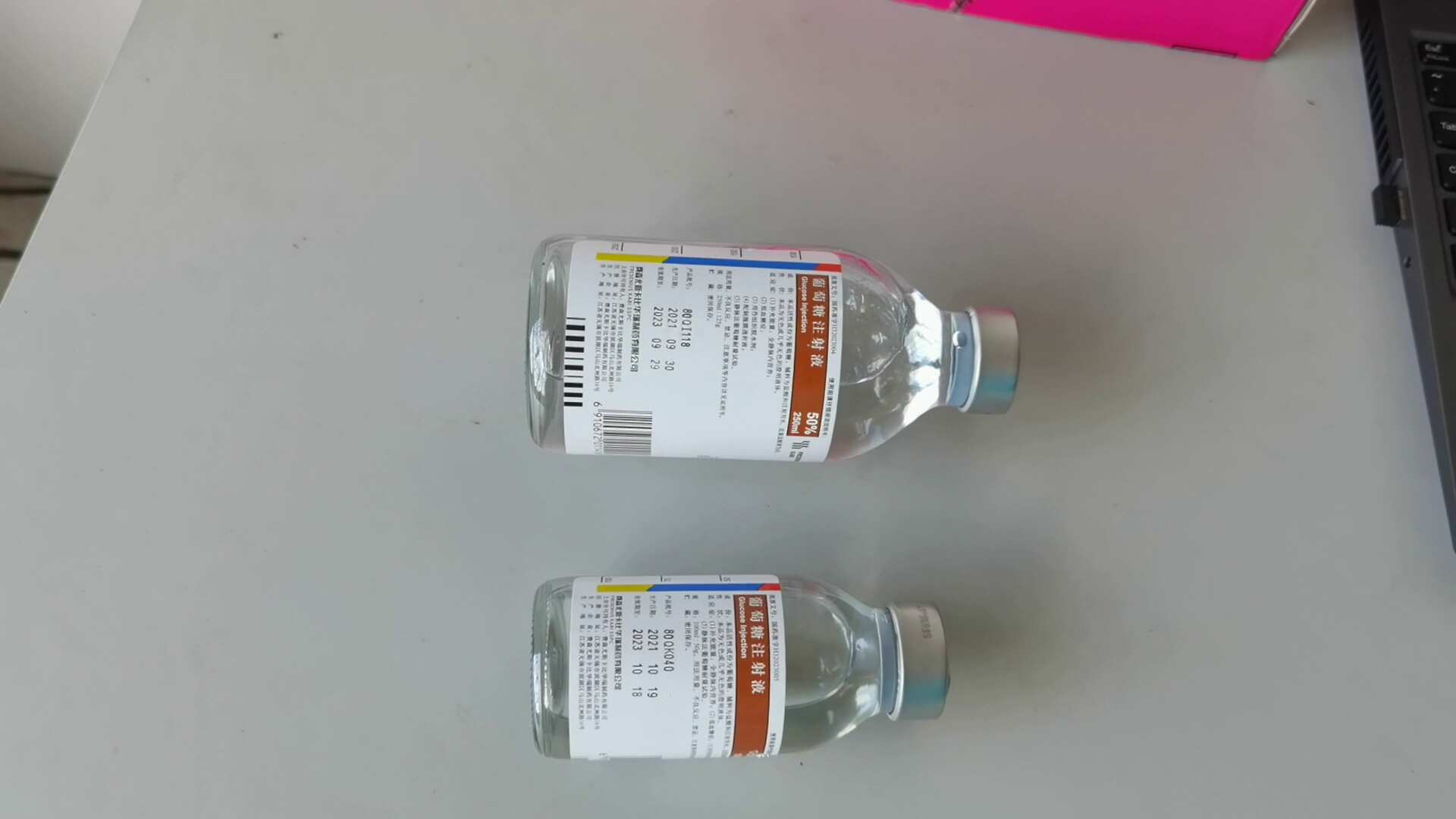

Supplement: S1 Dataset — (ZIP) [file pone.0298109.s001.zip › minimal data set/VOC2007/images/1023.jpg]

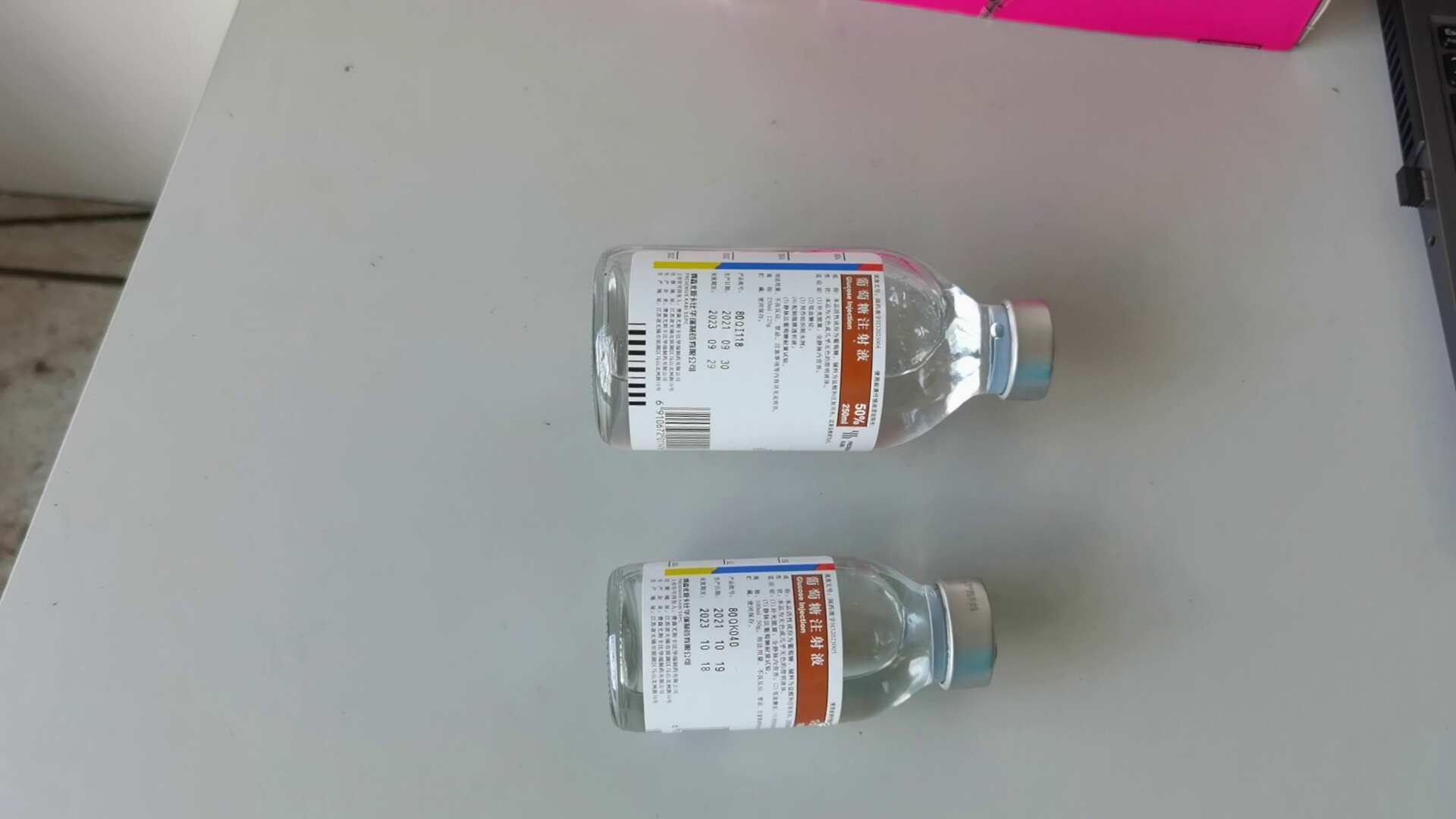

Supplement: S1 Dataset — (ZIP) [file pone.0298109.s001.zip › minimal data set/VOC2007/images/1024.jpg]

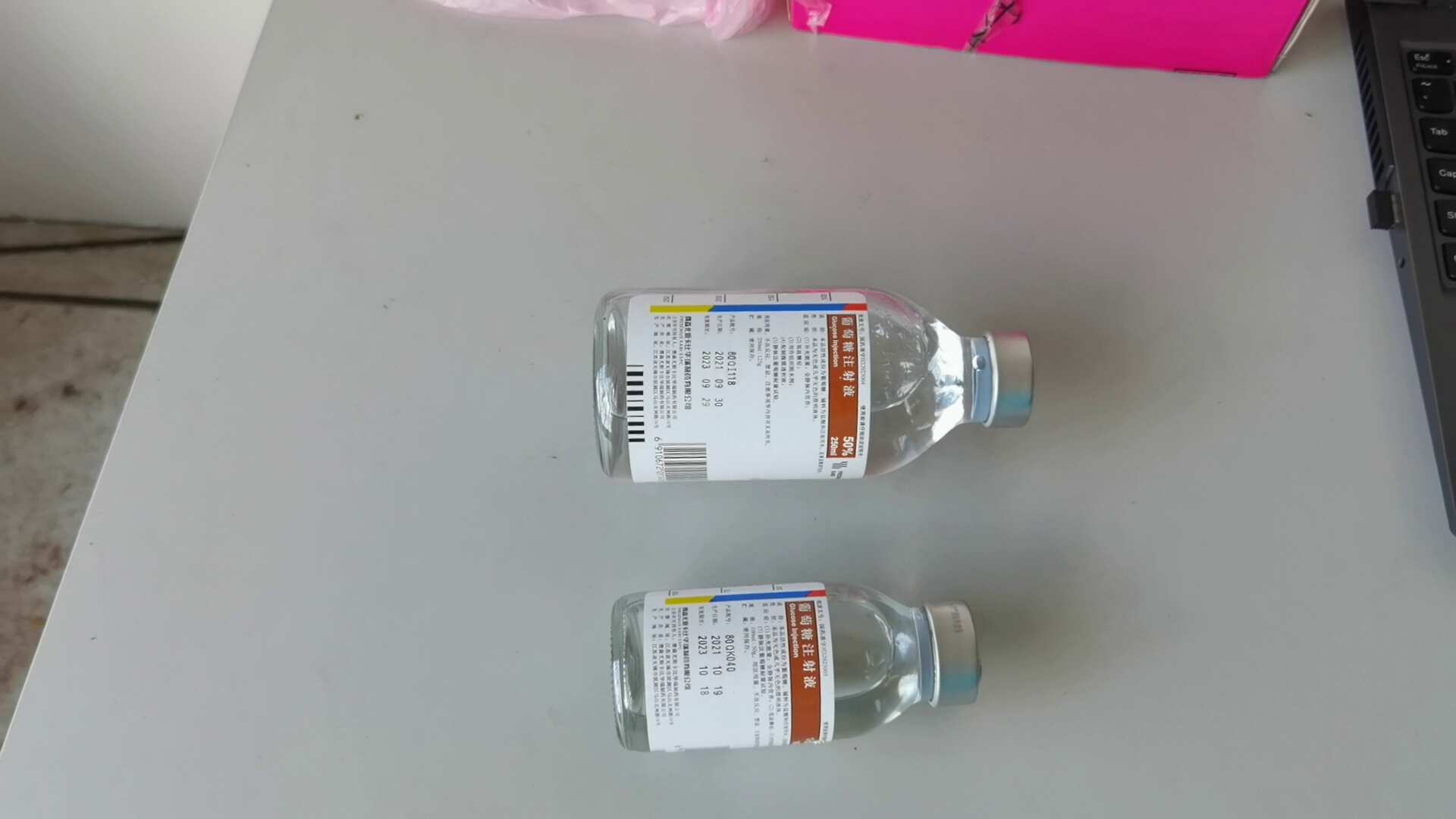

Supplement: S1 Dataset — (ZIP) [file pone.0298109.s001.zip › minimal data set/VOC2007/images/1025.jpg]

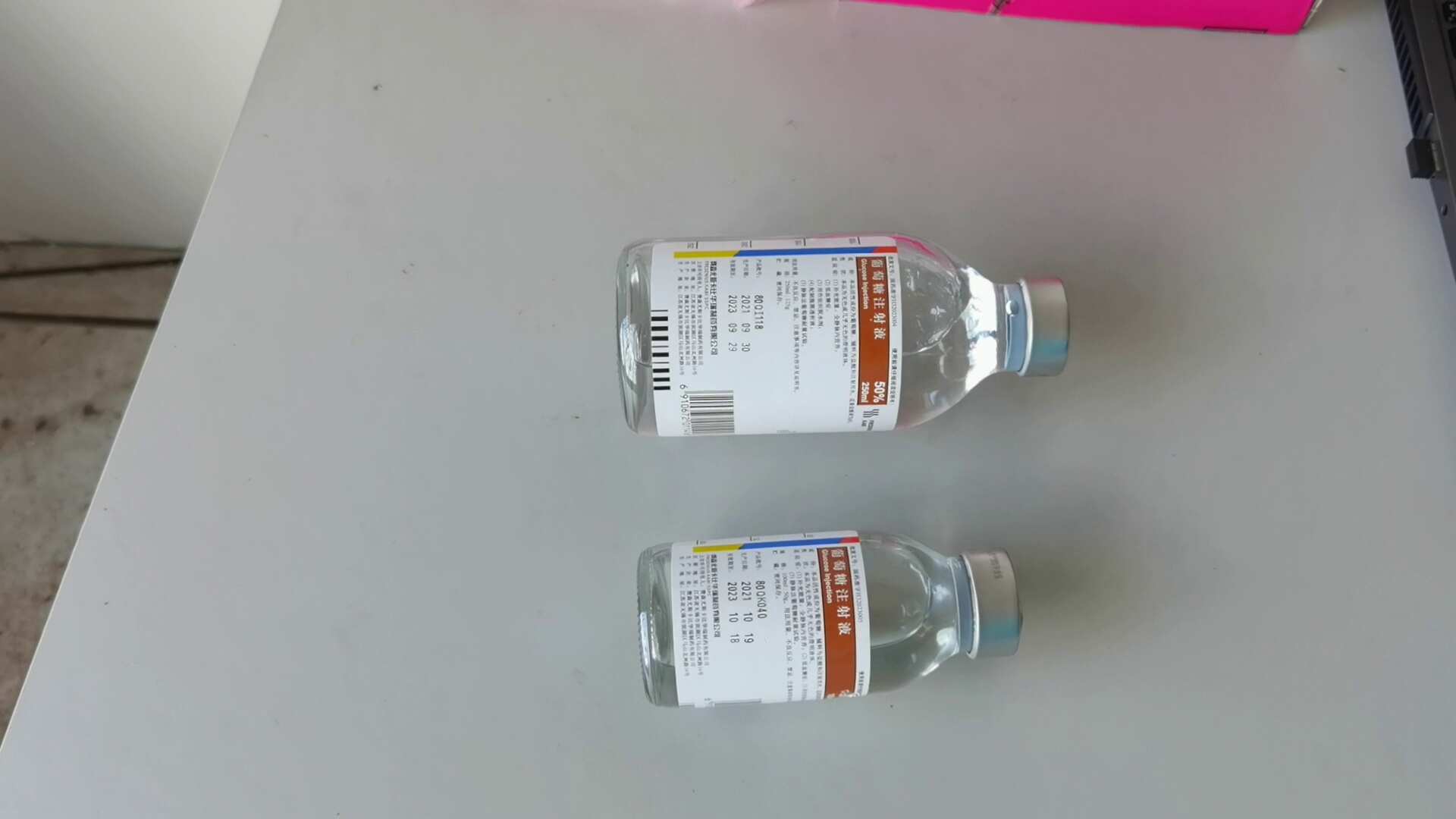

Supplement: S1 Dataset — (ZIP) [file pone.0298109.s001.zip › minimal data set/VOC2007/images/1026.jpg]

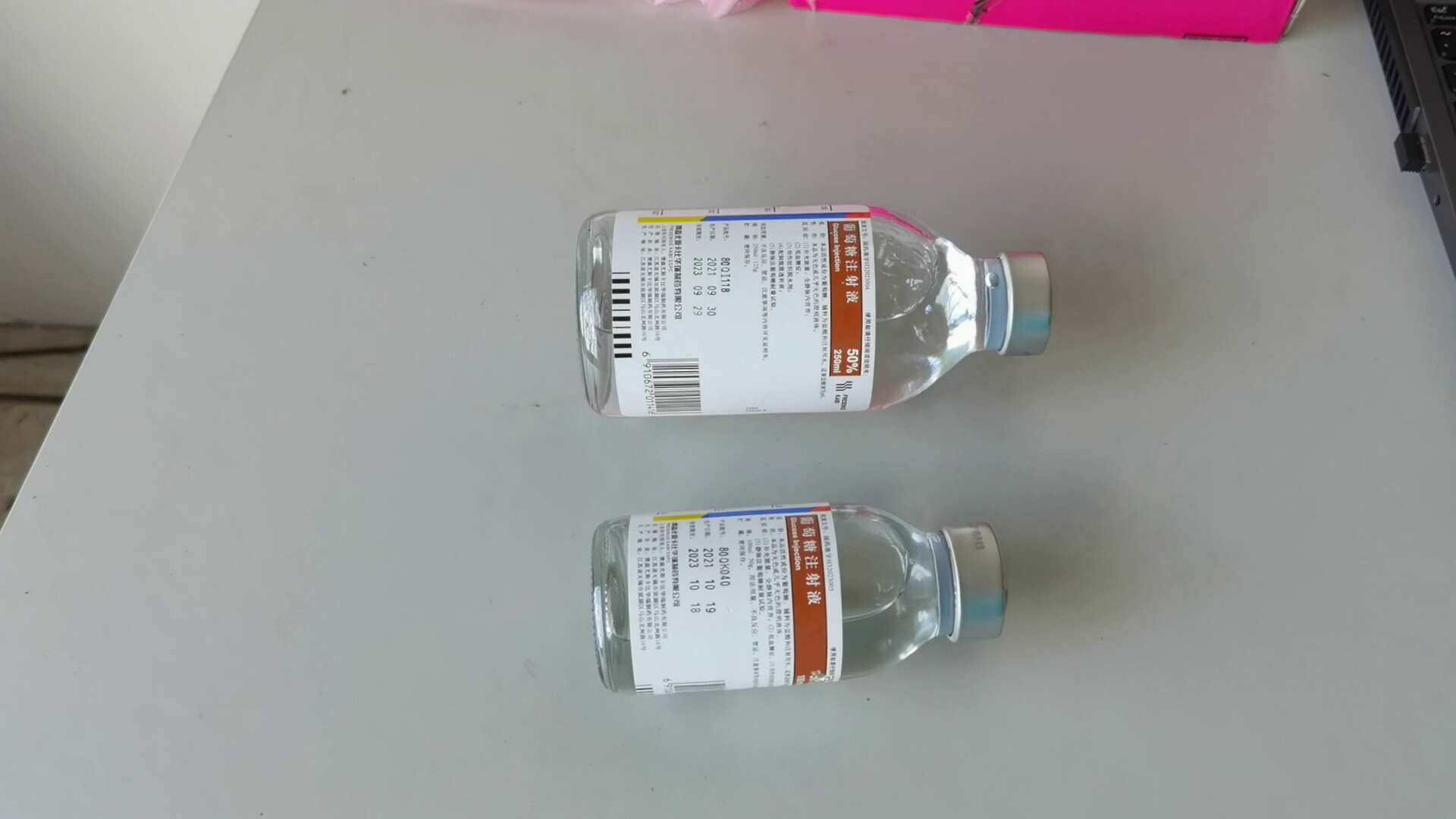

Supplement: S1 Dataset — (ZIP) [file pone.0298109.s001.zip › minimal data set/VOC2007/images/1027.jpg]

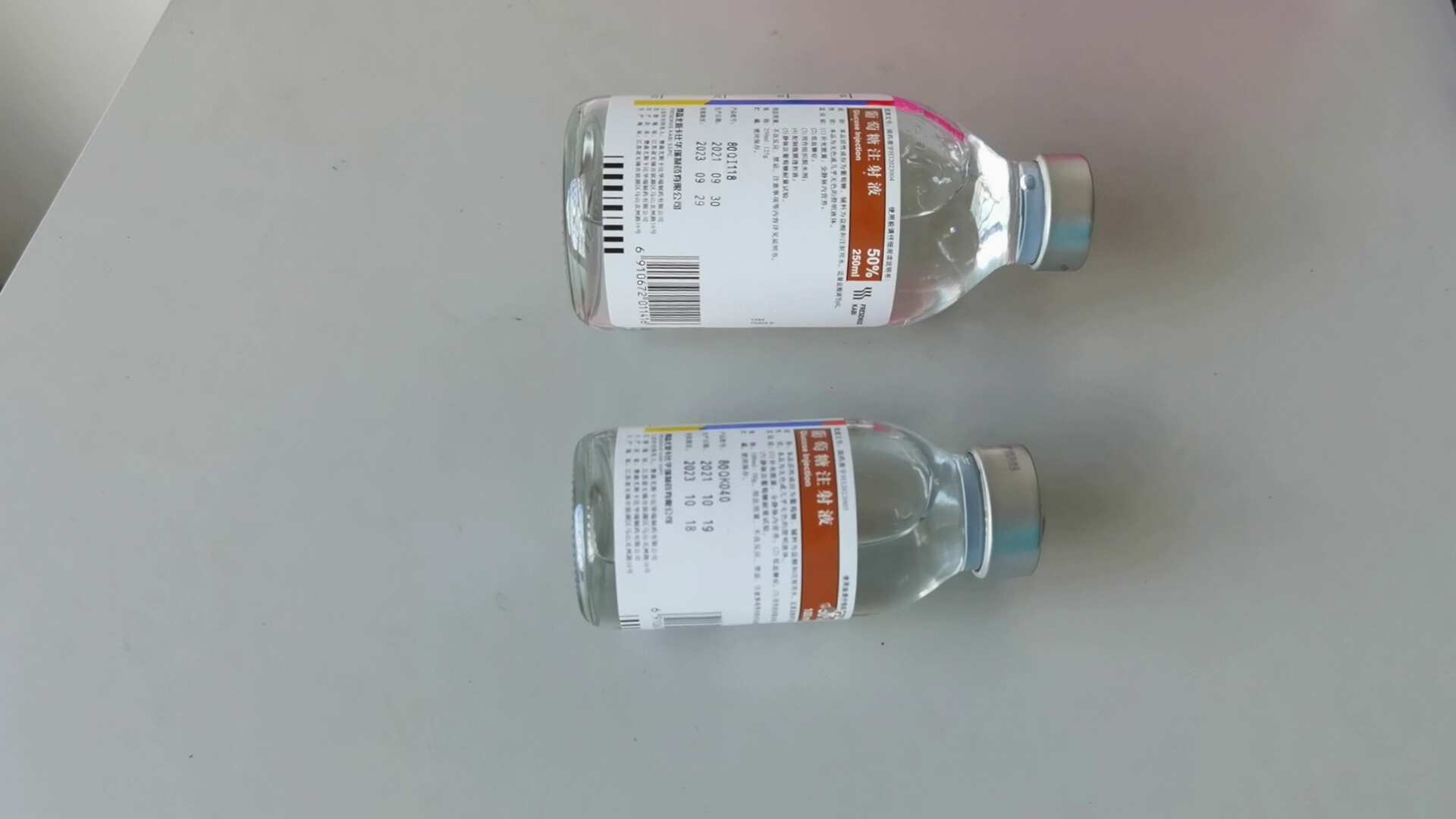

Supplement: S1 Dataset — (ZIP) [file pone.0298109.s001.zip › minimal data set/VOC2007/images/1028.jpg]

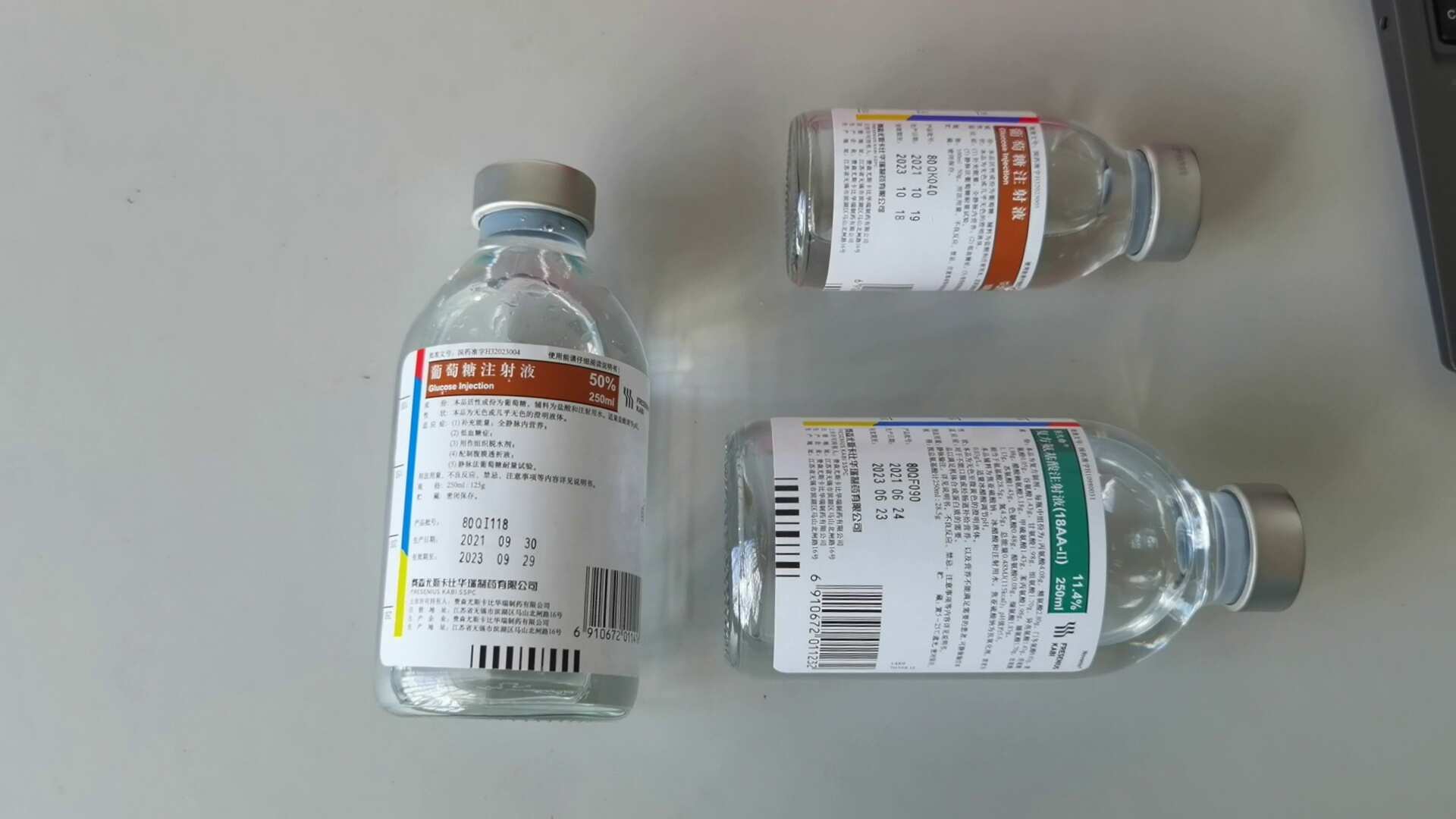

Supplement: S1 Dataset — (ZIP) [file pone.0298109.s001.zip › minimal data set/VOC2007/images/1029.jpg]

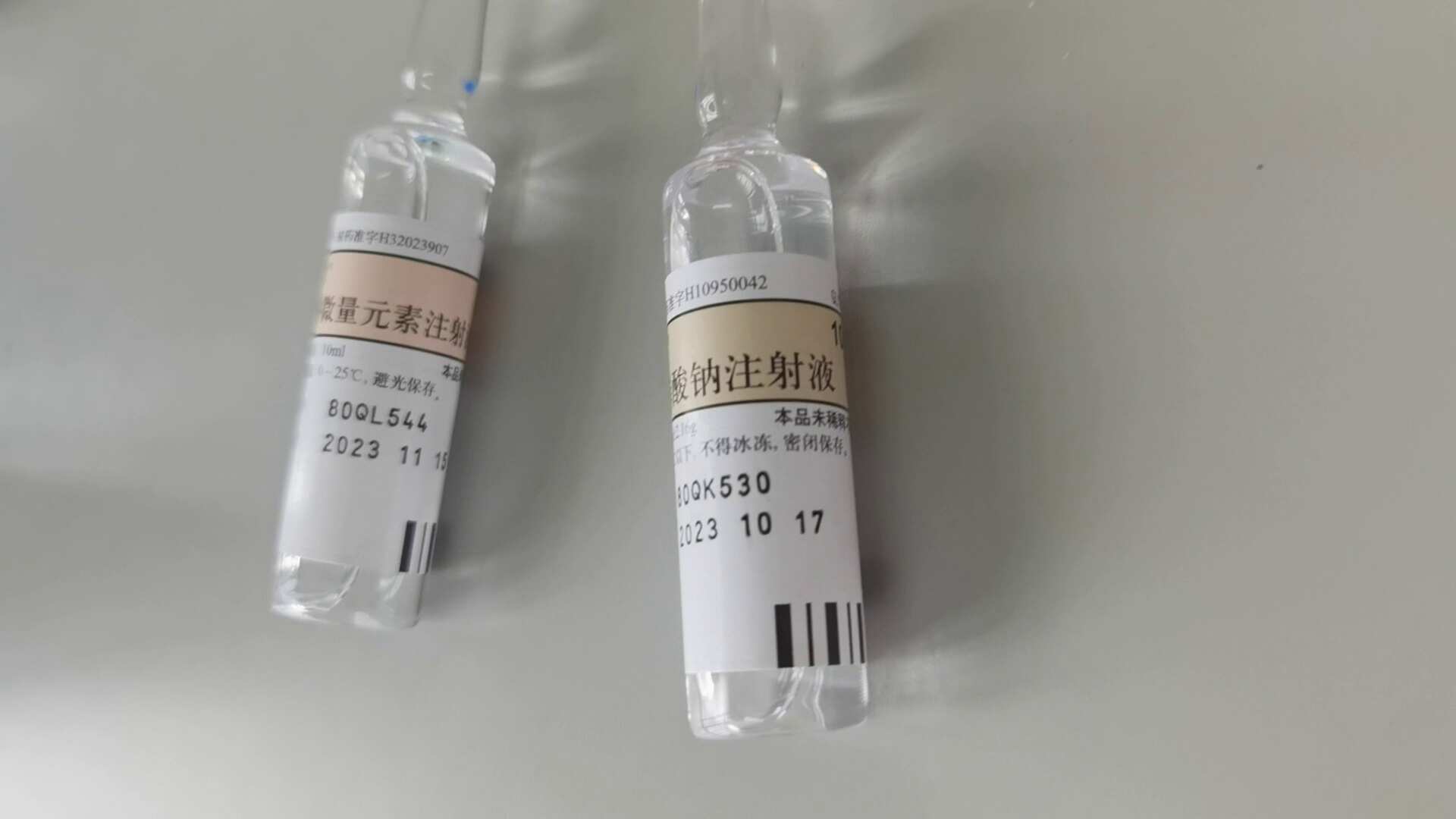

Supplement: S1 Dataset — (ZIP) [file pone.0298109.s001.zip › minimal data set/VOC2007/images/103.jpg]

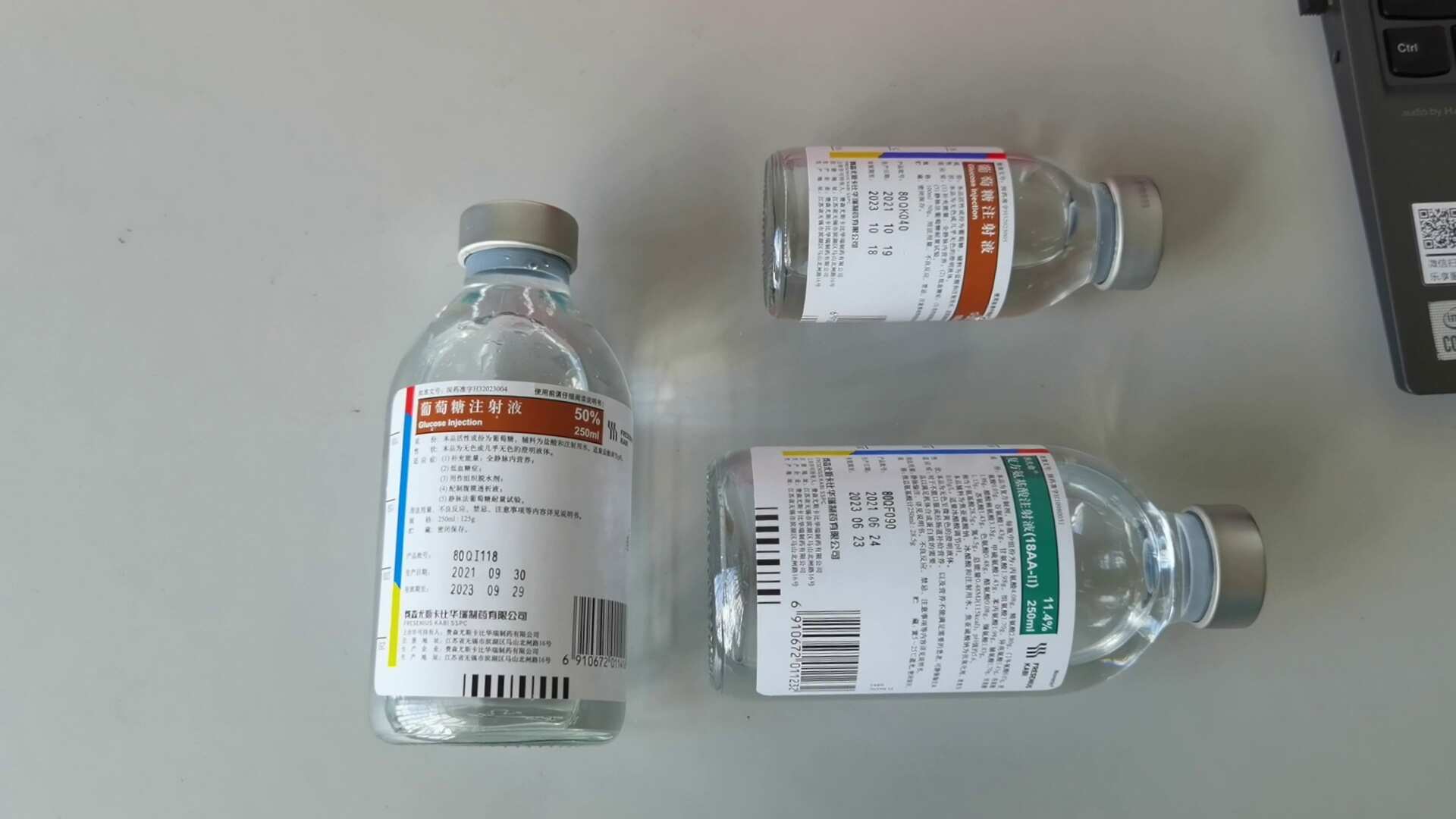

Supplement: S1 Dataset — (ZIP) [file pone.0298109.s001.zip › minimal data set/VOC2007/images/1030.jpg]

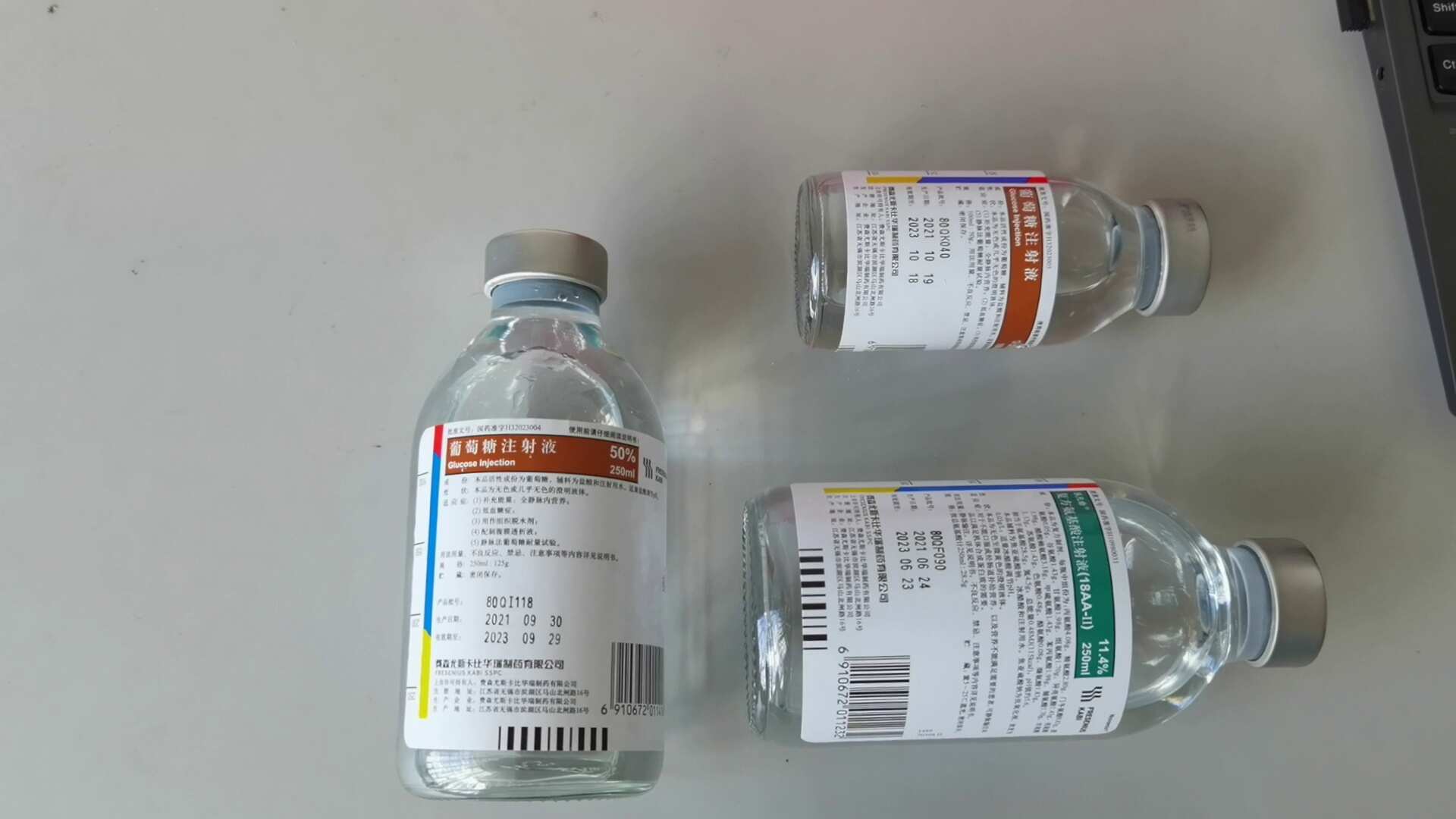

Supplement: S1 Dataset — (ZIP) [file pone.0298109.s001.zip › minimal data set/VOC2007/images/1031.jpg]

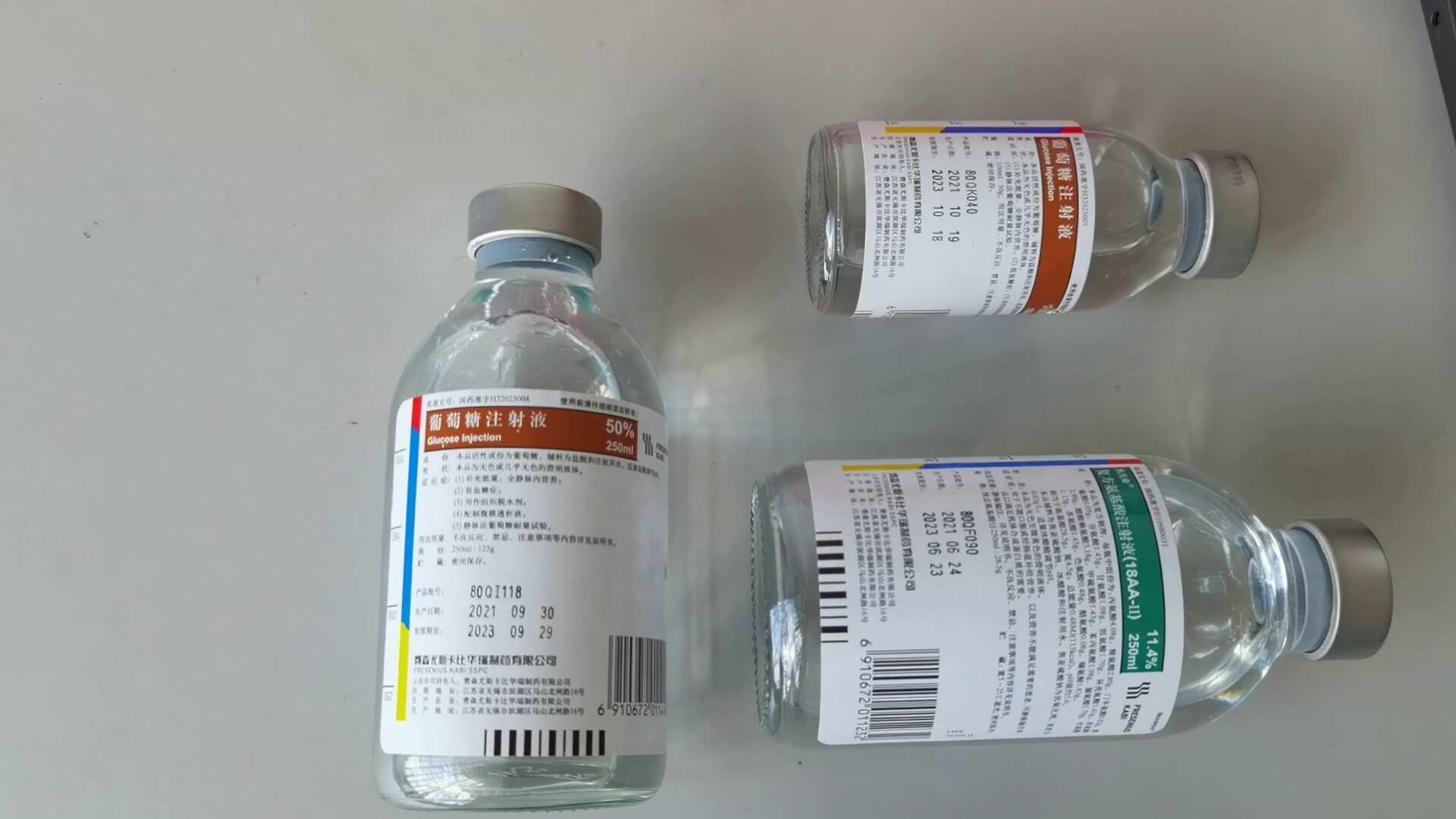

Supplement: S1 Dataset — (ZIP) [file pone.0298109.s001.zip › minimal data set/VOC2007/images/1032.jpg]

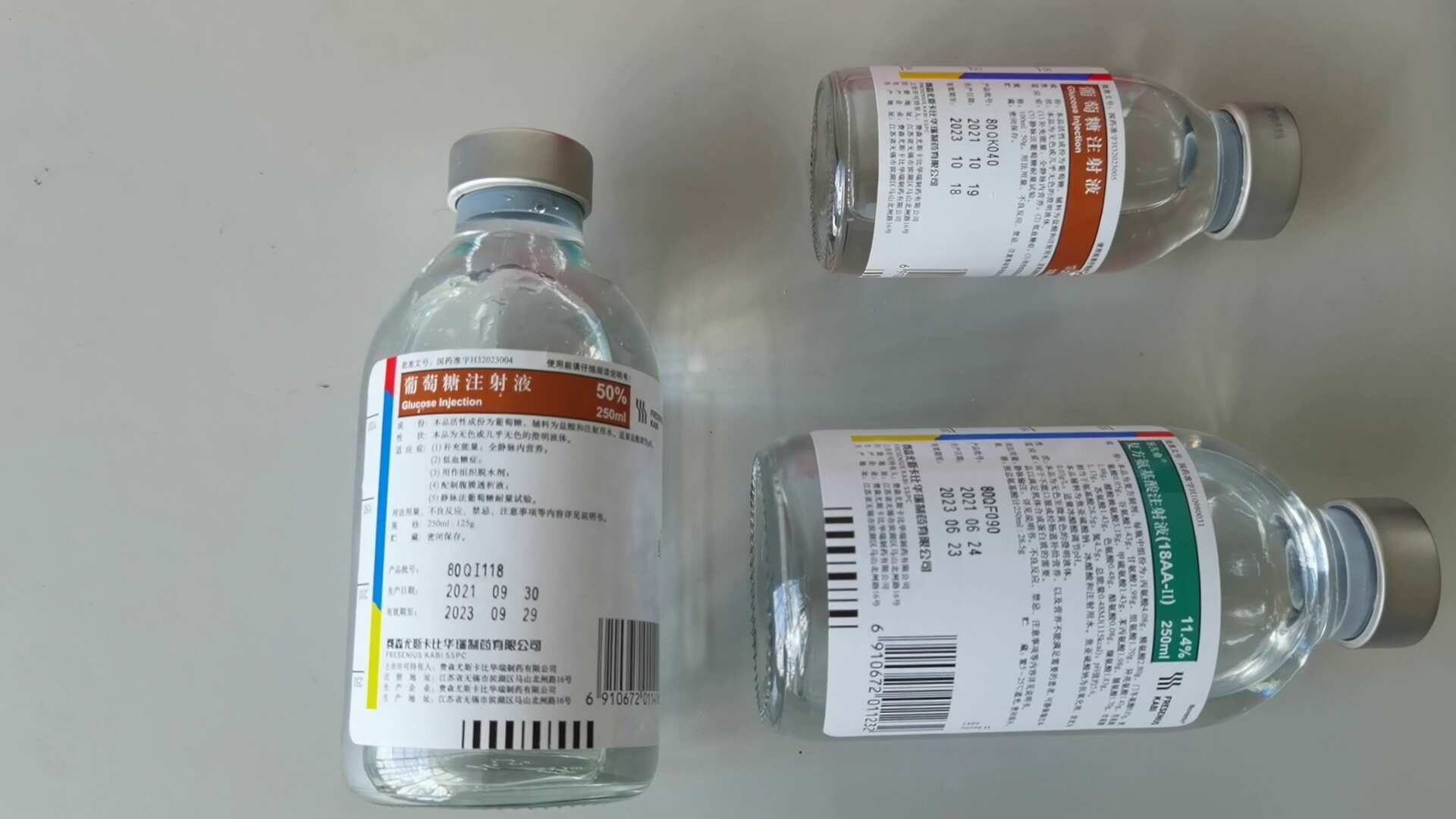

Supplement: S1 Dataset — (ZIP) [file pone.0298109.s001.zip › minimal data set/VOC2007/images/1033.jpg]

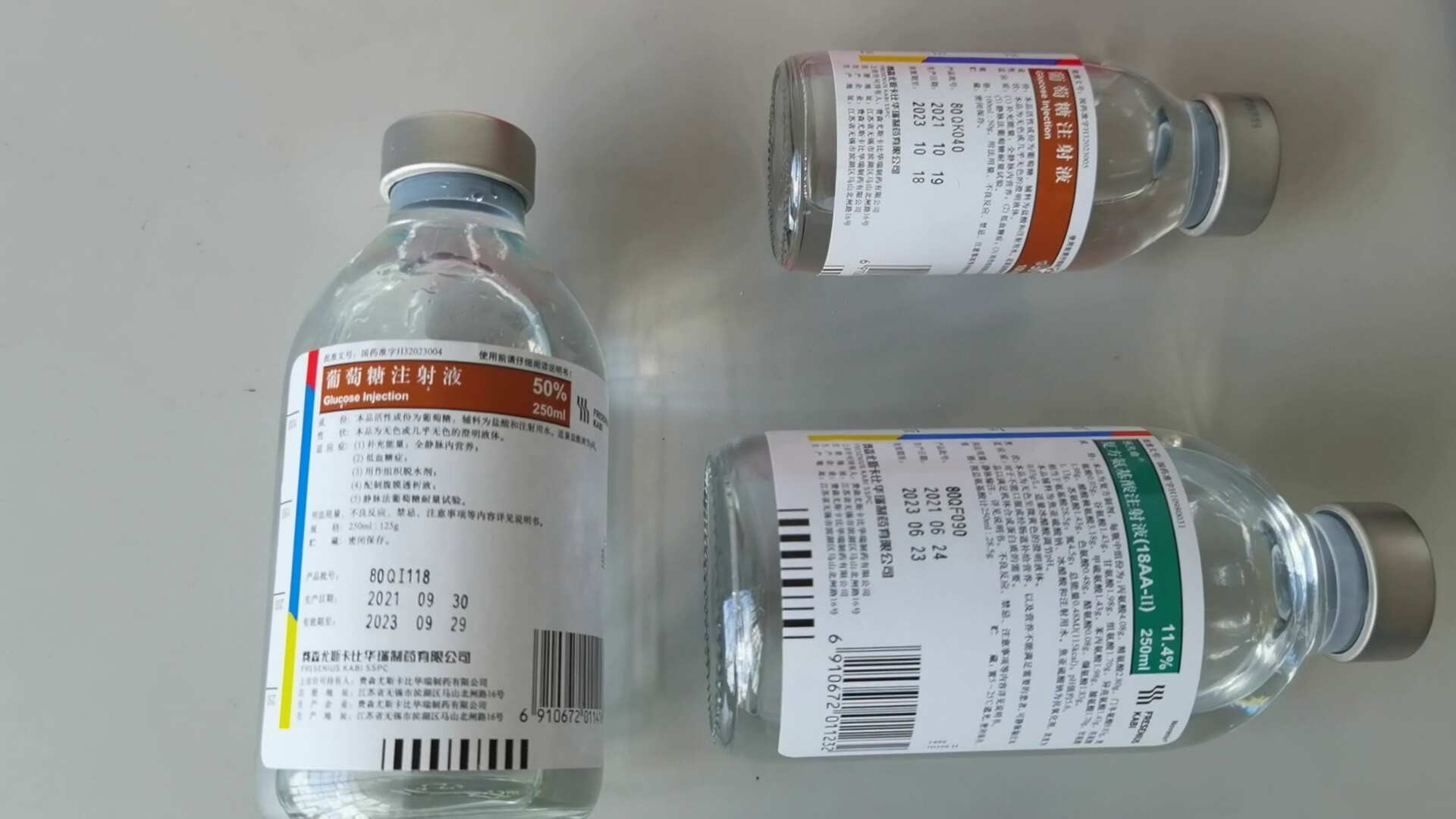

Supplement: S1 Dataset — (ZIP) [file pone.0298109.s001.zip › minimal data set/VOC2007/images/1034.jpg]

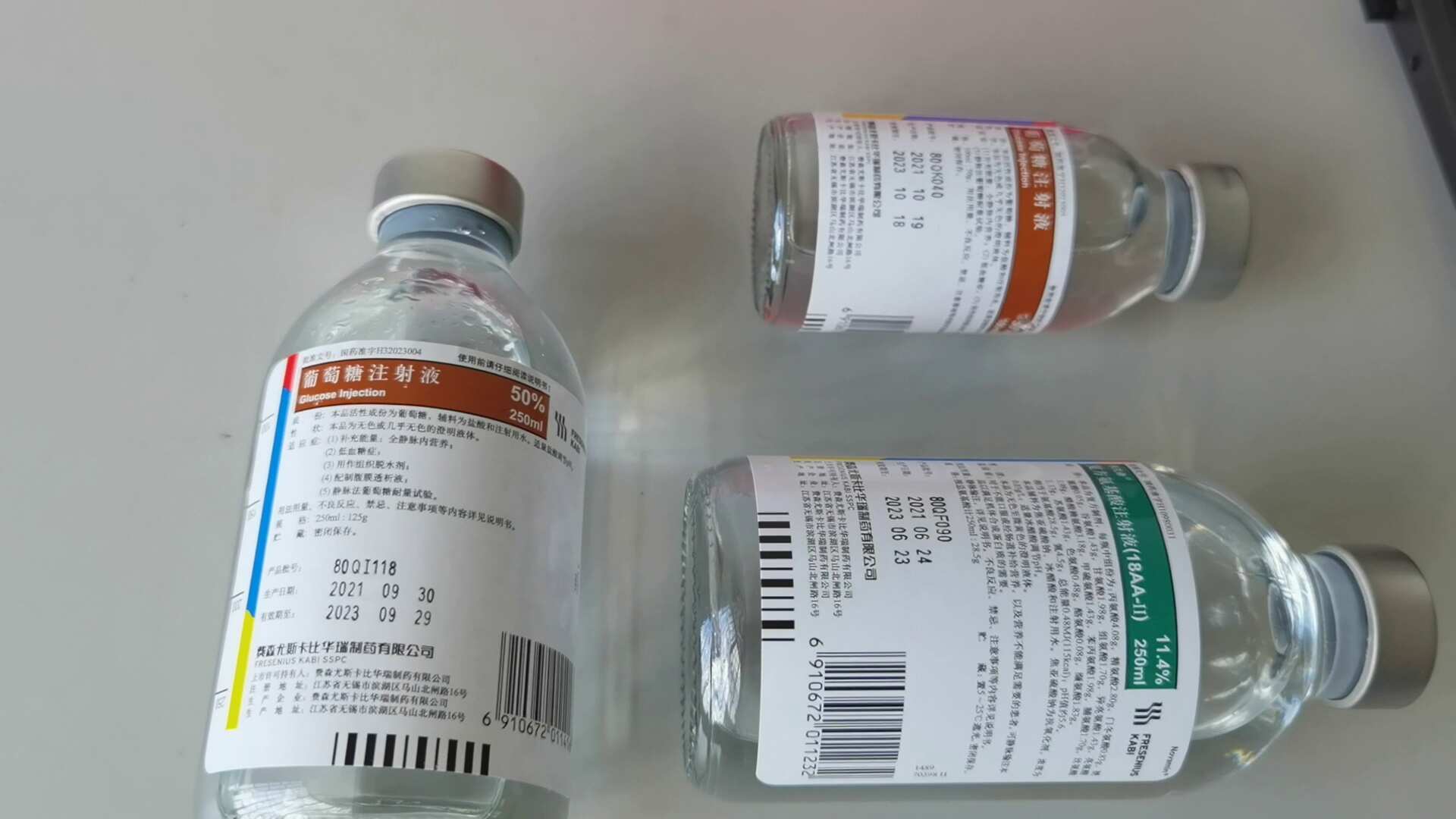

Supplement: S1 Dataset — (ZIP) [file pone.0298109.s001.zip › minimal data set/VOC2007/images/1035.jpg]

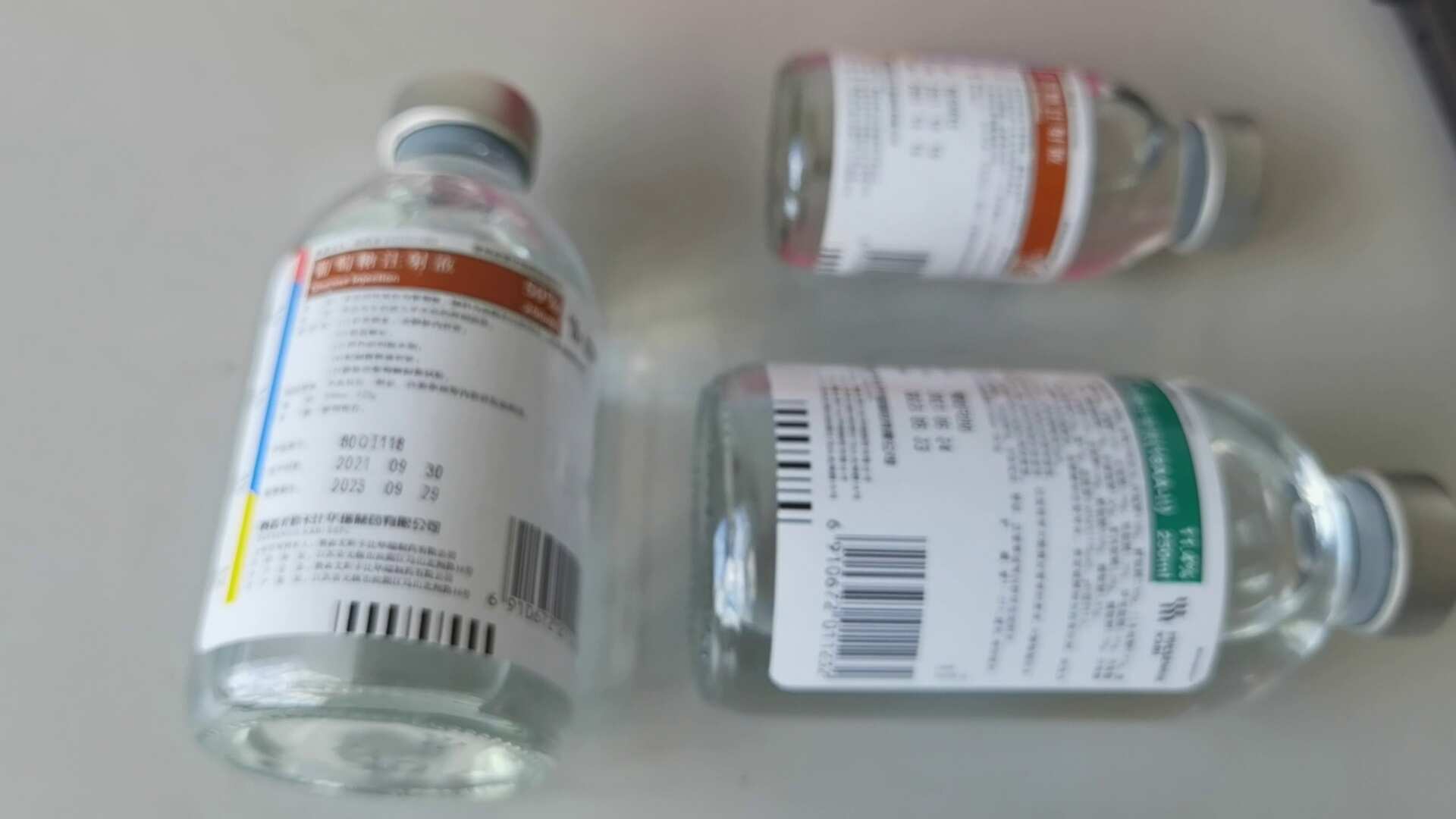

Supplement: S1 Dataset — (ZIP) [file pone.0298109.s001.zip › minimal data set/VOC2007/images/1036.jpg]

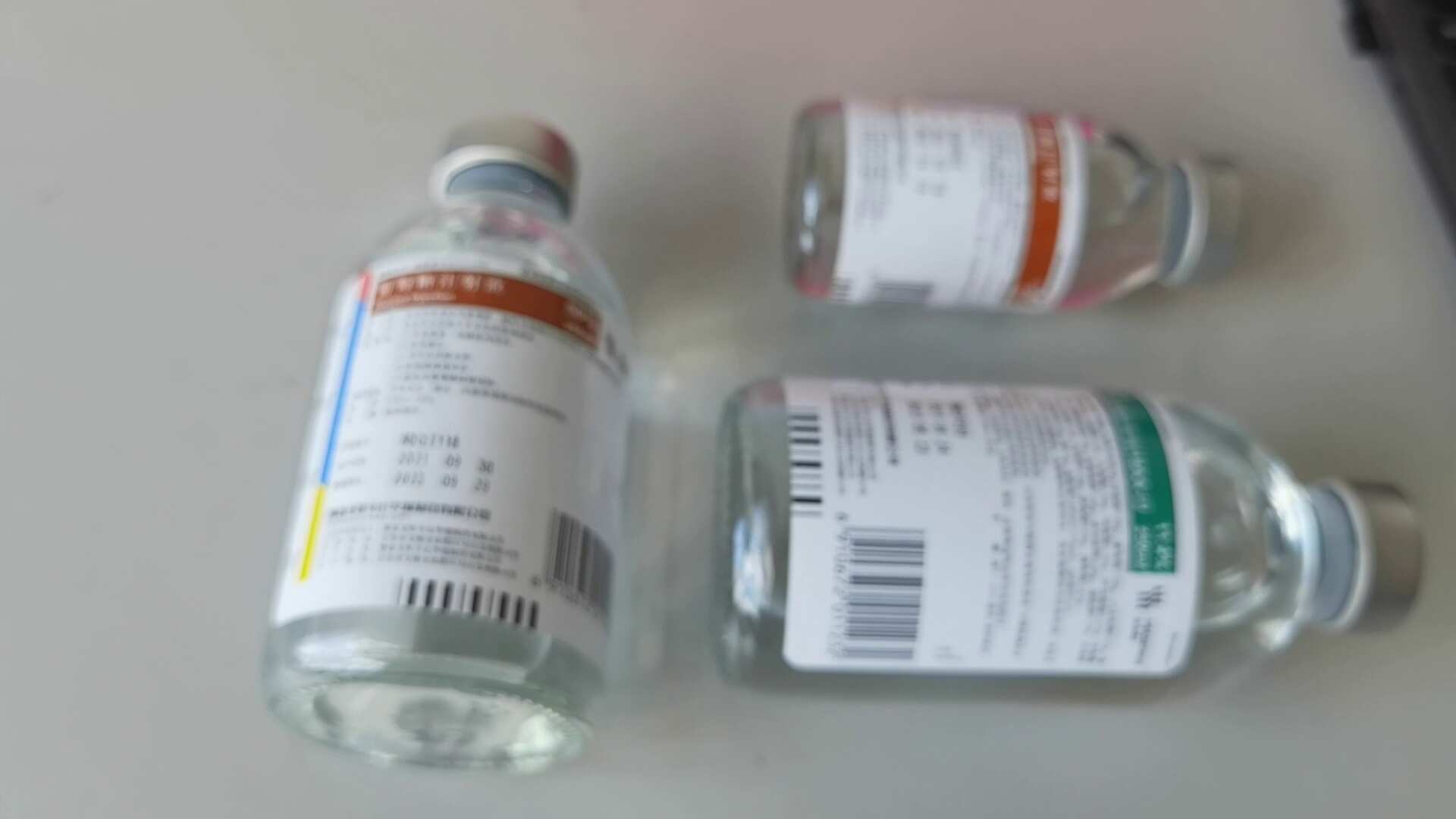

Supplement: S1 Dataset — (ZIP) [file pone.0298109.s001.zip › minimal data set/VOC2007/images/1037.jpg]

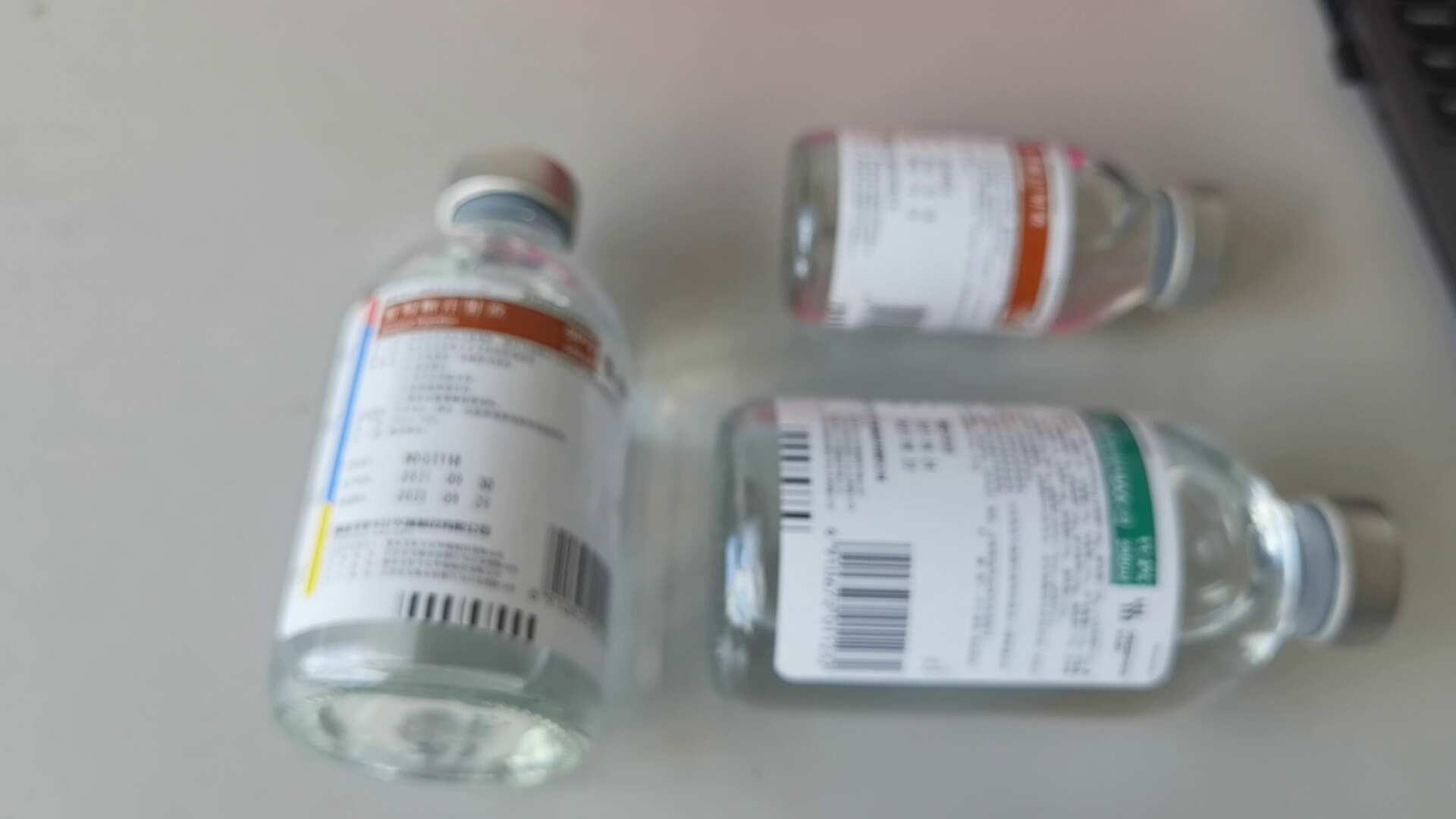

Supplement: S1 Dataset — (ZIP) [file pone.0298109.s001.zip › minimal data set/VOC2007/images/1038.jpg]

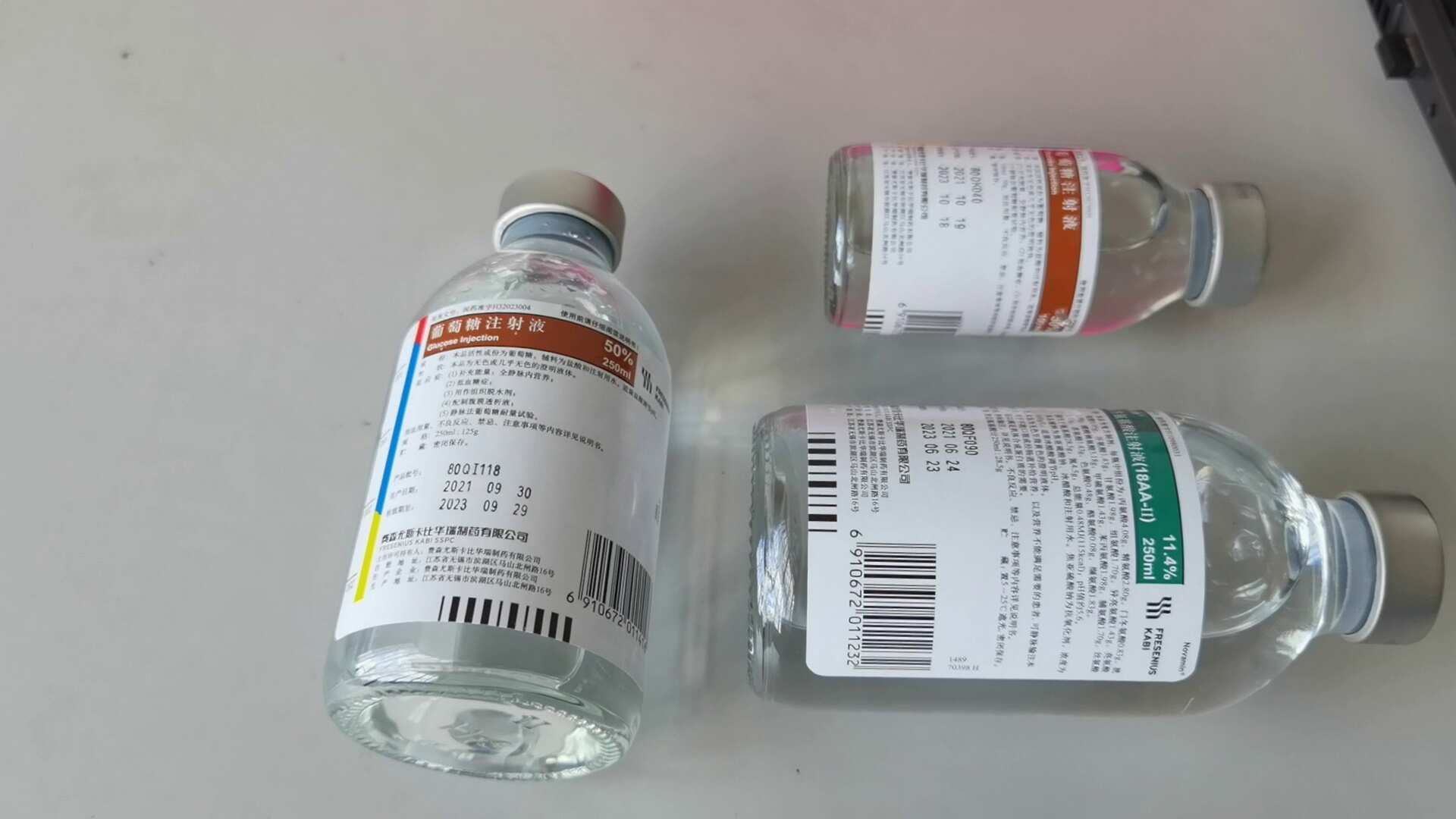

Supplement: S1 Dataset — (ZIP) [file pone.0298109.s001.zip › minimal data set/VOC2007/images/1039.jpg]

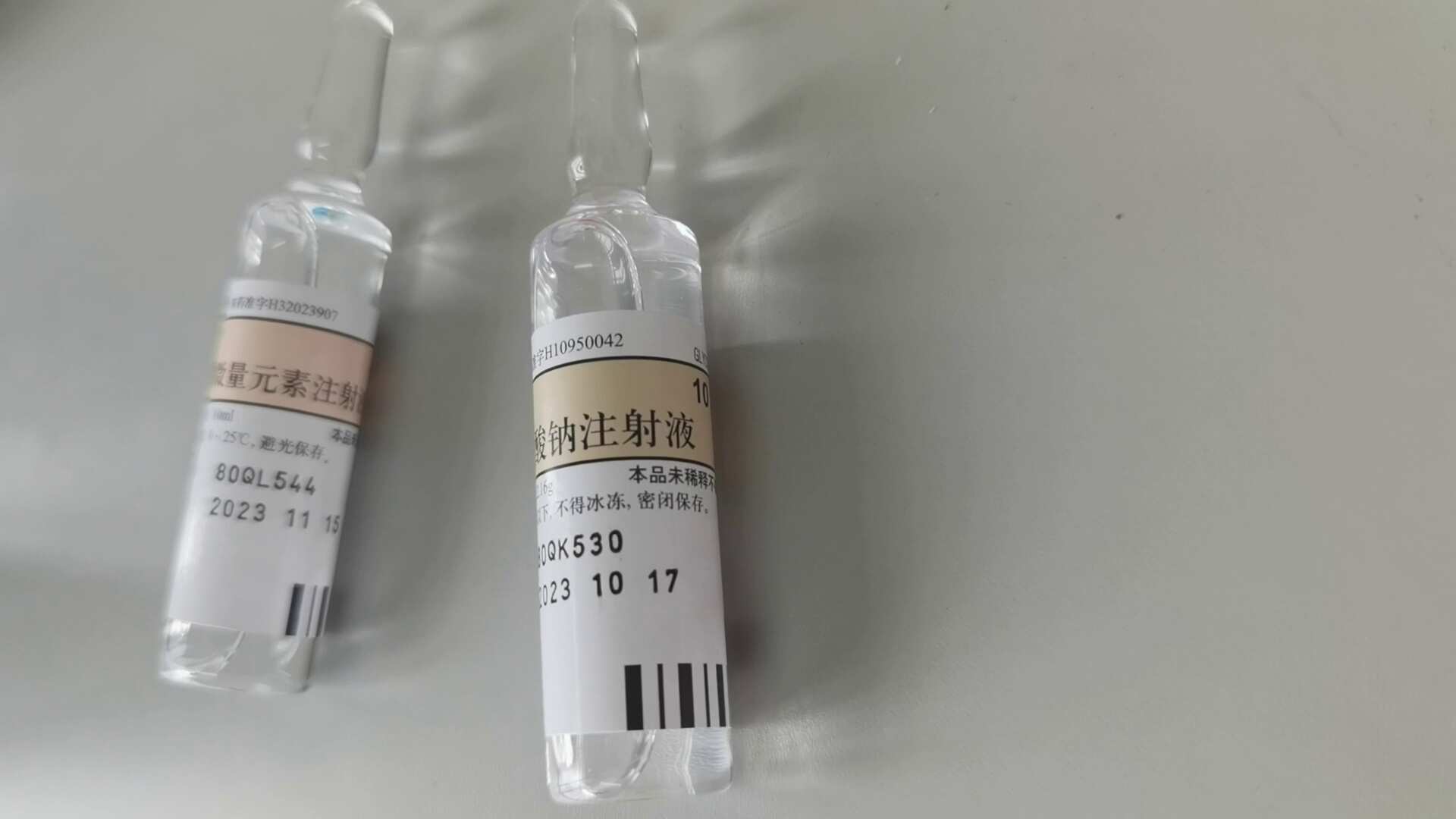

Supplement: S1 Dataset — (ZIP) [file pone.0298109.s001.zip › minimal data set/VOC2007/images/104.jpg]

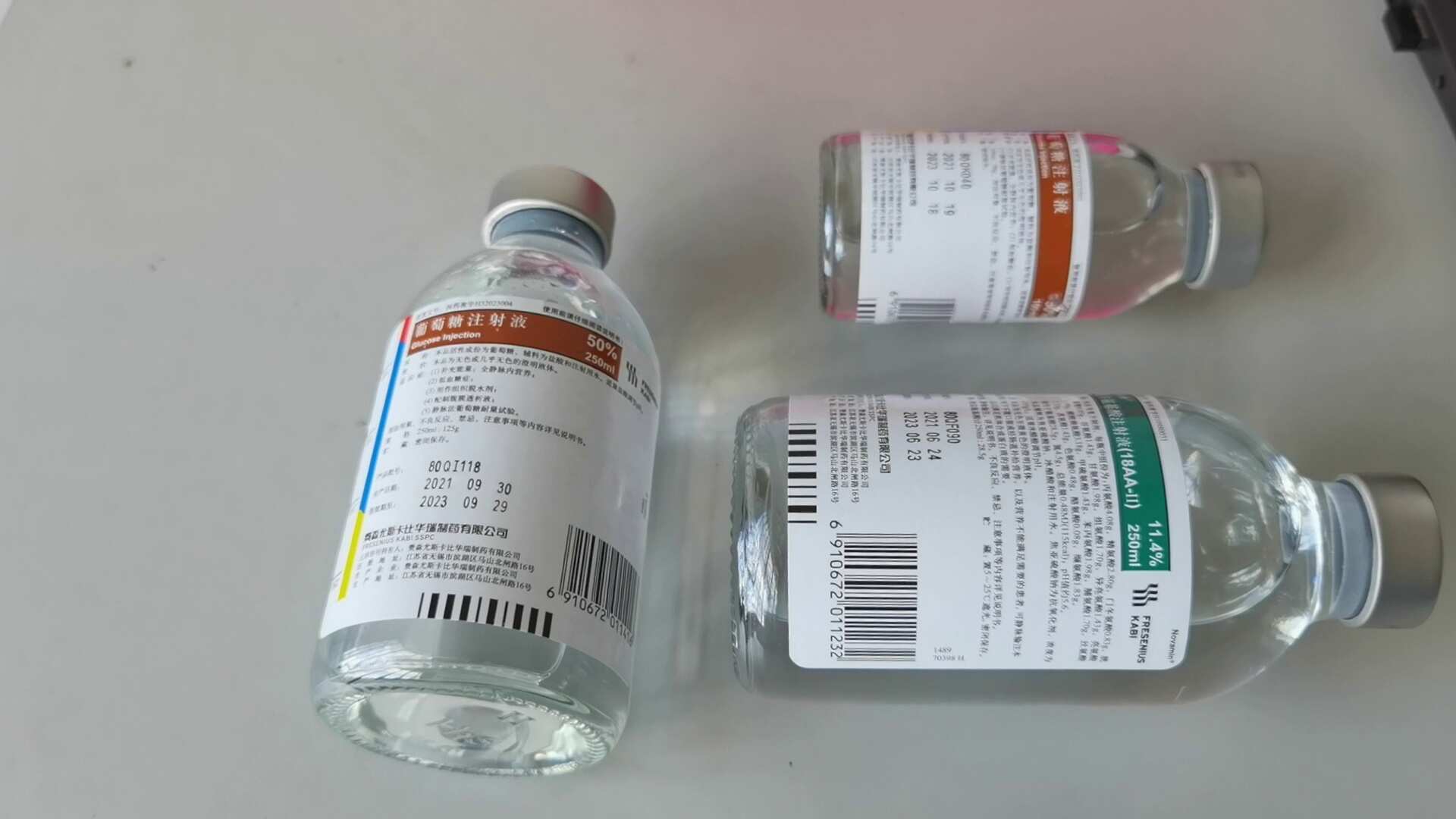

Supplement: S1 Dataset — (ZIP) [file pone.0298109.s001.zip › minimal data set/VOC2007/images/1040.jpg]

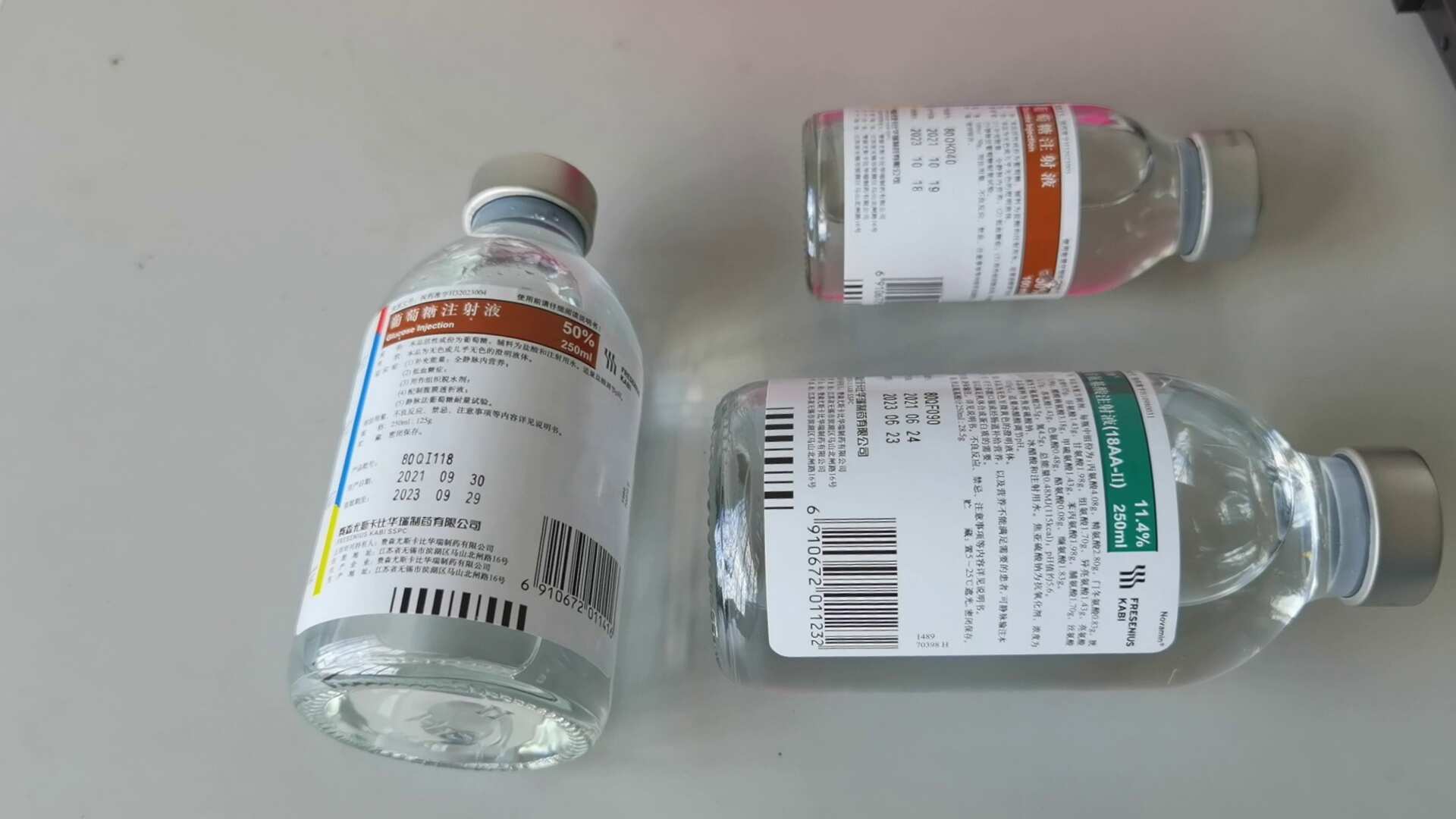

Supplement: S1 Dataset — (ZIP) [file pone.0298109.s001.zip › minimal data set/VOC2007/images/1041.jpg]

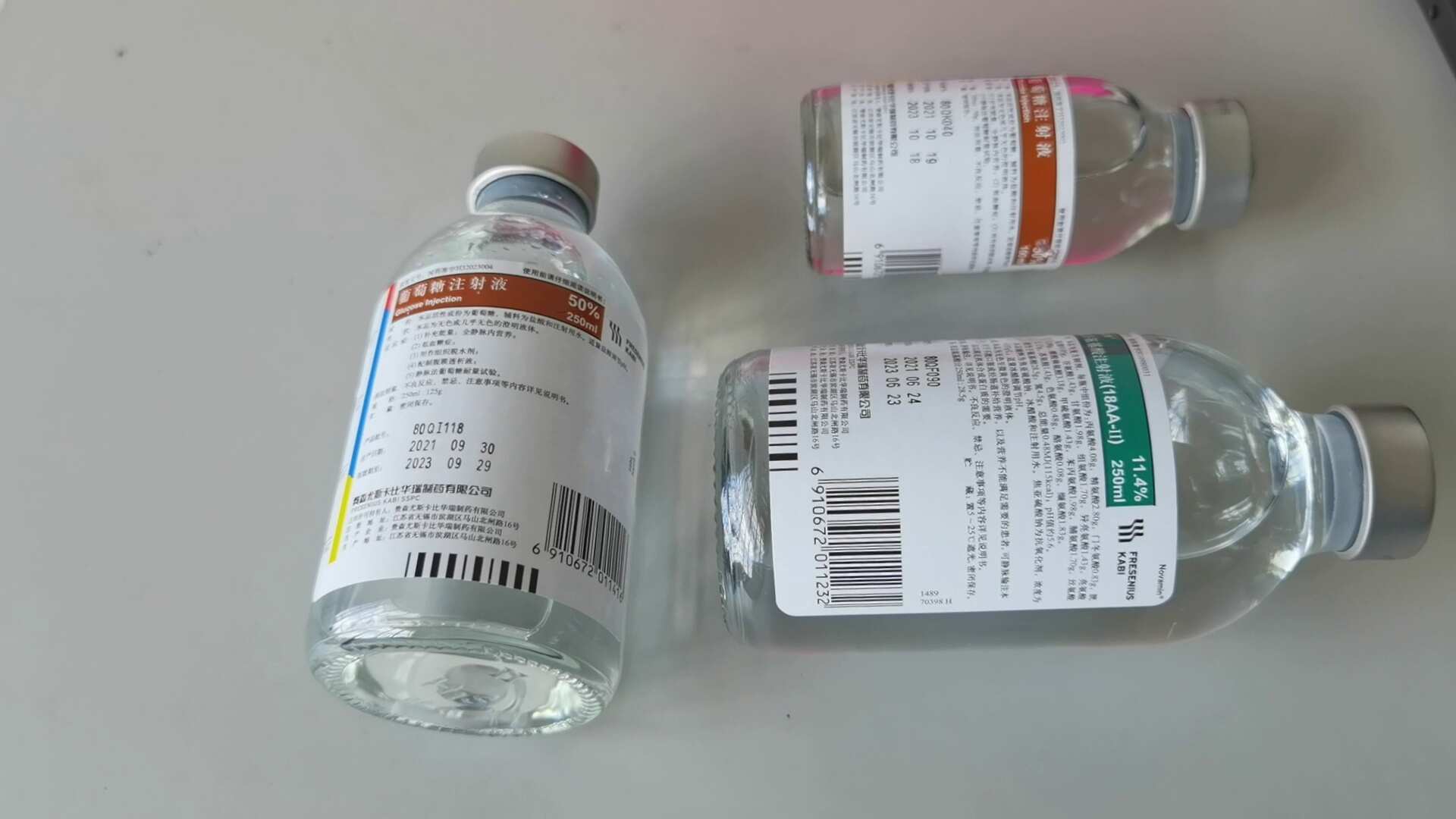

Supplement: S1 Dataset — (ZIP) [file pone.0298109.s001.zip › minimal data set/VOC2007/images/1042.jpg]

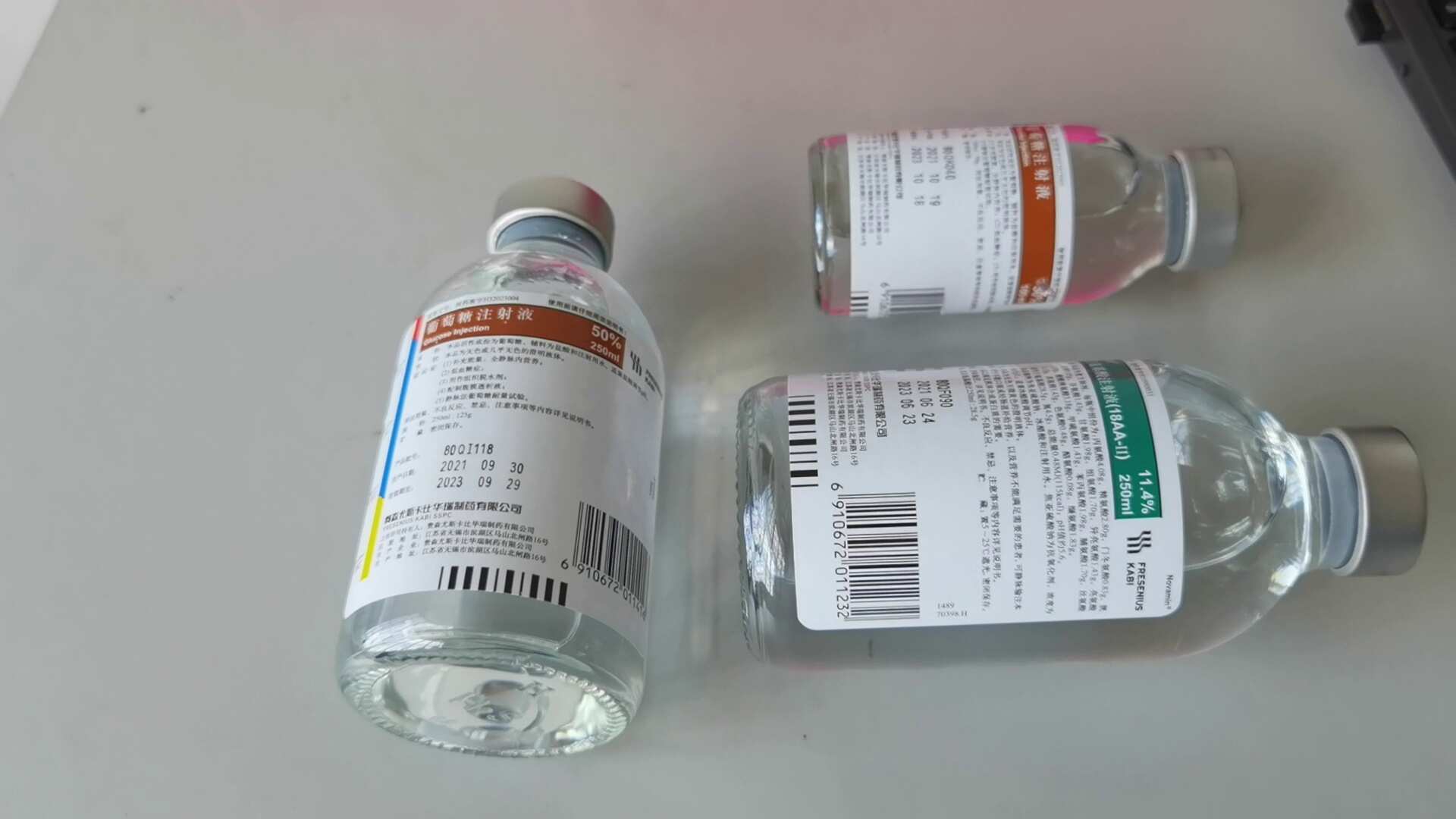

Supplement: S1 Dataset — (ZIP) [file pone.0298109.s001.zip › minimal data set/VOC2007/images/1043.jpg]

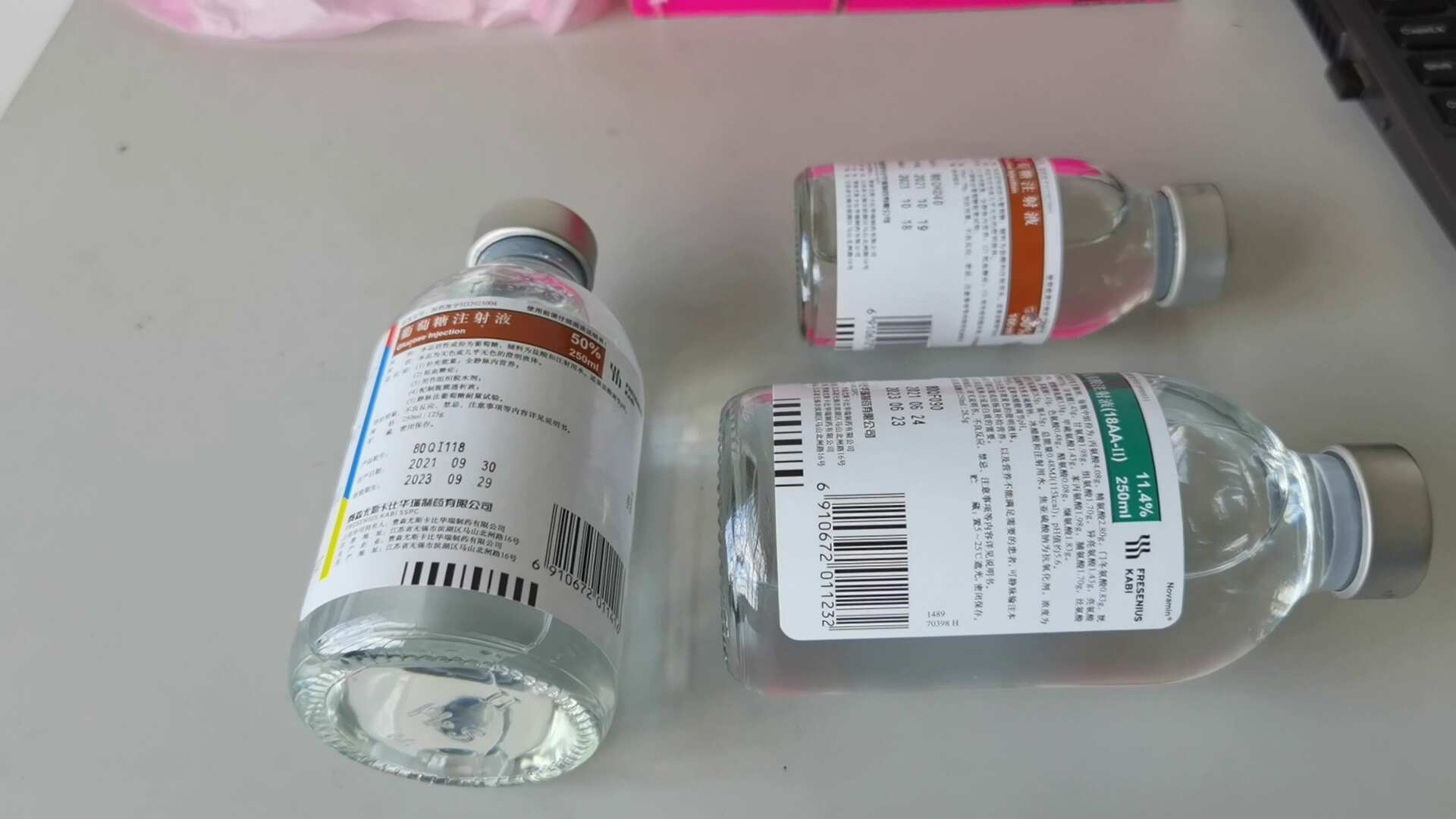

Supplement: S1 Dataset — (ZIP) [file pone.0298109.s001.zip › minimal data set/VOC2007/images/1044.jpg]

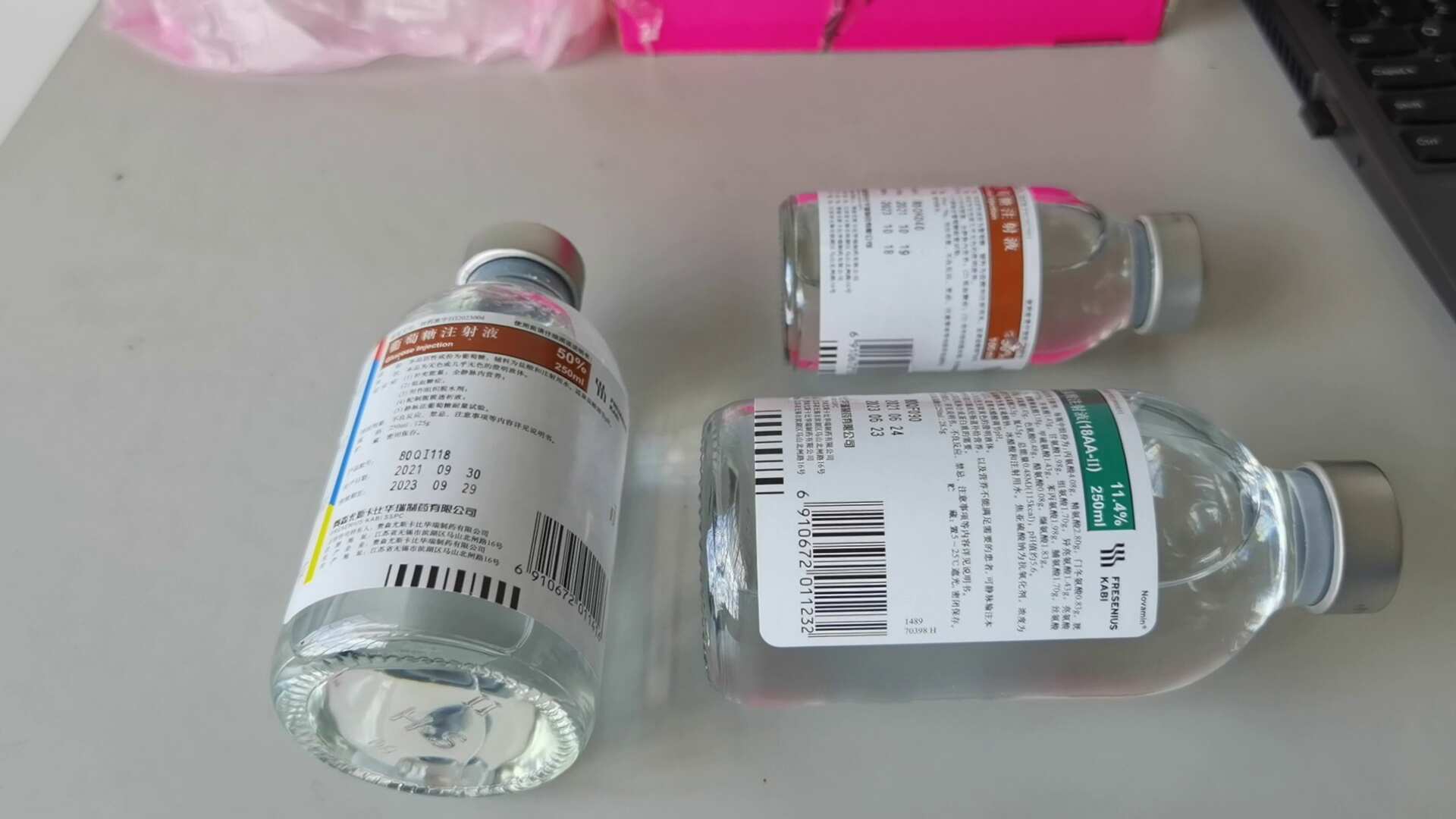

Supplement: S1 Dataset — (ZIP) [file pone.0298109.s001.zip › minimal data set/VOC2007/images/1045.jpg]

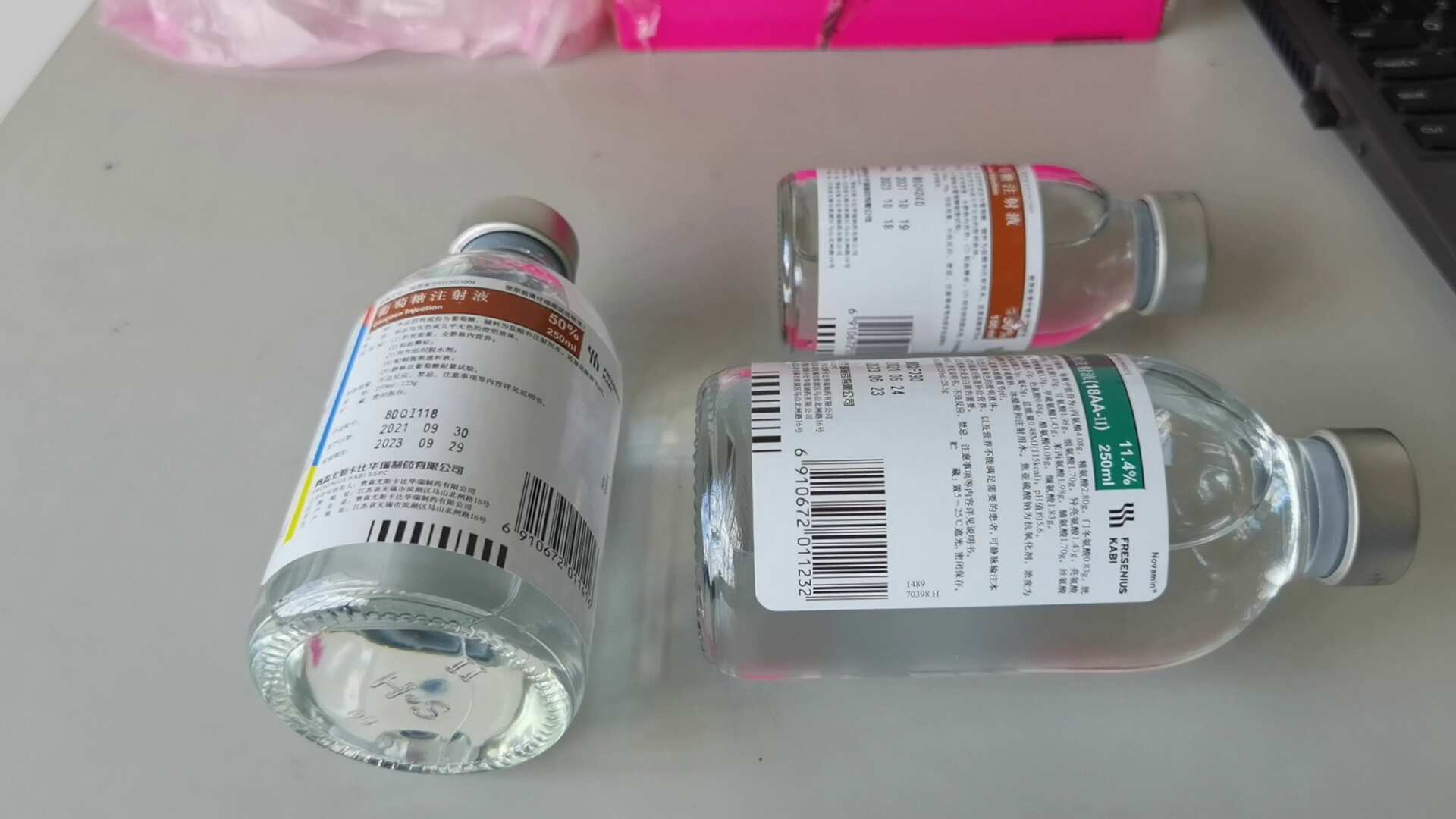

Supplement: S1 Dataset — (ZIP) [file pone.0298109.s001.zip › minimal data set/VOC2007/images/1046.jpg]

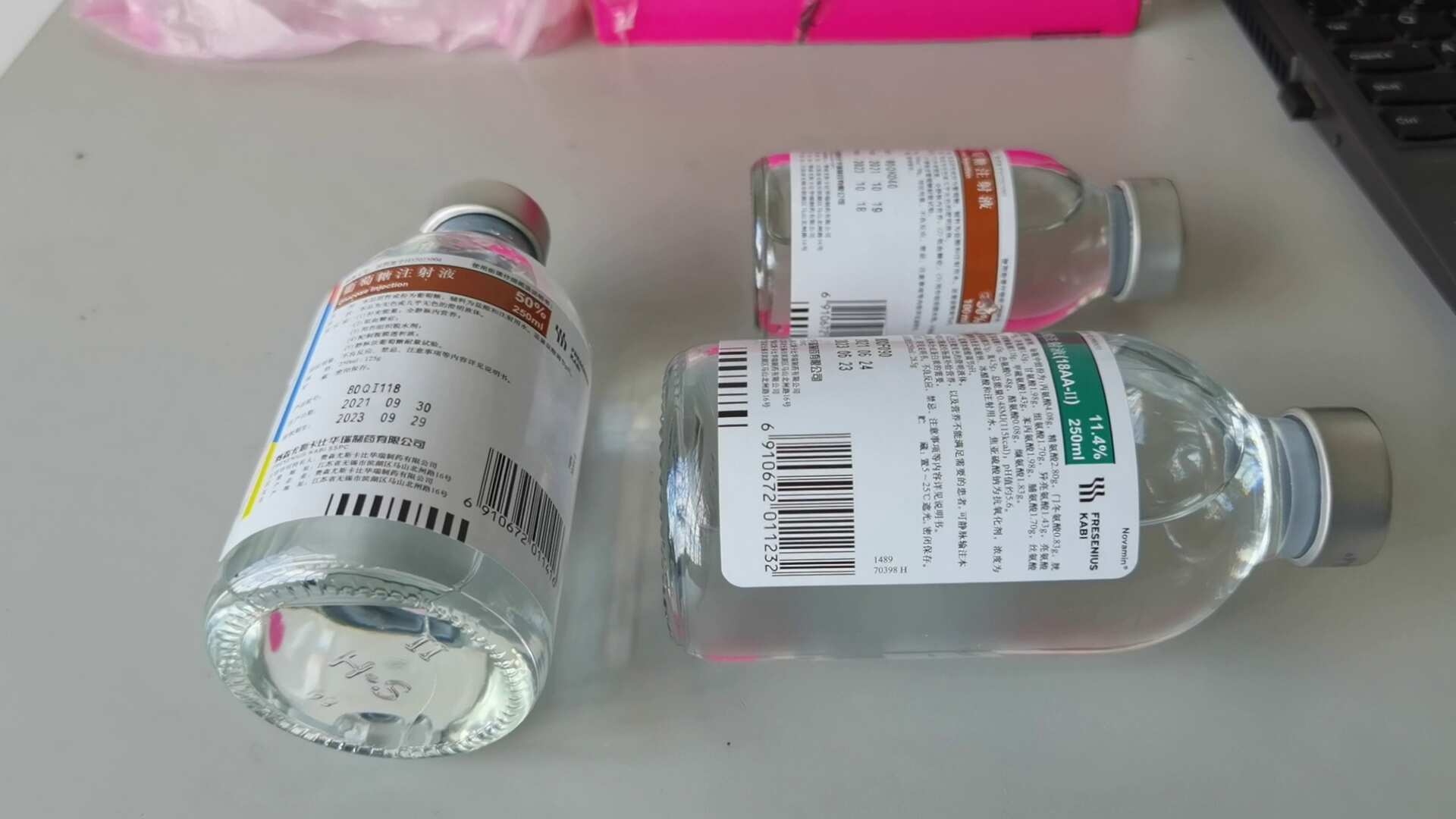

Supplement: S1 Dataset — (ZIP) [file pone.0298109.s001.zip › minimal data set/VOC2007/images/1047.jpg]

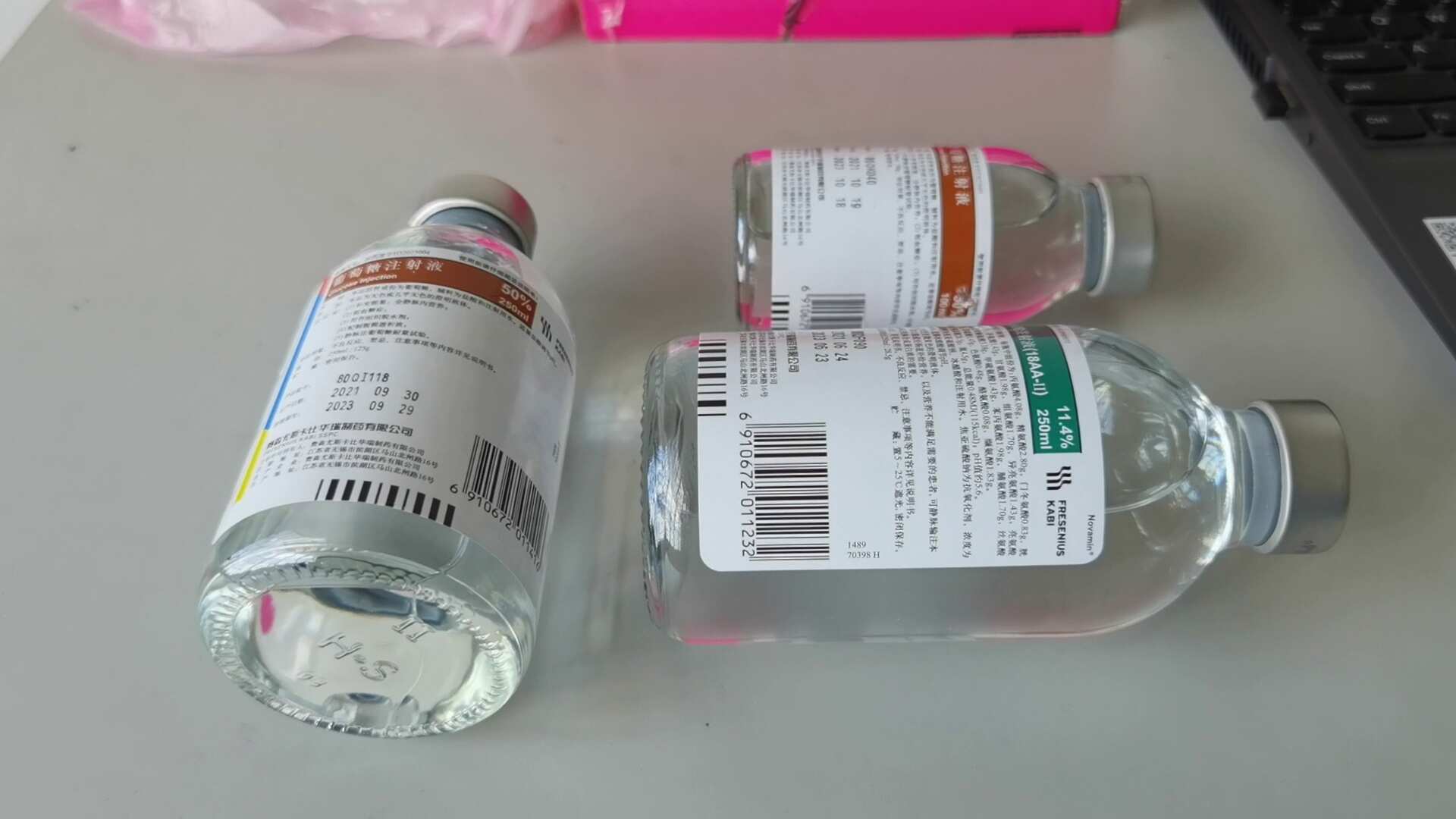

Supplement: S1 Dataset — (ZIP) [file pone.0298109.s001.zip › minimal data set/VOC2007/images/1048.jpg]

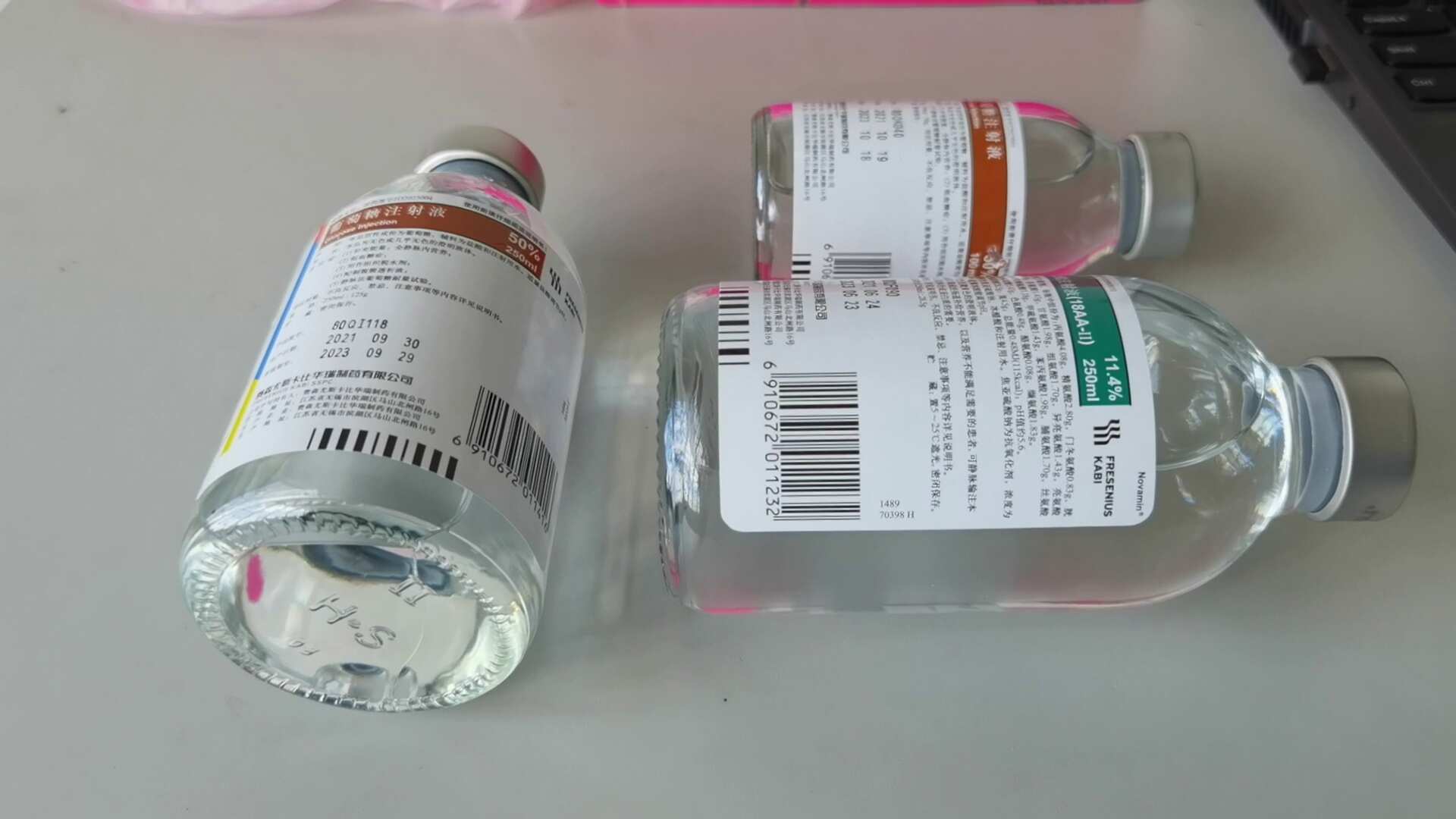

Supplement: S1 Dataset — (ZIP) [file pone.0298109.s001.zip › minimal data set/VOC2007/images/1049.jpg]

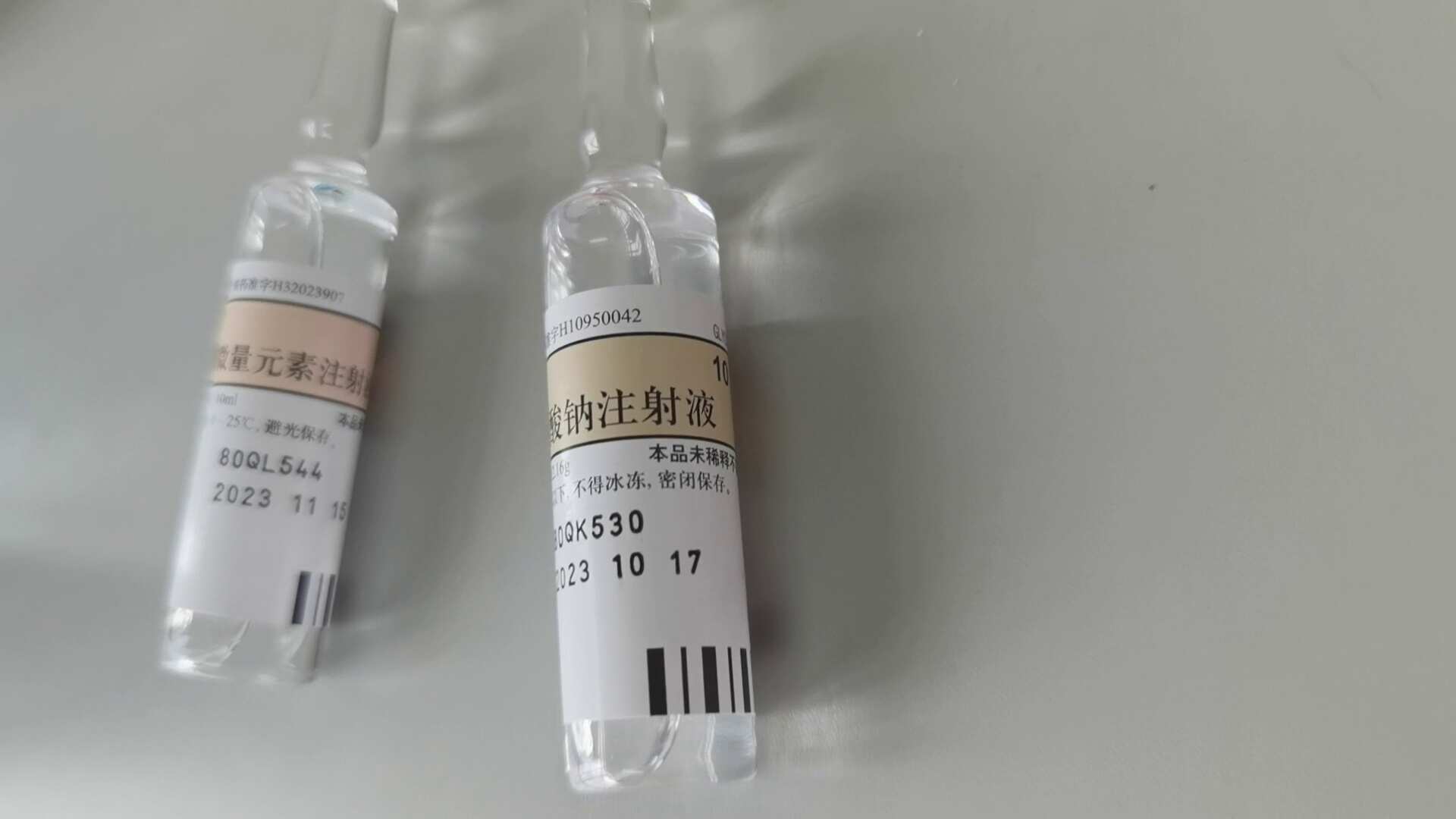

Supplement: S1 Dataset — (ZIP) [file pone.0298109.s001.zip › minimal data set/VOC2007/images/105.jpg]

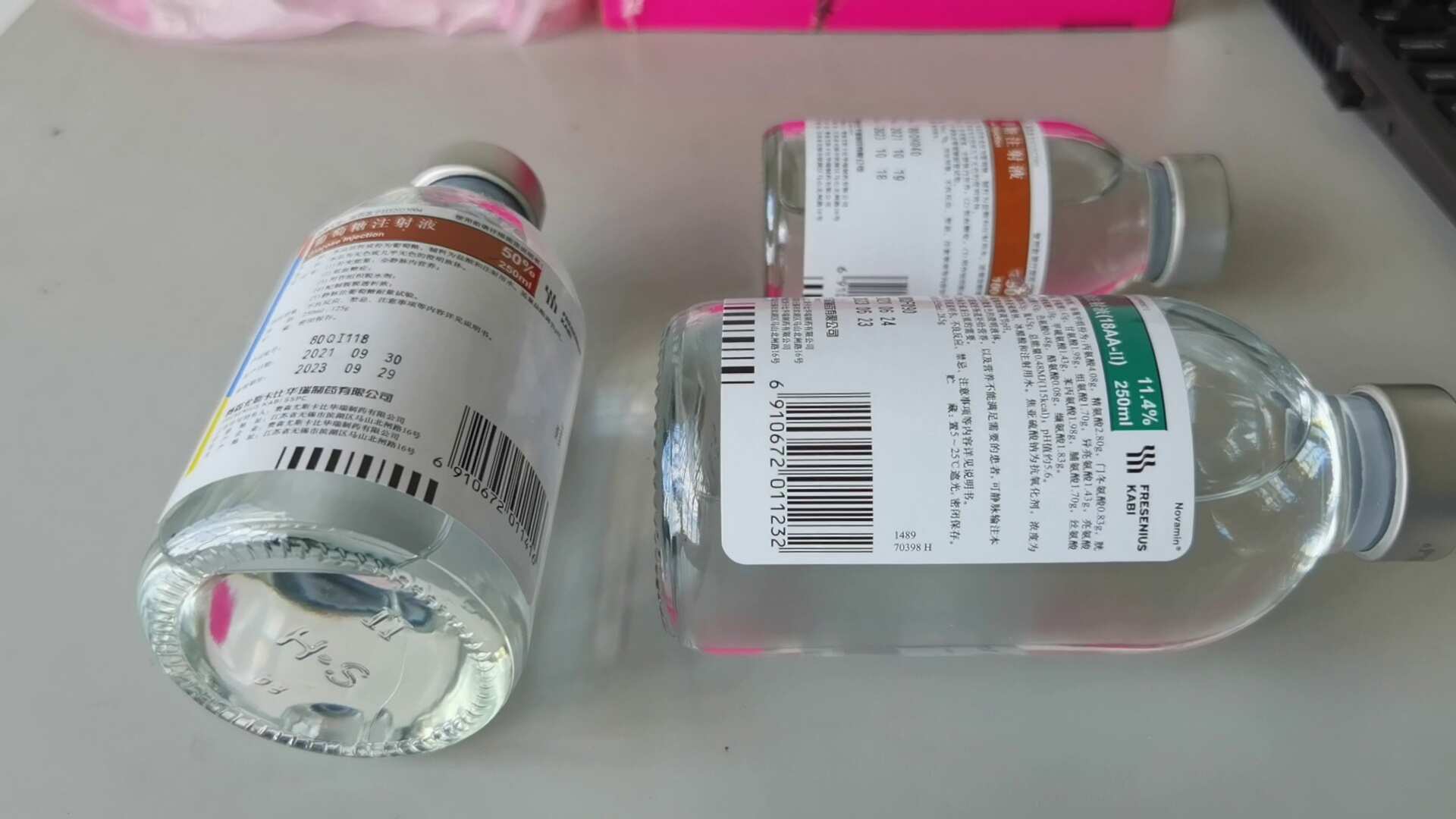

Supplement: S1 Dataset — (ZIP) [file pone.0298109.s001.zip › minimal data set/VOC2007/images/1050.jpg]

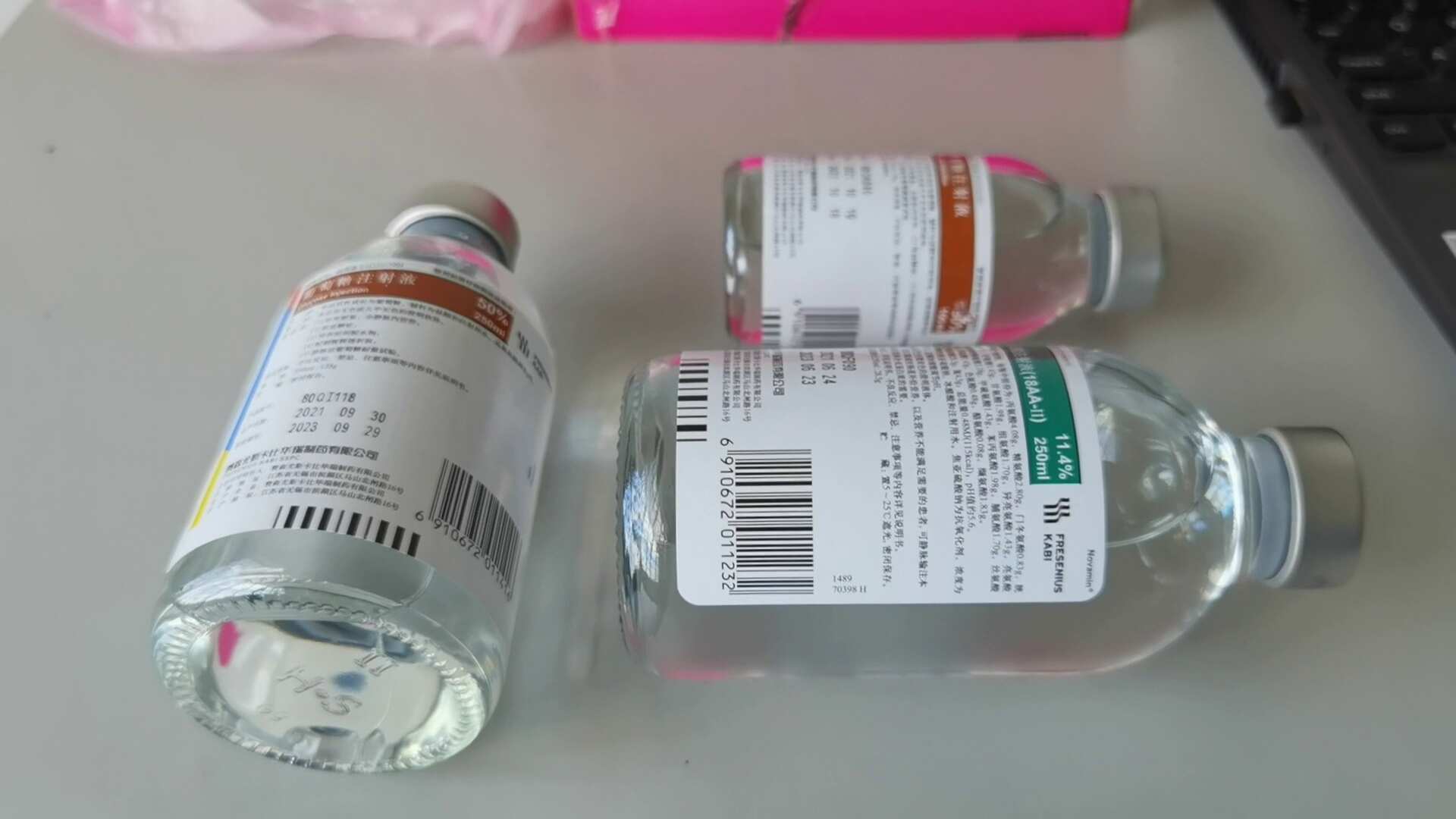

Supplement: S1 Dataset — (ZIP) [file pone.0298109.s001.zip › minimal data set/VOC2007/images/1051.jpg]

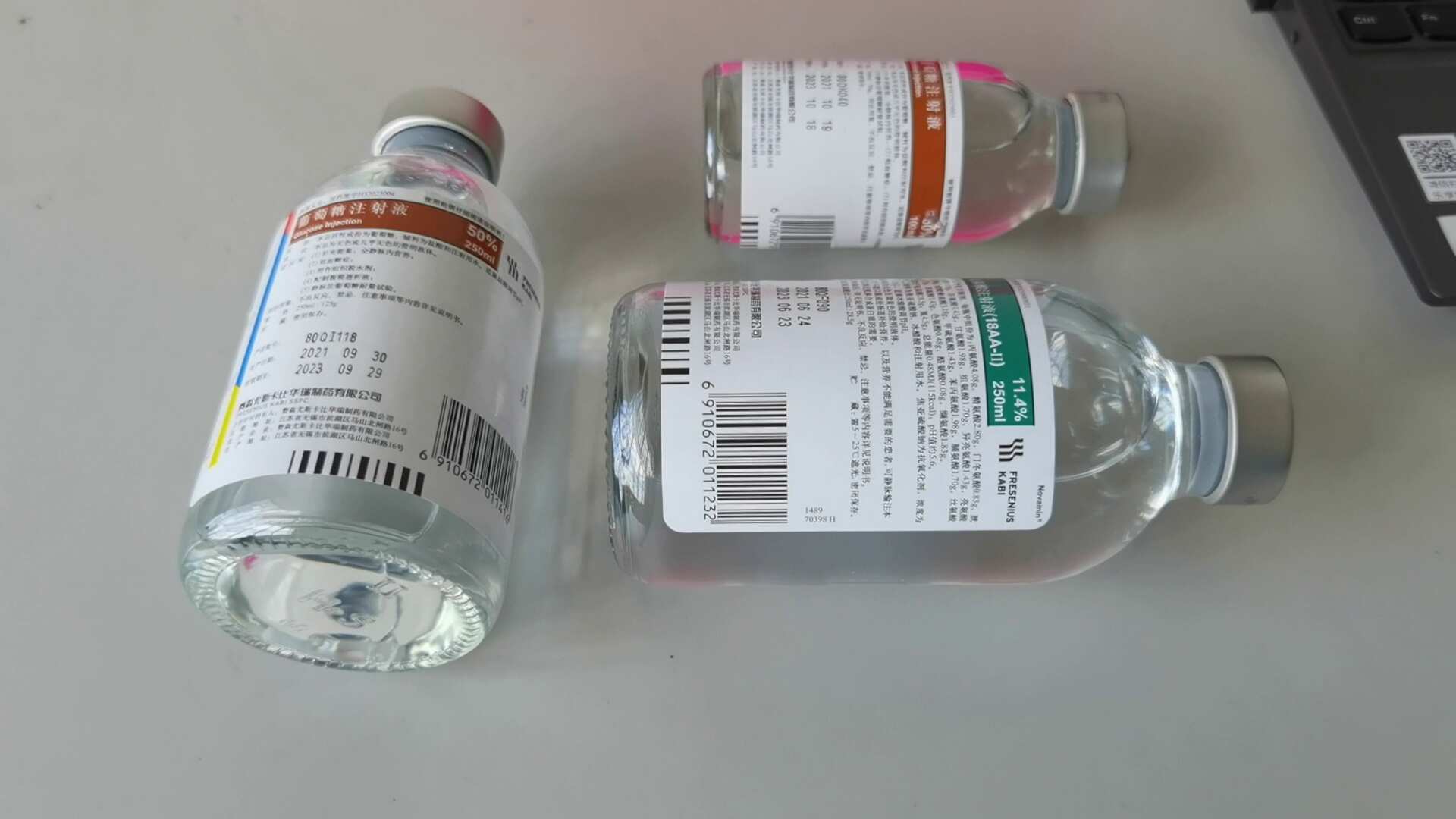

Supplement: S1 Dataset — (ZIP) [file pone.0298109.s001.zip › minimal data set/VOC2007/images/1052.jpg]

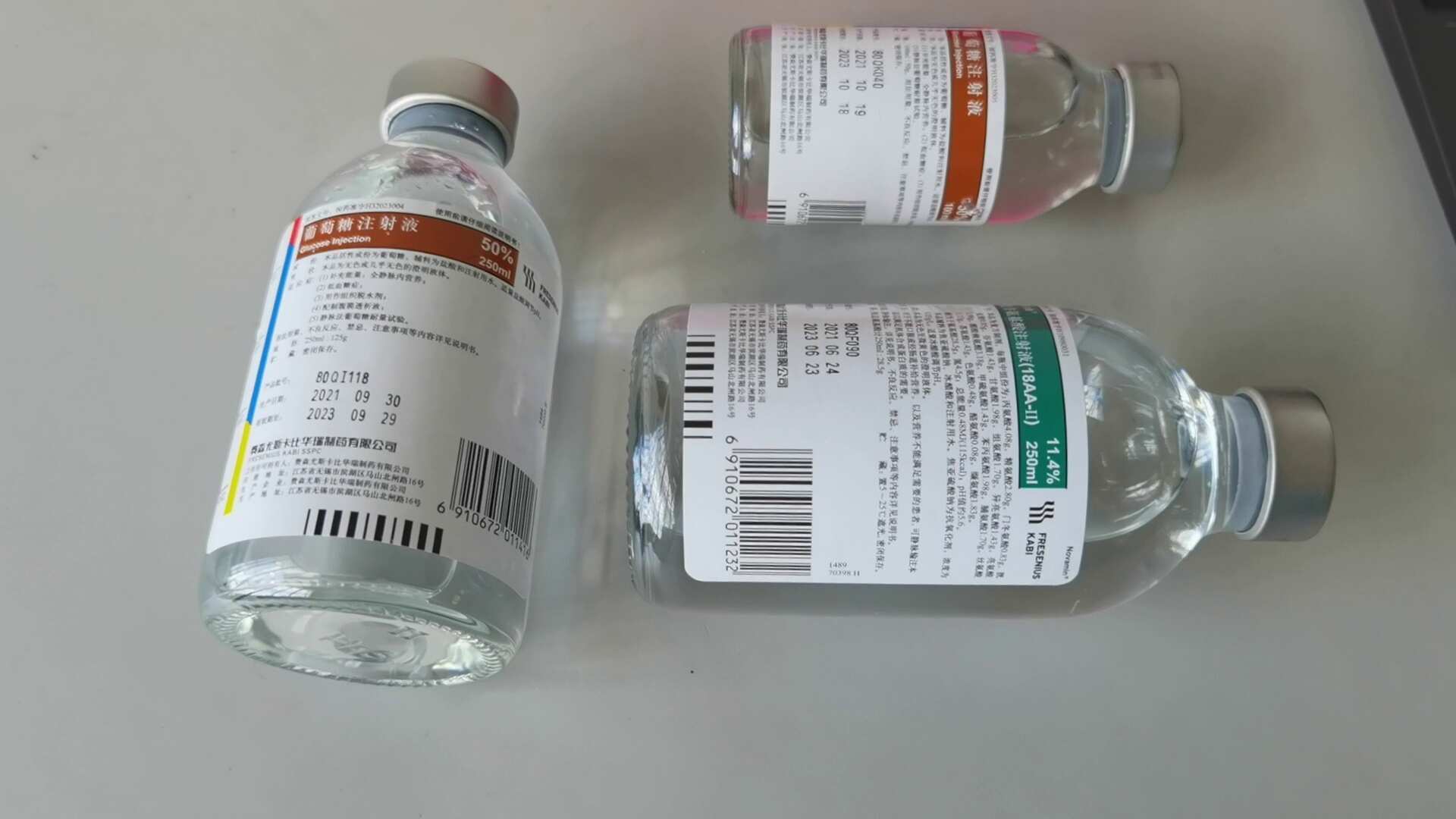

Supplement: S1 Dataset — (ZIP) [file pone.0298109.s001.zip › minimal data set/VOC2007/images/1053.jpg]

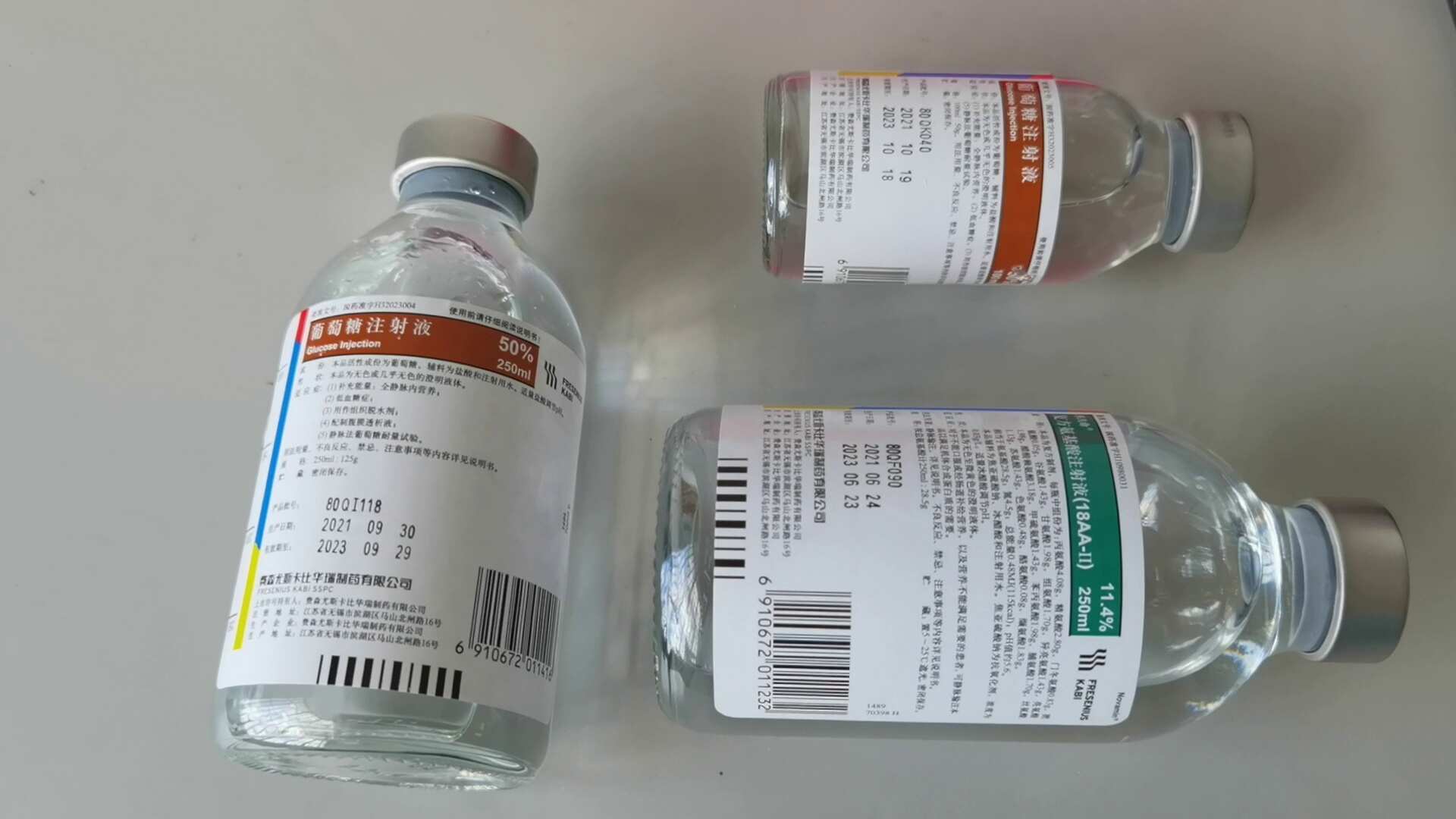

Supplement: S1 Dataset — (ZIP) [file pone.0298109.s001.zip › minimal data set/VOC2007/images/1054.jpg]

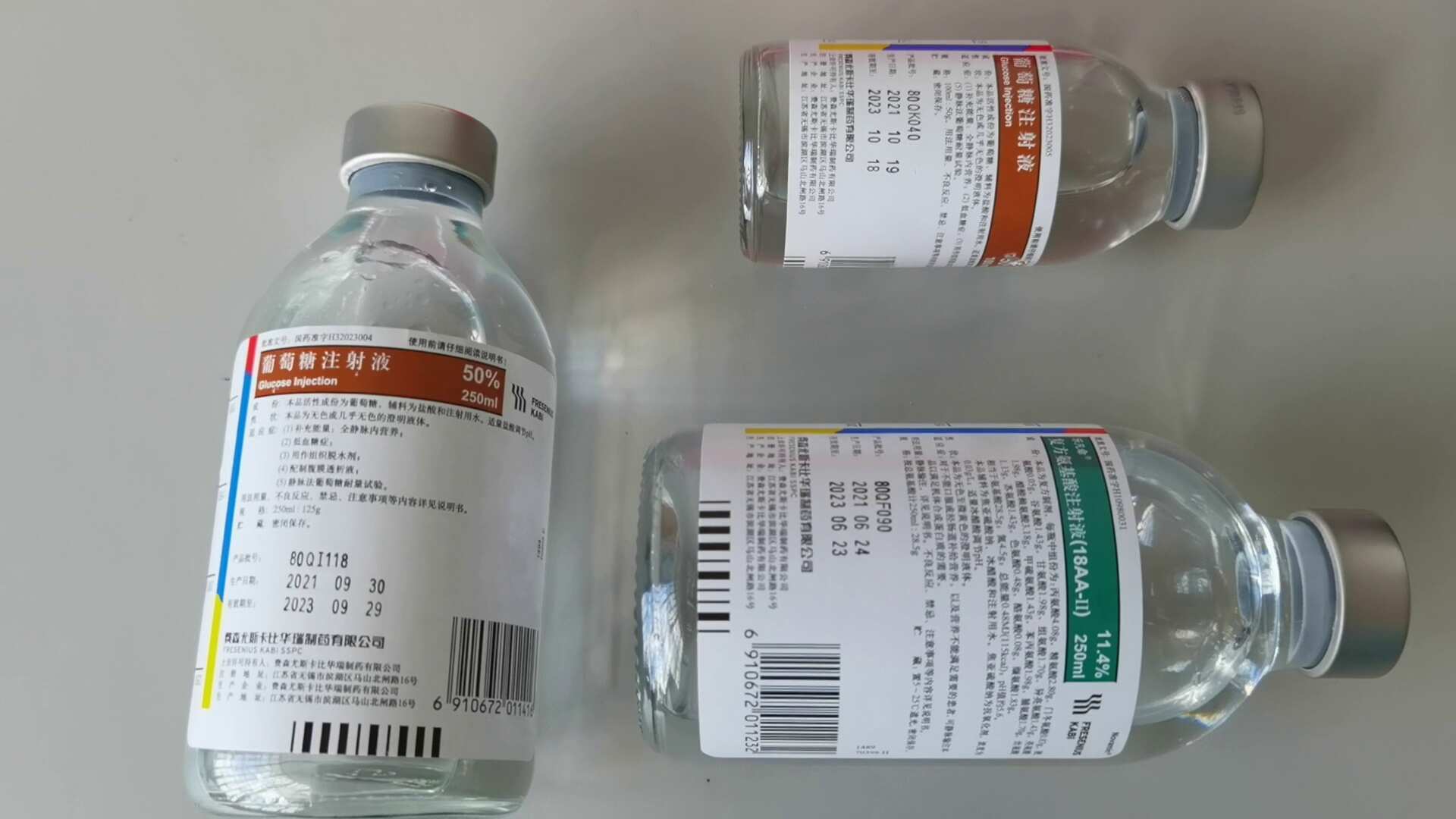

Supplement: S1 Dataset — (ZIP) [file pone.0298109.s001.zip › minimal data set/VOC2007/images/1055.jpg]

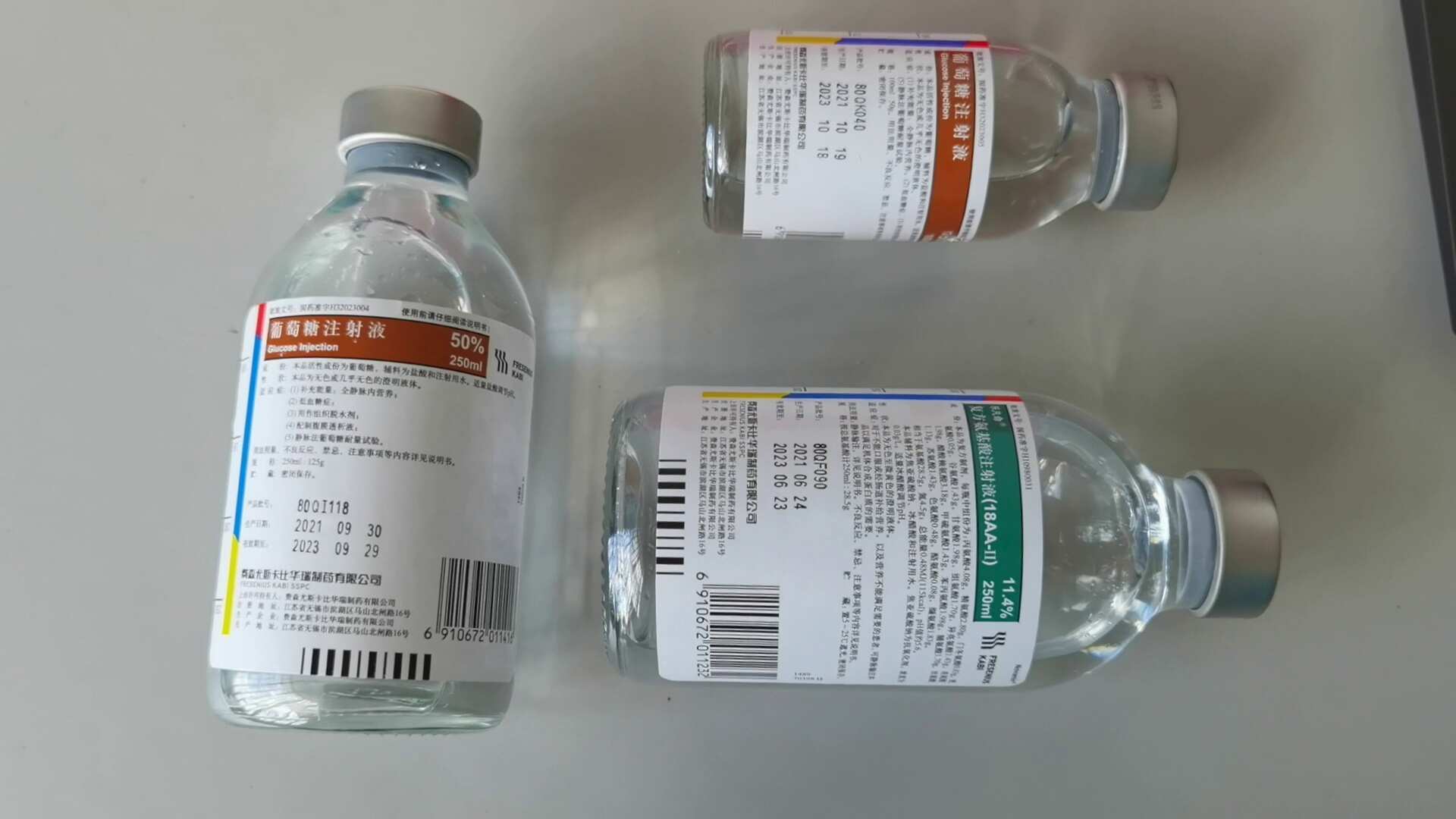

Supplement: S1 Dataset — (ZIP) [file pone.0298109.s001.zip › minimal data set/VOC2007/images/1056.jpg]

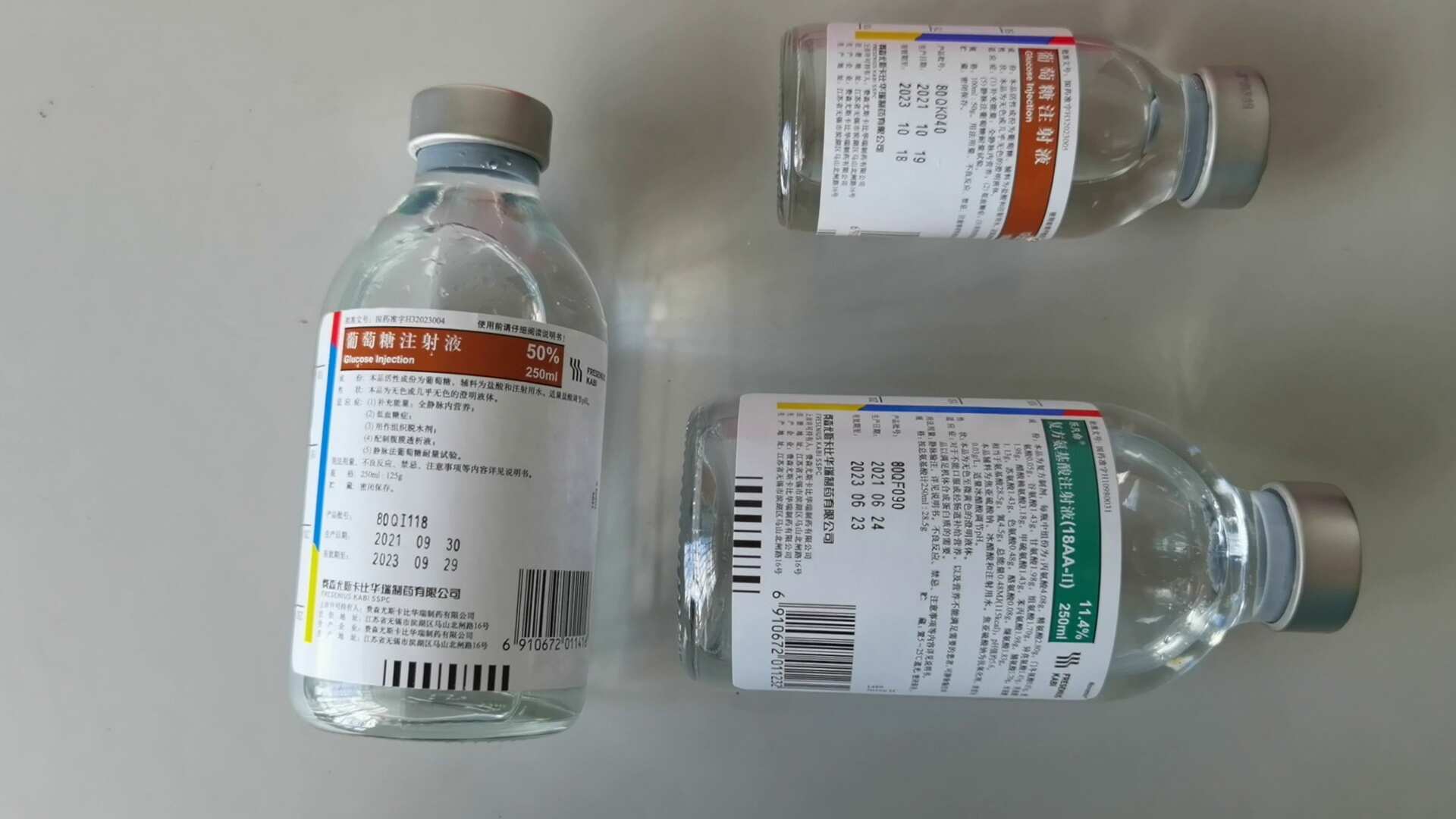

Supplement: S1 Dataset — (ZIP) [file pone.0298109.s001.zip › minimal data set/VOC2007/images/1057.jpg]

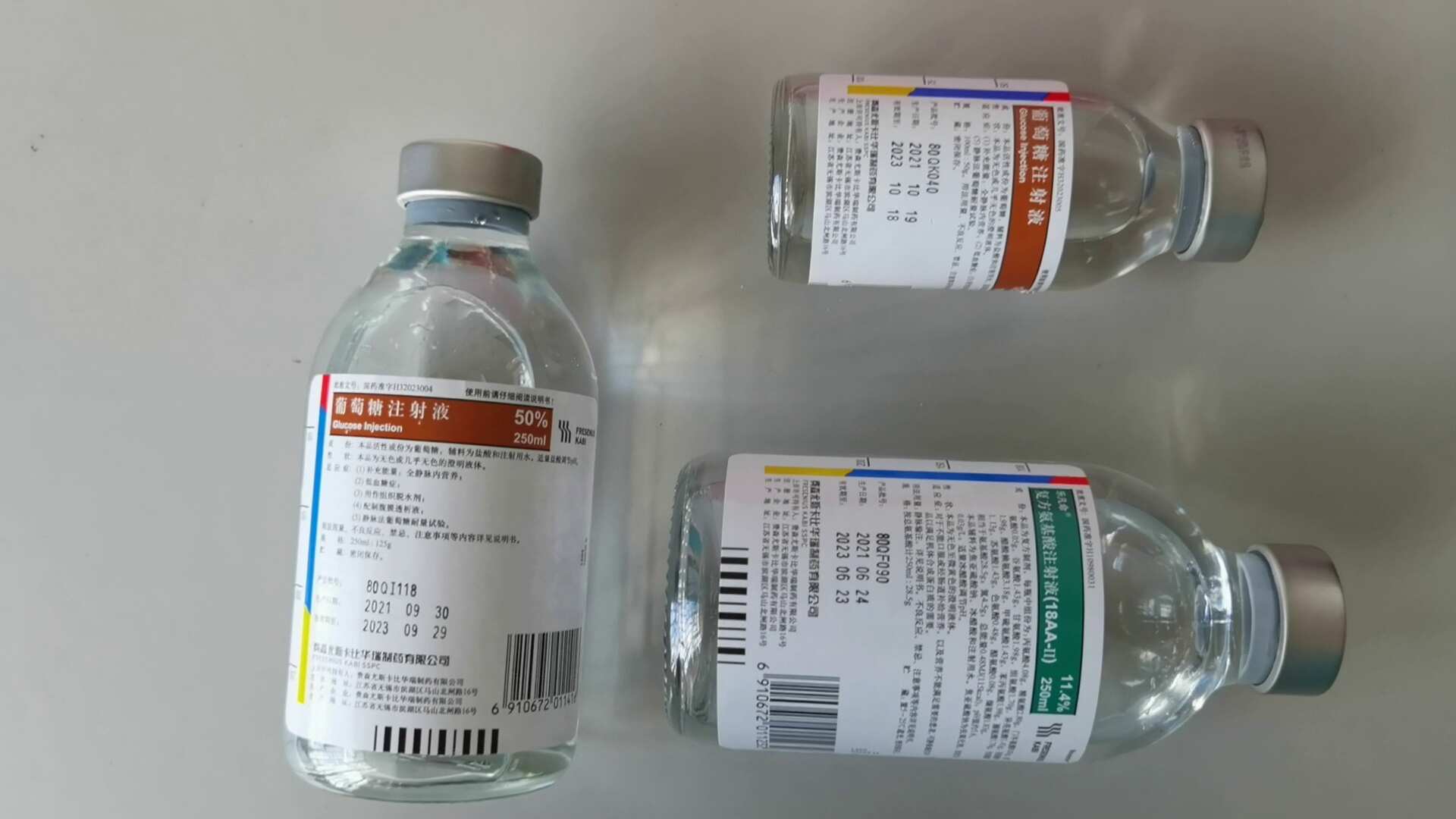

Supplement: S1 Dataset — (ZIP) [file pone.0298109.s001.zip › minimal data set/VOC2007/images/1058.jpg]

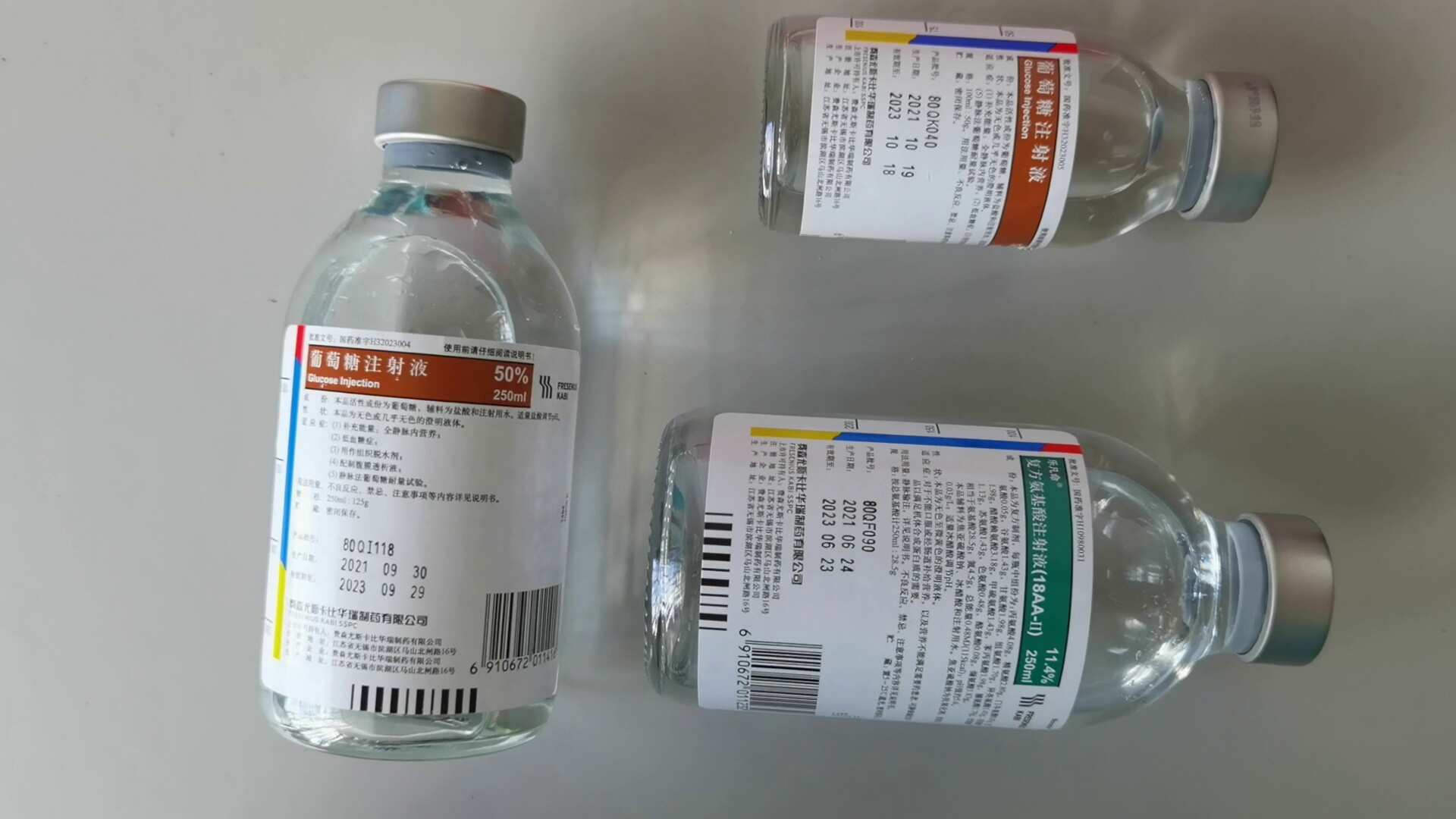

Supplement: S1 Dataset — (ZIP) [file pone.0298109.s001.zip › minimal data set/VOC2007/images/1059.jpg]

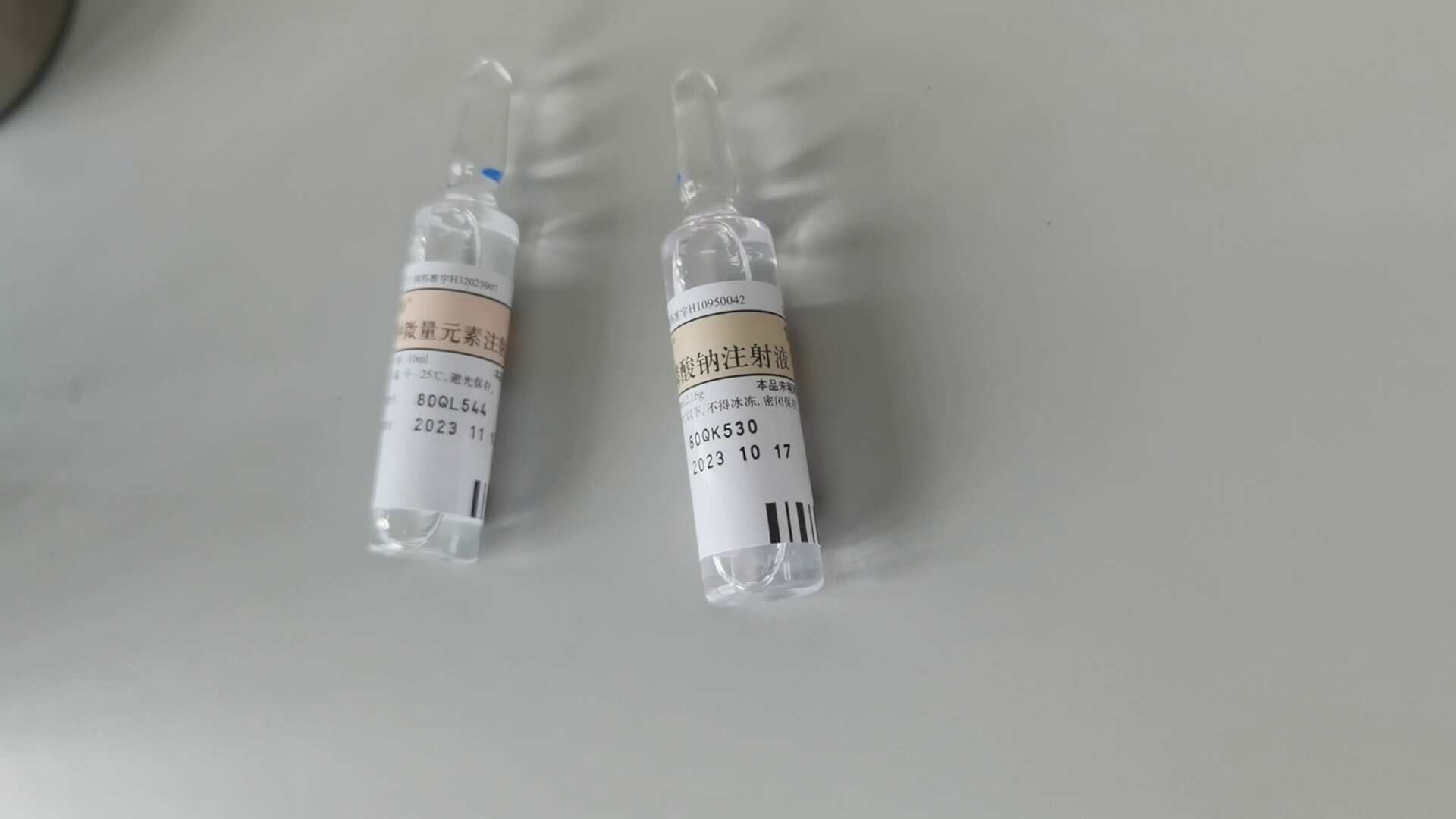

Supplement: S1 Dataset — (ZIP) [file pone.0298109.s001.zip › minimal data set/VOC2007/images/106.jpg]

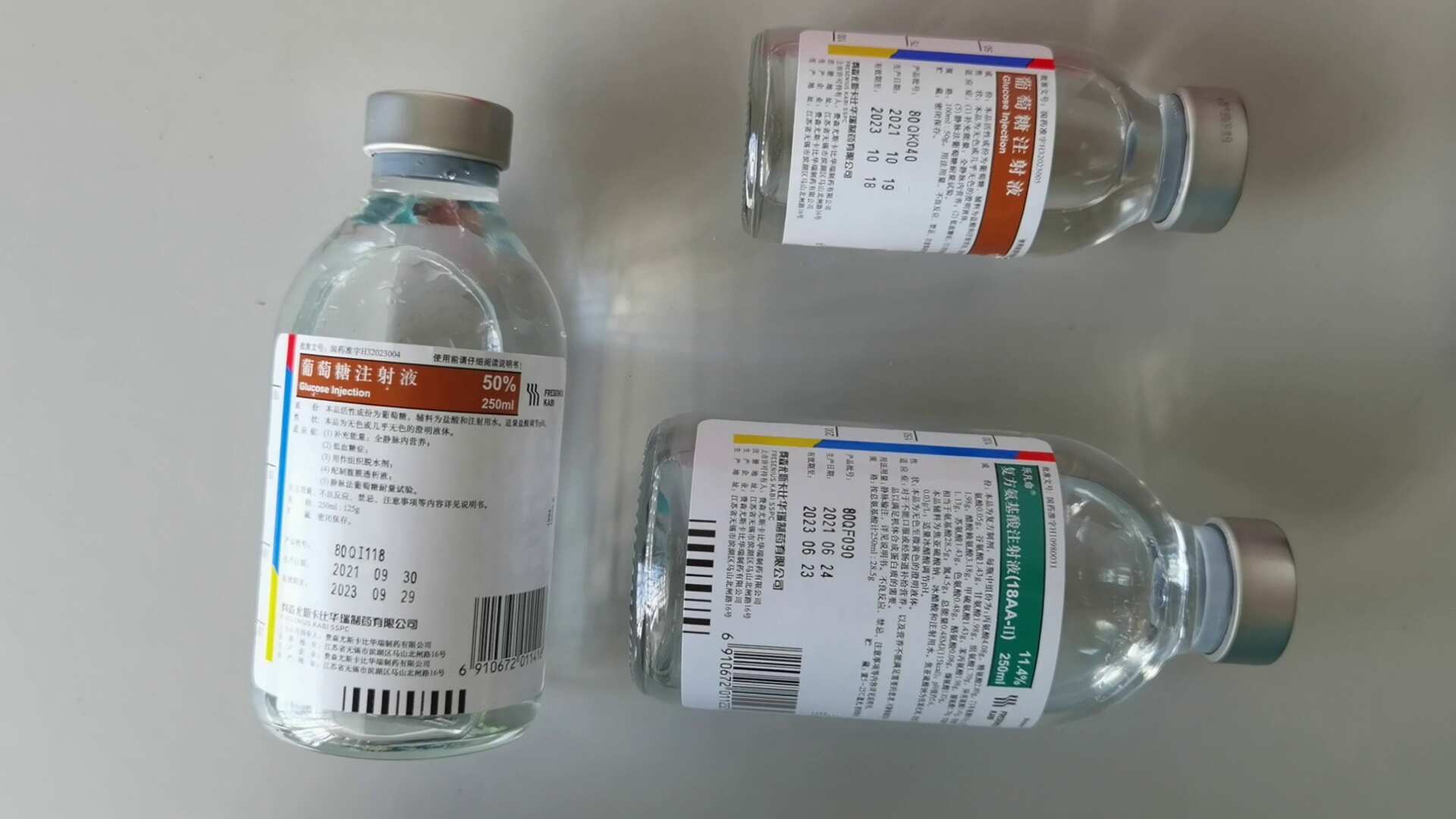

Supplement: S1 Dataset — (ZIP) [file pone.0298109.s001.zip › minimal data set/VOC2007/images/1060.jpg]

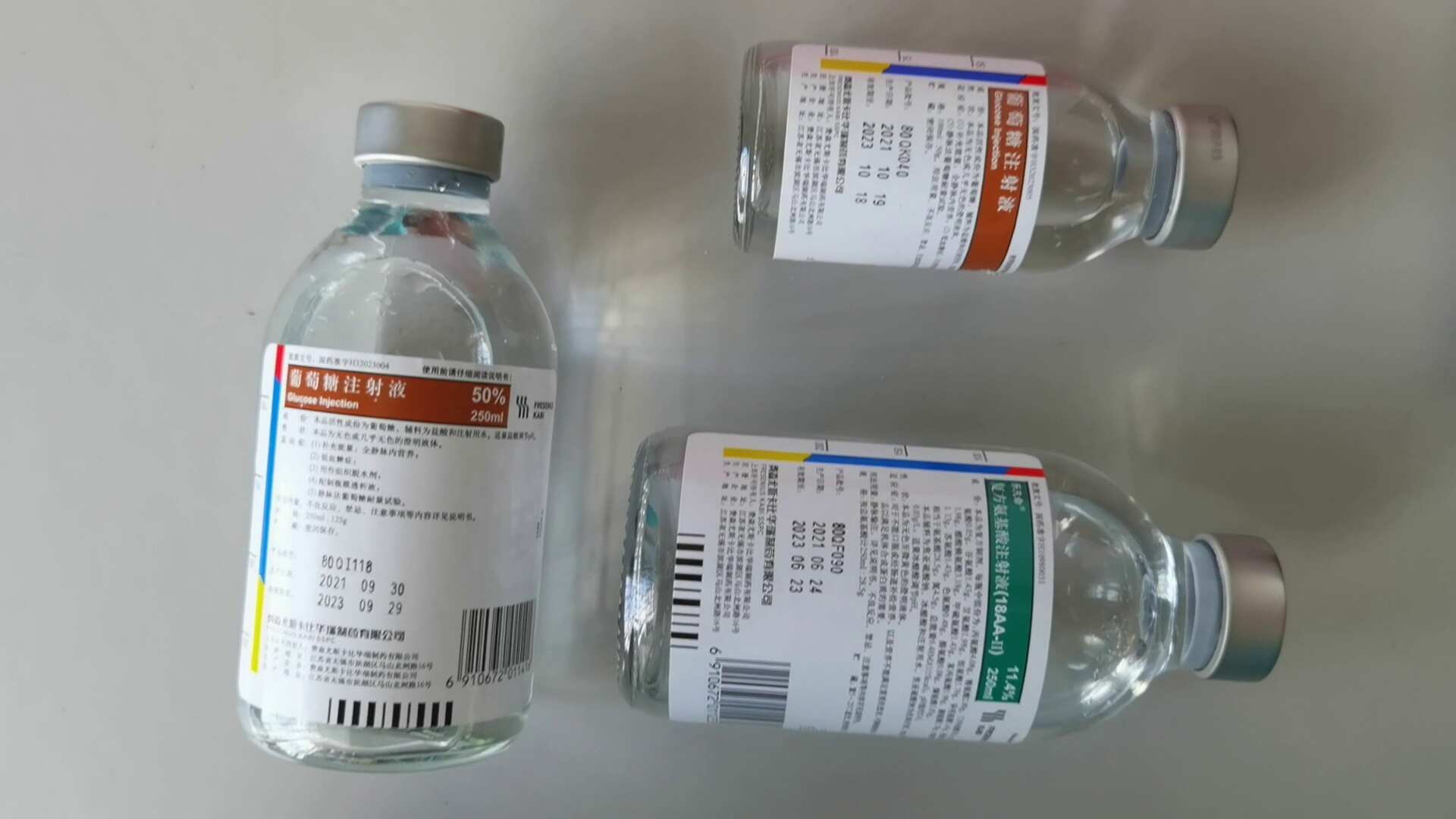

Supplement: S1 Dataset — (ZIP) [file pone.0298109.s001.zip › minimal data set/VOC2007/images/1061.jpg]

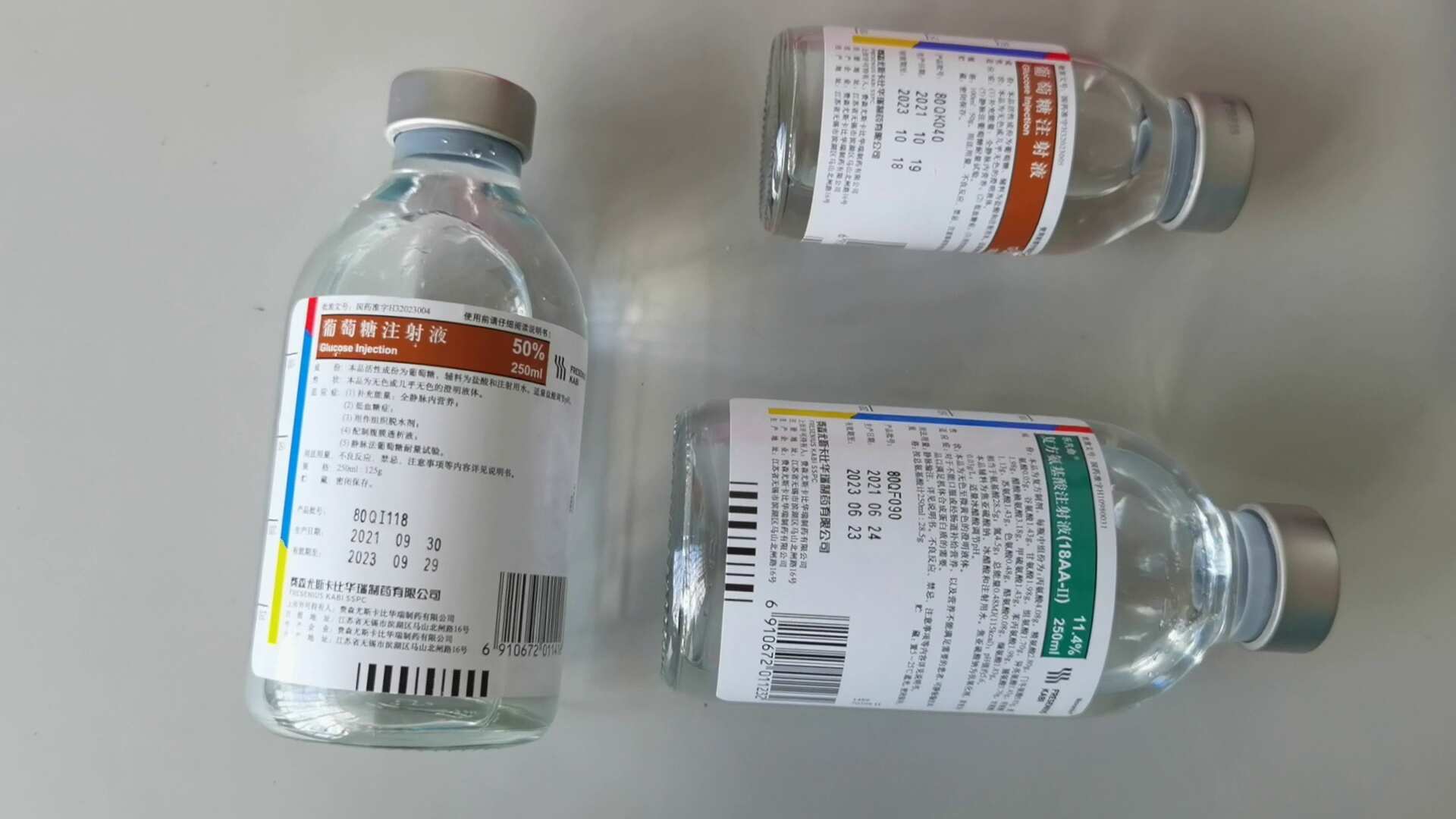

Supplement: S1 Dataset — (ZIP) [file pone.0298109.s001.zip › minimal data set/VOC2007/images/1062.jpg]

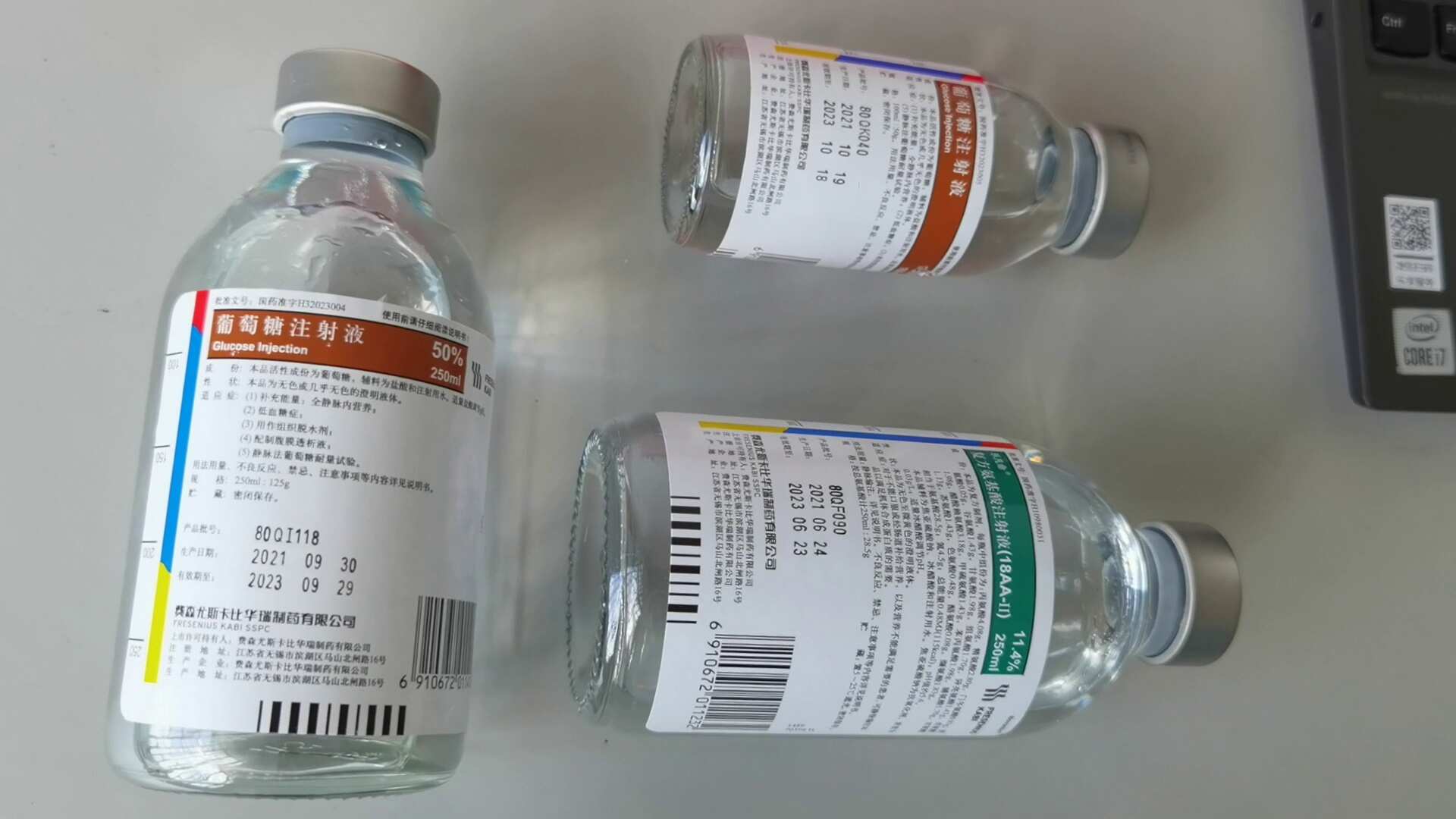

Supplement: S1 Dataset — (ZIP) [file pone.0298109.s001.zip › minimal data set/VOC2007/images/1063.jpg]

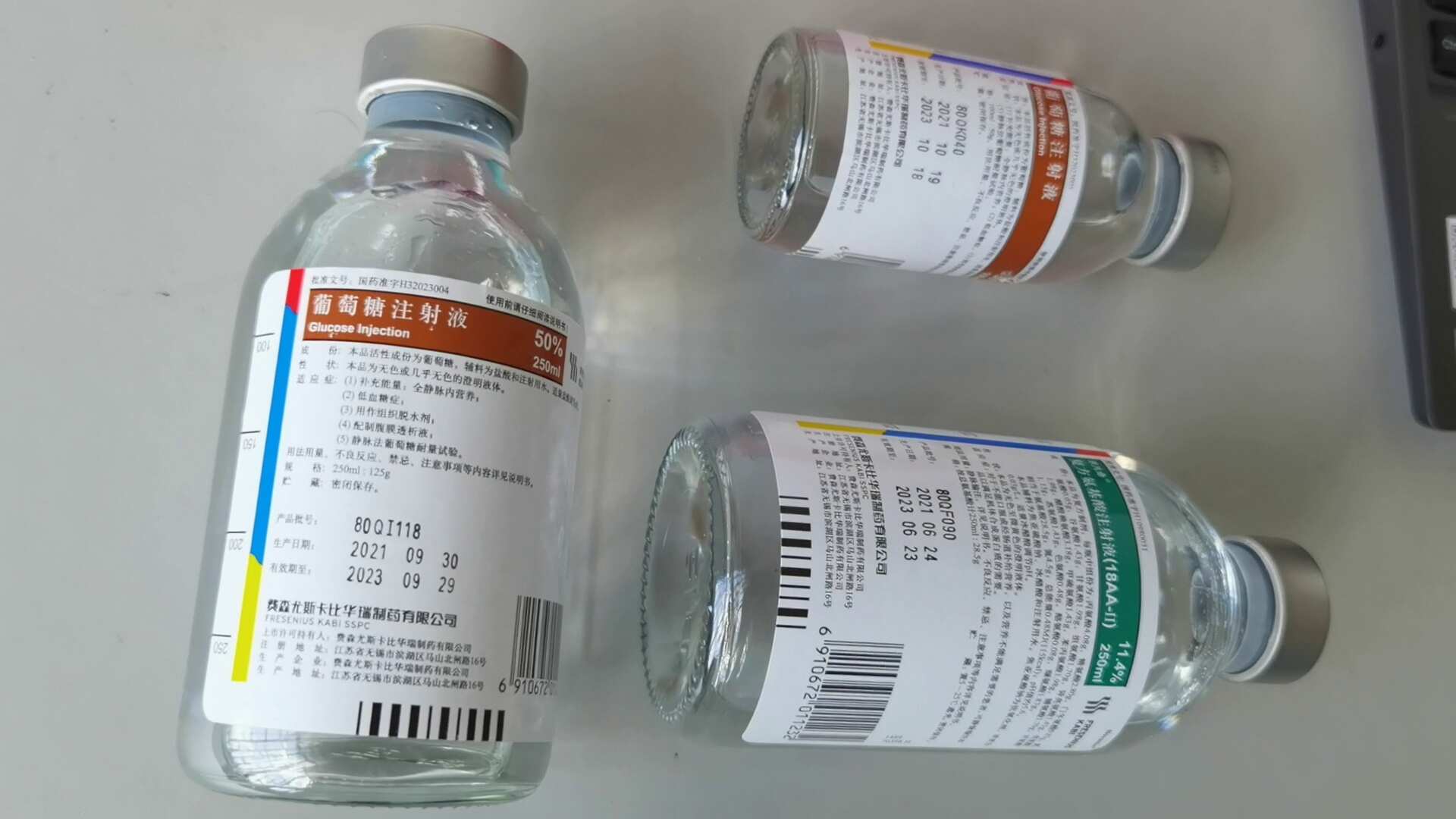

Supplement: S1 Dataset — (ZIP) [file pone.0298109.s001.zip › minimal data set/VOC2007/images/1064.jpg]

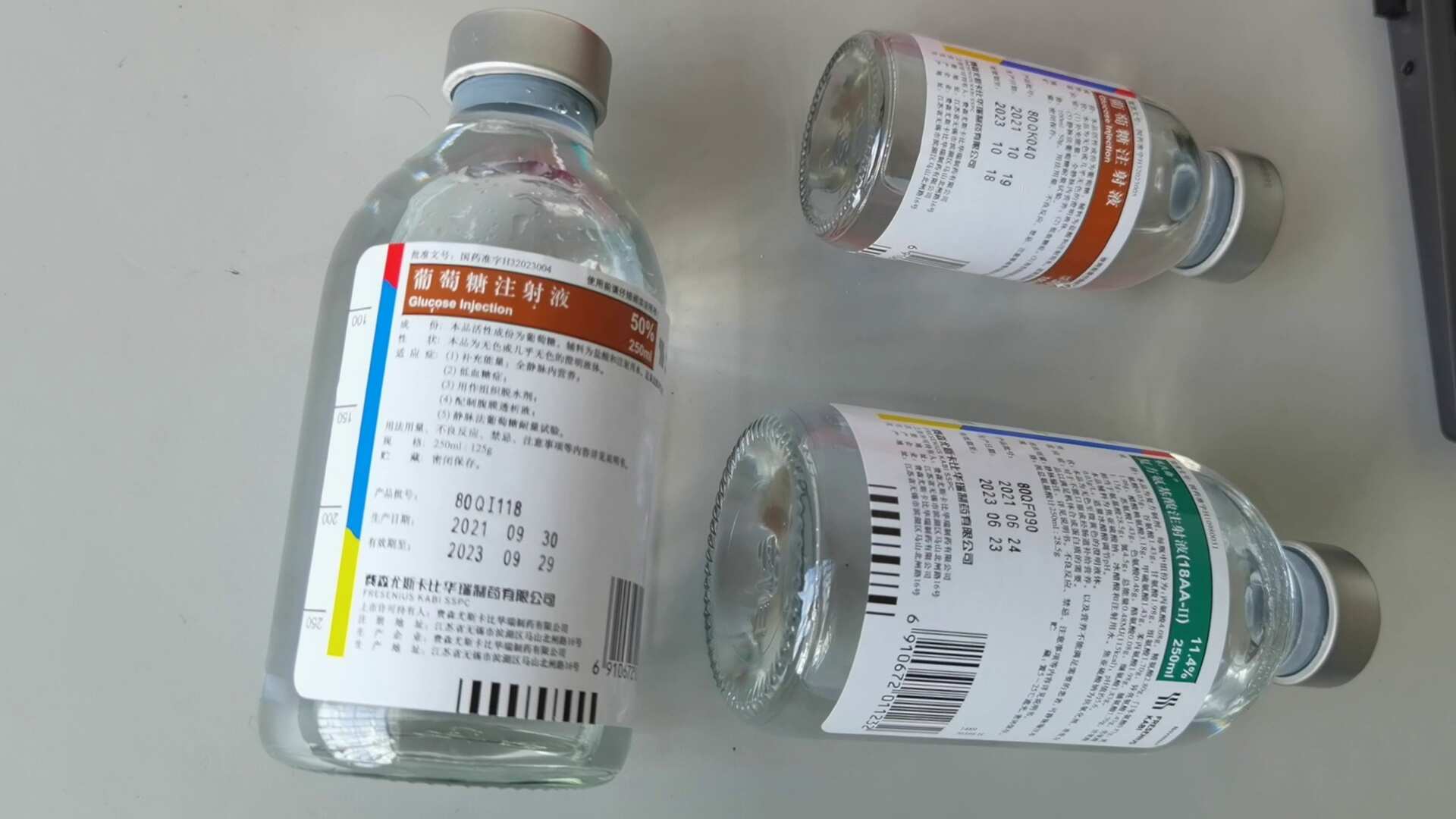

Supplement: S1 Dataset — (ZIP) [file pone.0298109.s001.zip › minimal data set/VOC2007/images/1065.jpg]

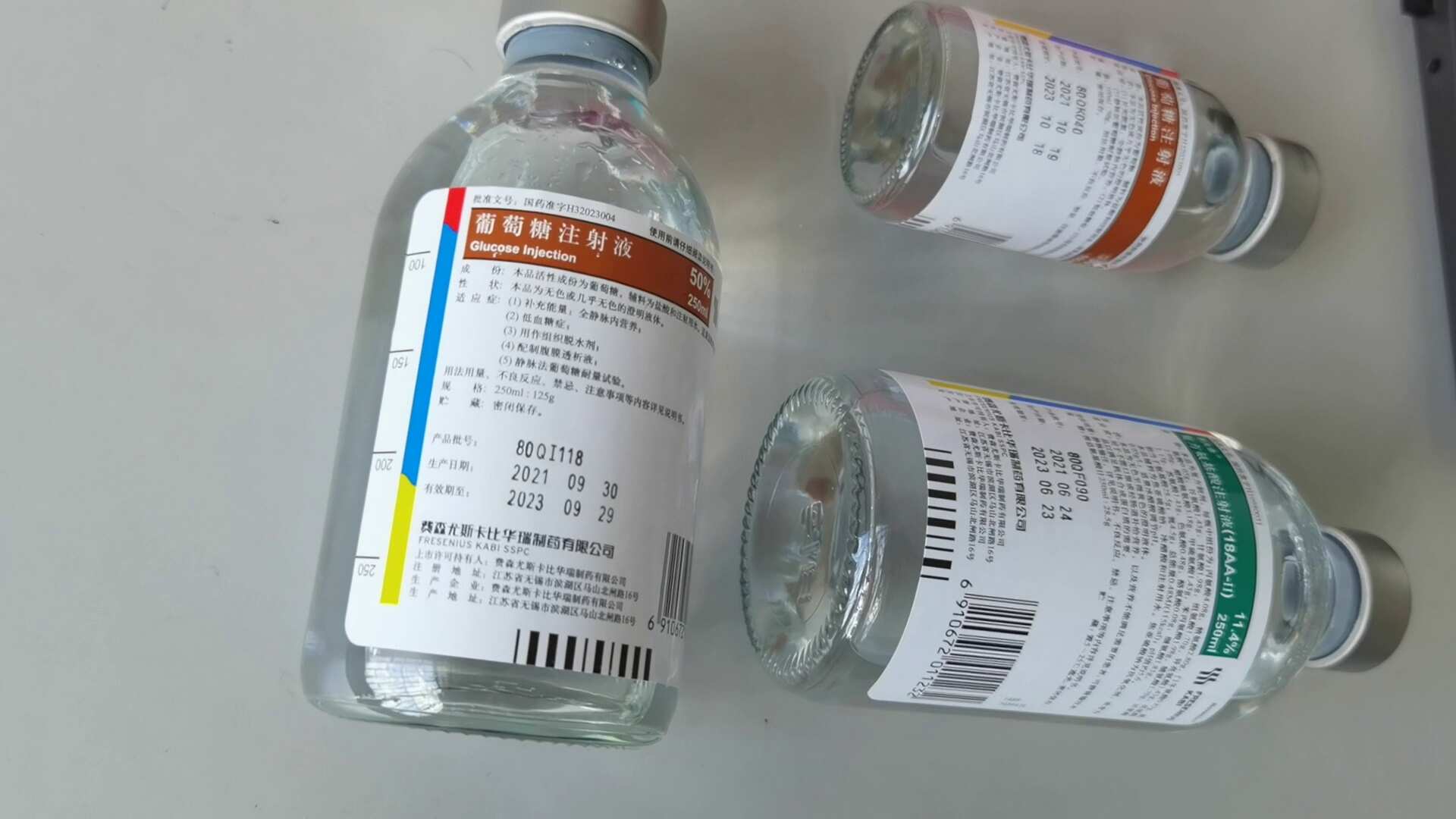

Supplement: S1 Dataset — (ZIP) [file pone.0298109.s001.zip › minimal data set/VOC2007/images/1066.jpg]

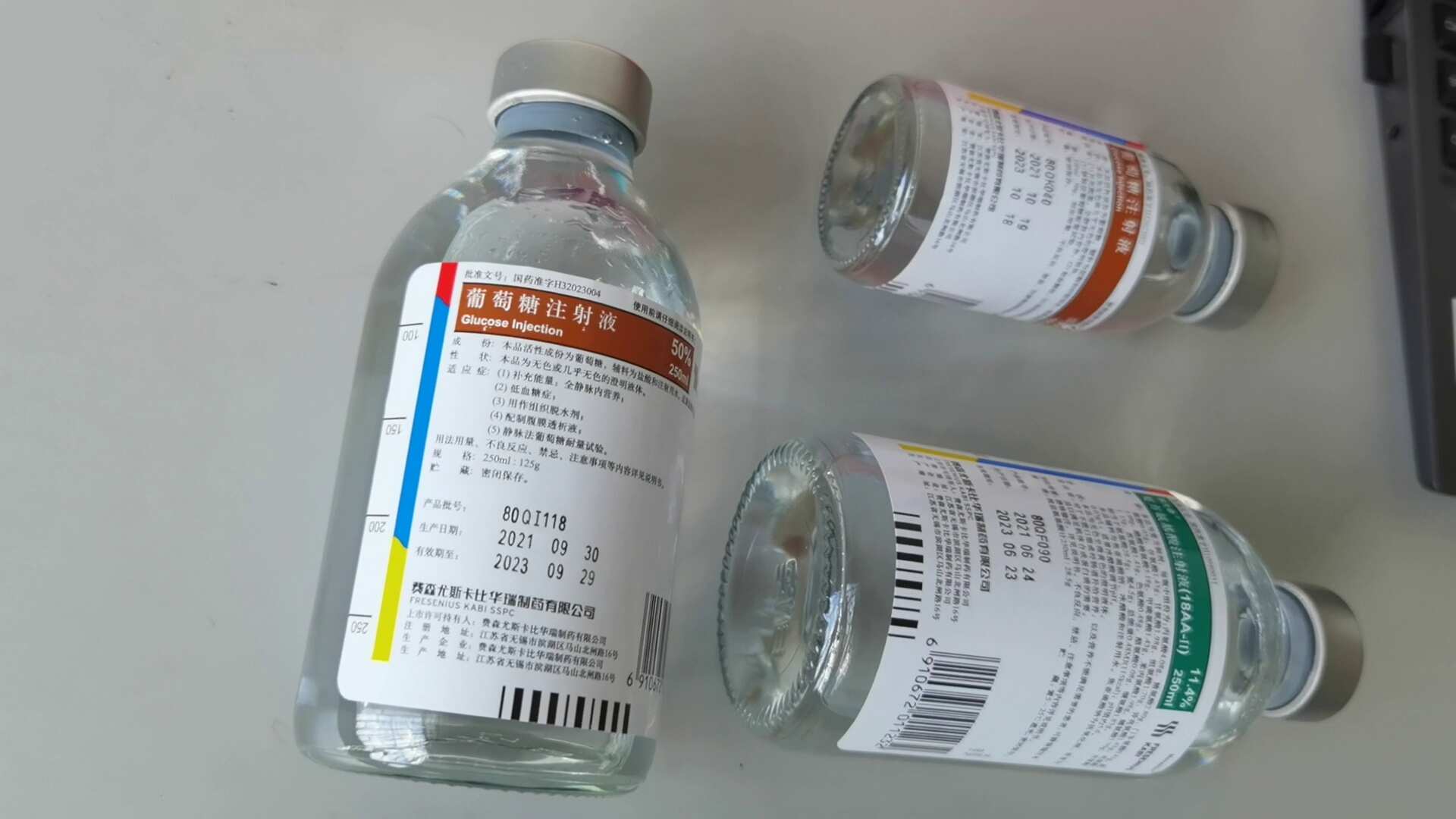

Supplement: S1 Dataset — (ZIP) [file pone.0298109.s001.zip › minimal data set/VOC2007/images/1067.jpg]

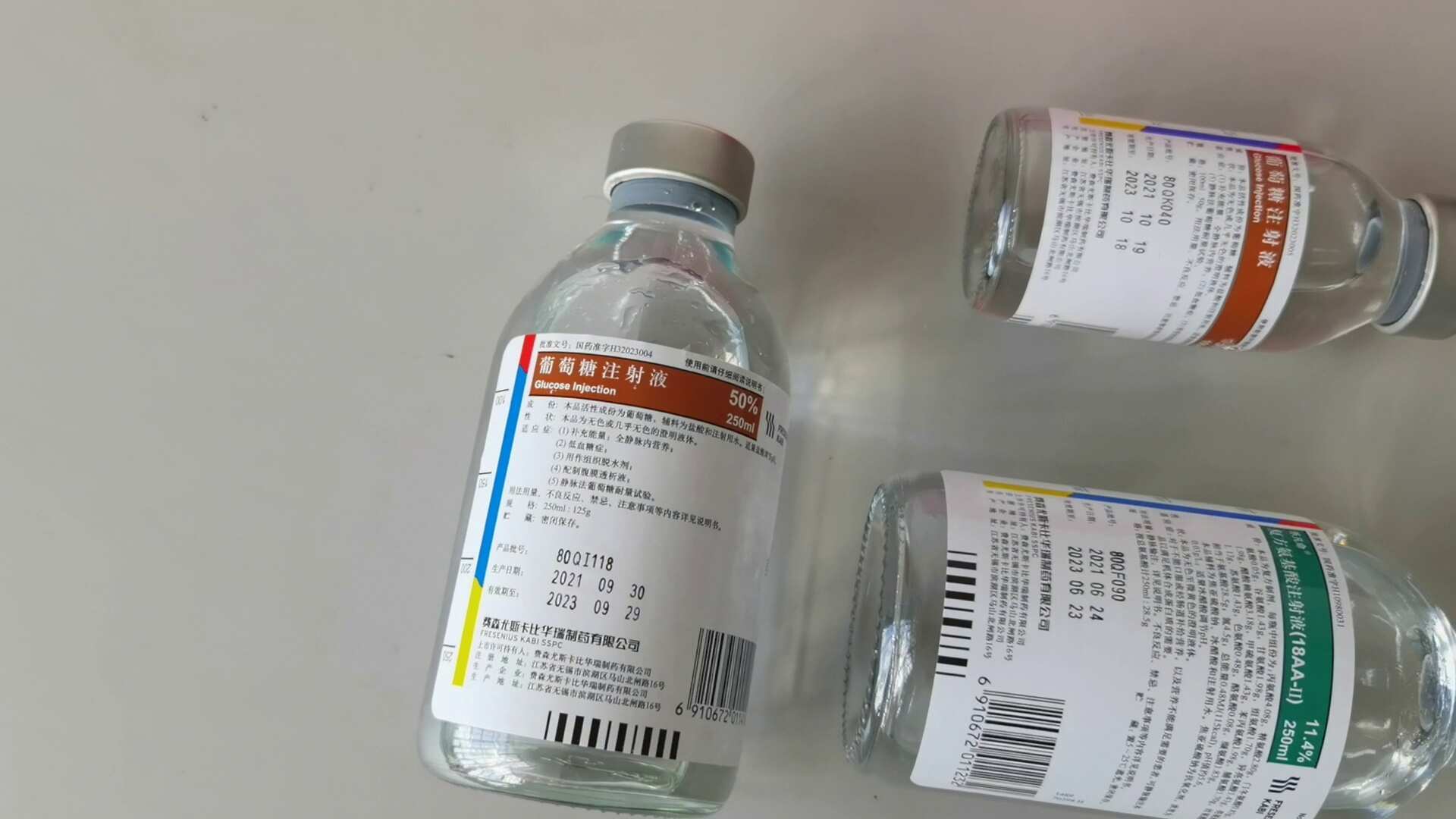

Supplement: S1 Dataset — (ZIP) [file pone.0298109.s001.zip › minimal data set/VOC2007/images/1068.jpg]

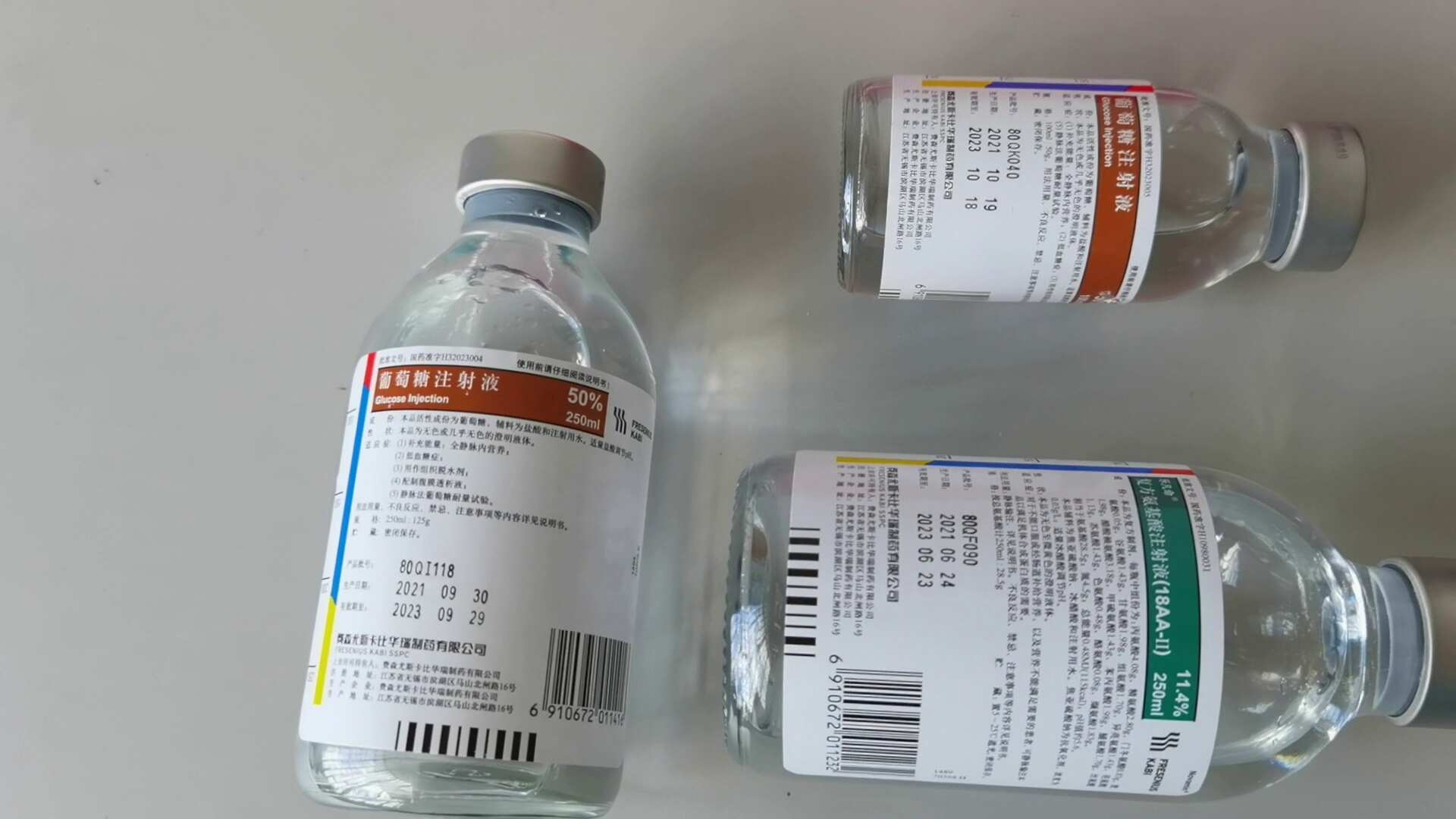

Supplement: S1 Dataset — (ZIP) [file pone.0298109.s001.zip › minimal data set/VOC2007/images/1069.jpg]

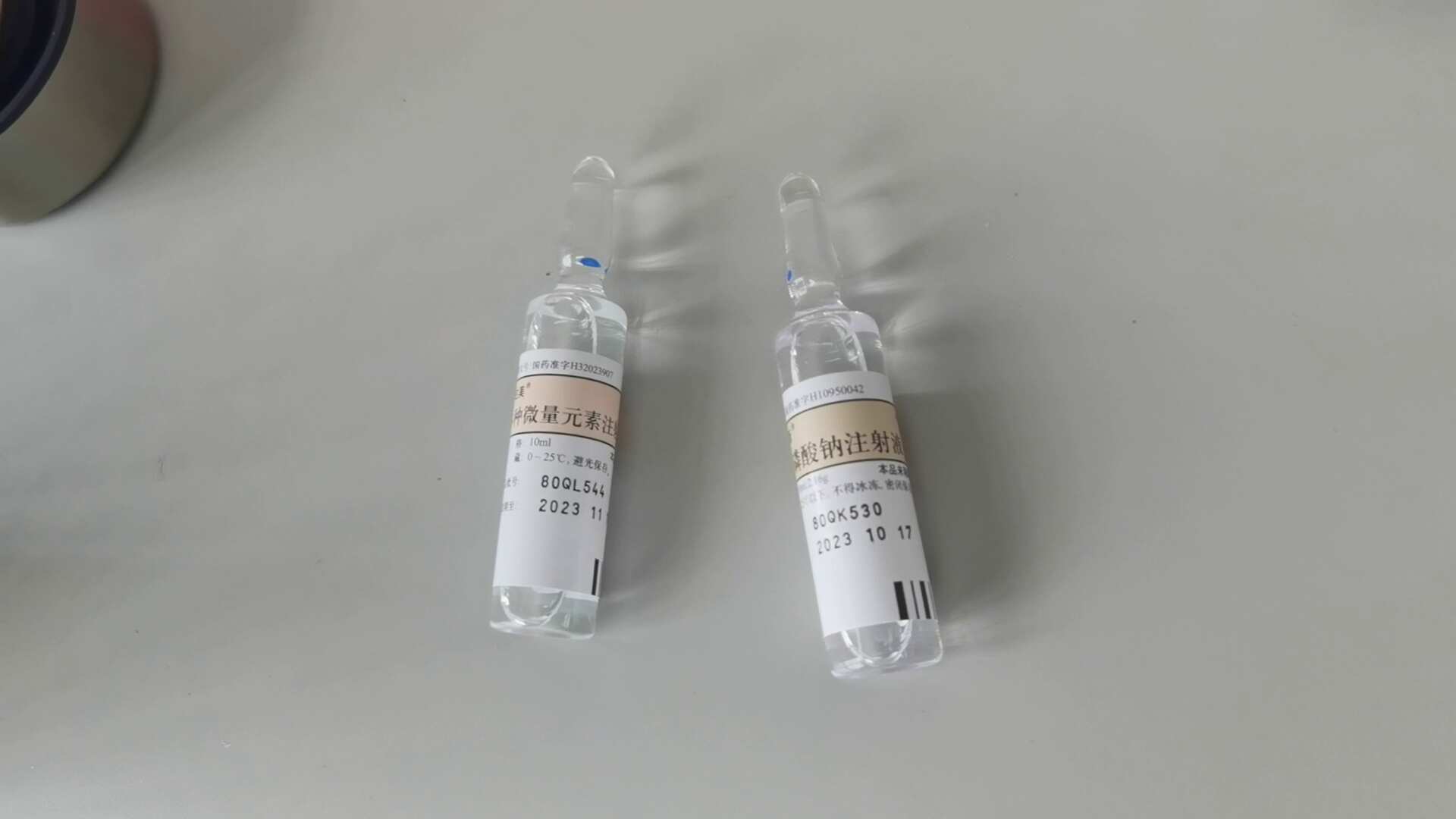

Supplement: S1 Dataset — (ZIP) [file pone.0298109.s001.zip › minimal data set/VOC2007/images/107.jpg]

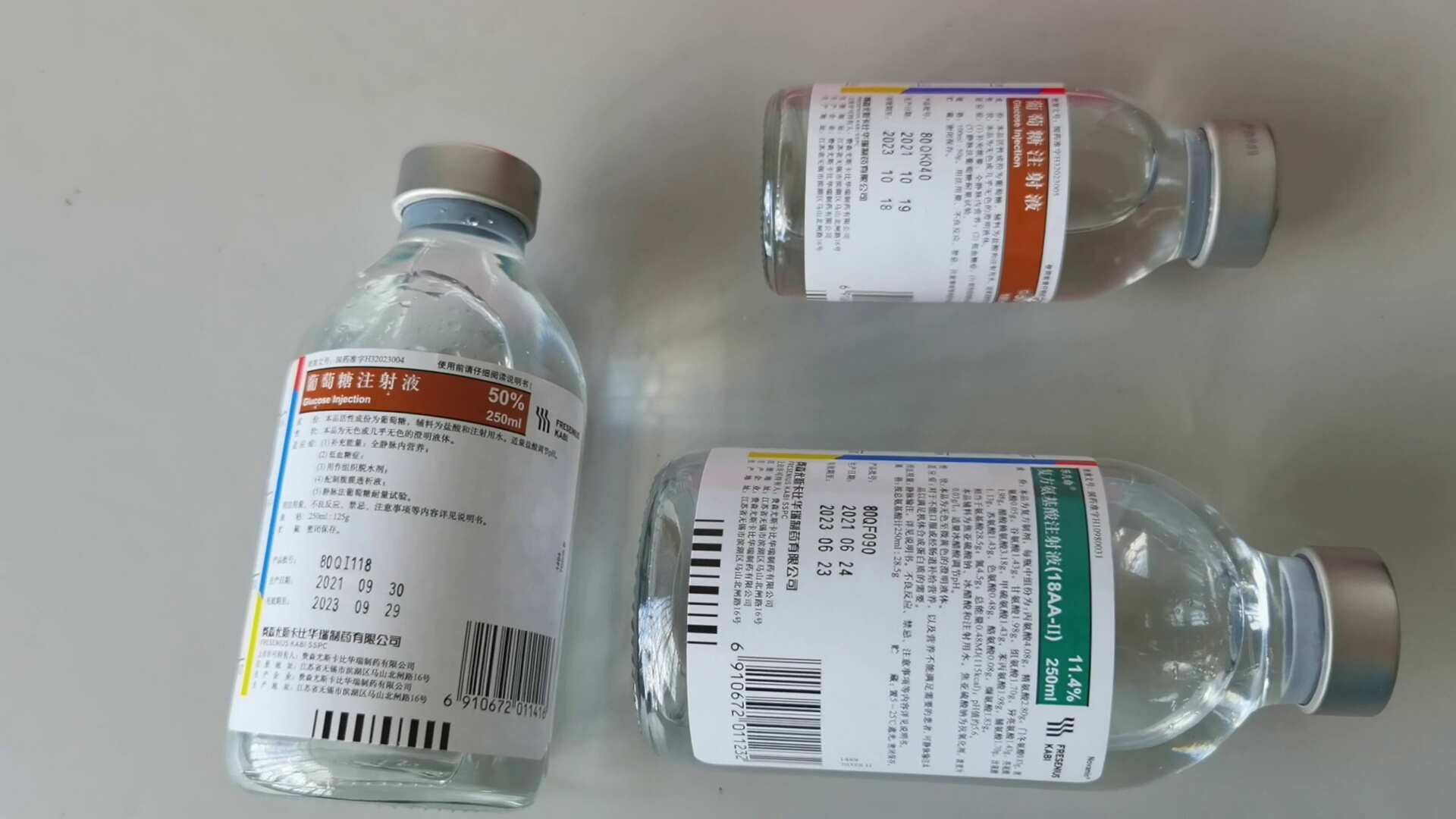

Supplement: S1 Dataset — (ZIP) [file pone.0298109.s001.zip › minimal data set/VOC2007/images/1070.jpg]

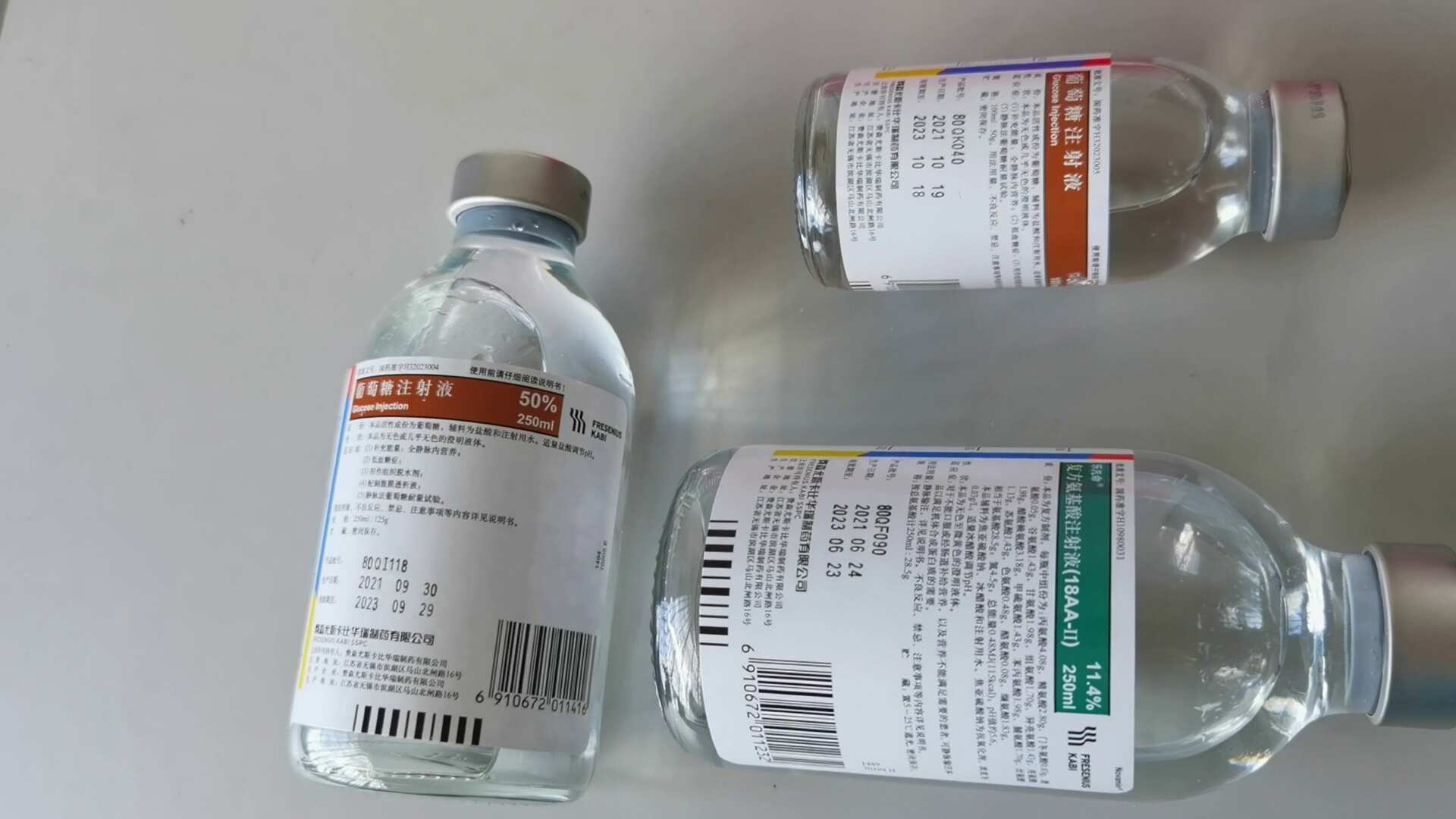

Supplement: S1 Dataset — (ZIP) [file pone.0298109.s001.zip › minimal data set/VOC2007/images/1071.jpg]

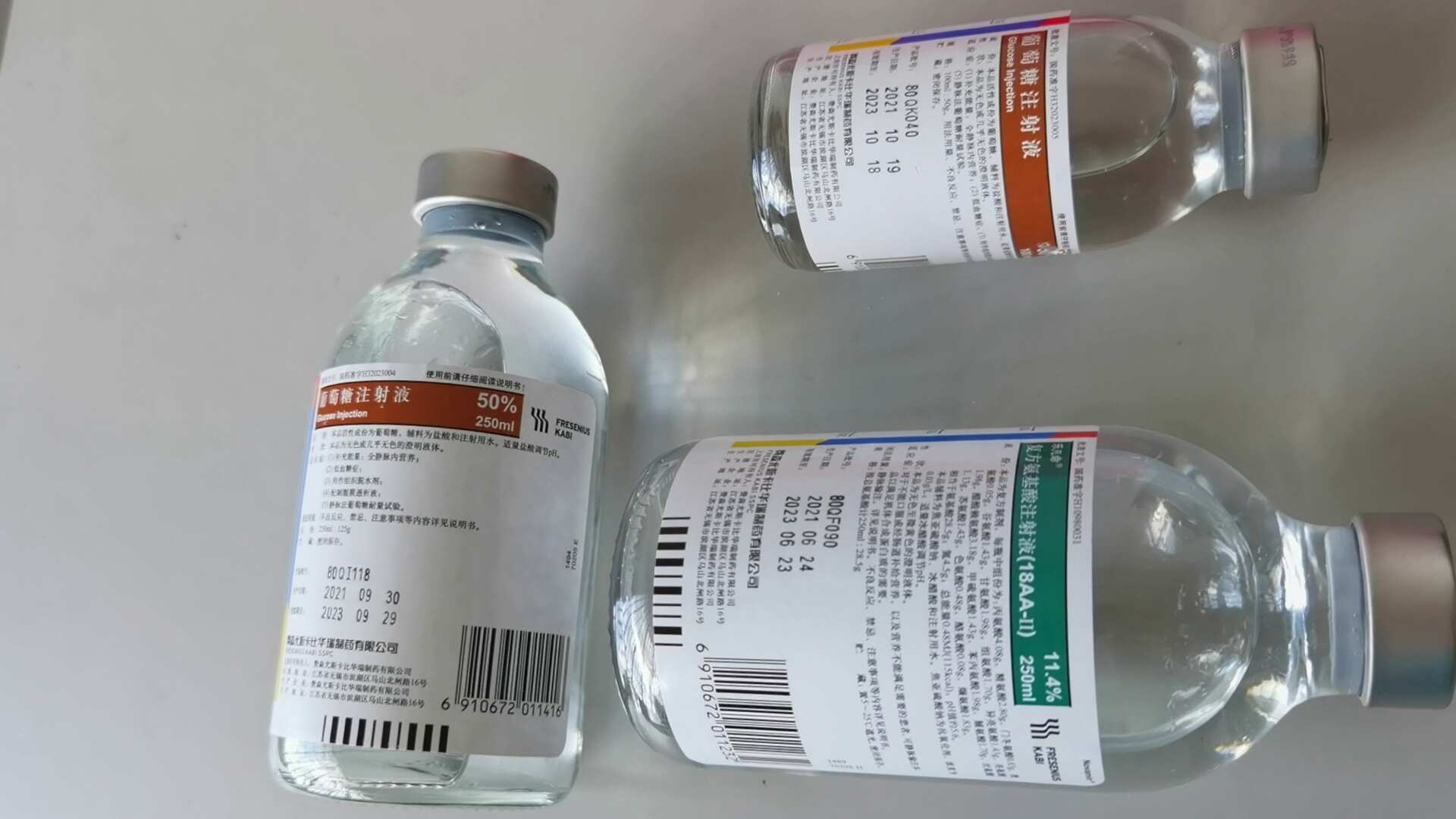

Supplement: S1 Dataset — (ZIP) [file pone.0298109.s001.zip › minimal data set/VOC2007/images/1072.jpg]

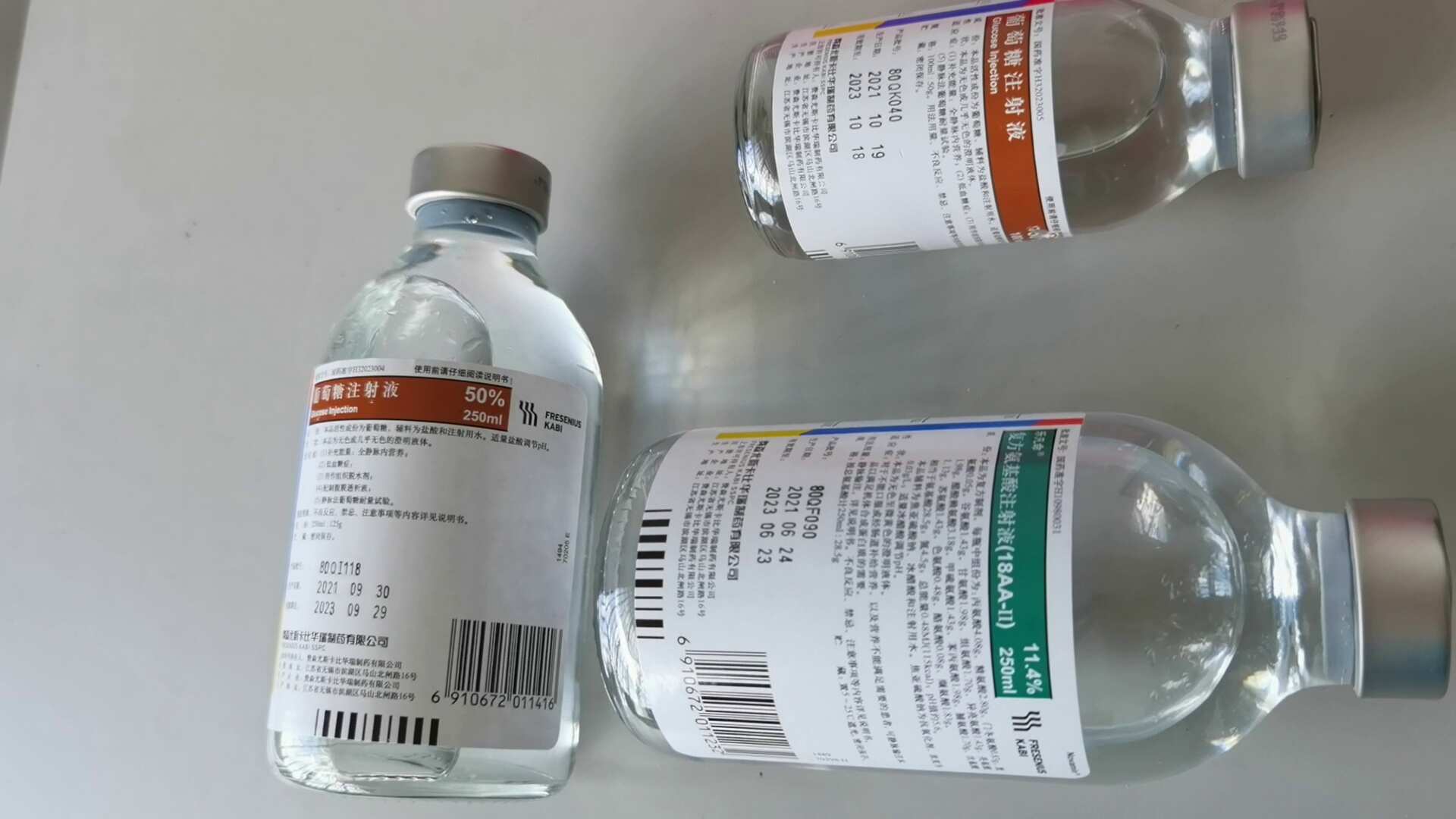

Supplement: S1 Dataset — (ZIP) [file pone.0298109.s001.zip › minimal data set/VOC2007/images/1073.jpg]

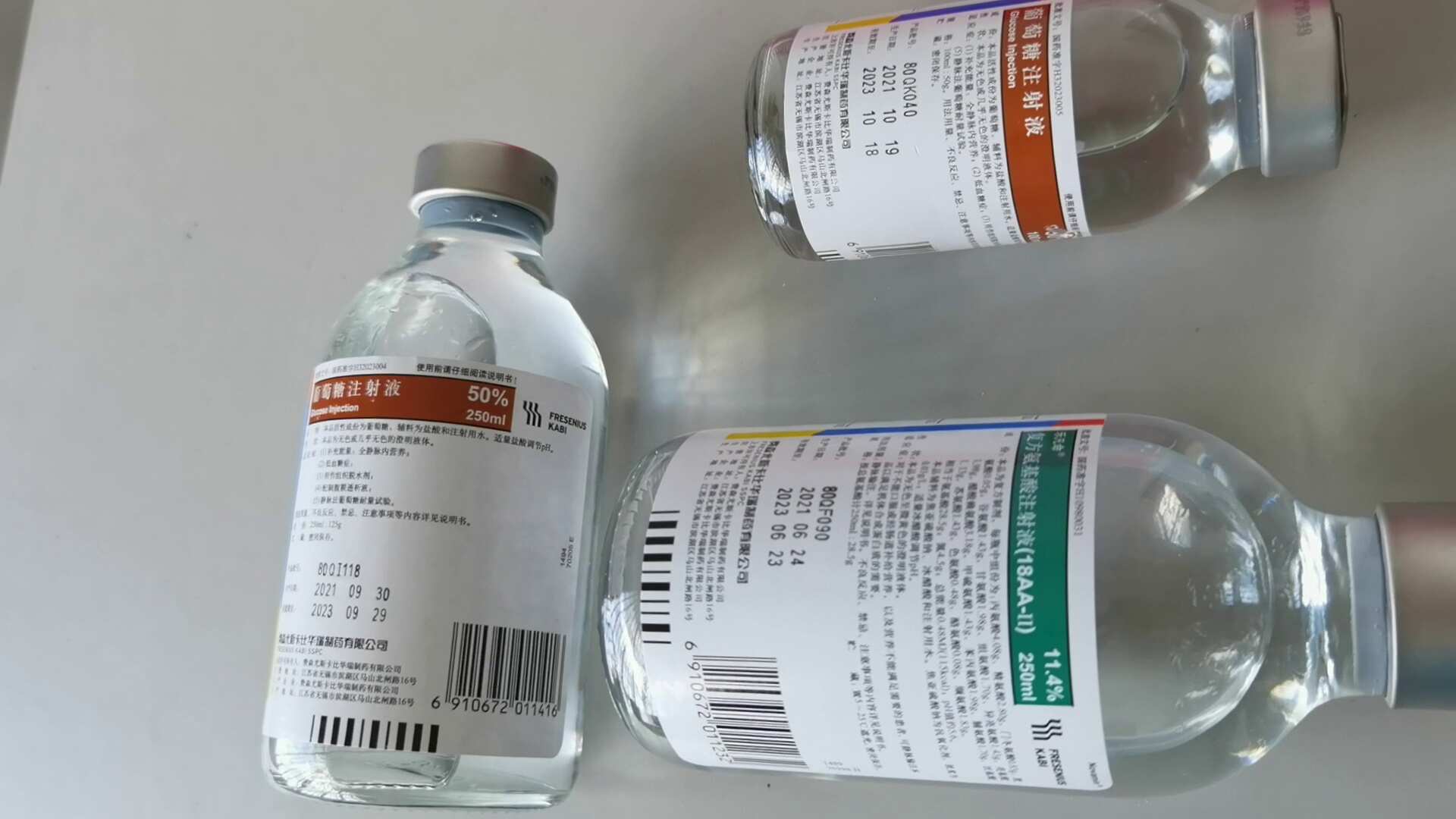

Supplement: S1 Dataset — (ZIP) [file pone.0298109.s001.zip › minimal data set/VOC2007/images/1074.jpg]

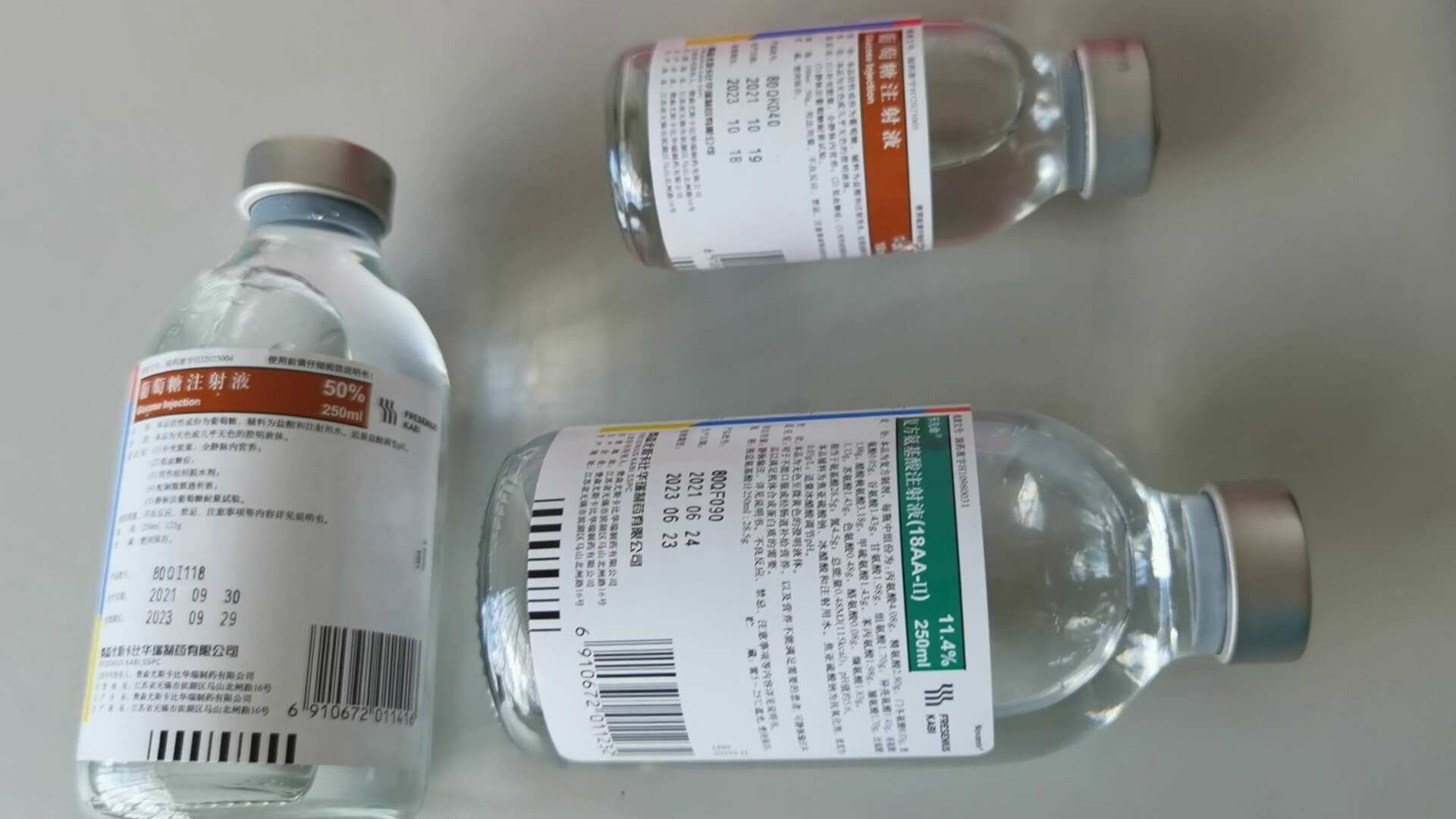

Supplement: S1 Dataset — (ZIP) [file pone.0298109.s001.zip › minimal data set/VOC2007/images/1075.jpg]

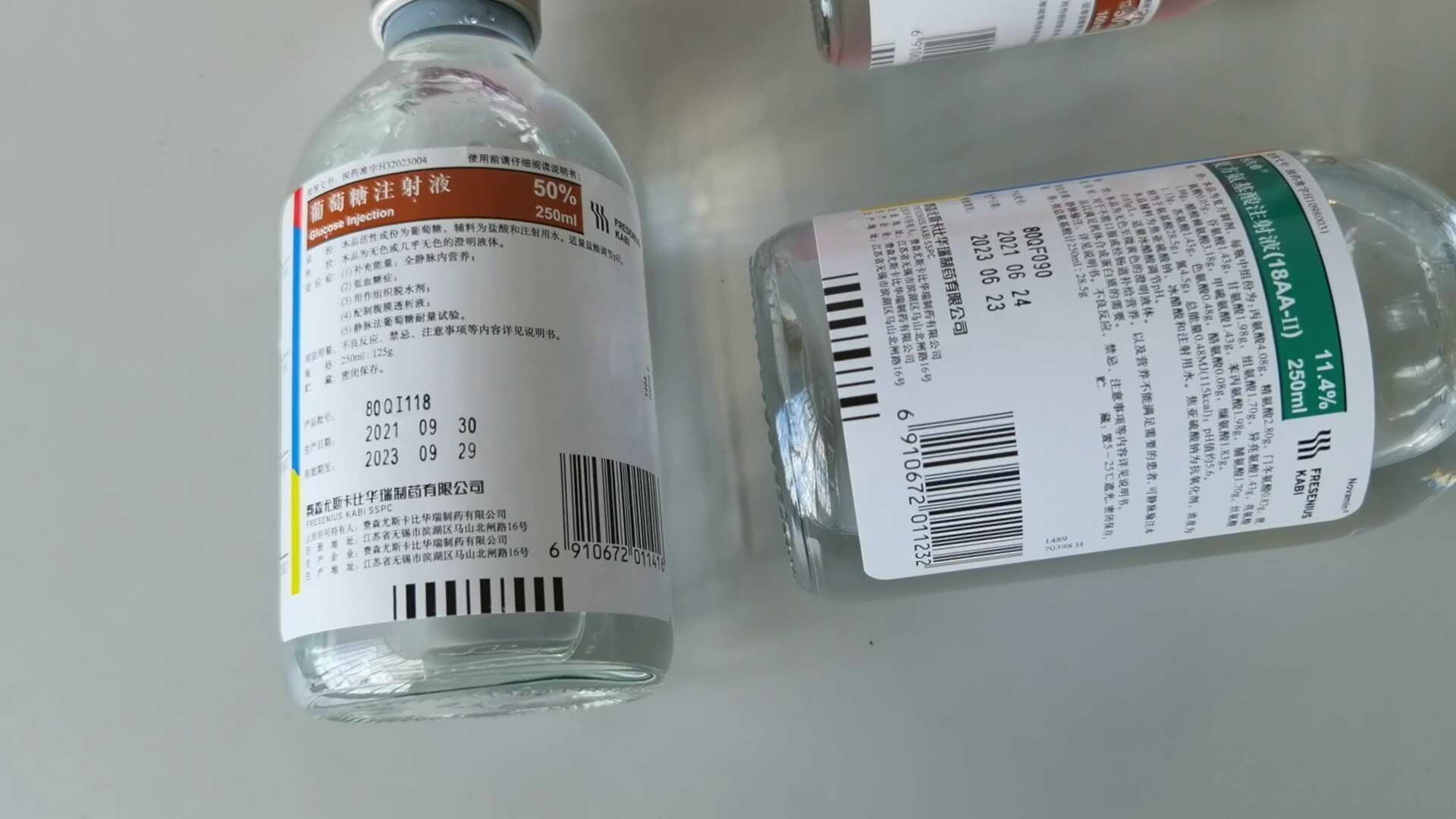

Supplement: S1 Dataset — (ZIP) [file pone.0298109.s001.zip › minimal data set/VOC2007/images/1076.jpg]

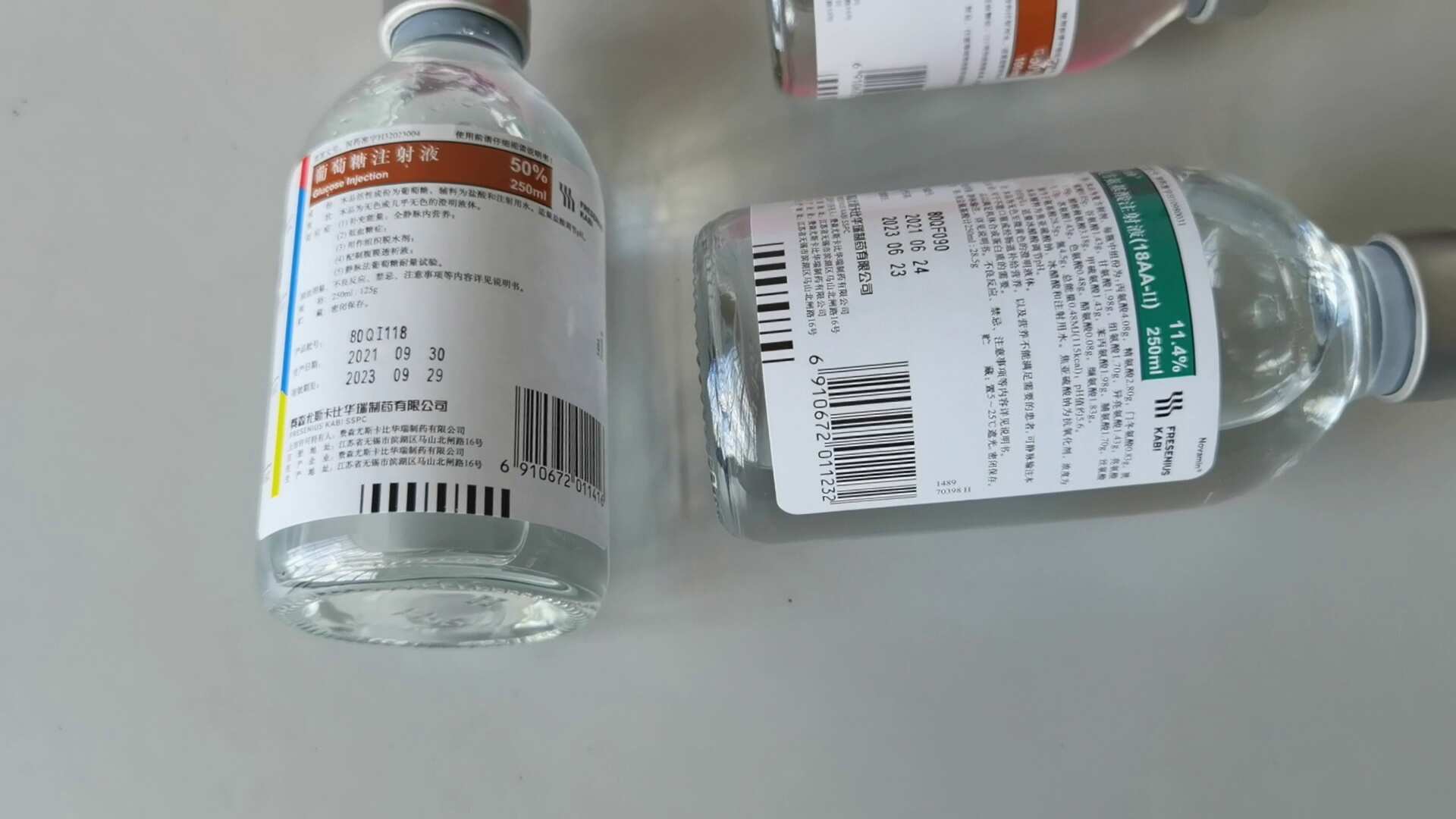

Supplement: S1 Dataset — (ZIP) [file pone.0298109.s001.zip › minimal data set/VOC2007/images/1077.jpg]

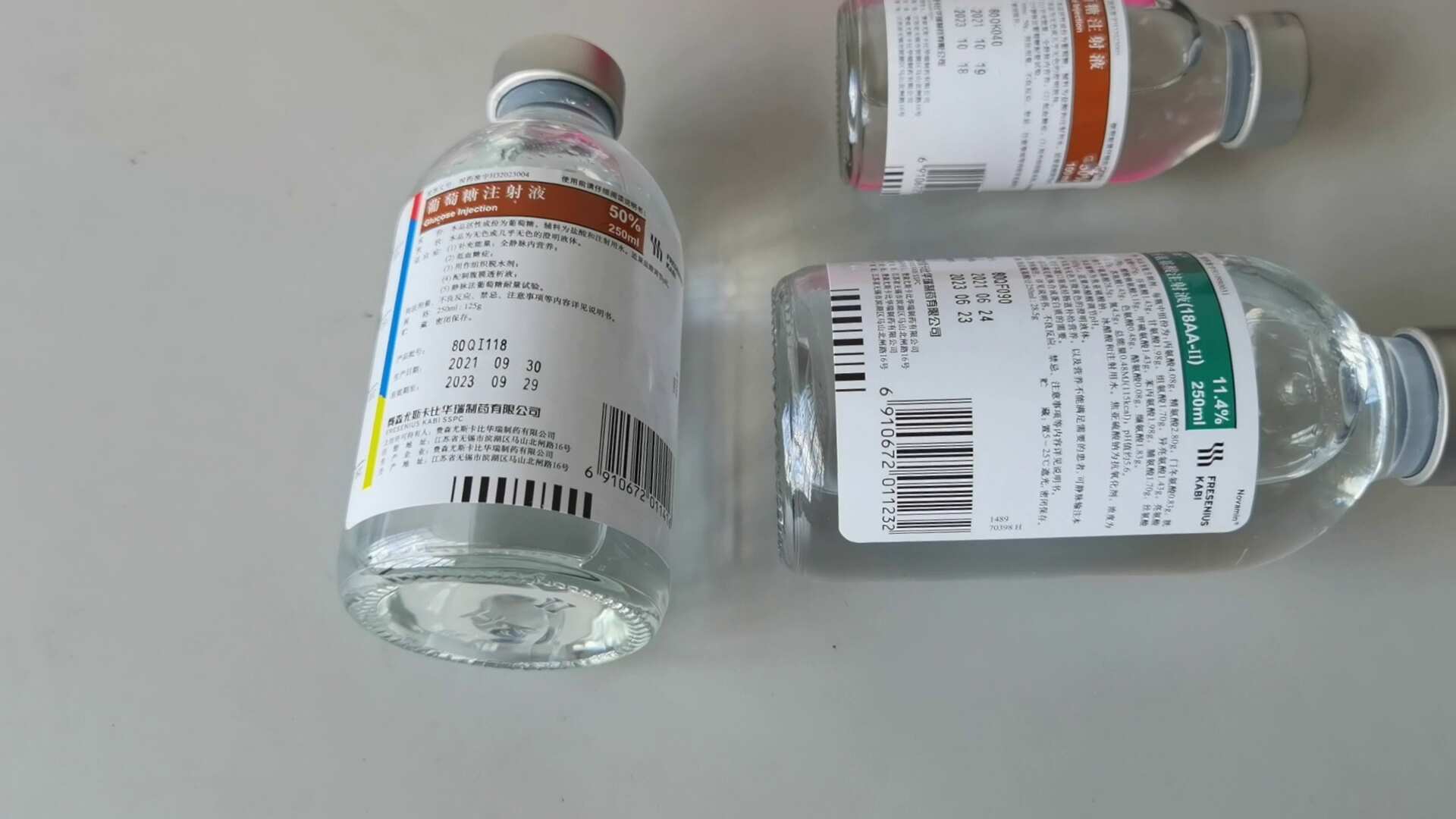

Supplement: S1 Dataset — (ZIP) [file pone.0298109.s001.zip › minimal data set/VOC2007/images/1078.jpg]

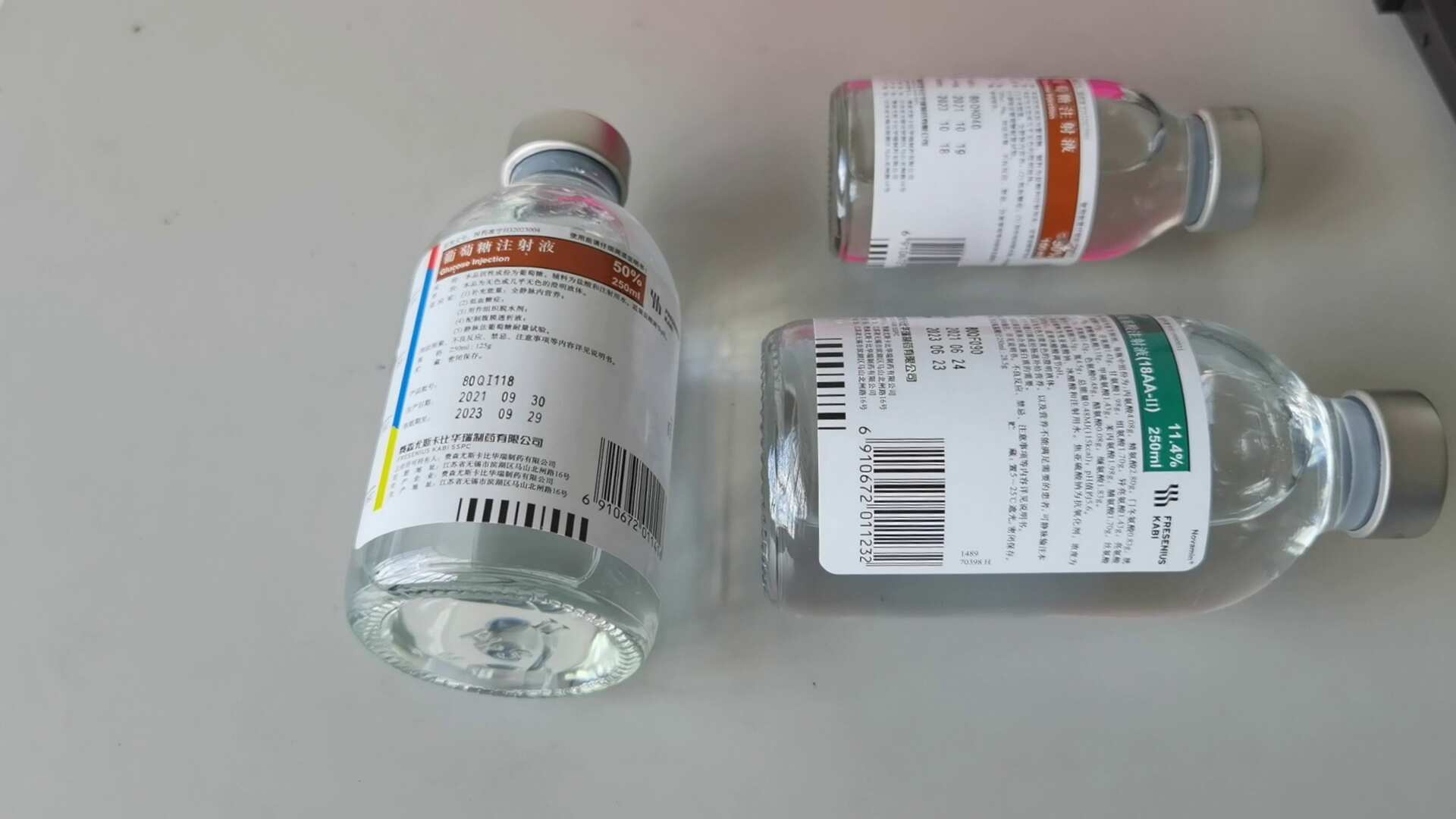

Supplement: S1 Dataset — (ZIP) [file pone.0298109.s001.zip › minimal data set/VOC2007/images/1079.jpg]

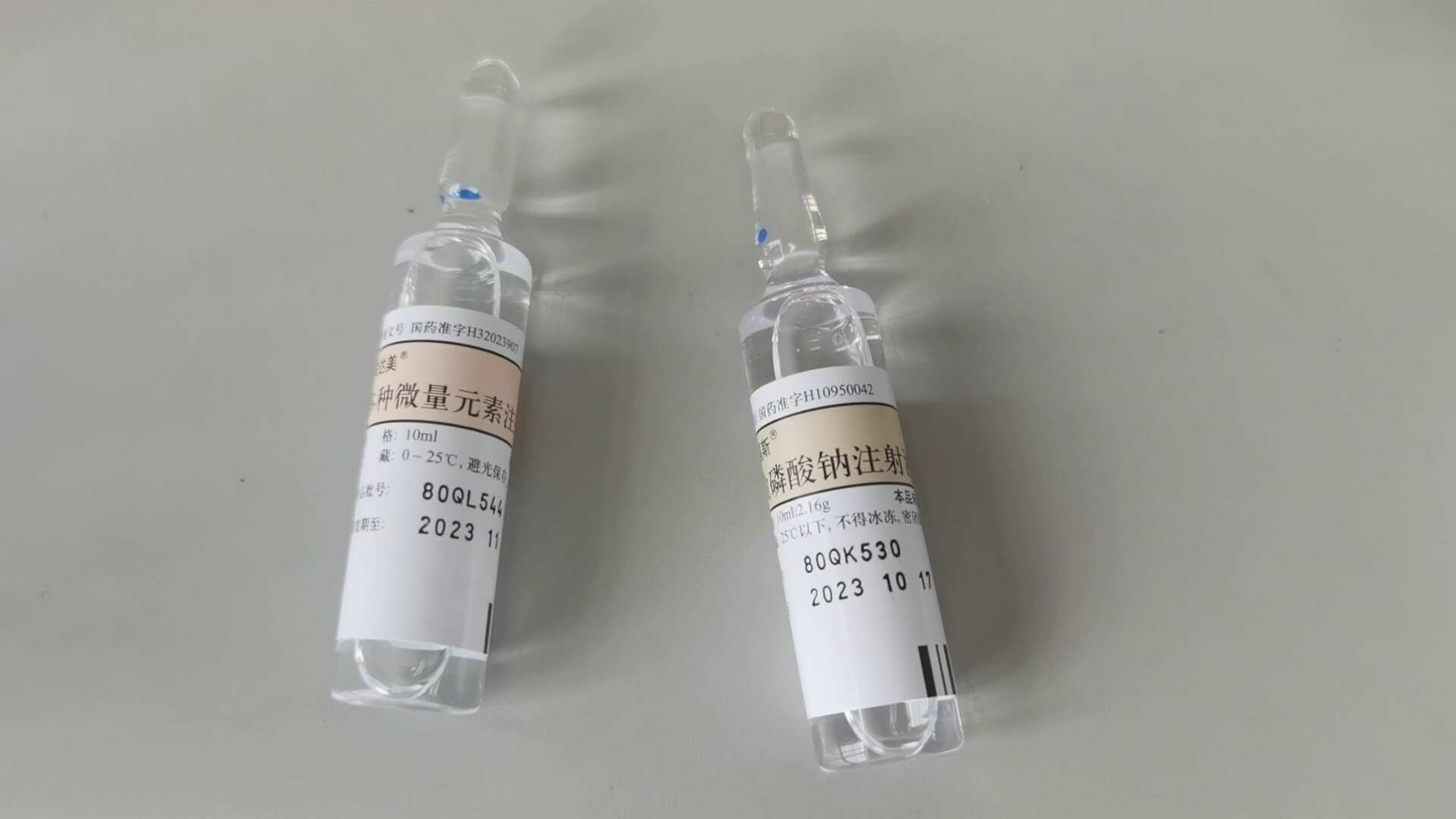

Supplement: S1 Dataset — (ZIP) [file pone.0298109.s001.zip › minimal data set/VOC2007/images/108.jpg]

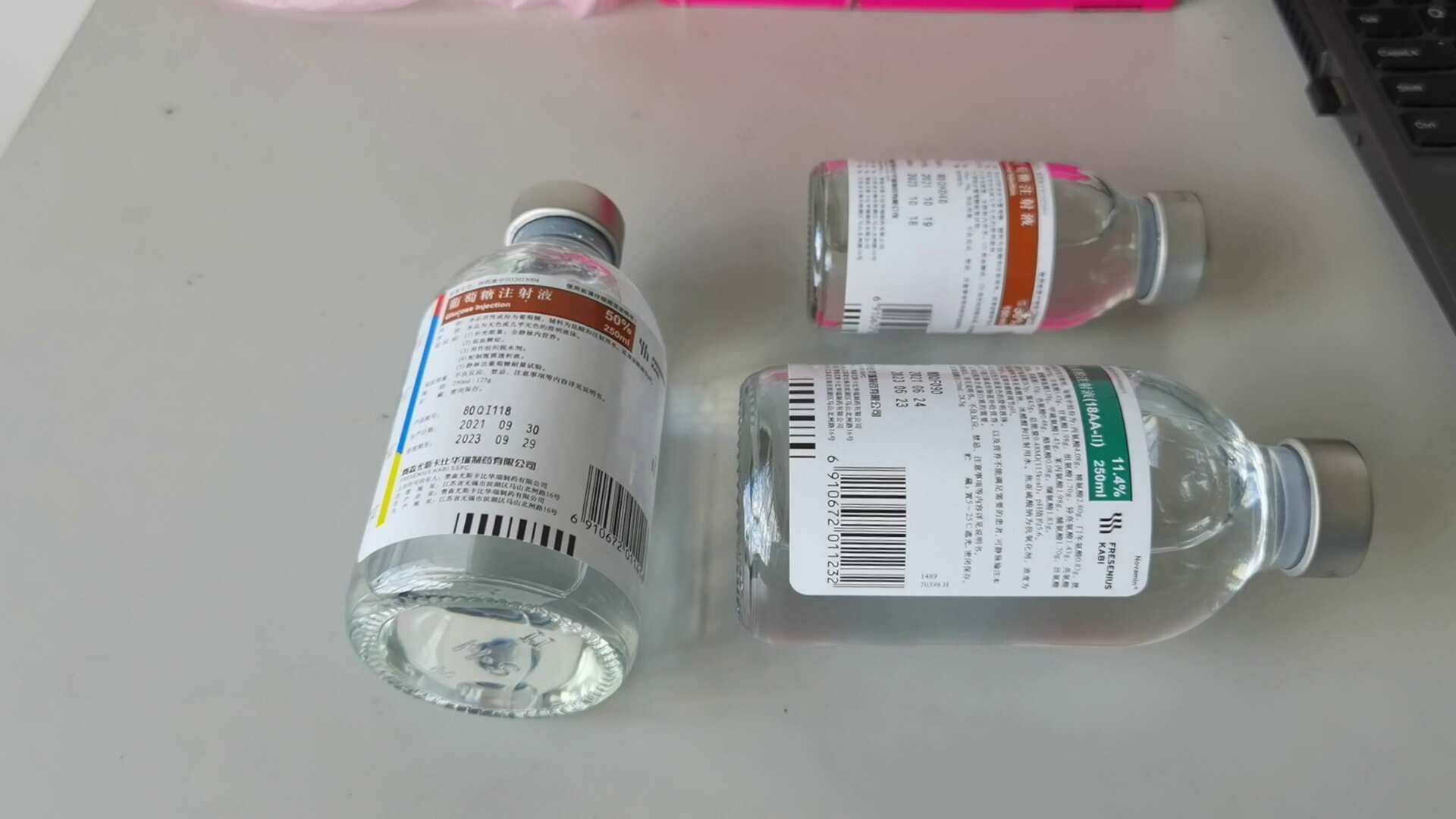

Supplement: S1 Dataset — (ZIP) [file pone.0298109.s001.zip › minimal data set/VOC2007/images/1080.jpg]

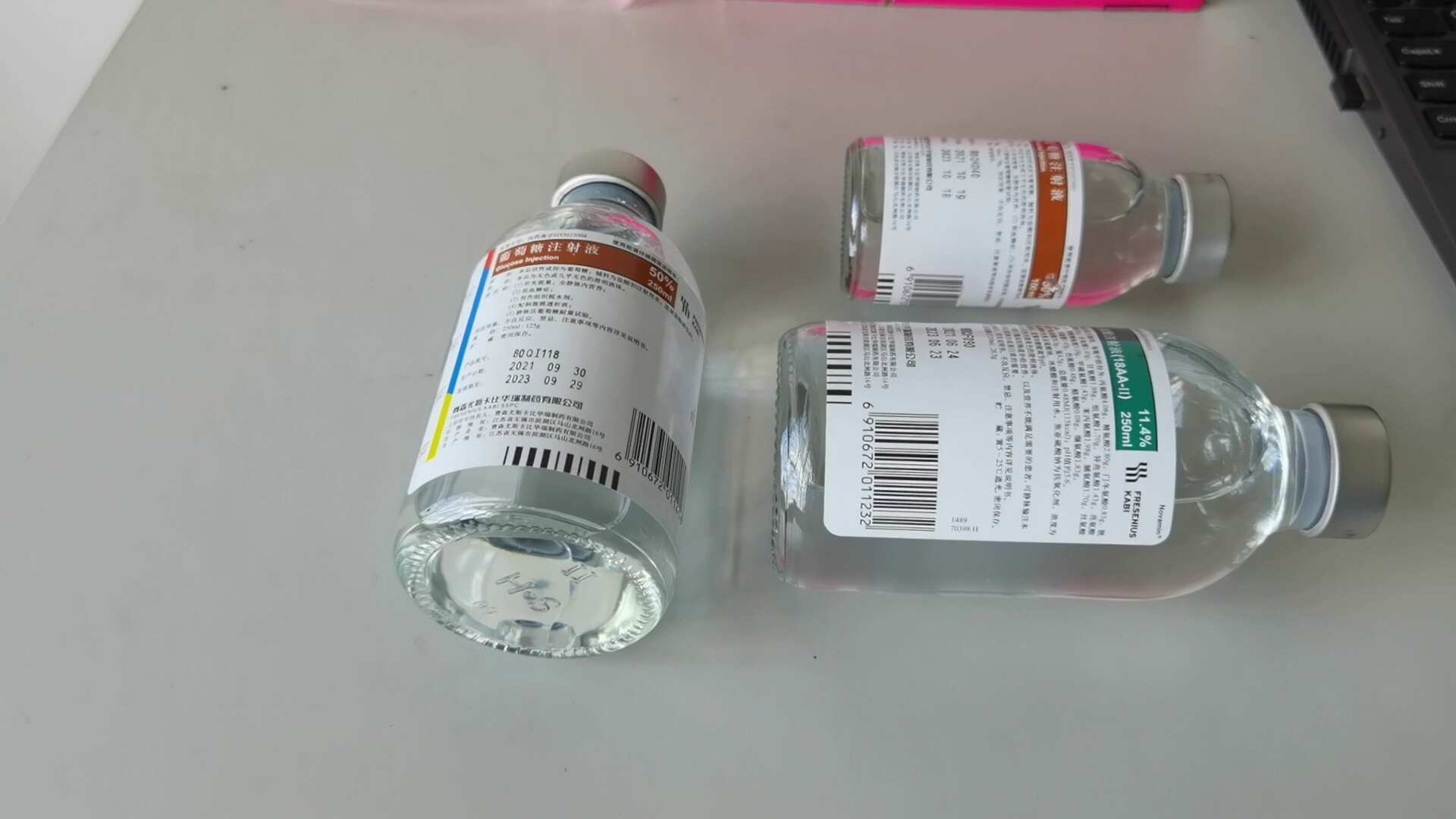

Supplement: S1 Dataset — (ZIP) [file pone.0298109.s001.zip › minimal data set/VOC2007/images/1081.jpg]

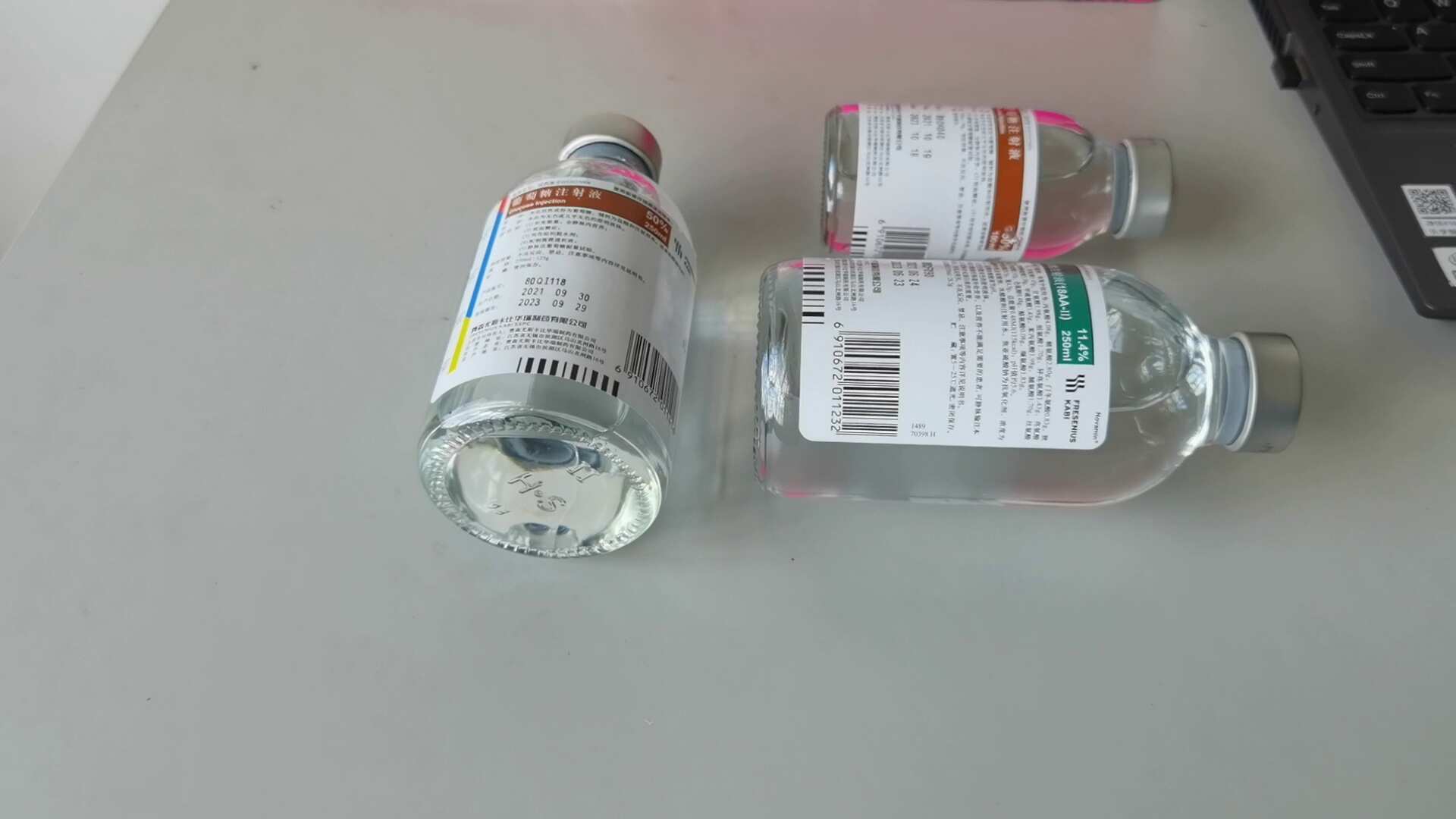

Supplement: S1 Dataset — (ZIP) [file pone.0298109.s001.zip › minimal data set/VOC2007/images/1082.jpg]

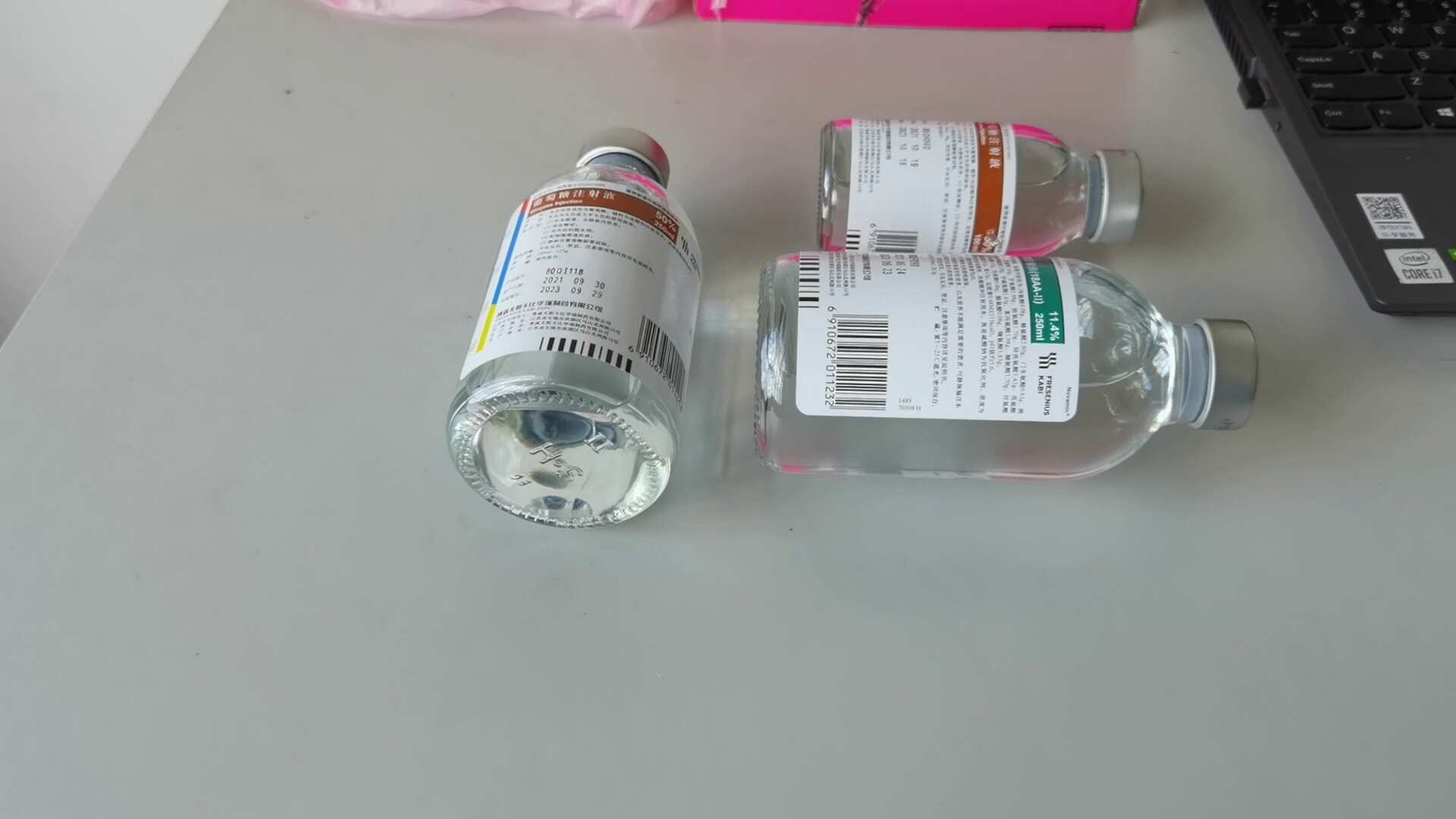

Supplement: S1 Dataset — (ZIP) [file pone.0298109.s001.zip › minimal data set/VOC2007/images/1083.jpg]

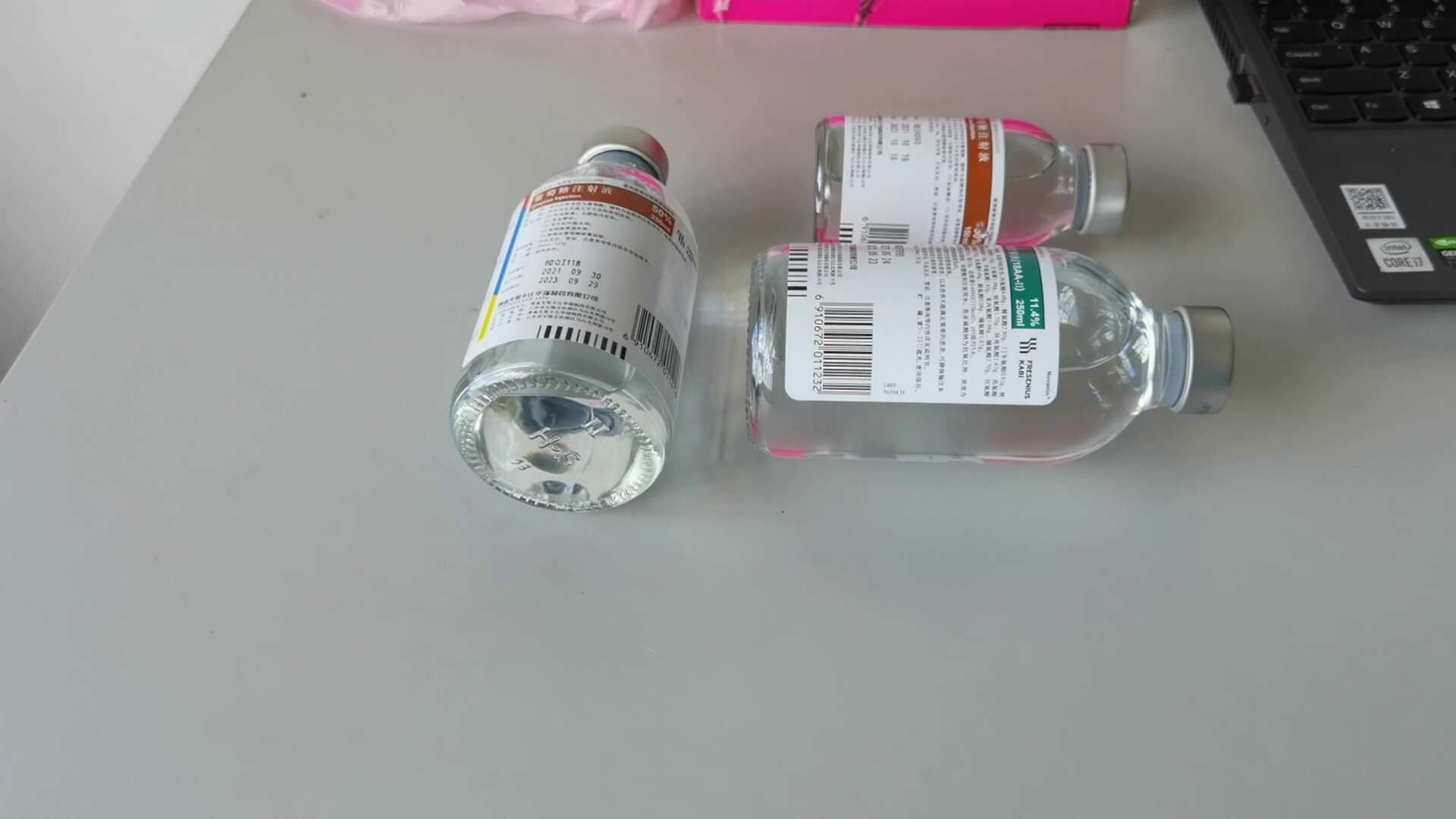

Supplement: S1 Dataset — (ZIP) [file pone.0298109.s001.zip › minimal data set/VOC2007/images/1084.jpg]

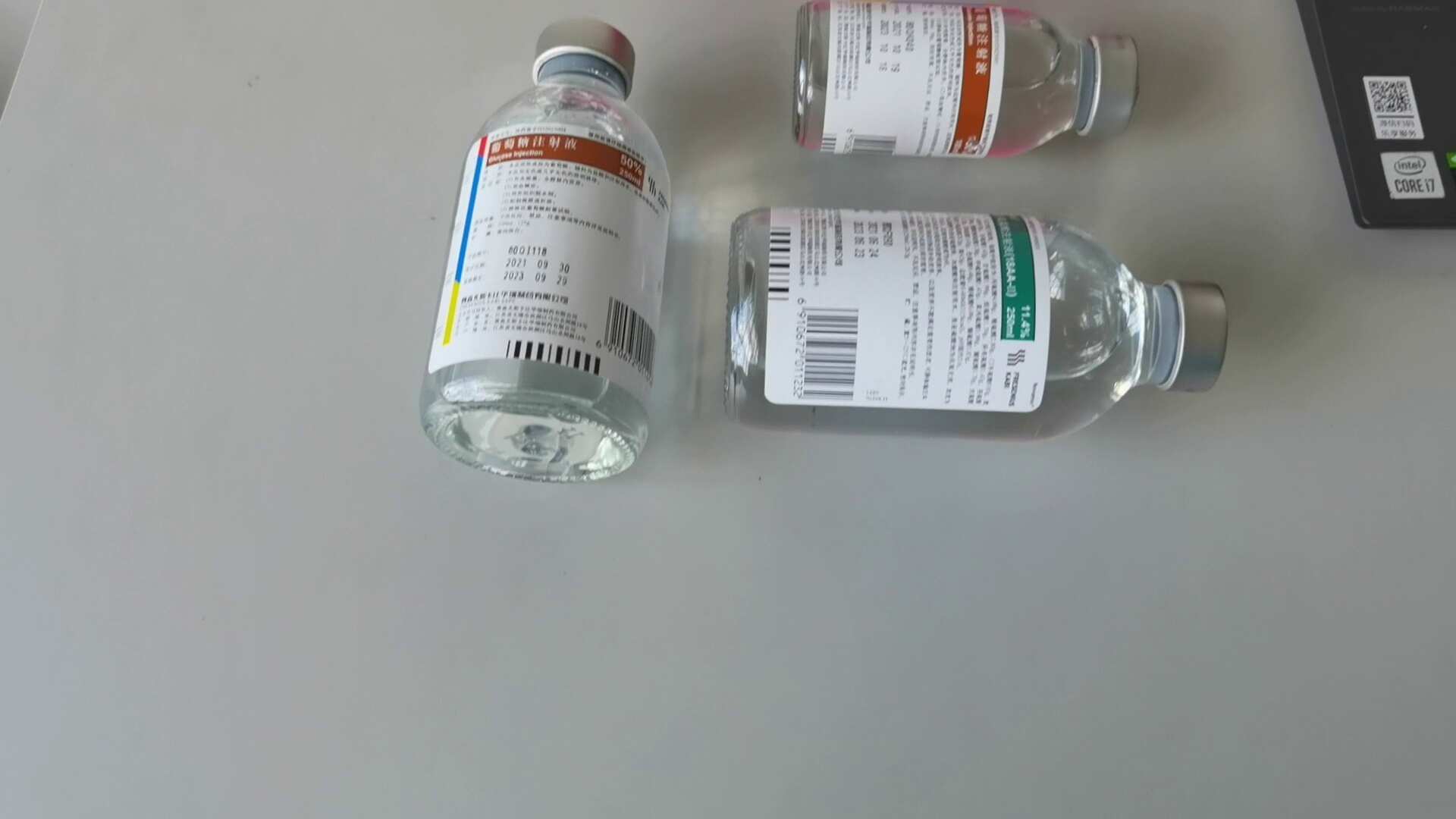

Supplement: S1 Dataset — (ZIP) [file pone.0298109.s001.zip › minimal data set/VOC2007/images/1085.jpg]

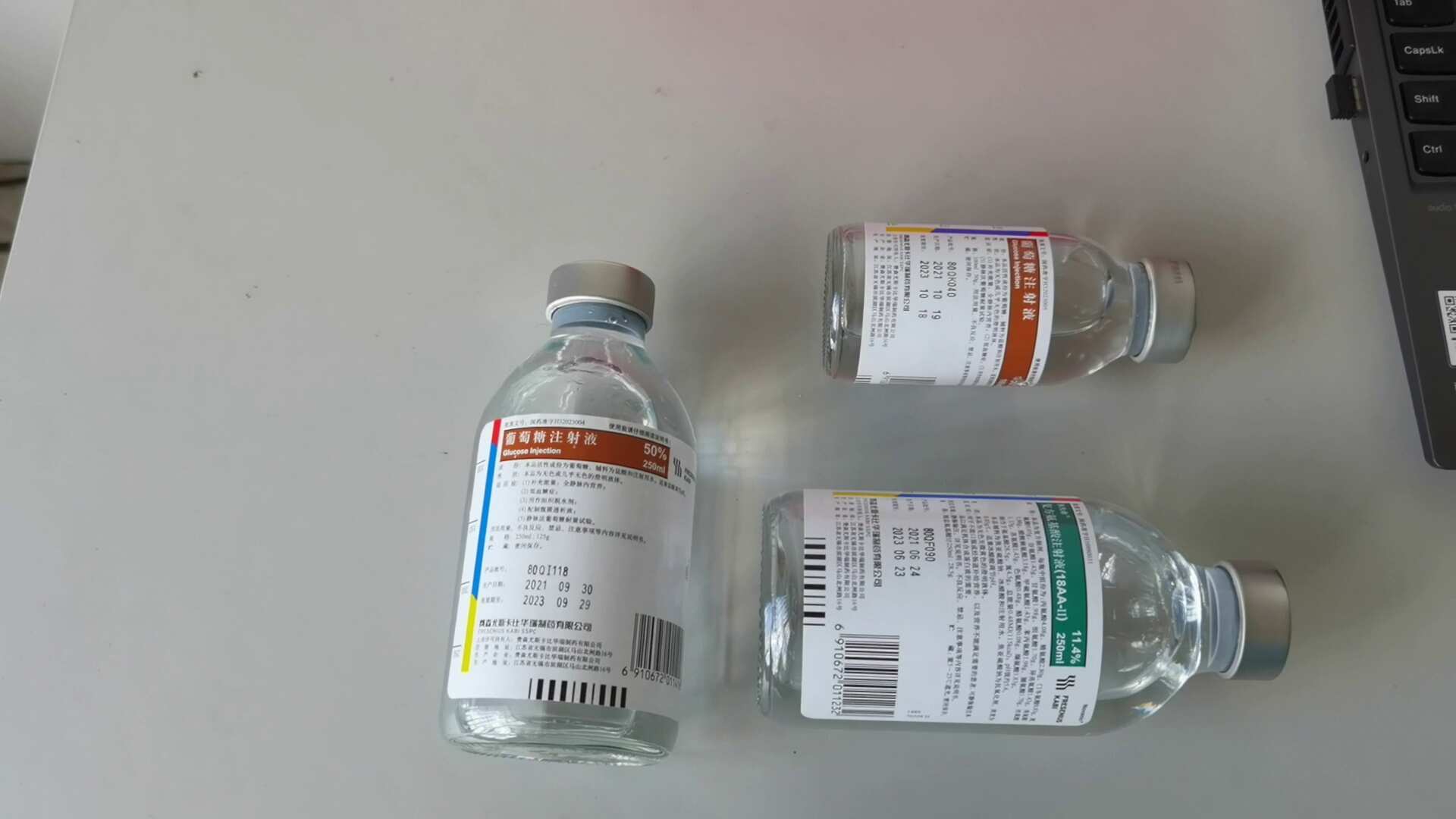

Supplement: S1 Dataset — (ZIP) [file pone.0298109.s001.zip › minimal data set/VOC2007/images/1086.jpg]

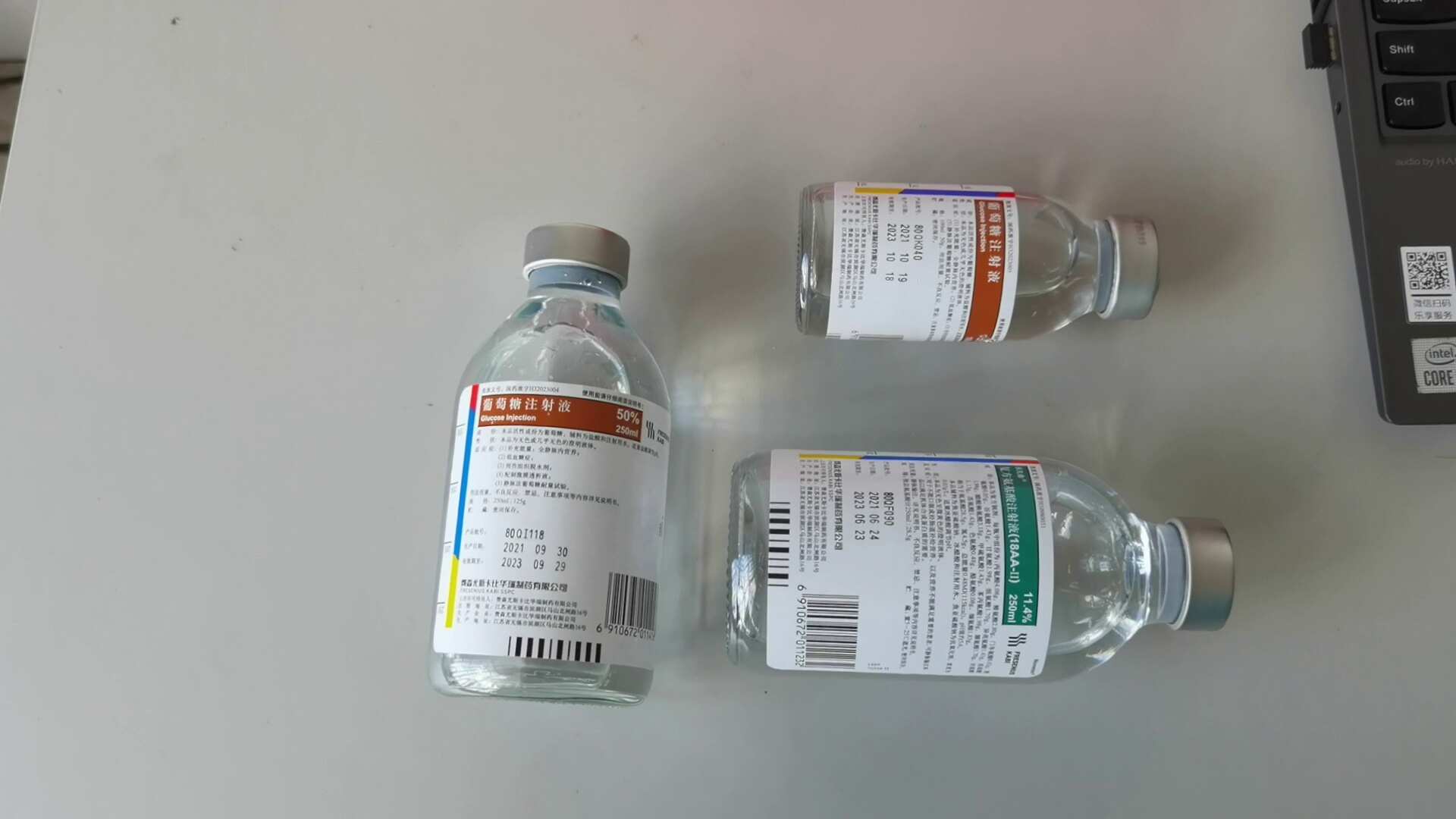

Supplement: S1 Dataset — (ZIP) [file pone.0298109.s001.zip › minimal data set/VOC2007/images/1087.jpg]

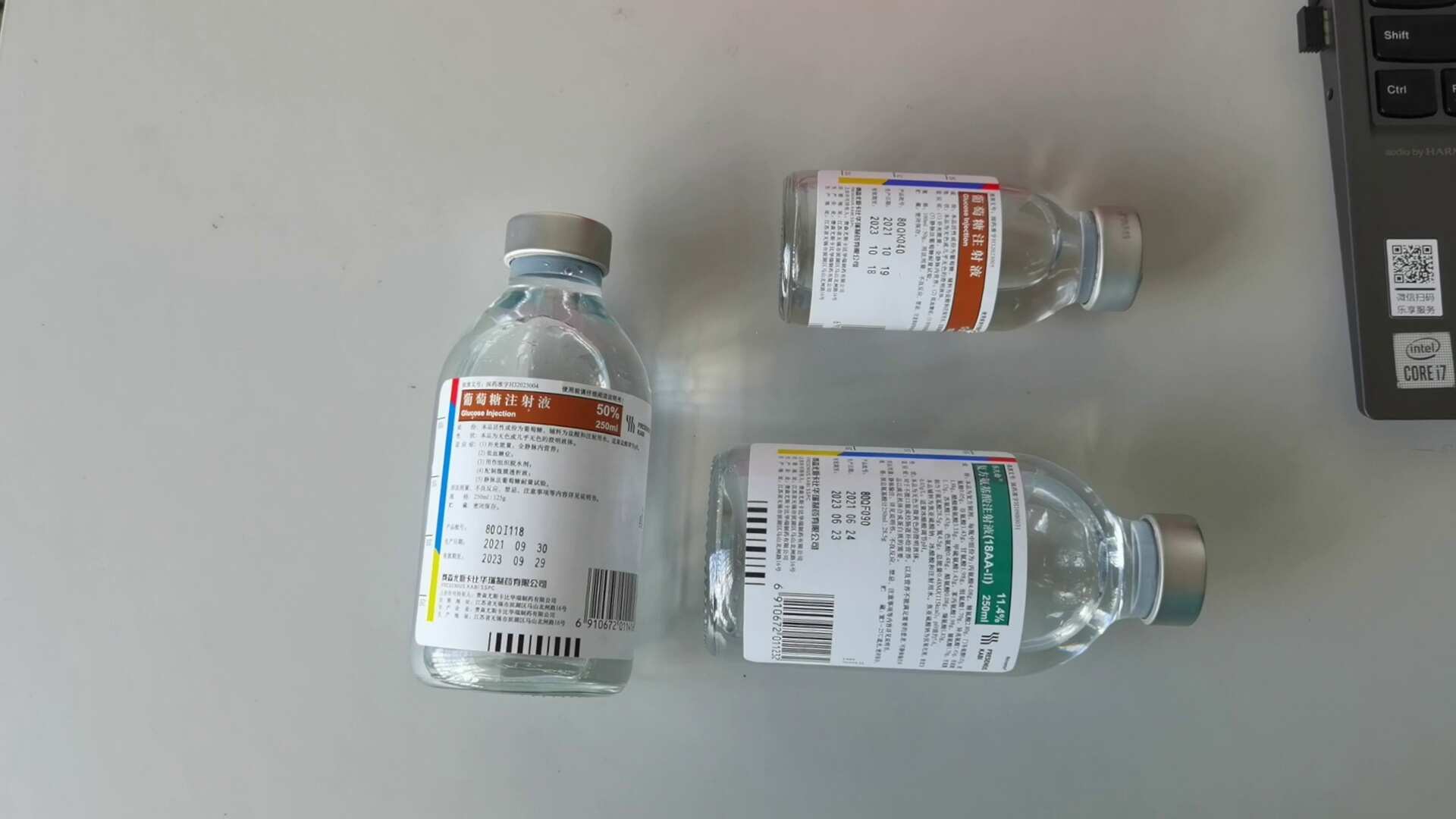

Supplement: S1 Dataset — (ZIP) [file pone.0298109.s001.zip › minimal data set/VOC2007/images/1088.jpg]
